# Personalized Strategy for Animal-Assisted Therapy for Individuals Based on the Emotions Induced by the Images of Different Animal Species and Breeds

**DOI:** 10.3390/ani12050597

**Published:** 2022-02-27

**Authors:** Jovita Luksaite, Egle Zokaityte, Vytaute Starkute, Sonata Sidlauskiene, Gintare Zokaityte, Elena Bartkiene

**Affiliations:** Institute of Animal Rearing Technologies, Faculty of Animal Sciences, Lithuanian University of Health Sciences, Mickeviciaus Str. 9, LT-44307 Kaunas, Lithuania; jovita.luksaite@stud.lsmu.lt (J.L.); egle.zokaityte@lsmuni.lt (E.Z.); vytaute.starkute@lsmuni.lt (V.S.); sonata.sidlauskiene@lsmuni.lt (S.S.); gintare.zokaityte@lsmuni.lt (G.Z.)

**Keywords:** animal species, animal breed, induced emotions, animal-assisted therapy, Likert scale

## Abstract

**Simple Summary:**

In this study, we hypothesized that the personalized strategies for the animal-assisted therapy (AAT) could be improved by selecting animal species and breeds for individuals (persons) according to the emotions induced in the persons by different animal species and breeds. To our knowledge, this study is the first in the area in which the FaceReader technique has been applied to improve the methodology of AAT, which could be the first step to avoiding and/or minimizing stressful situations during a person’s contact with an animal. To implement the aim, the images of different animal species (dogs, cats, pigs, sheep, and horses) and various breeds of those species were used. This study showed that the animal species is a significant factor in the intensity of the ‘neutral’ and ‘happy’ emotions as well as valence. In addition, animal breed is a significant factor in the intensity of the emotion ‘happy’ and valence. Finally, in this study, we obtained results that could be used as a personalized strategy for improvement of the AAT and to help the individuals select a pet.

**Abstract:**

The aim of this study was to apply the FaceReader technique to select the animal species and breed for a personalized AAT based on the emotions (‘neutral’, ‘happy’, ‘sad’, ‘angry’, ‘surprised’, ‘scared’, ‘disgusted’, and ‘contempt’) induced in the persons (18–64 years old) by the images of different animal species and breeds. To implement the aim, the images of different animal species (*Canis familiaris*, *Felis silvestris* *catus*, *Sus scrofa domesticus*, *Ovis aries*, and *Equus caballus*) and their breeds (dogs: Australian shepherd, pug, Labrador retriever, Doberman, miniature schnauzer, beagle, three mixed-breed types, Yorkshire terrier, Cane Corso, Samoyed, and Chihuahua; cats: British shorthair, Himalayan cat, three mixed breed types, Siamese cat, Sphynx, and Bengal cat; horses: Norwegian Fjord, Exmoor pony, Andalusian, and Friesian; pigs: Vietnamese pot-bellied and Kunekune; sheep: Herdwick sheep and Suffolk sheep) were used. This study showed that the animal species is a significant factor in the intensity of the emotions ‘neutral’ and ‘happy’ as well as valence, and the animal breed is a significant factor for the emotion ‘happy’ intensity and valence. The obtained results could be used as a personalized strategy for improving AAT and helping the individuals to select a pet.

## 1. Introduction

For many years, humans have known that animals can positively influence human health, but only recently has science begun to investigate the therapeutic effect of animals on individuals with various disorders [1,2]. AAT is the deliberate inclusion of an assisted animal in a treatment plan to realize specific goals [3]. Additionally, AAT, defined as a therapeutic intervention incorporating animals to improve health and wellness, is based on a human–animal interaction that includes the emotional, psychological, and physical interactions of people, animals, and the environment [4,5,6,7,8].

AAT is highly beneficial to human mental health as it can reduce anxiety, increase a sense of connection to a living being, reduce loneliness, and develop a variety of skills [3,9,10,11]. AAT is also used to minimize autistic spectrum symptoms [12]; improve medical conditions [13], including mental functioning [14]; reduce emotional difficulties [15]; and avoid undesirable behaviors [16] and physical problems [17].

AAT is implemented by a licensed practitioner [9,18], and, until now, some practical challenges have been associated with the AAT methodology, including the issues of process design [19]. For this reason, there is an increasing interest in improving the therapy’s efficacy through research [2]. One of the main challenges which remain in this area is to establish the conditions under which AAT can be most helpful. The influence of AAT on persons varies in relation with the animal used (e.g., dog, cat, pig, horse, etc.). In addition, the environment in which AAT is performed and the therapy duration are also influencing factors.

The published data of the AAT methodology indicate that research in this area is in its early stage [20]. It is essential to reassess the procedures of the assisted animal selection to update the methodologies in light of the new information about the changes in the role of animals and settings in which they work today [21]. Additionally, animals should be screened carefully for health and temperament [22]. Most of the studies focused on the selection of dogs and horses [21]. In several countries, farms operate as care farms in cooperation with the health authorities [23]. This practice is called Green Care or Farming for Health, a concept that is not restricted to the use of animals but also includes the effects of plants, gardens, forests, and landscapes [24].

In AAT, animals play a central role; for this reason, they have to be monitored to observe stress signals induced by therapeutic sessions [25]. In order to avoid situations in AAT which are stressful to both the person and animal, during the first step, a selection of the most appropriate animal-based images could be used to avoid situations which are stressful for the animal as well.

In this study, we hypothesized that the personalized strategies for AAT could be improved by selecting animal species and breeds for each individual (person), according to their emotions, as induced by different animal species and breeds. To our knowledge, this is the first study in this area in which the FaceReader technique has been applied for the improvement of the AAT methodology, which could be the first step to avoid and/or reduce stressful situations for persons during contact with an animal. In this study, the images of different animal species (dogs (*Canis familiaris*), cats (*Felis silvestris catus*), pigs (*Sus scrofa domesticus*), sheep (*Ovis aries*), and horses (*Equus caballus*)) and their breeds (dogs: 1.1. Australian shepherd, 1.2. pug, 1.3. Labrador retriever, 1.4. Doberman, 1.5. the miniature schnauzer, 1.6. beagle, 1.7. mixed breed, 1.8. Yorkshire terrier, 1.9. mixed breed, 1.10. mixed breed, 1.11. Cane Corso, 1.12. Samoyed, and 1.13. Chihuahua; cats: 2.1. mixed breed, 2.2. British shorthair, 2.3. Himalayan cat, 2.4. mixed breed, 2.5. mixed breed, 2.6. Siamese cat, 2.7. Sphynx, and 2.8. Bengal cat; horses: 3.1. Norwegian Fjord, 3.2. Exmoor pony, 3.3. Andalusian, and 3.4. Friesian; pigs: 4.1. Vietnamese pot-bellied and 4.2. Kunekune; and sheep: 5.1. Herdwick sheep and 5.2. Suffolk sheep) were tested.

Dogs are the most commonly used animals in AAT [2]. Their popularity is related to their ease of domestication, access, and training, as well as their specific behavioral characteristics, including affection towards humans, which is higher than that of other animals. Therapy dogs provide health benefits for individuals, read humans’ non-verbal body language, and react/respond to their emotions [26].

In addition to dogs, cats have become a popular companion animal for humans with positive effects on the pet owner’s health [27,28,29,30,31]. It was reported that the factors related to the quality of dog–owner relationships differ from those of cat–owner relationships [32]. From this point of view, the tools used to study the human–animal relationships need to be customized by species [28].

Horses are among the most important domesticated animals, and their number is steadily increasing [33]. Today, horses play an essential role in AAT [34,35,36,37,38,39,40,41,42]. In addition to physical benefits, the proximity to horses positively impacts emotional, social, and mental health [35,43] and increases the quality of the person’s and/or patient’s life [44]. Despite horses demonstrating a therapeutic effect on humans [45,46], it was reported that persons may tend to avoid this therapy because of their fear of horses [39]. From this point of view, as suggested in this study, a personalized strategy can help prevent unnecessary stress for patients before the AAT selection.

In addition to in-house pets and horses, AAT with farm animals has many benefits [23,47,48,49,50]. For example, pigs are incredibly intelligent and sensitive animals, making them good candidates for AAT [51].

During the last decade, the presence of animals in institutional care settings has grown. For this reason, different concepts of animal intervention have been created, so-called animal-assisted interventions (AAI) [52,53]. This strategy includes both AAT and animal-assisted activities (AAA); the latter refers to a general category of interventions without a protocol [53]. Finally, interacting with animals can lead to feelings of safety and comfort and allow individuals to express affection [52,54,55]. However, disgust as well as fear of animals could also cause concerns for individuals [56,57].

The aim of this study was to apply a FaceReader technique in selecting the animal species and breed for a personalized AAT, according to the emotions induced in each person by the images of different species and breeds.

## 2. Materials and Methods

### 2.1. Evaluation of the Emotions Induced by Different Animal Species and Breeds in Individuals

**Figure 1:**
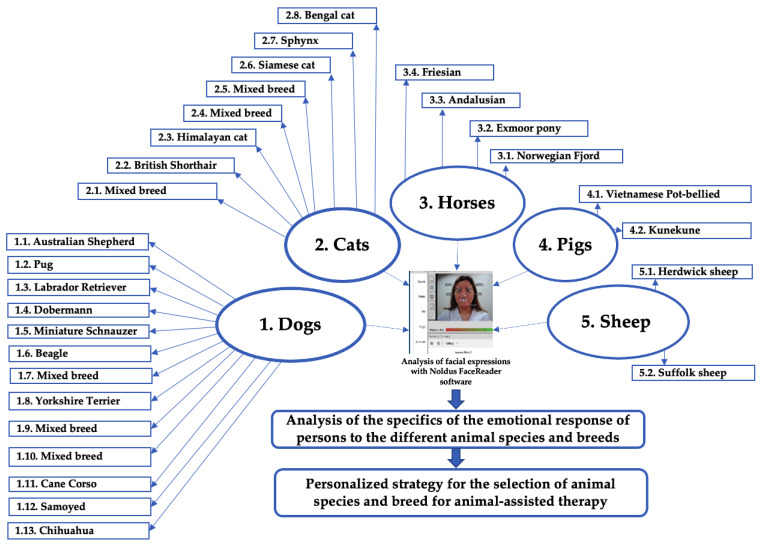
Principal scheme of the experiment.

The FaceReader technique (Noldus Information Technology, Wageningen, the Netherlands) was used to evaluate possible differences in the human emotions induced by different animal species and breeds. The principal scheme of the experiment is shown in Figure 1. The study tested one group of participants. The demographic data of the studied group are given in Table 1. This study involved 50 participants (not diagnosed with a mental disorder for at least a one-year period) aged between 18 and 64 years, where 82% were females and 18% were males. Most of the participants (66%) held a university degree, and 34% had secondary education. Concerning civil status, 28% of the participants were single, 54% were married or lived together as a marital couple, 14% were separated or legally divorced, and 4% were widowed. Regarding occupation, 4% of the participants were students, 74% were employed, 12% were working students, and 10% were retired. Concerning the participants’ professional activity and/or study field, the majority of the participants (72%) were engaged in the field of education, 4% in health, 2% in law and public policy, 2% in community and social services, and 20% in agriculture.

A FaceReader-6 software was used for the analysis of the participants’ facial expressions. The acceptability of various animal species and breeds was assessed using a 10-point Likert scale from 0 (dislike extremely) to 10 (like extremely).

During the study, the images of different animal species and breeds (images showing the classic characteristics and shape of animals, collected from accessible internet sources) were presented to the person showing the cards one-by-one. Simultaneously, the participant was provided with information about the main character traits of the seen animal species and breed (Supplementary File S1). After seeing each card, the person was asked to rank their likability towards the shown animal on the Likert scale from 0 (dislike extremely) to 10 (like extremely). No timer was used for the session. The procedure was recorded with the Microsoft LifeCam Studio webcam mounted on the laptop and focused on the participant, using Media Recorder software (Noldus Information Technology, Wageningen, the Netherlands). The recordings were analyzed with the mentioned FaceReader-6 software, which recognizes eight basic patterns of human emotions (‘neutral’, ‘happy’, ‘sad’, ‘angry’, ‘surprised’, ‘scared’, ‘disgusted’, and ‘contempt’). The emotion ‘surprised’ can be either positive or negative. ‘Happy’ is the only positive emotion, whereas ‘sad’, ‘angry’, ‘scared’, and ‘disgusted’ are considered negative emotions.

In addition, FaceReader-6 also analyzed the valence, which indicates whether a person’s emotional state is positive or negative. Valence is calculated as the intensity of ‘happy’ minus the intensity of negative emotion with the highest intensity. The tested valence scores ranged from −1 to 1.

For each card, the section of intentional facial expression (precisely from the point when the subject had finished raising the hand to give a signal until the subject started lowering the hand again) was extracted and used for the statistical analysis, in which the facial expression patterns’ maximum values of the respective section were used (Supplementary File S2).

### 2.2. Statistical Analysis

For investigating the emotions induced by different animal species and breeds, 50 adult subjects were tested. The data were prepared and analyzed using Microsoft Excel (Microsoft Corp., Redmond, WA, USA) and SPSS Version 24 software (SPSS Inc., Chicago, IL, USA). We used a multivariate ANOVA analysis to evaluate the impact of different animal species and breeds on evoked emotions and calculated the Pearson correlations (r) between the intensity of the emotions induced in each individual and the Likert scale results. The strength measured by the Pearson correlation coefficient was interpreted, according to Evans [58]. The statistical differences with *p* values ≤0.05 and ≤0.01 were considered significant, and, using the Fisher’s least significant difference (LSD) post hoc test, the mean values were compared.

### 2.3. Ethical Approval

The protocol of this study was approved by the Lithuanian University of Health Sciences (Kaunas, Lithuania) Bioethics Committee (No. BEC-GZS(M)-03). All test participants were informed about the study using a ‘Personal Information Form’. The individuals were included in the study upon their participation agreement and signing an ‘Informed Consent Form’.

## 3. Results

### 3.1. Emotions Induced in the Individuals by Different Animal Species

**Figure 2:**
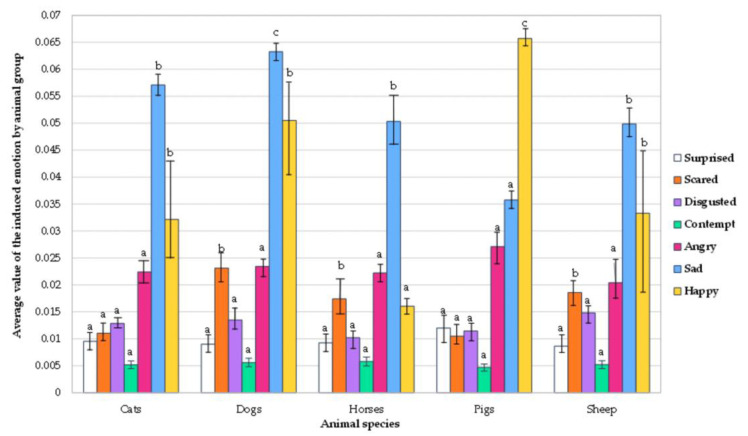
Average values of induced emotions for individuals by different animal species (^a–c^: the mean values in the columns of the same color marked with different letters are significantly different (*p* ≤ 0.05)).

The average intensity values of the emotions induced by different animal species are shown in Figure 2. The highest intensity of the emotion ‘happy’ was evoked when presenting pig images and behavior characteristics (on average, 0.066) to the individuals. Compared with other animal species, pigs induced a higher intensity of the ‘happy’ emotion than cats, dogs, horses, and sheep (51.5%, 24.2%, 75.8%, and 50.0%, respectively). Regarding the emotion ‘sad’ evoked by different animal species, the highest emotion intensity was observed upon seeing the images of dogs (0.063), and the lowest when seeing pig images, i.e., 44.4% lower than the dog image-induced emotion results. All tested animal species induced the emotion ‘angry,’ on average, 0.023, and no significant differences between the intensities of this emotion evoked by different animal species were found. The ‘contempt’ emotion induced by all tested animal species was, on average, 0.005, and no significant differences between the intensities of this emotion, as influenced by different animal species, were found. All tested animal groups evoked the intensity of the emotion ‘surprised,’ on average, 0.01, and there were no significant emotion intensity differences between the groups. Similarly, no significant differences were found between the intensity of the emotion ‘disgusted’ evoked by various animal species—on average, the intensity of ‘contempt’ was 0.012. The highest intensity of the emotion ‘scared’ was observed when seeing the images of sheep, horse, and dog groups (0.020), whereas the cat and pig groups induced, on average, an intensity of the emotion ‘scared’ two times lower.

**Figure 3:**
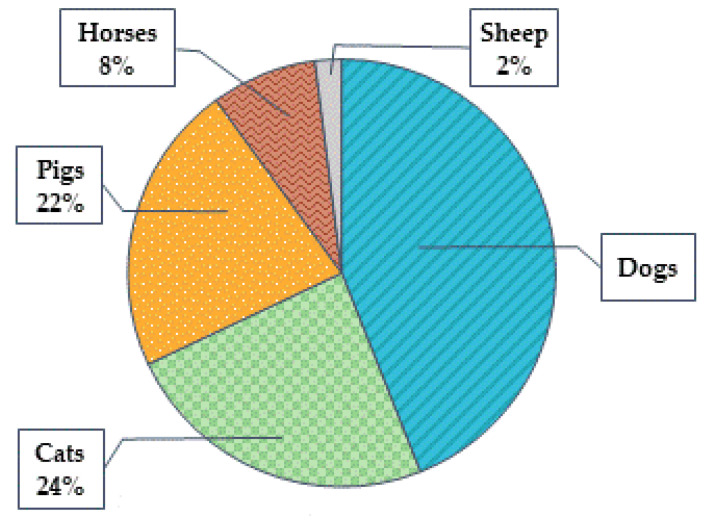
Percentage of the participants with the maximum valence induced by different animal species.

The percentage of the participants with maximum valence results induced by different animal species is shown in Figure 3. We observed the highest valence results in 22 test participants out of 50 when presenting dog group; in 12 when demonstrating the cat group; in 11 when showing pig group; and in 4 and 1 when seeing the horse and sheep groups, respectively.

### 3.2. Emotions Induced in the Participants by Different Animal Species and Breeds

**Figure 4:**
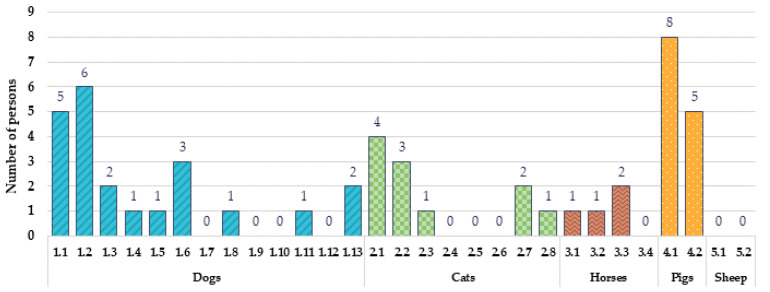
Number of participants with the maximum emotion ‘happy’ induced by different animal species and breeds (dogs: 1.1. Australian shepherd, 1.2. pug, 1.3. Labrador retriever, 1.4. Doberman, 1.5. miniature schnauzer, 1.6. beagle, 1.7. mixed breed, 1.8. Yorkshire terrier, 1.9. mixed breed, 1.10. mixed breed, 1.11. Cane Corso, 1.12. Samoyed, and 1.13. Chihuahua; cats: 2.1. mixed breed, 2.2. British shorthair, 2.3. Himalayan cat, 2.4. mixed breed, 2.5. mixed breed, 2.6. Siamese cat, 2.7. Sphynx, and 2.8. Bengal cat; horses: 3.1. Norwegian Fjord, 3.2. Exmoor pony, 3.3. Andalusian, and 3.4. Friesian; pigs: 4.1. Vietnamese pot-bellied and 4.2. Kunekune; sheep: 5.1. Herdwick sheep and 5.2. Suffolk sheep).

The number of participants with the maximum emotion ‘happy’ induced by different animal species and breeds is shown in Figure 4. Dog breeds 1.2. and 1.1. induced the ‘happy’ emotion in 12% and 10% of the participants, respectively. Additionally, dog breed 1.6. induced the maximum of the ‘happy’ emotion in 6% of the participants. Two out of thirteen of the evaluated dog images (1.3. and 1.13.) induced the maximum emotion ‘happy’ intensity in 4% of the participants, and four of the thirteen evaluated dog images (1.4., 1.5., 1.8., and 1.11.) induced the maximum emotion ‘happy’ intensity in 2% of the participants. In comparison, the cat breed 2.1. induced the maximum ‘happy’ emotion in 8% of the total participants. Additionally, breed 2.2. induced the maximum intensity of the ‘happy’ emotion in 6% of the participants, while the breeds 2.7., 2.3., and 2.8. induced the maximum ‘happy’ emotion intensity in 4%, 2%, and 2% of the participants, respectively. Regarding horse breeds, the breed 3.3. induced the maximum ‘happy’ emotion in 4% of the participants, while the breeds 3.1. and 3.2. induced the maximum intensity ‘happy’ emotion in 2% of the participants. Both of the tested pig breed images showed highly positive results, as the breed 4.1. induced the maximum emotion ‘happy’ intensity in 16% of the participants and the breed 4.2. induced the maximum emotion ‘happy’ intensity in 10% of the participants. However, both of the tested sheep images induced a lower intensity of the ‘happy’ emotion, and a maximum intensity by the tested sheep breeds was not observed.

**Figure 5:**
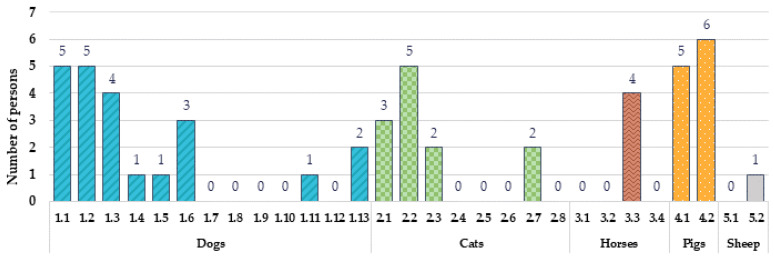
Number of the participants with the maximum valence induced by different animal species and breeds (dogs: 1.1. Australian shepherd, 1.2. pug, 1.3. Labrador retriever, 1.4. Doberman, 1.5. miniature schnauzer, 1.6. beagle, 1.7. mixed breed, 1.8. Yorkshire terrier, 1.9. mixed breed, 1.10. mixed breed, 1.11. Cane Corso, 1.12. Samoyed, and 1.13. Chihuahua; cats: 2.1. mixed breed, 2.2. British shorthair, 2.3. Himalayan cat, 2.4. mixed breed, 2.5. mixed breed, 2.6. Siamese cat, 2.7. Sphynx, and 2.8. Bengal cat; horses: 3.1. Norwegian Fjord, 3.2. Exmoor pony, 3.3. Andalusian, and 3.4. Friesian; pigs: 4.1. Vietnamese pot-bellied and 4.2. Kunekune; sheep: 5.1. Herdwick sheep and 5.2. Suffolk sheep).

The number of the participants with the maximum valence induced by different animal species and breeds are shown in Figure 5. Dog breeds 1.1. and 1.2. induced maximum valence in 10% of the participants. Breed 1.3. induced the maximum valence in 8% of the participants, breed 1.6. induced it in 6%, and breed 1.13. in 4%. Breeds 1.11., 1.4., and 1.5 induced the maximum valence in 2% of the participants. In comparison, cat breed 2.2. induced the maximum valence in 10% of the participants. Additionally, breed 2.1. induced the maximum valence in 6% of the participants, and the breeds 2.3. and 2.7. induced it in 4%. Only breed 3.3. out of the four horse breeds evaluated in this experiment induced the maximum valence in 8% of the participants. The most positive results were obtained with pig testing. From the two evaluated pig breed images, breed 4.1. induced the maximum valence in 10% of the participants, and breed 4.2. induced it in 12%. Finally, from the two evaluated sheep breed images, only breed 5.2. induced the maximum valence in 2% of the participants.

### 3.3. Influence of Different Animal Species and Breeds on the Emotions Induced in Individuals

The significance of the analyzed factors (animal species and breed) and their interaction with the emotions induced in individuals are shown in Table 2 (Supplementary File S3). It was found that the animal species is a significant factor on the intensity of the ‘neutral’ and ’happy’ emotions (*p* = 0.0001) as well as valence (*p* = 0.006). In addition, the animal breed was a significant factor on the intensity of the ‘happy’ emotion (*p* = 0.0001) and valence (*p* = 0.001). However, the interaction of the analyzed factors was not significant in the induced emotions’ intensity or valence.

Pearson correlations between the intensity of the emotions induced in individuals and the Likert scale results are shown in Table 3. Two very weak correlations between the Likert scale results and the emotions induced in the participants by the images of animal species and breeds were found to be significant (between the Likert scale results and the emotions ‘surprised’ and ‘disgusted’, r = −0.084 and r = 0.078, respectively). Weak negative correlations were established between the ‘neutral’ emotion and the emotions of ‘sad’, ‘scared’, and ‘disgusted’ (r = −0.353, *p* = 0.0001; r = −0.287, *p* = 0.0001; r = −0.207, *p* = 0.0001, respectively), as well as between valence and the ‘angry’ and ‘scared’ emotions (r = −0.335, *p* = 0.0001 and r = −0.224, *p* = 0.0001, respectively). A moderately negative correlation between the ‘happy’ and ‘neutral’ emotions was established (r = −0.447, *p* = 0.0001), and a strong positive correlation between the ‘happy’ emotion and valence was found (r = 0.693, *p* = 0.0001). Alternatively, a strong negative correlation between the ‘sad’ emotion and valence was established (r = −0.693, *p* = 0.0001). A weak negative correlation was also found between valence and the ‘scared’ emotion (r = −0.224, *p* = 0.0001).

A personalized selection of animal species and breeds for individuals, according to their induced ‘happy’ emotion and valence, is shown in Table 4. The obtained results showed that in most participants the maximum intensity of the ‘happy’ emotion induced by the images of different animal species and breeds was related to the maximum valence. However, some of the participants’ maximum ‘happy’ emotion and valence were different for different animal species and breeds. Some participants who expressed their maximum ‘happy’ emotion for different breeds of horses (3.1. and 3.2.) showed the maximum valence for dogs and cats (breeds 1.5. and 2.2., respectively). In addition, the individuals who expressed their maximum ‘happy’ emotion for different breeds of dogs (1.2. and 1.8.) showed the maximum valence for pigs and cats (breeds 4.2. and 2.2., respectively). The participants who expressed their maximum ‘happy’ emotion for cats (breed 2.1.) showed the maximum valence for horses (breed 3.3.). Additionally, the participants who expressed their maximum ‘happy’ emotion for pigs (4% of the participants for breed 4.1. and 2% for breed 4.2.) showed the maximum valence for horses (breed 3.3.), dogs (breed 1.3.), and sheep (breed 5.2.). These results showed that the suggested methodology could lead to a personalized animal selection for AAT for the individuals using both their maximum expression of the ‘happy’ emotion and their maximum valence, as they both were associated with a good mood and a comfortable, positive human emotional state.

## 4. Discussion

Positive emotions, as well as the feelings of happiness, are very important factors for human mental and physical health. Nowadays, the body of scientific studies associated with the compensatory mechanism of positive emotions for persons is growing [59]. It was reported that extroversion and gregariousness are the main factors of a subjective well-being. In addition to being happier, these humans are typically healthier and live longer [60,61]. Happiness is a fundamental emotion, which is described as the presence of positive emotions, life satisfaction, social engagement, and objectives in life [62]. It was also reported that negative emotions come from the specific negative feelings such as fear, anger, and sadness, and positive emotions can be related to the specific positive feelings, i.e., playfulness, nurturance, and exploration-seeking urges [63]. A positive emotional state could be scientifically measured via the self-reported Likert-type rating scales [59]. The ‘happy’ emotion is typically measured by asking the individuals to rate their current level of happiness using either a verbally anchored scale or a pictorial one [64]. Additionally, the ‘happy‘ emotion could be evaluated behaviorally, i.e., by the presence of Duchenne smiling, which has been found to be positively related to a personal subjective self-report on a positive emotion [65]. The Likert scale results could be related with the explicit-controlled emotions, and the FaceReader fixed emotions could be associated with the implicit ones. The three explicit-controlled emotion regulation mechanism—selective attention, distraction, and reappraisal [66]—have been studied the most. Our study showed that between the intensity of the induced emotions and the Likert scale results just two very weak correlations were established (between the Likert scale results and the emotions ‘surprised’ and ‘disgusted’, r = −0.084 and r = 0.078, respectively). However, a very strong positive correlation between the emotion ‘happy’ and valence was found (r = 0.693, *p* = 0.0001). Despite that, it must be pointed out that the intensity of some emotions induced by the different animal species and breed images also showed correlations, which means that the FaceReader technique can attribute the expression of one emotion to another. However, this study showed that different animal species and breeds induced different emotions in different individuals, and the highest intensity of the emotion ‘happy’ was induced when the images and behavioral characteristics of pigs were presented. In the past, pigs were kept near their owners’ homes, fed with leftovers from their guardians’ kitchens, and enjoyed a generally close relationship with humans [67]. Nowadays, the number of minipigs and Vietnamese potbellied pigs kept as pets has increased over the last few years [68]. It could be explained that pigs are intelligent and sensitive animals. In the United States, pig owners often choose to share their pig with their community schools and nursing homes, just to induce positive emotions in the fundraisers [69]. However, under the Americans With Disabilities Act of 1990 (the federal law which governs the use of service animals), pigs cannot be recognized as service animals [70]. It was published that the mini-pig was found to be similarly effective to companion dogs and, in some cases, even better as it was novel to see and touch a mini-pig [71].

Significant differences between the emotions ‘angry’, ‘contempt’, ‘disgusted’, and ‘surprised’ induced by different animal species and breeds were not established. In addition, by summarizing the emotional profile, the highest number of participants showed the highest valence when the dog group was evaluated, followed by the cat and pig groups. It was reported that the individuals’ valence showed a positive correlation with the success rates for interactions with the pet, especially during training and play interactions [28]. However, the characteristic temperament of a pet may be the key factor to the mechanism underlying the positive health effects gained through pet ownership. These characteristics could be associated with animal breed characteristics. However, there are ‘general‘ skill and capacity requirements for any animal who participates in therapeutic settings [66,72]. Our study showed that maximum valence was induced in a higher number of individuals by cat breed 2.2., dog breed 1.1. and 1.2., horse breed 3.3., and pig breed 4.2. The highest number of participants with the maximum emotion ‘happy’ were induced by cat breed 2.1., dog breed 1.2., horse breed 3.3., and pig breed 4.1. There were some differences between the intensity of the ‘happy’ emotion and the valence expressed by testing different animal groups; this could be explained by ‘happy’ being the only positive emotion and ‘surprised’ being either positive or negative. From this point of view, the calculation of valence can differ. However, this study showed that the animal species is a significant factor for the intensity of the ‘neutral’ and ‘happy’ emotions as well as valence. In addition, the animal breed is a significant factor for the intensity of the ‘happy’ emotion and valence. A personalized selection of animal species and breeds for individuals showed that, in the most participants, the maximum intensity of the ‘happy’ emotion induced by the images of different animal species and breeds was related to their maximum valence. However, some of the participants’ maximum ‘happy’ and valence were different for the different animal species and breeds. Finally, these results showed that the suggested methodology could lead to a personalized animal selection for AAT of the individuals by using both their maximum expression of the emotion ‘happy’ and maximum valence, as they both are associated with a good mood and a comfortable, positive human emotional state.

## 5. Conclusions

In this study, the obtained results could be used as a personalized strategy to improve AAT by selecting animal species and breeds for the individuals based on the emotions induced by different animal species and breeds. To our knowledge, this is the first study in this area in which the FaceReader technique has been applied to improve the AAT methodology, which could be the first step to avoiding and/or minimizing stressful situations for persons during contact with an animal. Additionally, it should be pointed out that the current animal selection procedures assess the specific behaviors and skills required for the animals to become a part of the therapy. However, some challenges have been associated with the applied practices because animal characteristics and person reactions (emotions) to the different animal breed and species are critical in achieving the most effective applied AAT results. In addition, this methodology could be used for helping the individuals select their pet. Despite the promising results, it should be pointed out that the cards of animal species and breeds should be standardized, and more participants from different groups (age groups, persons with various disorders, etc.) should be involved in order to improve this methodology.

## Figures and Tables

**Table 1:** Sociodemographic characterization.

Sociodemographic Data		Frequency (Number)	Percentage (%)
Gender	Female	41	82
	Male	9	18
Highest Level of Education	Primary School	0	0
	Secondary School	17	34
	University Degree	33	66
Civil Status	Single	14	28
	Married/Living together	27	54
	Divorced/Separated	7	14
	Widowed	2	4
Occupation	Student	2	4
	Employed	37	74
	Unemployed	0	0
	Retired	5	10
	Working student	6	12
Professional field	Education	36	72
	Health	2	4
	Law and Public policy	1	2
	Community and Social Services	1	2
	Agriculture	10	20
	Other	0	0
Total Number of Participants		50	

**Table 2:** Significance of the analyzed factors and their interaction with different animal species and breeds on the emotions induced in individuals.

Factors	Emotions and Valence	*p*
Animal species	‘Neutral’	**0.0001**
	‘Happy’	**0.0001**
	‘Sad’	0.776
	‘Angry’	0.705
	‘Surprised’	0.552
	‘Scared’	**0.009**
	‘Disgusted’	0.626
	‘Contempt’	0.568
	Valence	**0.006**
Animal breed	‘Neutral’	0.891
	‘Happy’	**0.0001**
	‘Sad’	0.422
	‘Angry’	0.887
	‘Surprised’	0.918
	‘Scared’	0.860
	‘Disgusted’	0.570
	‘Contempt’	0.901
	Valence	**0.001**
Animal species and breed interaction	‘Neutral’	0.986
	‘Happy’	0.621
	‘Sad’	0.984
	‘Angry’	0.958
	‘Surprised’	0.864
	‘Scared’	0.795
	‘Disgusted’	0.788
	‘Contempt’	0.742
	Valence	0.958

*p*: significance; *p* values of ≤0.05 were considered to be significant. Significant results are marked in bold.

**Table 3:** Pearson correlations between the emotion’s intensity induced in persons, and the Likert scale results.

		Likert Scale	‘Neutral’	‘Happy’	‘Sad’	‘Angry’	‘Sur-prised’	‘Scared’	‘Disgus-ted’	‘Con-tempt’	Valence
Likert scale	r	1	−0.033	−0.015	0.008	0.018	−0.084 **	0.013	0.078 **	−0.040	−0.028
	*p*		0.240	0.609	0.776	0.523	**0.003**	0.638	**0.006**	0.154	0.315
‘Neutral’	r	−0.033	1	−0.447 **	−0.353 **	−0.174 **	−0.030	−0.287 **	−0.207 **	−0.115 **	0.118 **
	*p*	0.240		**0.0001**	**0.0001**	**0.0001**	0.291	**0.0001**	**0.0001**	**0.0001**	**0.0001**
‘Happy’	r	−0.015	−0.447 **	1	−0.162 **	−0.087 **	0.069 *	−0.015	0.013	0.075 **	0.693 **
	*p*	0.609	**0.0001**		**0.0001**	**0.002**	**0.014**	0.602	0.642	**0.008**	**0.0001**
‘Sad’	r	0.008	−0.353 **	−0.162 **	1	0.010	−0.130 **	−0.019	0.018	−0.044	−0.693 **
	*p*	0.776	**0.0001**	**0.0001**		0.731	**0.0001**	0.494	0.522	0.122	**0.0001**
‘Angry’	r	0.018	−0.174 **	−0.087 **	0.010	1	−0.085 **	−0.054	0.026	0.013	−0.335 **
	*p*	0.523	**0.0001**	**0.002**	0.731		**0.003**	0.058	0.362	0.651	**0.0001**
‘Surprised’	r	−0.084 **	−0.030	0.069 *	−0.130 **	−0.085 **	1	−0.009	−0.051	−0.013	0.150 **
	*p*	**0.003**	0.291	**0.014**	**0.0001**	**0.003**		0.743	0.072	0.654	**0.0001**
‘Scared’	r	0.013	−0.287 **	−0.015	−0.019	−0.054	−0.009	1	−0.040	0.039	−0.224 **
	*p*	0.638	**0.0001**	0.602	0.494	0.058	0.743		0.162	0.165	**0.0001**
‘Disgusted’	r	0.078 **	−0.207 **	0.013	0.018	0.026	−0.051	−0.040	1	−0.007	−0.108 **
	*p*	**0.006**	**0.0001**	0.642	0.522	0.362	0.072	0.162		0.803	**0.0001**
‘Contempt’	r	−0.040	−0.115 **	0.075 **	−0.044	0.013	−0.013	0.039	−0.007	1	0.085 **
	*p*	0.154	**0.0001**	**0.008**	0.122	0.651	0.654	0.165	0.803		**0.003**
Valence	r	−0.028	0.118 **	0.693 **	−0.693 **	−0.335 **	0.150 **	−0.224 **	−0.108 **	0.085 **	1
	*p*	0.315	**0.0001**	**0.0001**	**0.0001**	**0.0001**	**0.0001**	**0.0001**	**0.0001**	**0.003**	

r: Pearson correlation coefficient. The Pearson correlation coefficients that are statistically significant are marked in bold. *p*: significance (2-tailed). ** Correlation is significant at the 0.01 level (2-tailed). * Correlation is significant at the 0.05 level (2-tailed).

**Table 4:** Personalized selection of animal species and breeds for individuals, according to the animal species and breed-induced ‘happy’ and valence values.

Participant ID	Max of ‘Happy’ Mean	Animal ID No.	Animal	Max Valence Mean	Animal ID No.	Animal
1	0.7366	2.2	Cats	0.7286	2.2	Cats
2	0.3474	1.1	Dogs	0.3394	1.1	Dogs
3	0.2078	4.1	Pigs	0.1698	4.1	Pigs
4	0.0862	1.1	Dogs	0.0681	1.1	Dogs
5	0.2848	1.6	Dogs	0.2381	1.6	Dogs
**6**	**0.0152**	**3.1**	**Horses**	**−0.0040**	**1.5**	**Dogs**
7	0.2181	2.2	Cats	0.1670	2.2	Cats
8	0.3419	4.2	Pigs	0.3405	4.2	Pigs
9	0.4051	1.1	Dogs	0.3975	1.1	Dogs
10	0.3482	1.1	Dogs	0.3369	1.1	Dogs
11	0.2997	2.1	Cats	0.2961	2.1	Cats
12	0.0081	2.8	Cats	−0.0107	2.3	Cats
13	0.0412	2.7	Cats	−0.0012	2.7	Cats
**14**	**0.0080**	**3.2**	**Horses**	**−0.0103**	**2.2**	**Cats**
**15**	**0.0594**	**1.2**	**Dogs**	**−0.0025**	**4.2**	**Pigs**
16	0.1361	3.3	Horses	0.0893	3.3	Horses
17	0.2393	4.2	Pigs	0.2270	4.2	Pigs
18	0.4028	1.1	Dogs	0.4010	1.1	Dogs
19	0.1469	4.1	Pigs	0.1102	4.1	Pigs
20	0.4085	1.2	Dogs	0.3397	1.2	Dogs
21	0.2799	2.3	Cats	0.2502	2.3	Cats
22	0.7498	1.13	Dogs	0.7194	1.13	Dogs
23	0.3399	1.6	Dogs	0.3332	1.6	Dogs
24	0.4035	1.2	Dogs	0.3860	1.2	Dogs
25	0.4034	1.2	Dogs	0.28345	1.2	Dogs
**26**	**0.1676**	**1.8**	**Dogs**	**0.0977**	**2.2**	**Cats**
27	0.3088	4.1	Pigs	0.2994	4.1	Pigs
28	0.4284	1.2	Dogs	0.3971	1.2	Dogs
29	0.0796	1.6	Dogs	0.0222	1.6	Dogs
30	0.1761	2.1	Cats	0.1641	2.1	Cats
**31**	**0.0590**	**2.1**	**Cats**	**−0.0116**	**3.3**	**Horses**
32	0.1314	2.1	Cats	0.0748	2.1	Cats
**33**	**0.0553**	**4.1**	**Pigs**	**0.0130**	**3.3**	**Horses**
**34**	**0.0938**	**4.1**	**Pigs**	**0.0110**	**1.3**	**Dogs**
35	0.2546	1.11	Dogs	0.1807	1.11	Dogs
36	0.0527	3.3	Horses	0.0445	3.3	Horses
37	0.2049	1.13	Dogs	0.1345	1.3	Dogs
38	0.1494	2.7	Cats	0.0686	2.7	Cats
39	0.1802	4.1	Pigs	0.1268	4.2	Pigs
40	0.4666	1.3	Dogs	0.4638	1.3	Dogs
41	0.3785	4.1	Pigs	0.3378	4.1	Pigs
42	0.6317	4.2	Pigs	0.6042	4.2	Pigs
43	0.4275	4.2	Pigs	0.4249	4.2	Pigs
44	0.2304	4.1	Pigs	0.2236	4.1	Pigs
45	0.6481	1.2	Dogs	0.6411	1.2	Dogs
46	0.5313	1.3	Dogs	0.5267	1.3	Dogs
47	0.2396	2.2	Cats	0.2386	2.2	Cats
48	0.5970	1.4	Dogs	0.5697	1.4	Dogs
49	0.7119	1.5	Dogs	0.6299	1.13	Dogs
**50**	**0.6317**	**4.2**	**Pigs**	**0.3889**	**5.2**	**Sheep**

The results, which differed in the selection of the animal according to max emotion ‘happy’ and max valence, are marked in bold. Dogs: 1.1. Australian shepherd, 1.2. pug, 1.3. Labrador retriever, 1.4. Doberman, 1.5. miniature schnauzer, 1.6. beagle, 1.7. mixed breed, 1.8. Yorkshire terrier, 1.9. mixed breed, 1.10. mixed breed, 1.11. Cane Corso, 1.12. Samoyed, and 1.13. Chihuahua; cats: 2.1. mixed breed, 2.2. British shorthair, 2.3. Himalayan cat, 2.4. mixed breed, 2.5. mixed breed, 2.6. Siamese cat, 2.7. Sphynx, and 2.8. Bengal cat; horses: 3.1. Norwegian Fjord, 3.2. Exmoor pony, 3.3. Andalusian, and 3.4. Friesian; pigs: 4.1. Vietnamese pot-bellied and 4.2. Kunekune; sheep: 5.1. Herdwick sheep and 5.2. Suffolk sheep.

## Data Availability

Data available on request due to restrictions e.g., privacy or ethical. The data are not publicly available due to privacy restrictions.

## Supplementary Materials

Following supporting information can be downloaded at: https://www.mdpi.com/article/10.3390/ani12050597/s1, File S1: cards of different animal species and breeds; File S2: emotions induced in persons by different animal species and breeds; File S3: multivariate ANOVA results.
