# Supplementary material for: Personalized Strategy for Animal-Assisted Therapy for Individuals Based on the Emotions Induced by the Images of Different Animal Species and Breeds
Source: Animals (Basel). 2022 Feb 27;12(5):597. doi: 10.3390/ani12050597 (PMC8909388; doi:10.3390/ani12050597)
Supplement: Supplementary file 1 [file animals-12-00597-s001.zip › Supplementary file 1. Cards of the different animal species and breeds.pdf]

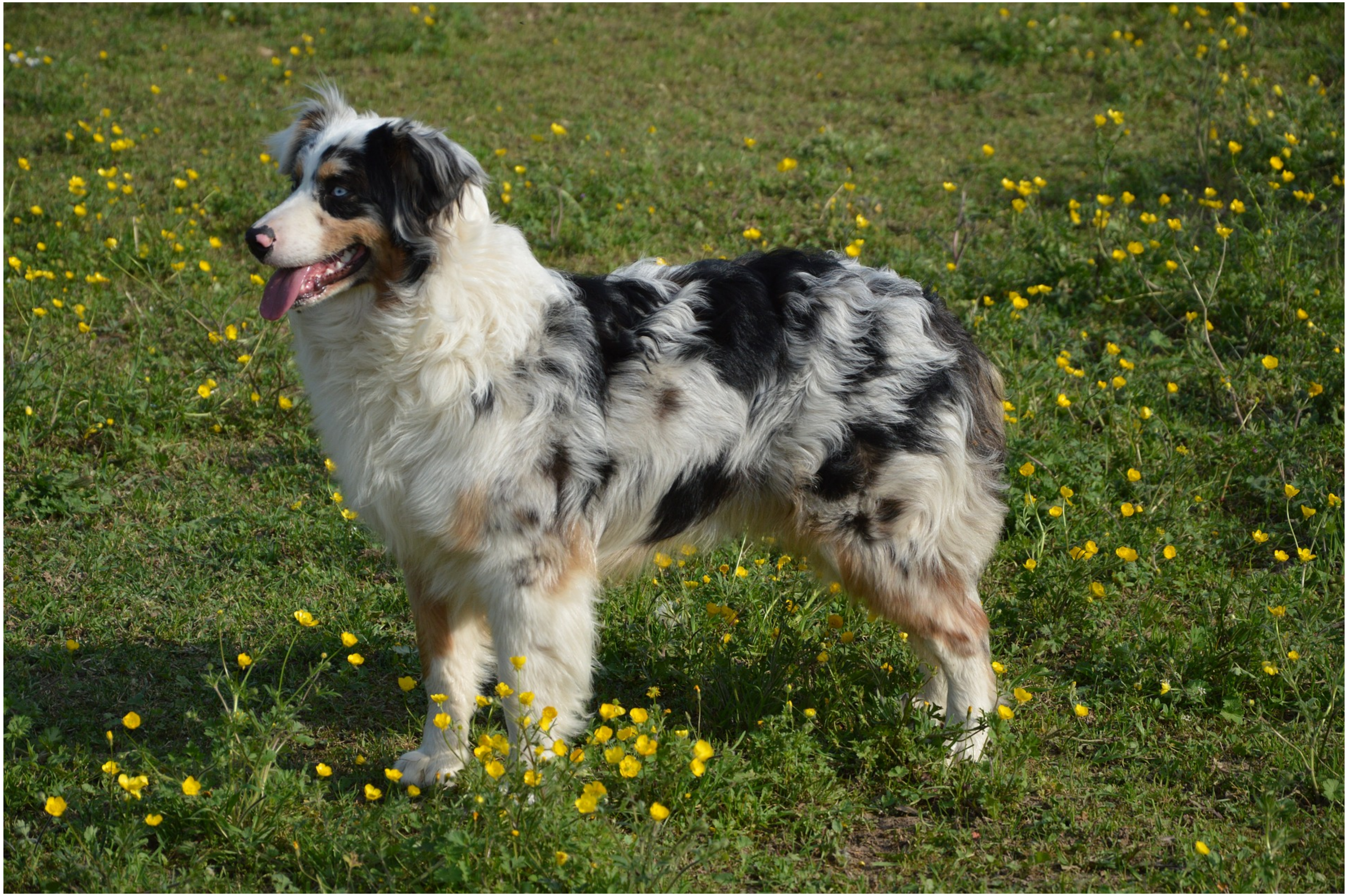

https://pixabay.com/photos/dog-young-dog-australian-sheperd-326650/

1.1. Australian Shepherd

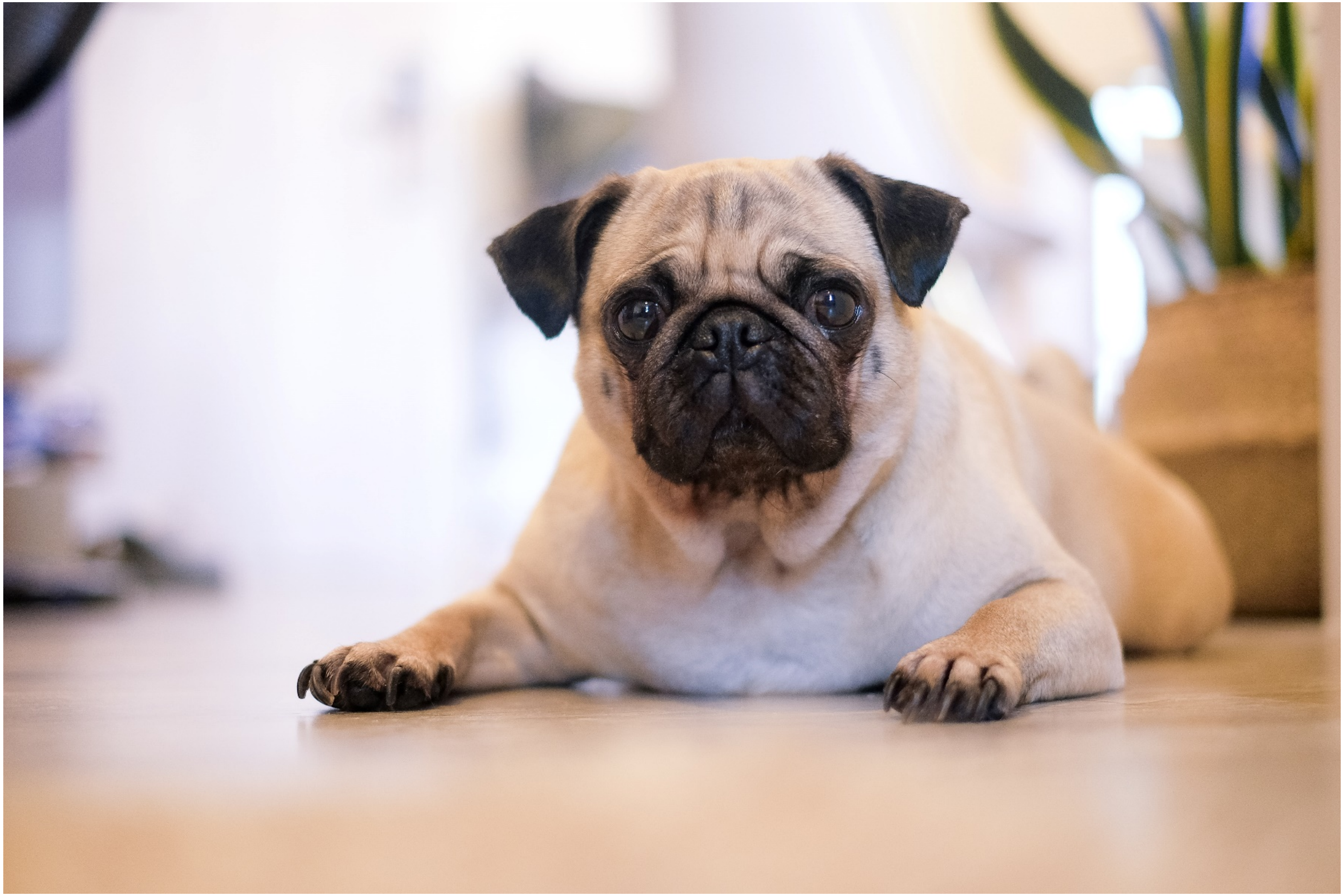

https://unsplash.com/photos/UAsFSsMDpa0

1.2. Pug

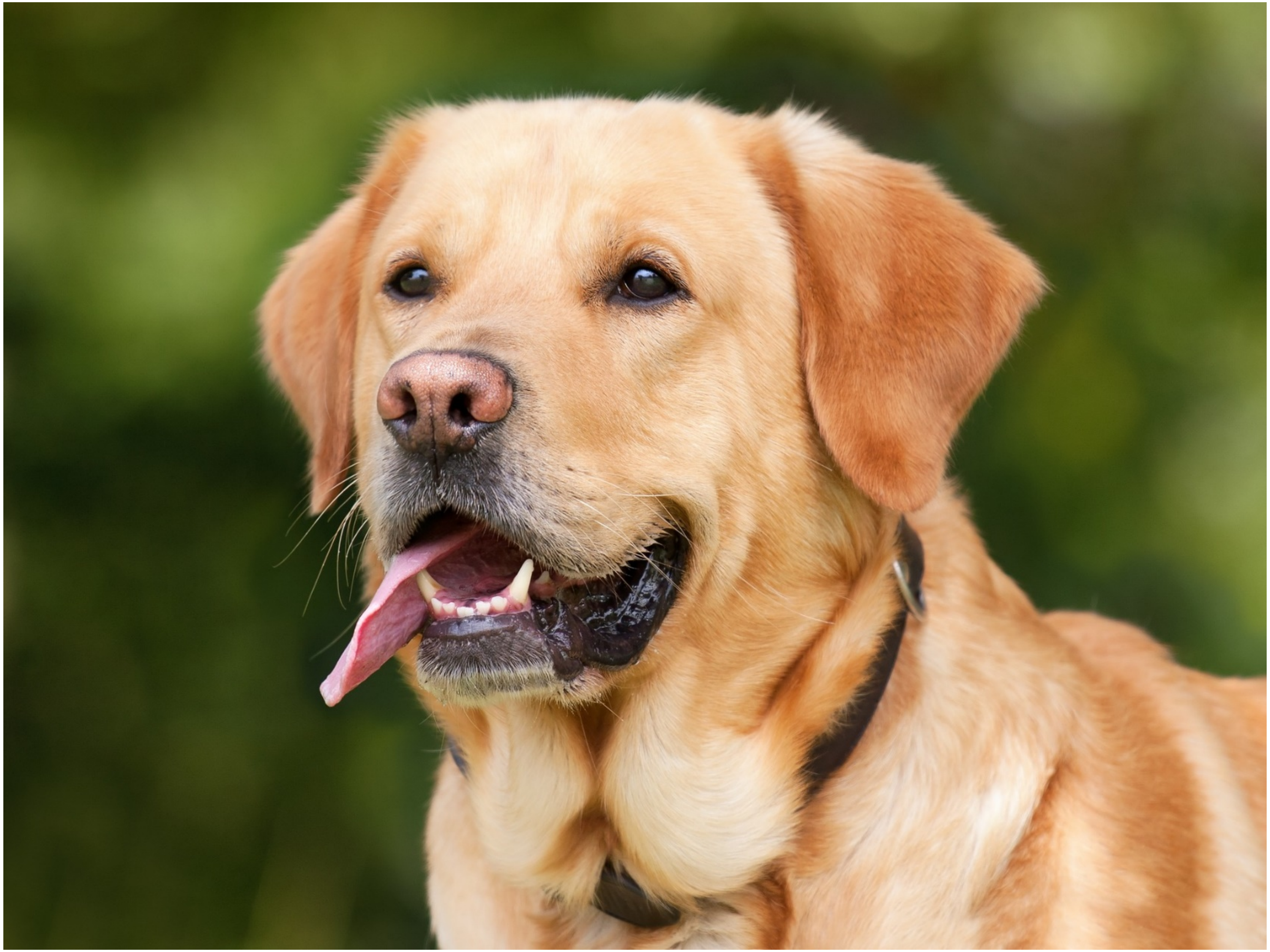

https://pixabay.com/photos/labrador-retriever-dog-pet-1210559/

1.3. Labrador Retriever

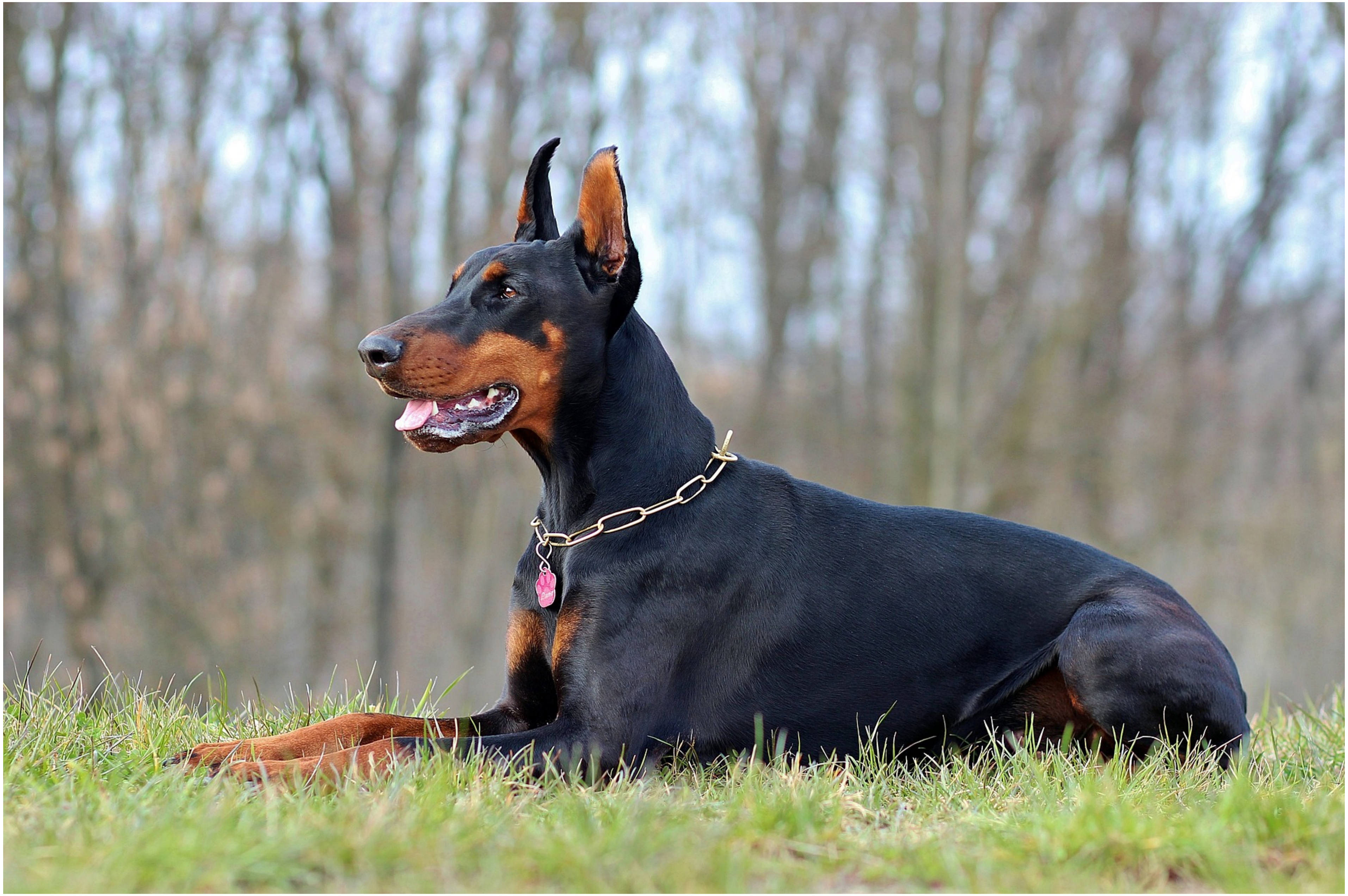

https://pixabay.com/photos/lying-doberman-dog-animal-species-1283356/

1.4. Dobermann

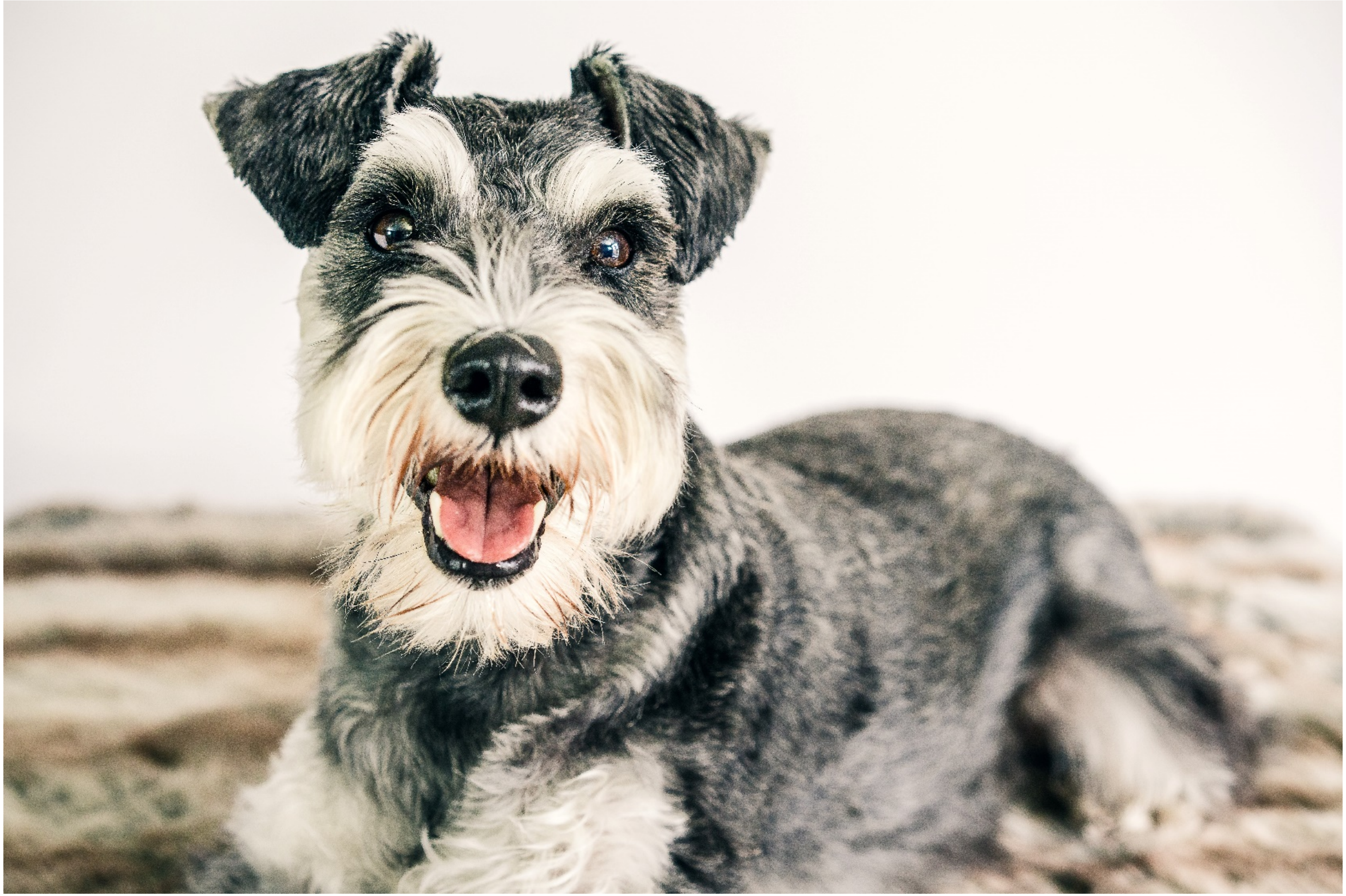

https://unsplash.com/photos/aFDgHo2u10M

1.5. Miniature Schnauzer

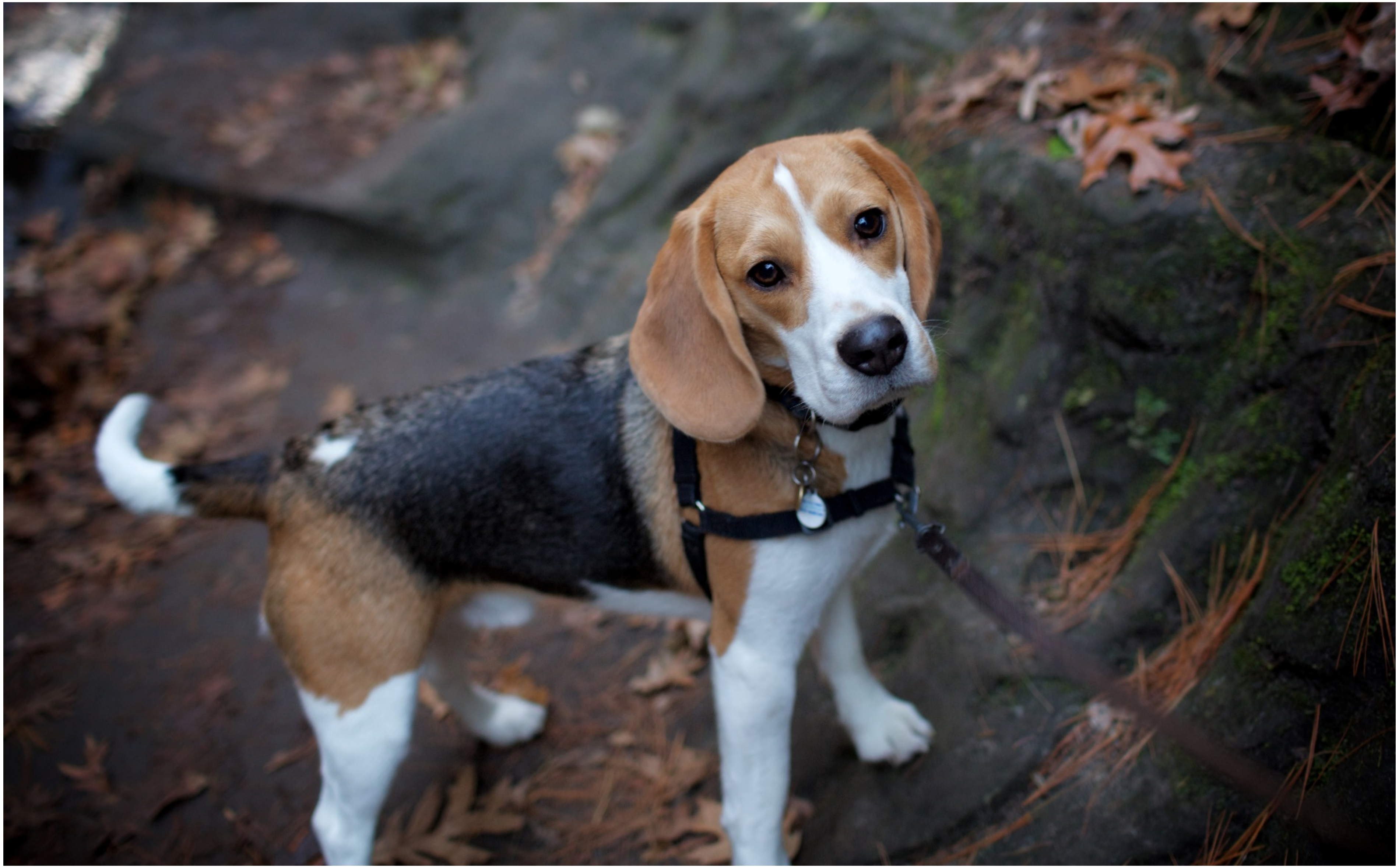

https://unsplash.com/photos/Bf2yJ99JMT0

1.6. Beagle

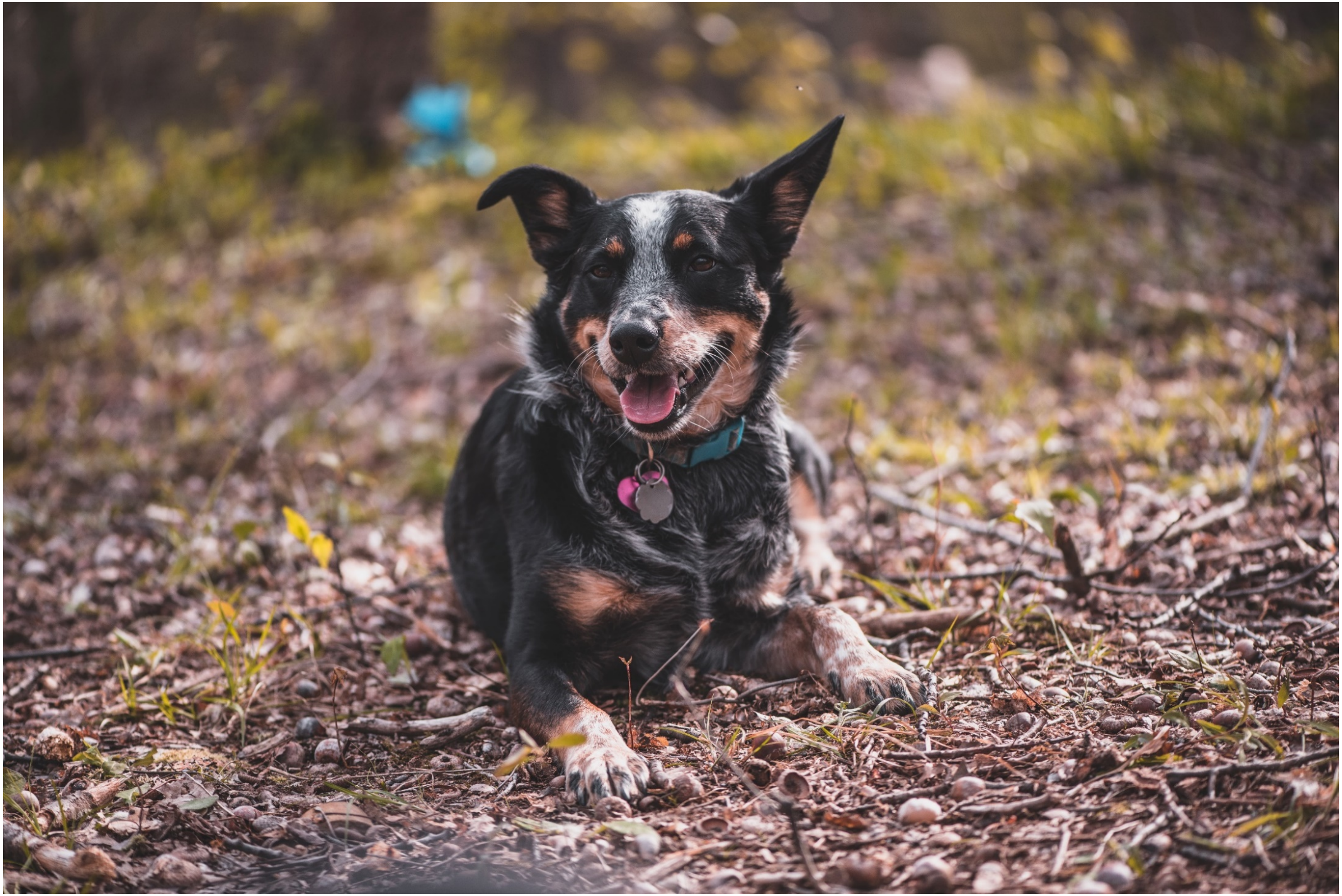

https://unsplash.com/photos/oQ0HZZ1md08

1.7. Mixed breed

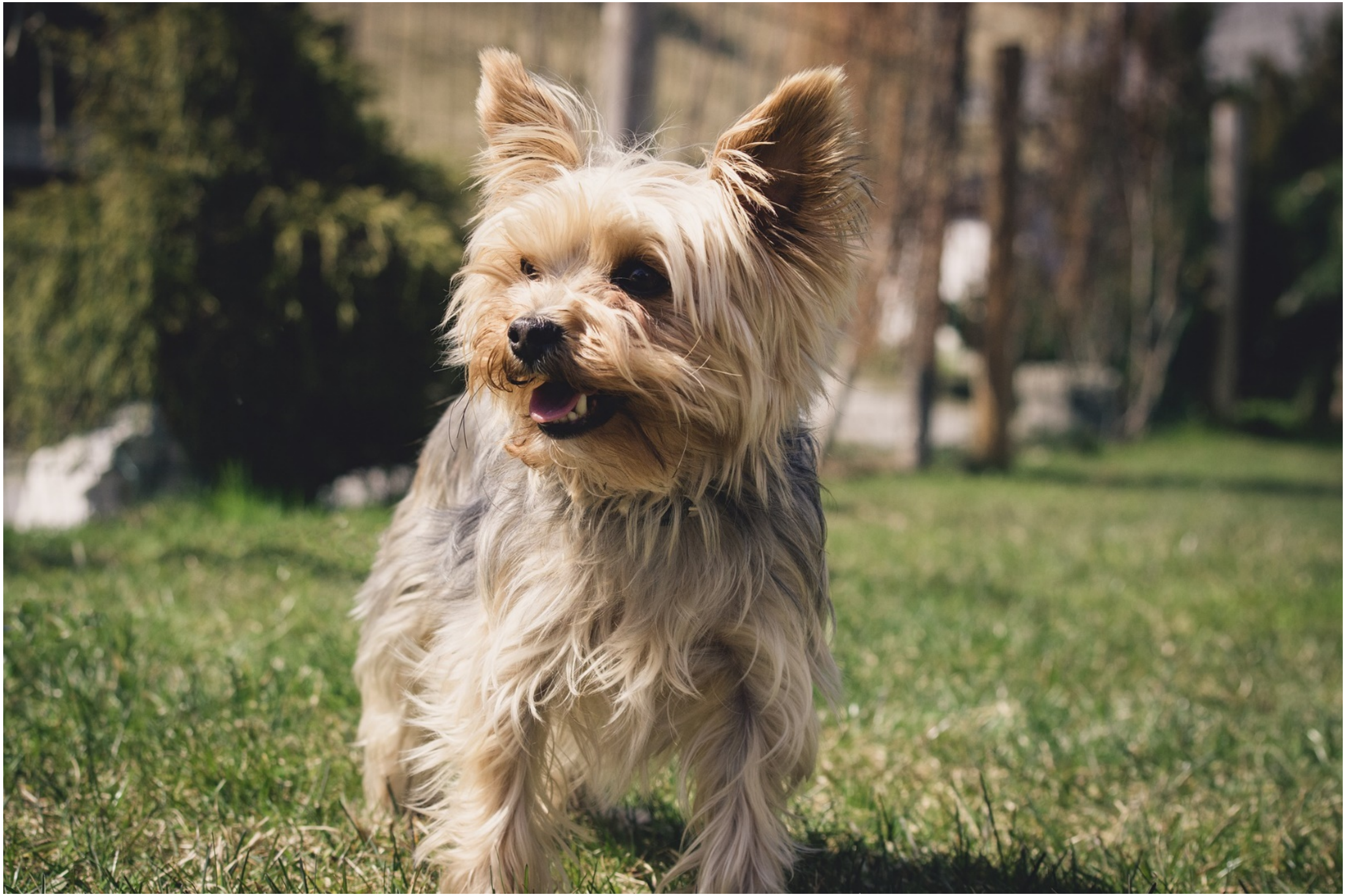

https://pixabay.com/photos/dog-small-yorkie-yorkshire-terrier-5037931/

1.8. Yorkshire Terrier

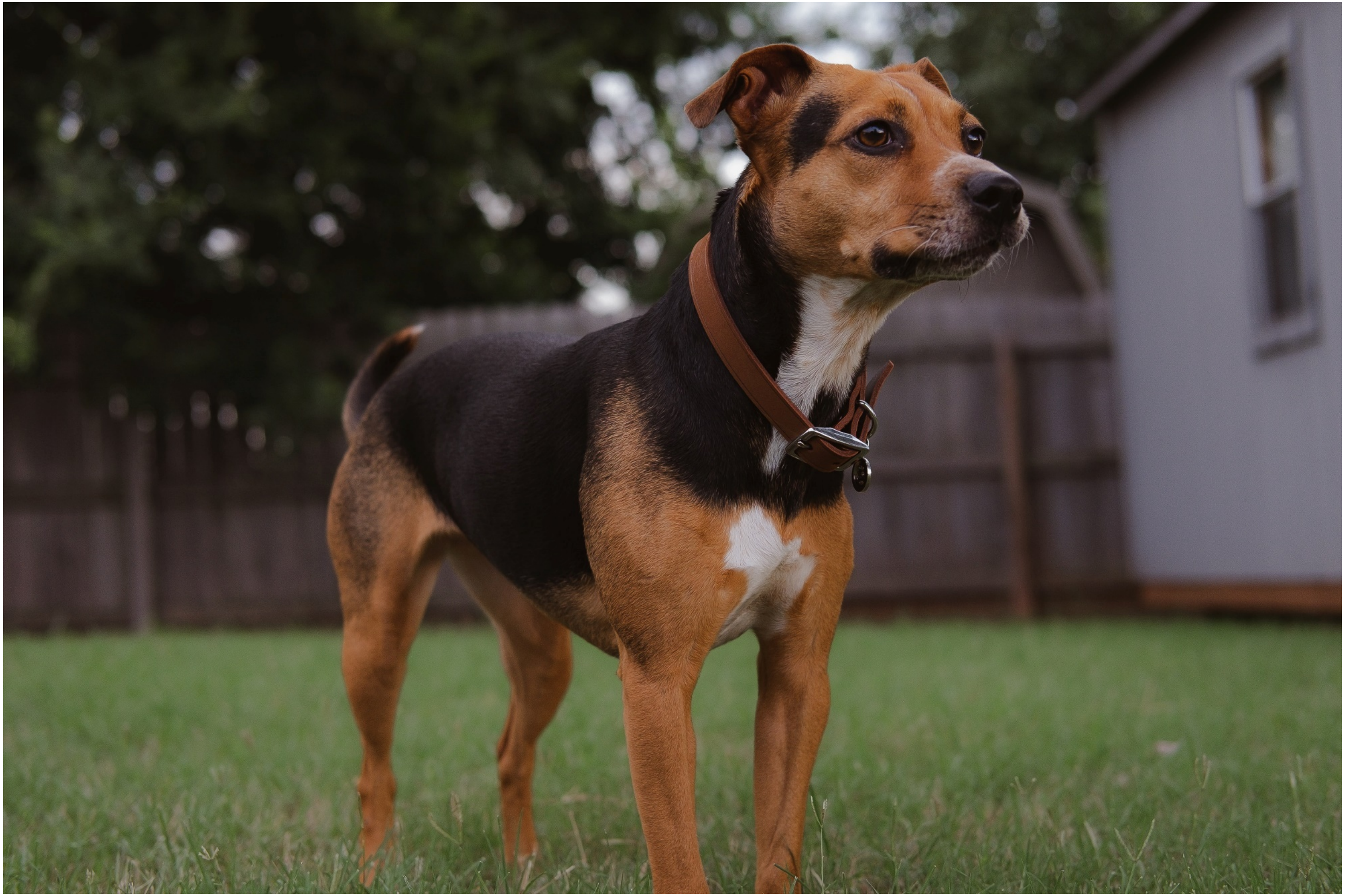

https://unsplash.com/photos/ofgRowfw_TQ

1.9. Mixed breed

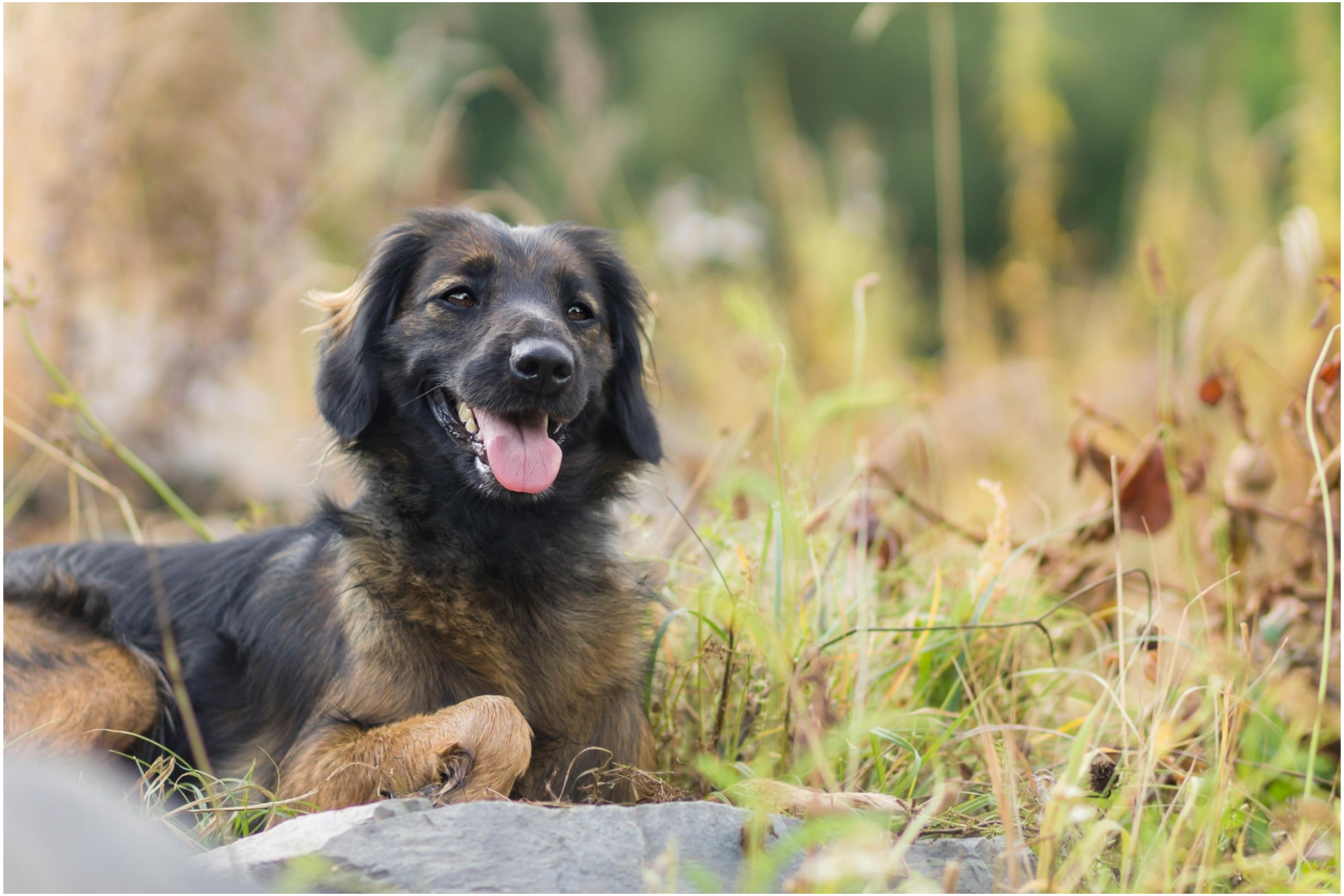

https://unsplash.com/photos/MSr_RB232HI

1.10. Mixed breed

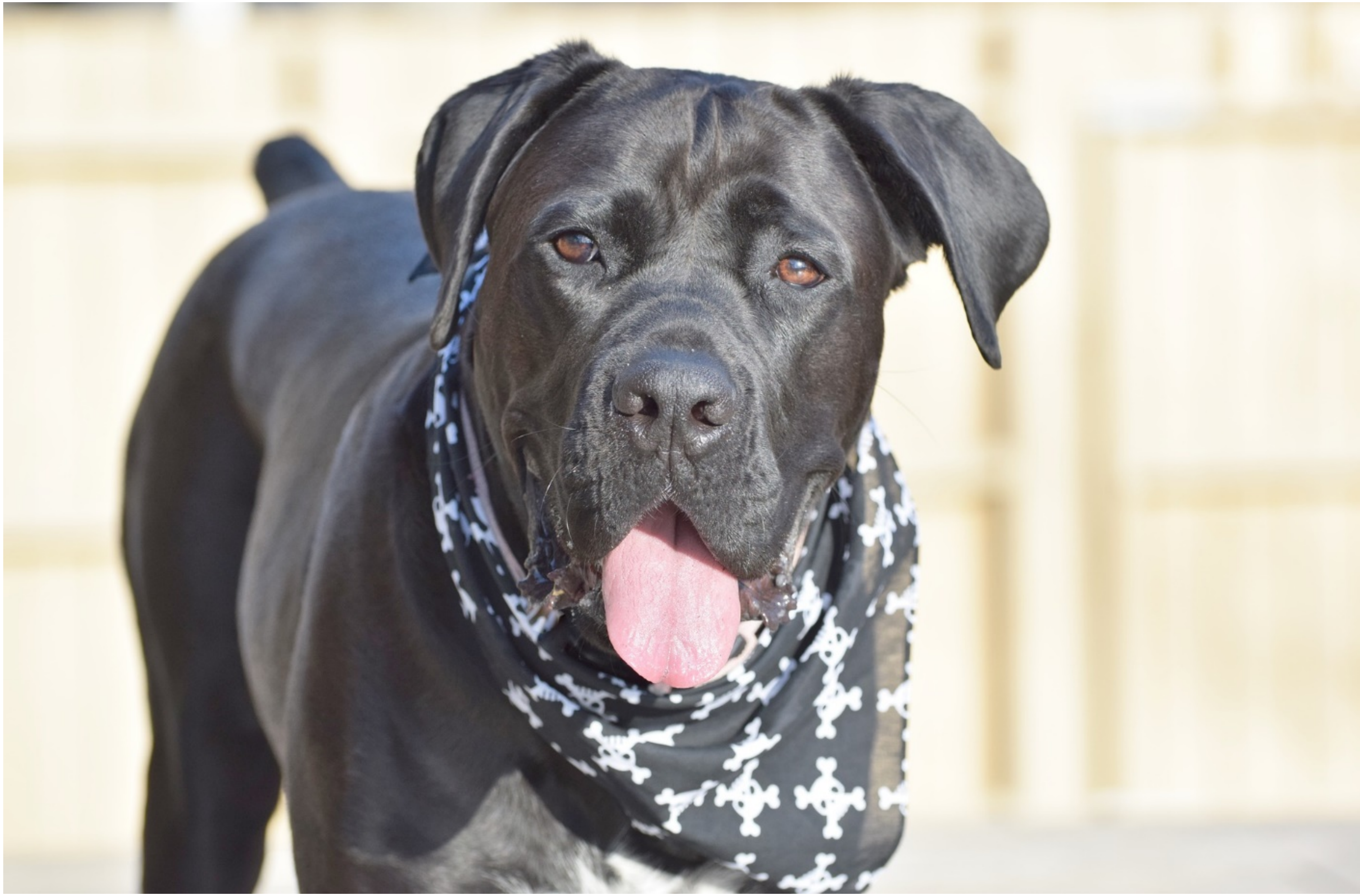

https://pixabay.com/photos/dog-puppy-pet-animal-cute-mastiff-4044828/

1.11. Cane Corso

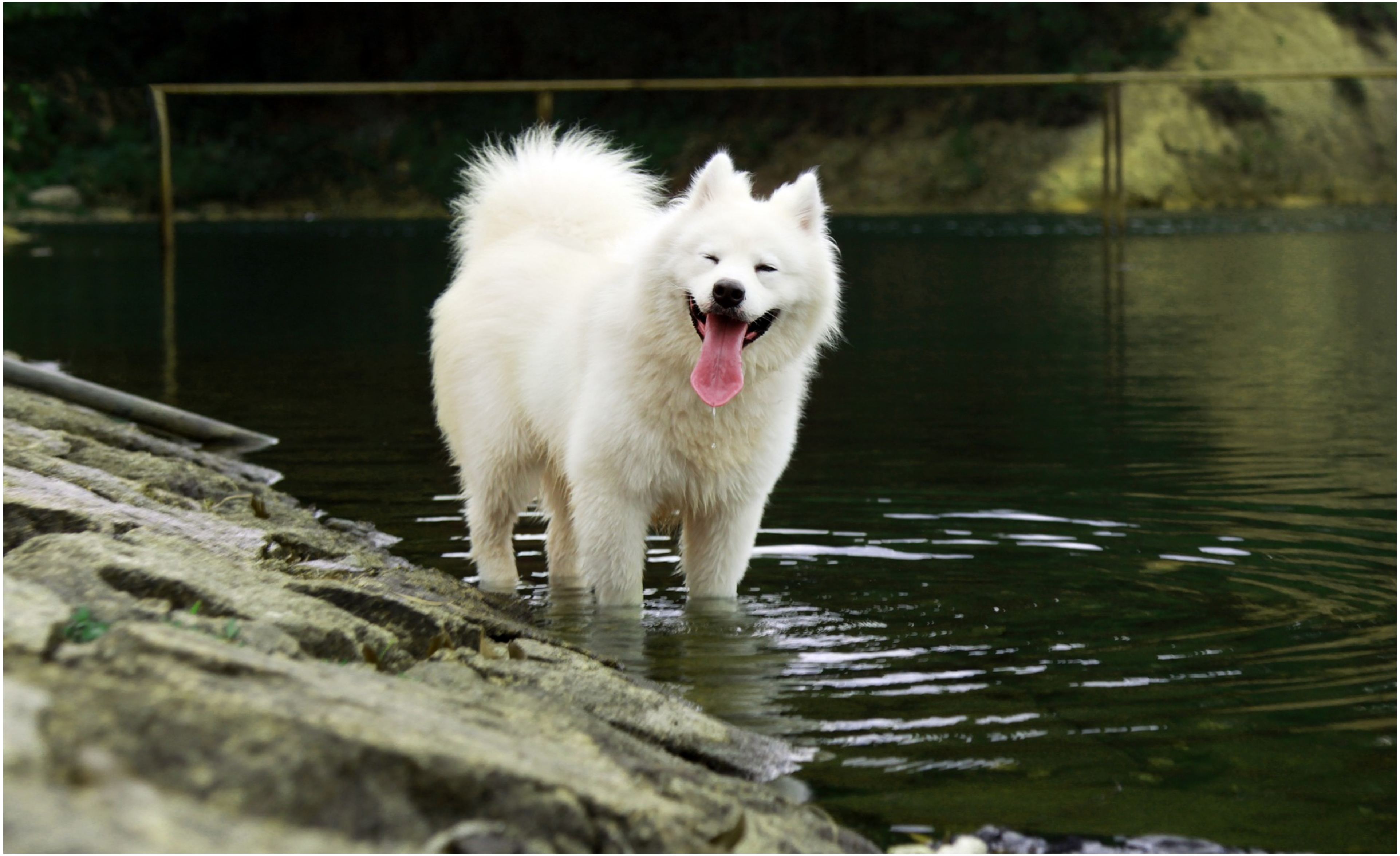

https://pixabay.com/photos/samoyed-pets-sled-dogs-2765557/

1.12. Samoyed

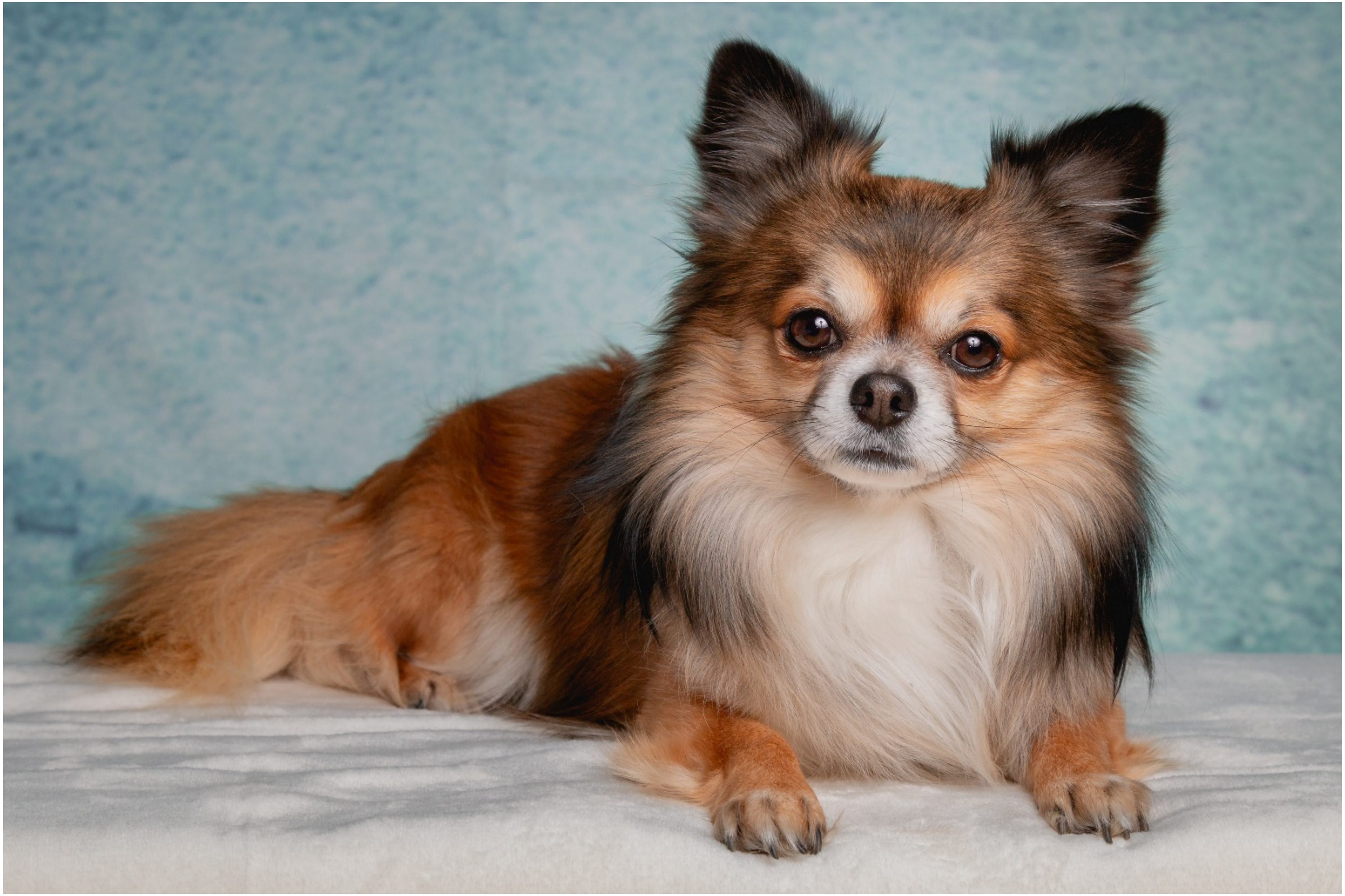

https://unsplash.com/photos/AXwQKtPS-s4

1.13. Chihuahua

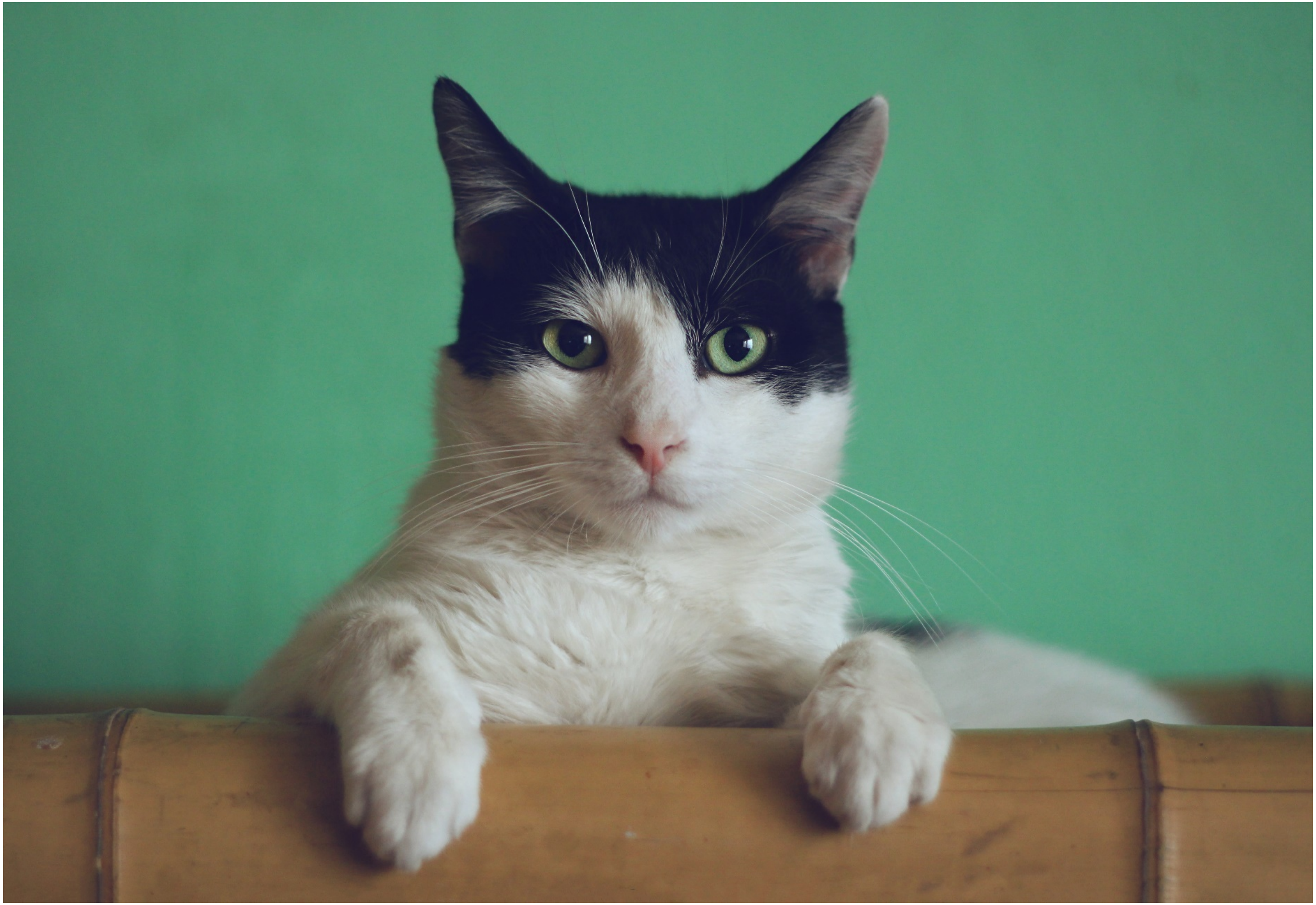

https://unsplash.com/photos/gKXKBY-C-Dk

2.1. Mixed breed

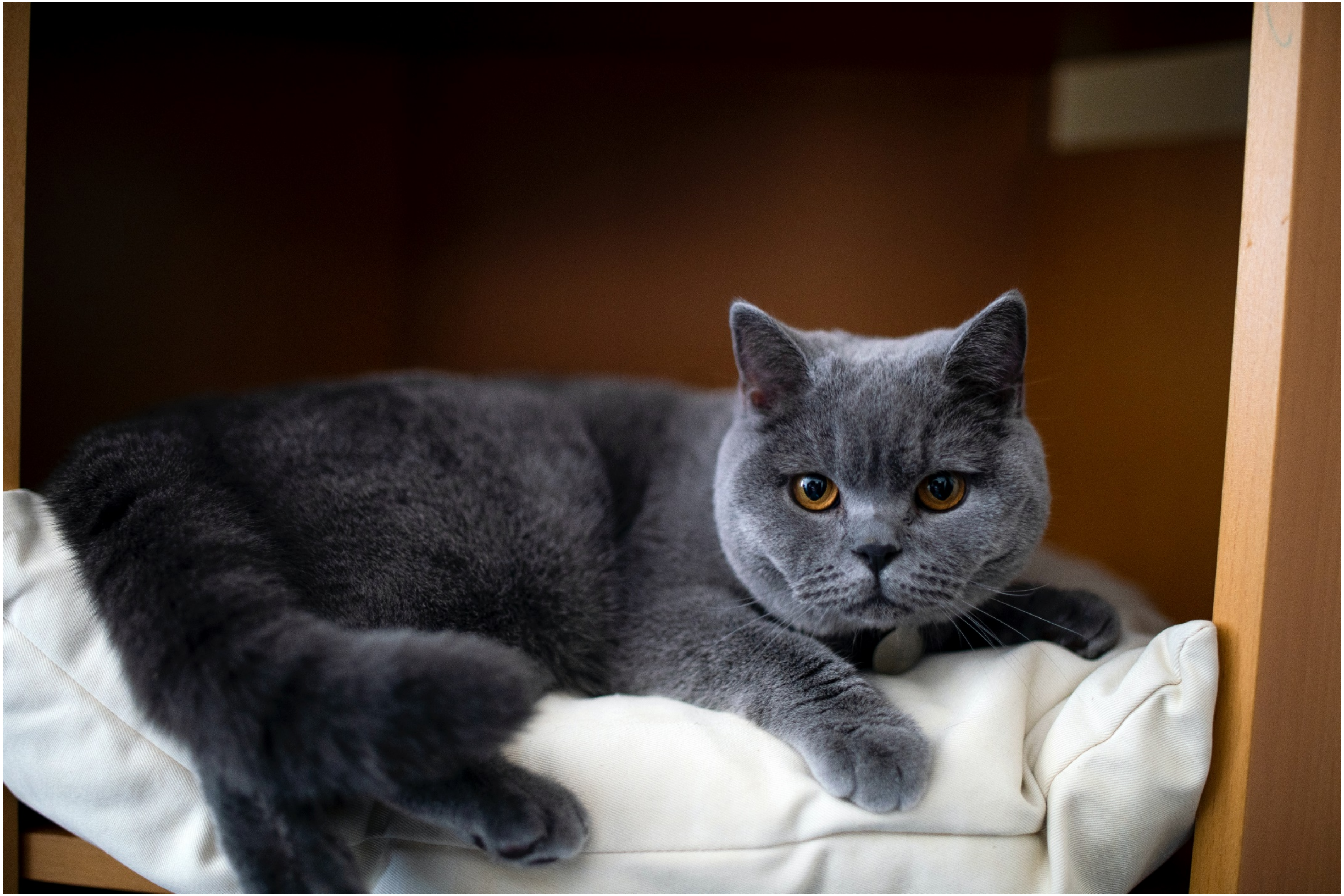

https://unsplash.com/photos/PXTsSbh2uy4

2.2. British Shorthair

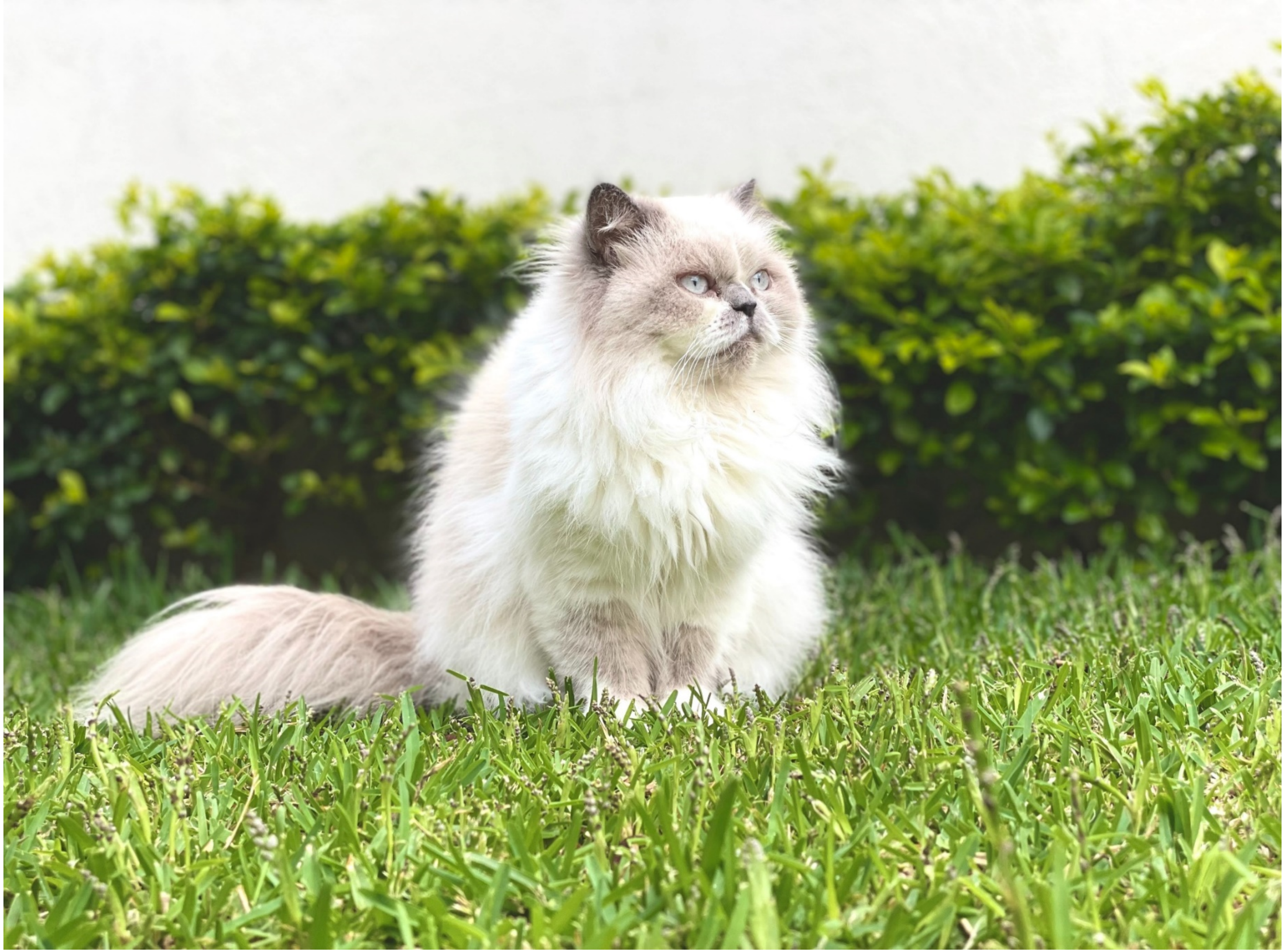

https://unsplash.com/photos/ucWiiistGKE

2.3. Himalayan cat

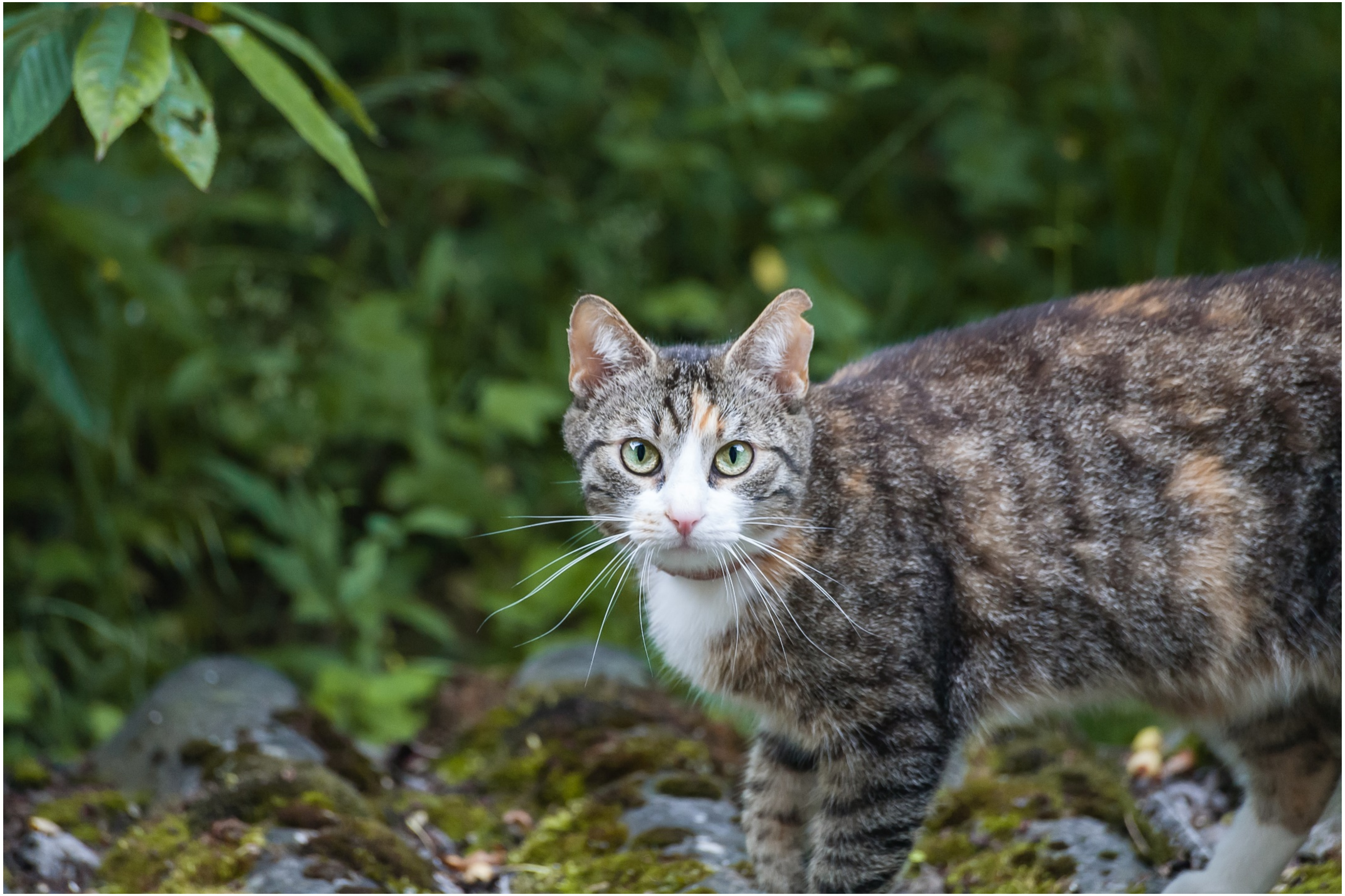

https://unsplash.com/photos/gkuiAxHbkb0

2.4. Mixed breed

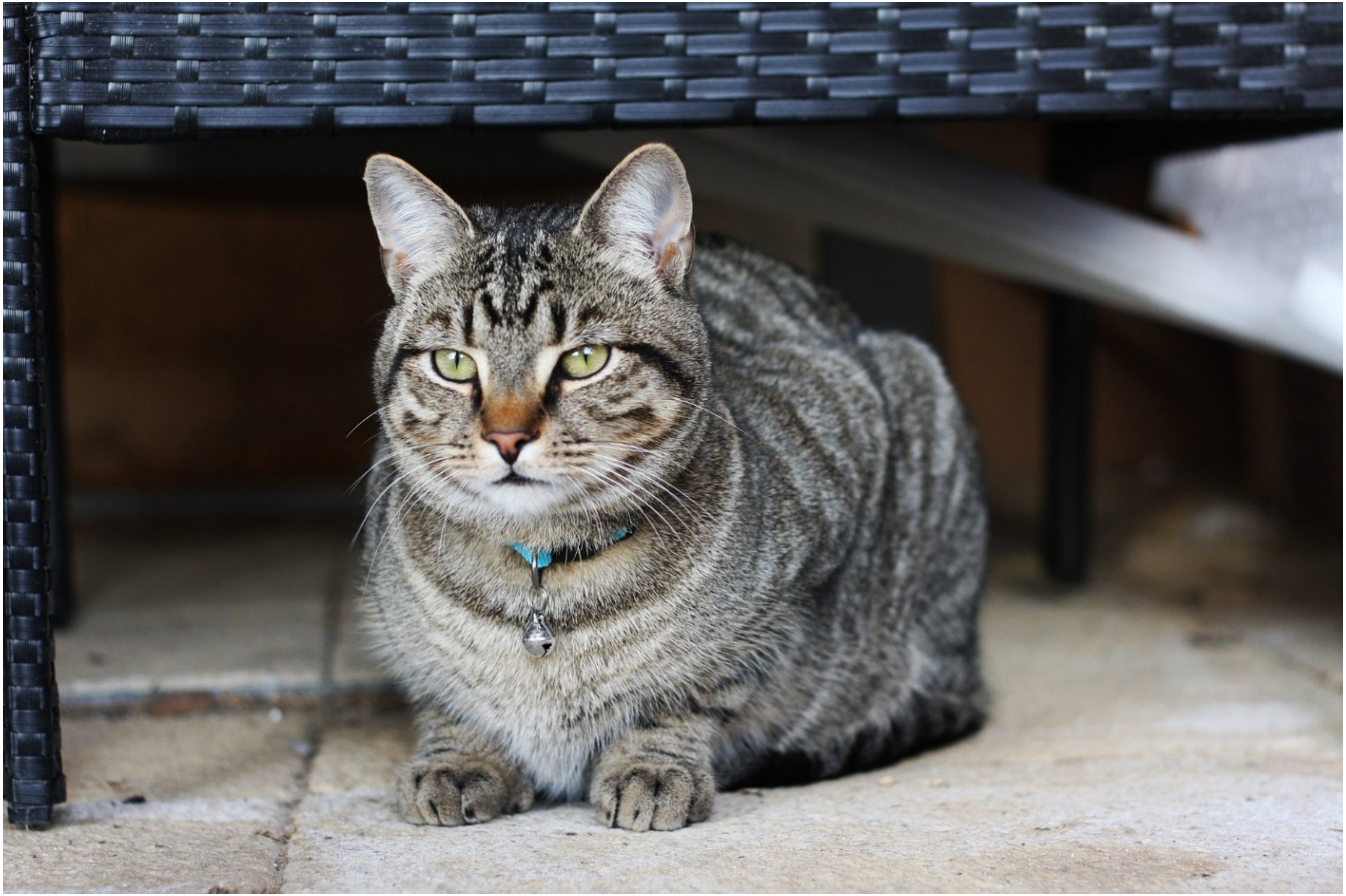

https://pixabay.com/photos/cat-tabby-mutt-pet-animal-kitten-3806799/

2.5. Mixed breed

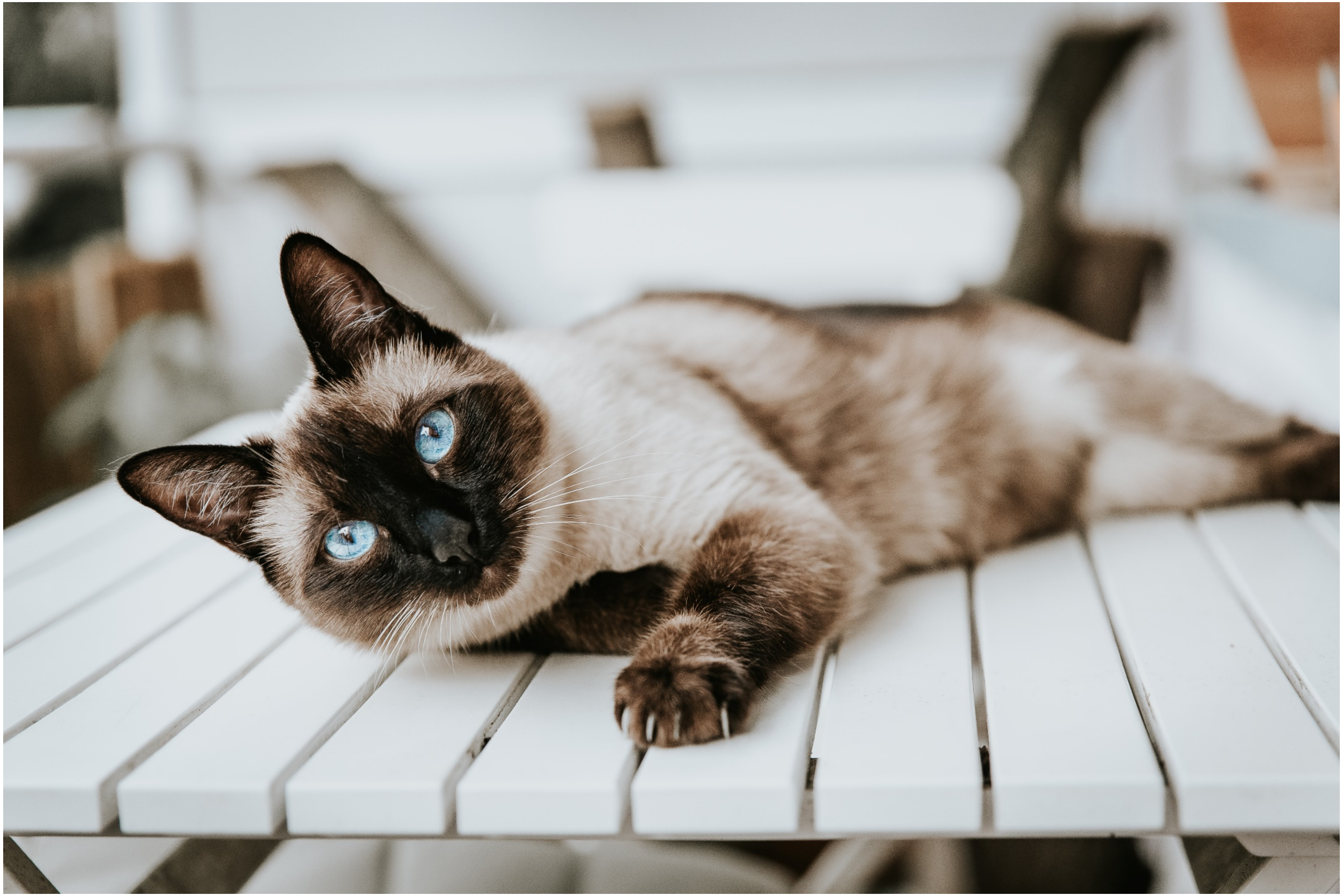

https://unsplash.com/photos/ZY97RvOiAOA

2.6. Siamese cat

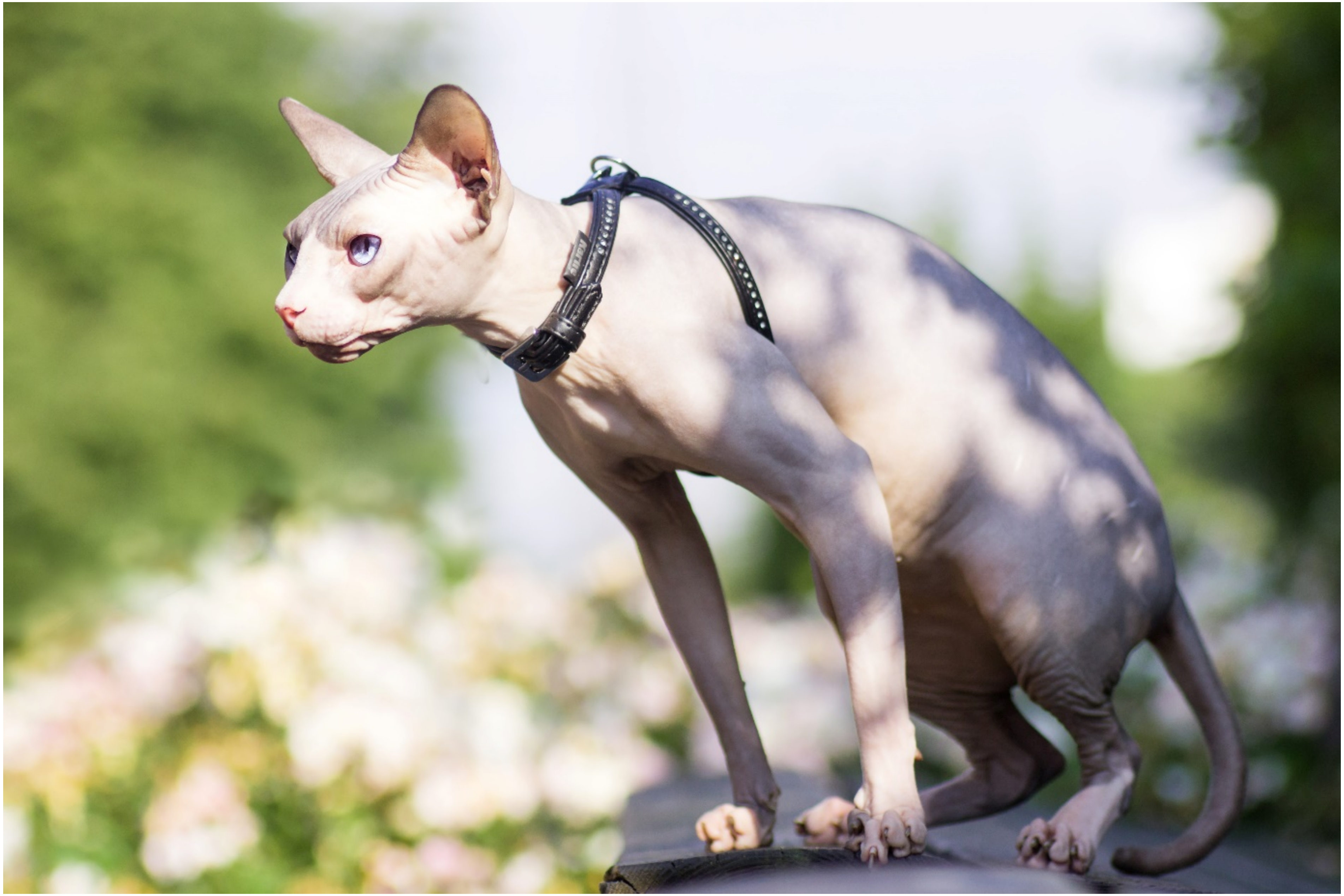

https://unsplash.com/photos/BqIWI6FS240

2.7. Sphynx

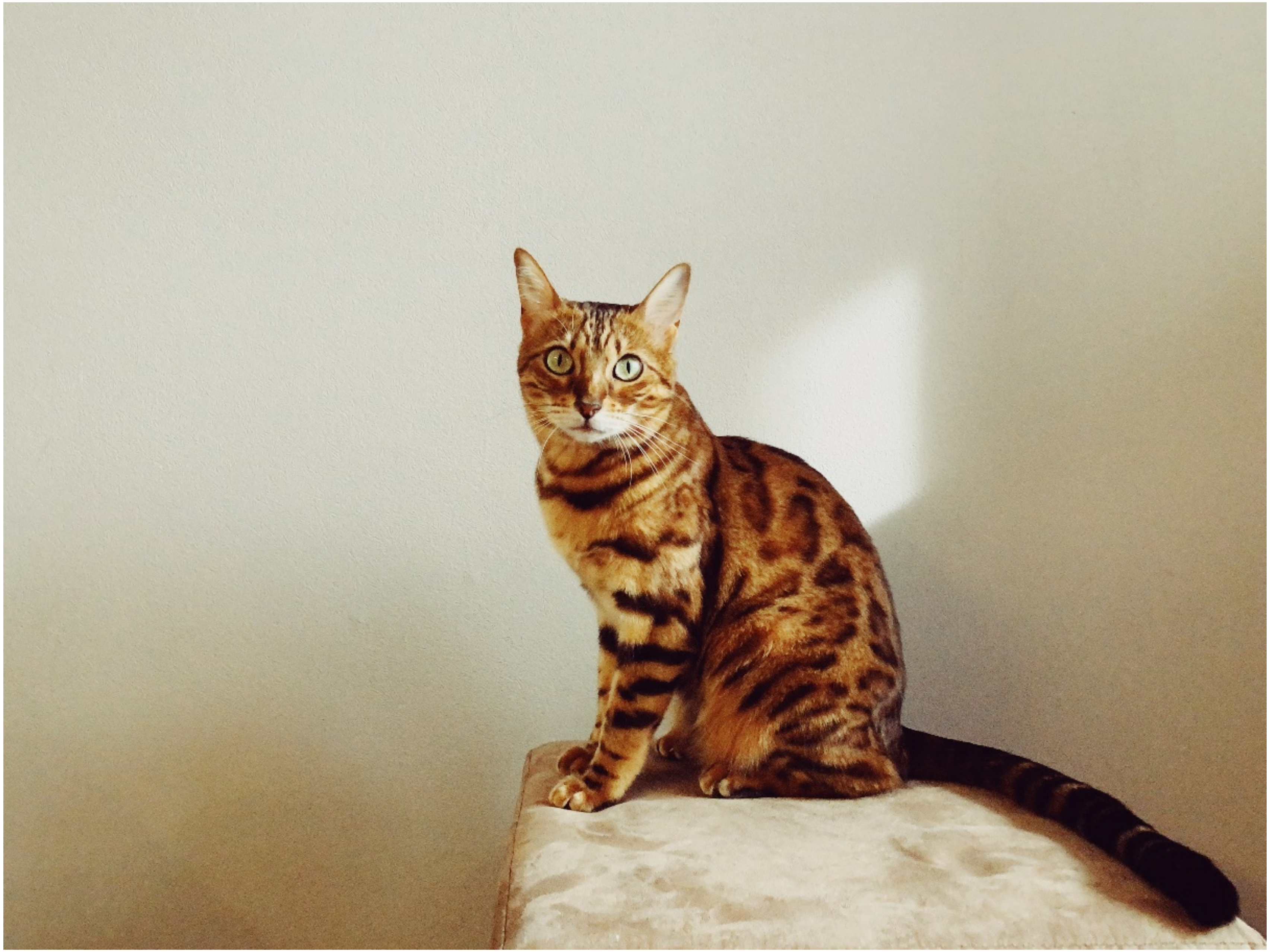

https://unsplash.com/photos/cjJYr-m2H9U

2.8. Bengal cat

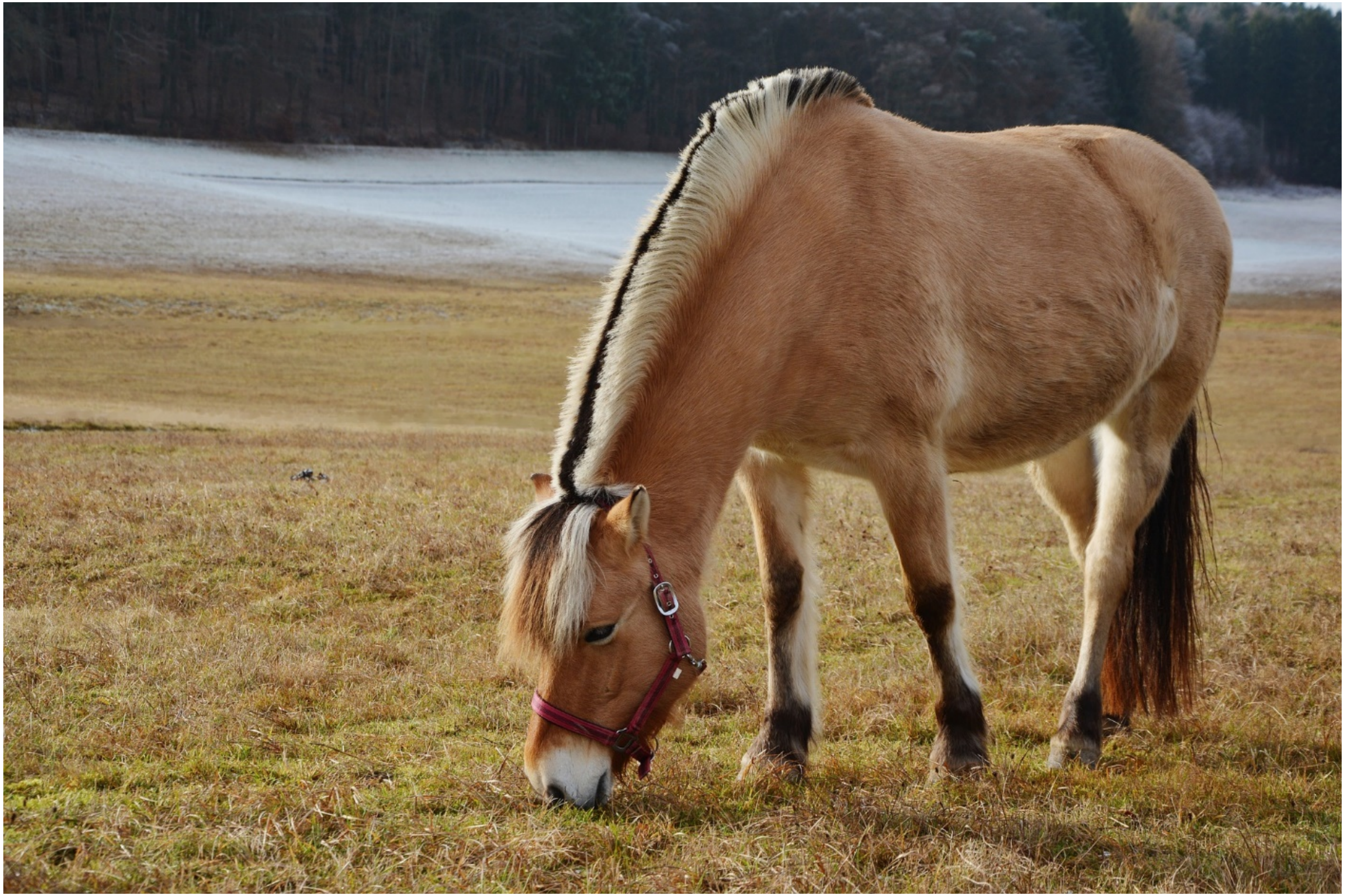

https://pixabay.com/photos/horse-norwegian-fjord-horse-pasture-1946869/

3.1. Norwegian Fjord

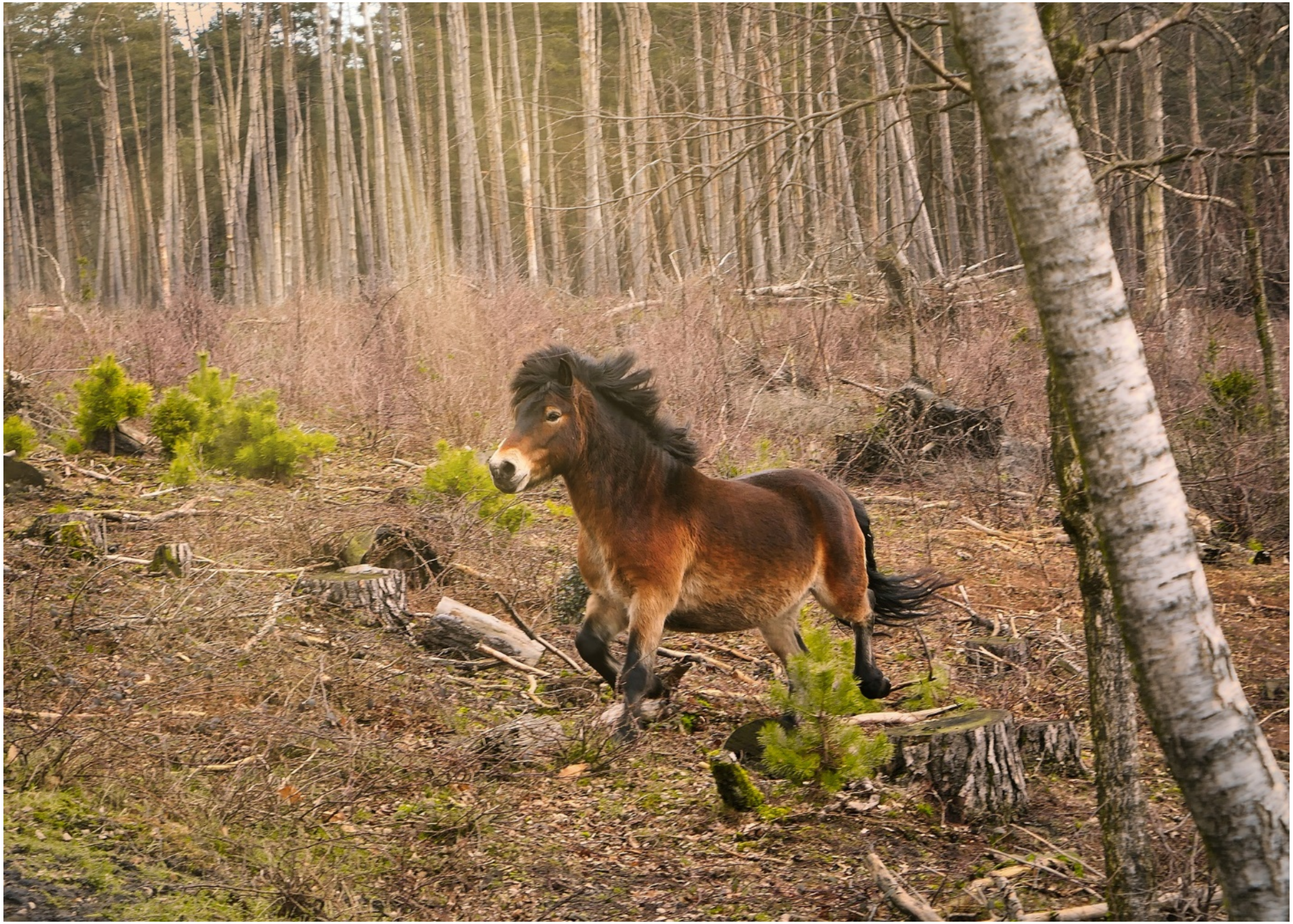

https://pixabay.com/photos/exmoor-pony-horse-forest-senne-6092191/

3.2. Exmoor pony

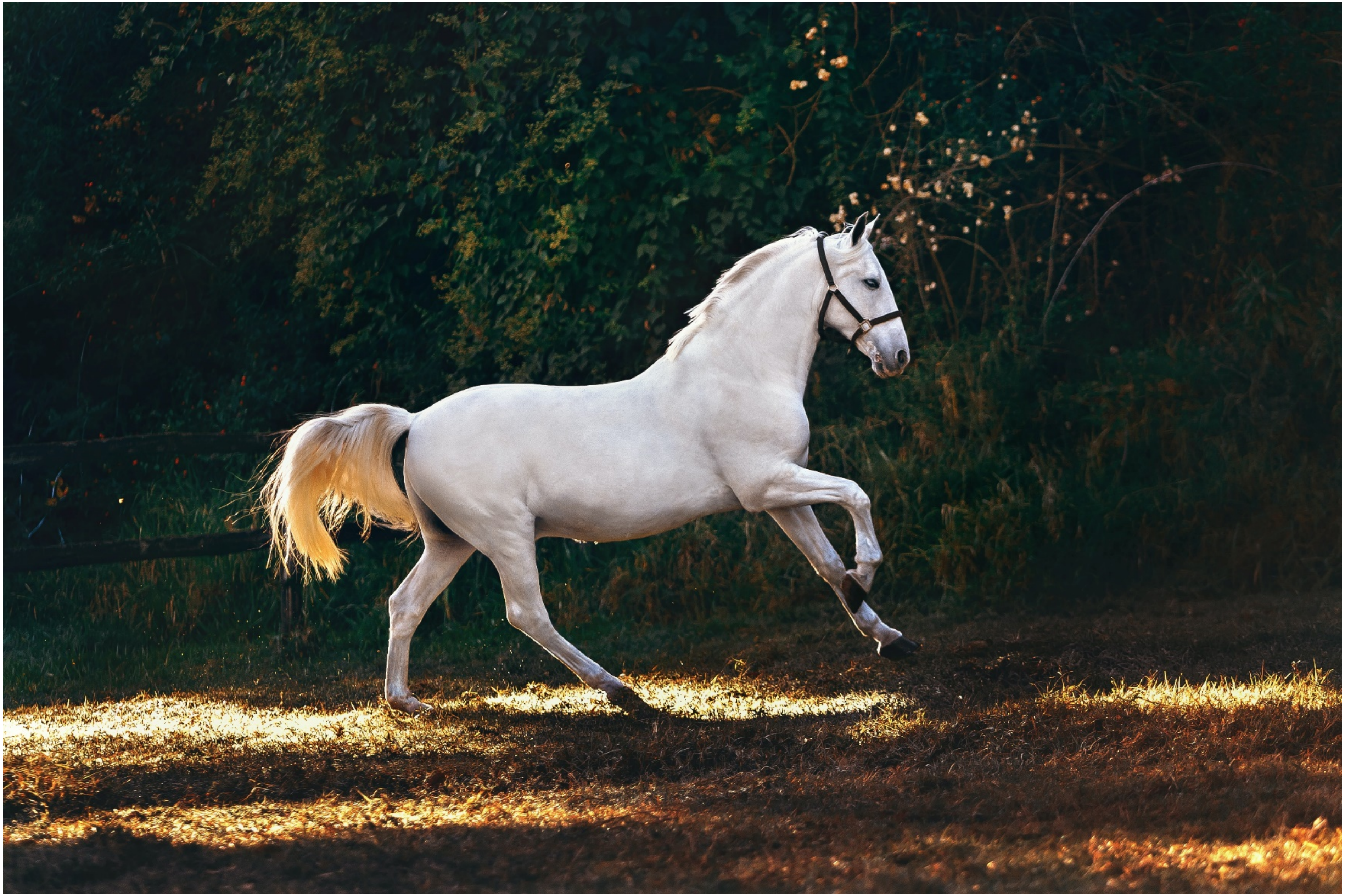

https://unsplash.com/photos/7FC4WpyYcfQ

3.3. Andalusian

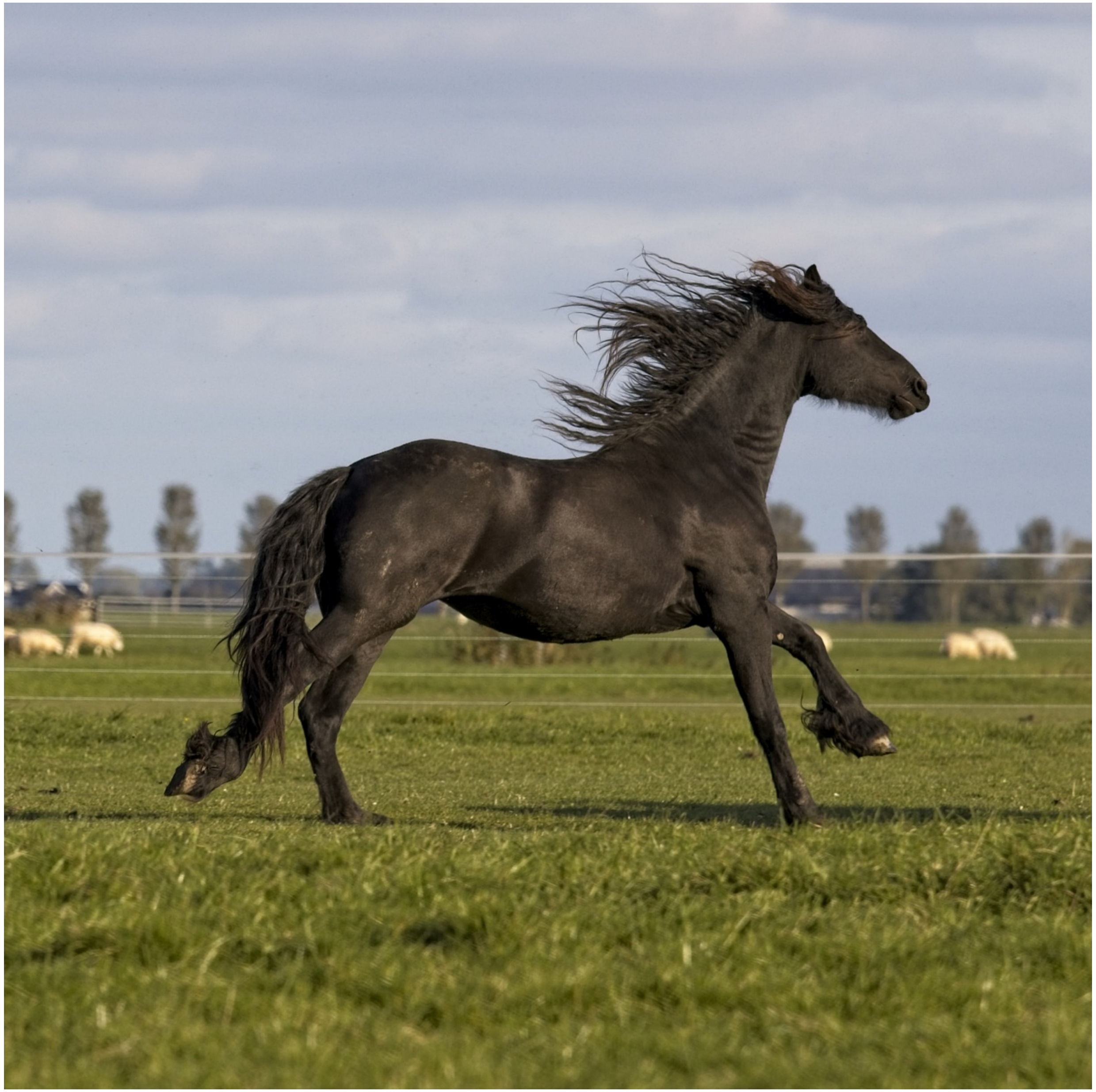

https://pixabay.com/photos/horse-friesian-horse-gallop-paddock-5675542/

3.4. Friesian

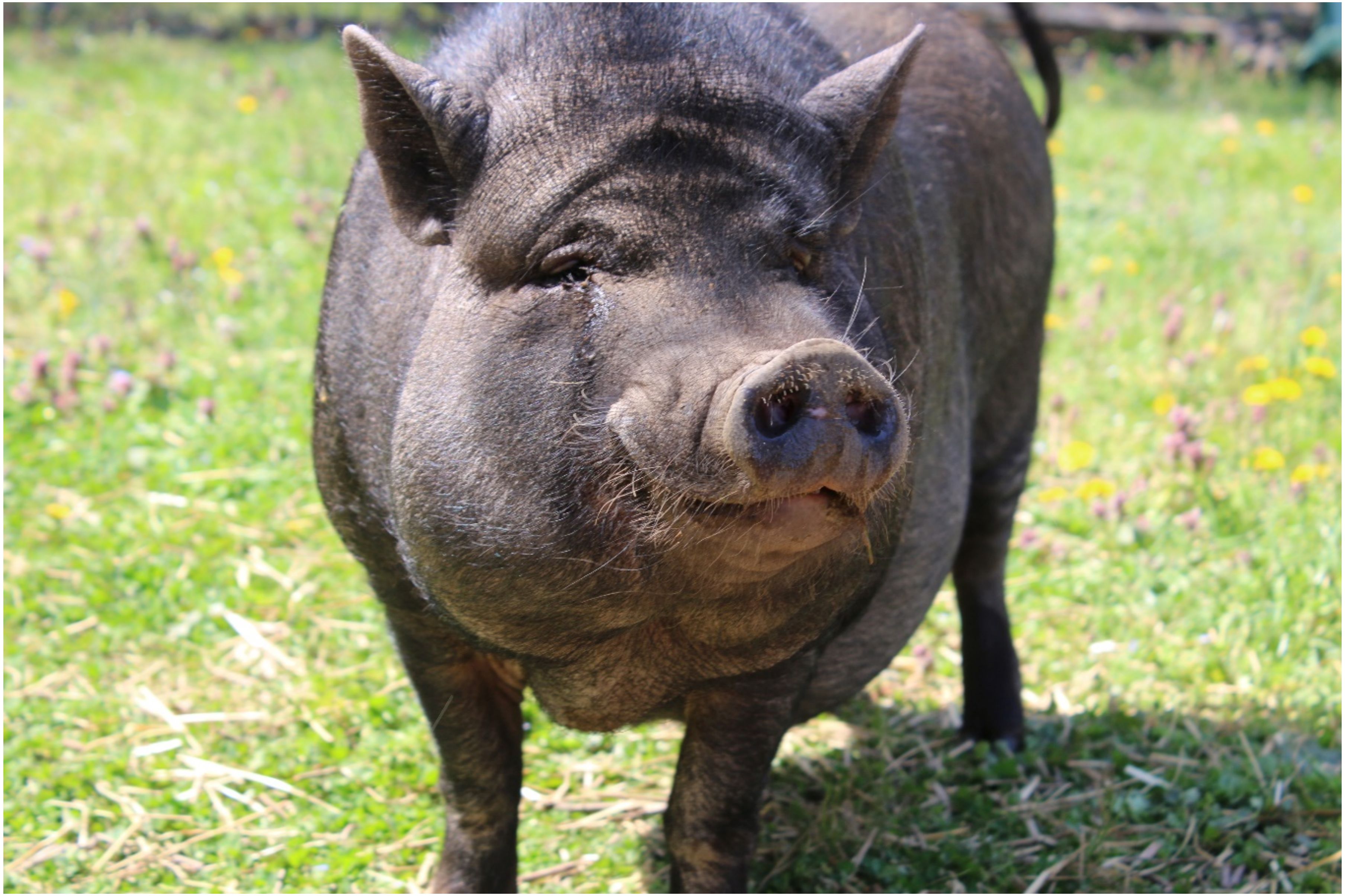

https://unsplash.com/photos/NxvAV87HDT8

4.1. Vietnamese Pot-bellied

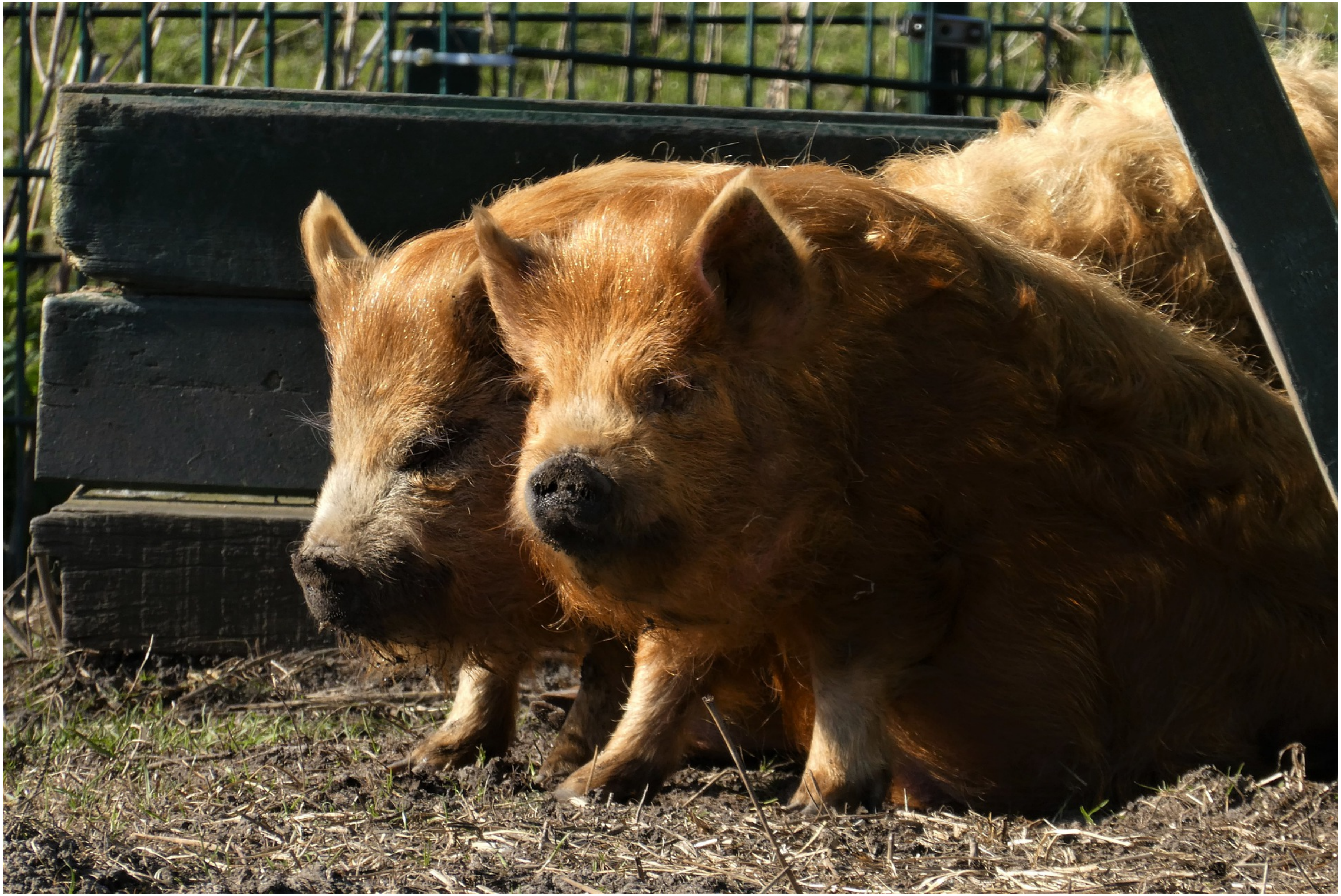

https://pixabay.com/photos/kunekune-pigs-petting-zoo-4962022/

4.2. Kunekune

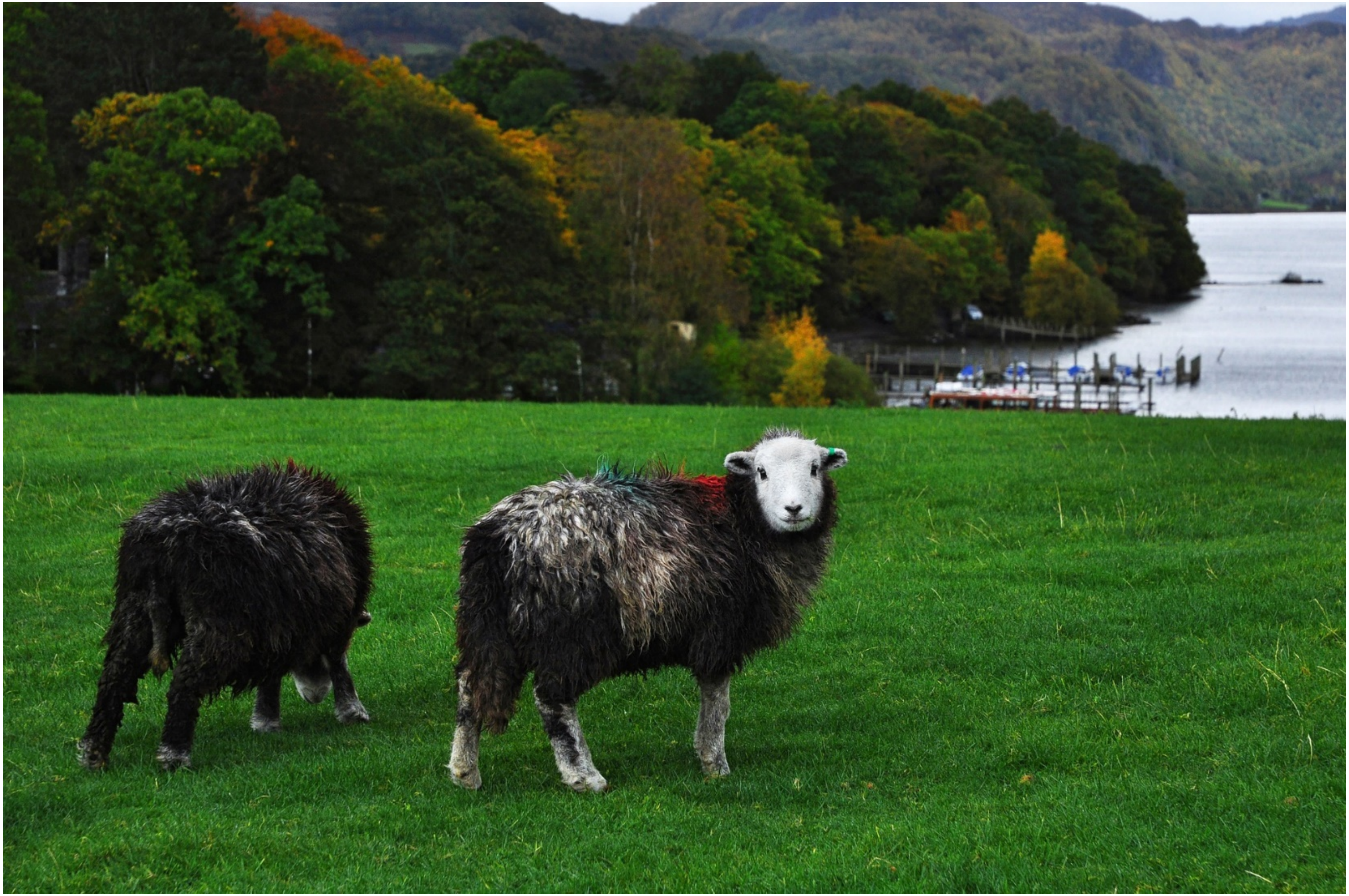

https://pixabay.com/photos/sheep-england-cumbria-derwentwater-1341278/

5.1. Herdwick sheep

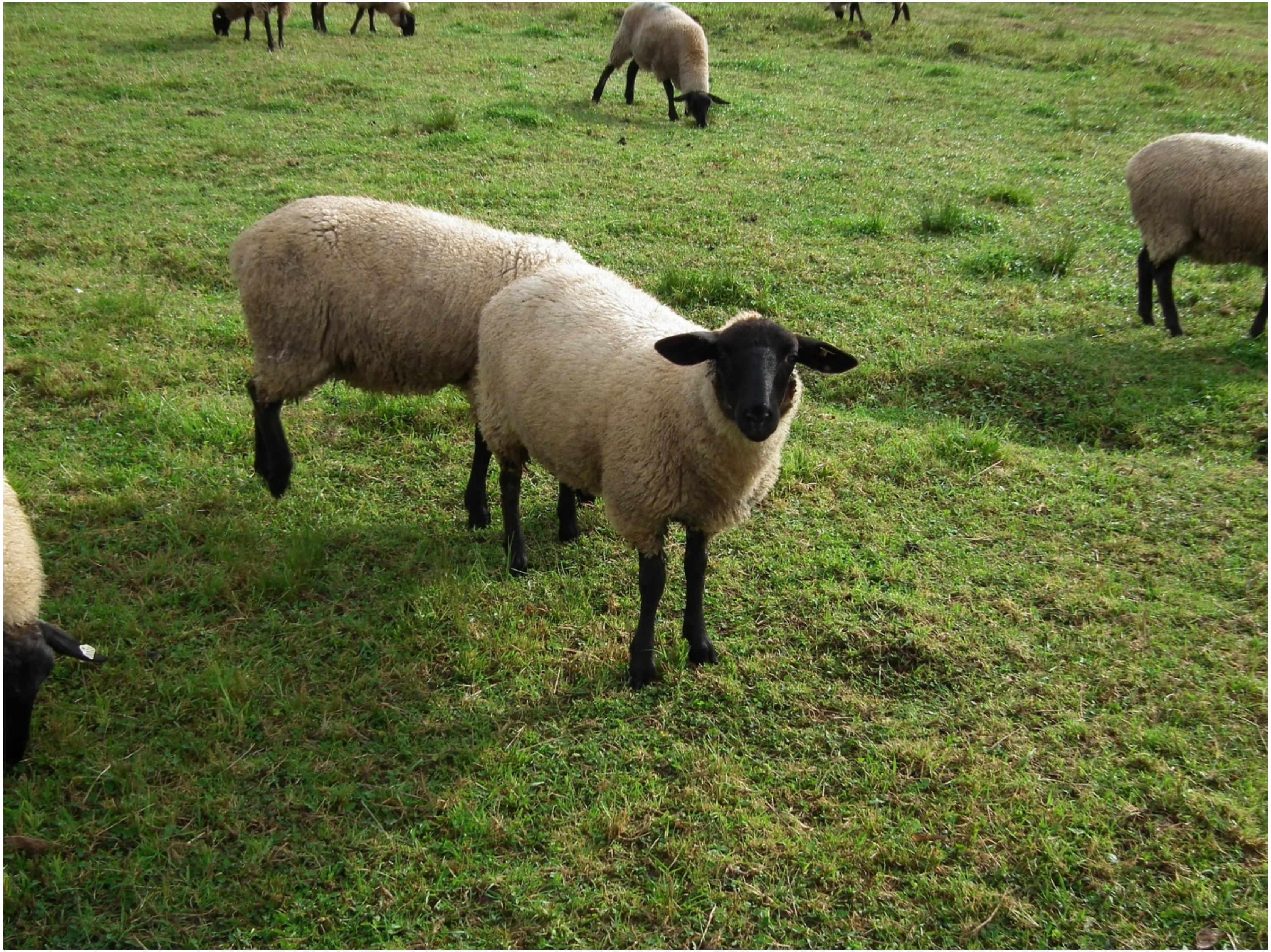

https://pixabay.com/photos/hokkaido-takikawa-autumn-sheep-2734589/

5.2. Suffolk sheep
