# Supplementary material for: Personalized Strategy for Animal-Assisted Therapy for Individuals Based on the Emotions Induced by the Images of Different Animal Species and Breeds
Source: Animals (Basel). 2022 Feb 27;12(5):597. doi: 10.3390/ani12050597 (PMC8909388; doi:10.3390/ani12050597)
Supplement: Supplementary file 1 [file animals-12-00597-s001.zip › Supplementary File 2. Emotions induced for persons by different animal species and breeds.pdf]

**Participant 1**											
**Participant_ID**	**Neutral_Mean**	**Happy_Mean**	**Sad_Mean**	**Angry_Mean**	**Surprised_Mean**	**Scared_Mean**	**Disgusted_Mean**	**Contempt_Mean**	**Valence_Mean**	**Animal_Id**	**Animal**
1	0,6433976	0,04647479	0,02006616	0,02329217	0,01603611	0,01031045	0,001367725	0,007710149	0,01573519	1_1	Dogs
1	0,4910977	0,2308445	0,01062147	0,006426669	0,01010996	0,02519221	0,000228067	0,004805582	0,1952352	1_2	Dogs
1	0,4720244	0,1606682	0,03804983	0,01121461	0,009540212	0,02128029	0,000015197	0,000792384	0,1016403	1_3	Dogs
1	0,5579855	0,06706423	0,03260501	0,00865638	0,005446828	0,01220258	0,00000575	0,001438143	0,02272812	1_4	Dogs
1	0,6113558	0,01342435	0,04442276	0,004900594	0,005209616	0,004712516	0,001377594	0,002178742	-0,03231015	1_5	Dogs
1	0,5724856	0,002992867	0,0673607	0,003499809	0,004313566	0,003543103	0,03874012	0,000788835	-0,09566119	1_6	Dogs
1	0,5245994	0,000437919	0,03301191	0,000948203	0,000839092	0,003014921	0,07603932	0,0004022	-0,09403849	1_7	Dogs
1	0,5678608	0,000001461	0,0886611	0,00427298	0,001522664	0,000091352	0,02539835	0,000186458	-0,09917581	1_8	Dogs
1	0,5760809	0,000000257	0,1022117	0,001843434	0,004267665	0,008026296	0,01916757	0,002957756	-0,1063118	1_9	Dogs
1	0,6708879	0,000000032	0,05378139	0,0004961	0,05593507	0,04035137	0,02986524	0,000987269	-0,08581083	1_10	Dogs
1	0,6049084	0,000000009	0,06662585	0,000165182	0,05947857	0,04642779	0,05484059	0,000460548	-0,1055029	1_11	Dogs
1	0,6202578	0,000000002	0,04365646	0,04330217	0,02152887	0,01870537	0,0250426	0,001387153	-0,07412778	1_12	Dogs
1	0,6536115	0,000023906	0,01062557	0,028338	0,00335456	0,00268638	0,09372903	0,002665371	-0,1151286	1_13	Dogs
1	0,5355642	0,158003	0,05101084	0,101385	0,006549799	0,00613204	0,01651631	0,01415148	0,04327152	2_1	Cats
1	0,1936311	0,7366835	0,000032469	0,008115523	0,01524303	0,000325889	0,000010777	0,004193272	0,728568	2_2	Cats
1	0,3970689	0,489779	0,000009452	0,06493255	0,006064738	0,000094863	0,000003137	0,001932628	0,4248464	2_3	Cats
1	0,4884393	0,3495939	0,000002916	0,04018779	0,01311794	0,01222927	0,000000968	0,002280298	0,2972345	2_4	Cats
1	0,1880458	0,6877103	0,000000006	0,000082408	0,04768723	0,04526461	0,000000002	0,00000747	0,6424457	2_5	Cats
1	0,2150553	0,6317856	0,000000005	0,000062579	0,06537296	0,04238223	0,000000002	0,000023043	0,5894034	2_6	Cats
1	0,3487839	0,3705842	0,000000002	0,000197642	0,1123072	0,02967815	0,000000001	0,000031603	0,3409061	2_7	Cats
1	0,3615364	0,3719408	0,000000001	0,02196087	0,06200752	0,01430907	0	0,007651965	0,3434996	2_8	Cats
1	0,7749726	0,07727136	0,0284668	0,01389761	0,007792248	0,001593803	0,02795296	0,000619008	0,01590031	3_1	Horses
1	0,9974043	0,000000614	0,001368156	0,000000007	0,000001533	0	0,000235071	0,000863074	-0,001367542	3_2	Horses
1	0,9332165	0	0,1123662	0,000001734	0	0	0	0,000198251	-0,1123662	3_3	Horses
1	0,9711837	0,004210615	0,000020562	0,000008662	0,001740089	0,00023631	0,01285361	0,000007824	-0,008706721	3_4	Horses
1	0,9213067	0,03553315	0,01968008	0,000137759	0,00096683	0,001134187	0,01618231	0,000214102	-0,000647282	4_1	Pigs
1	0,9758224	0,000005054	0,02414629	0,000151373	0,004782157	0,000000177	0,001335964	0,001308704	-0,0253902	4_2	Pigs
1	0,6728016	0,006926931	0,2435015	0,09370001	0,00437677	0,000260314	0,001859229	0,001082928	-0,2560673	5_1	Sheep
1	0,8454415	0,02315811	0,000006327	0,000001944	0,02124327	0,000320443	0,007651279	0,000291013	0,01550195	5_2	Sheep

**Participant 2**											
**Participant_ID**	**Neutral_Mean**	**Happy_Mean**	**Sad_Mean**	**Angry_Mean**	**Surprised_Mean**	**Scared_Mean**	**Disgusted_Mean**	**Contempt_Mean**	**Valence_Mean**	**Animal_Id**	**Animal**
2	0,6093686	0,3474237	0,000000467	0,001317008	0,000593695	0,002164755	0,006233061	0,000163761	0,3393543	1_1	Dogs
2	0,7075568	0,2733516	0,000000001	0,001942482	0,000000003	0,00064377	0,003059614	0,000139242	0,2689081	1_2	Dogs
2	0,8046162	0,1179776	0,000792091	0,000018483	0,000002302	0,000154052	0,002223207	0,000000143	0,1155711	1_3	Dogs
2	0,9876319	0	0	0,00150779	0,000521088	0	0	0,000040317	-0,00150779	1_4	Dogs
2	0,8938686	0,06303625	0,00006001	0,000006112	0,0002996	0,000060811	0,002466455	0,00004519	0,06051803	1_5	Dogs
2	0,9610071	0,000720485	0,000193589	0,000015011	0,000503606	0	0,03214308	0,000554951	-0,03153072	1_6	Dogs
2	0,7925503	0,1620708	0,000248305	0,002148143	0,00234775	0,000290796	0,0384597	0,000815183	0,1226005	1_7	Dogs
2	0,9891973	0	0,001107066	0,002350335	0,000459329	0,000006958	0,003773884	0,00035082	-0,005892123	1_8	Dogs
2	0,9809313	0	0,000000008	0,004284153	0,000920776	0	0,01779431	0,000048026	-0,01996807	1_9	Dogs
2	0,9660301	0	0,001420078	0,000062123	0,00075042	0	0,03493626	0,000679172	-0,03503249	1_10	Dogs
2	0,9665417	0	0,01837217	0,000200854	0,0140316	0	0,01730059	0,00000156	-0,03289532	1_11	Dogs
2	0,7761752	0,1736681	0,000371956	0,000051171	0,001435318	0,000066638	0,02249648	0,000078141	0,1508698	1_12	Dogs
2	0,9536549	0	0,000147444	0,000000004	0,02235243	0,000134604	0,001863735	0,000649244	-0,002056562	1_13	Dogs
2	0,9823899	0	0,001467707	0,000000025	0,000236722	0	0,003161393	0,000148414	-0,00450851	2_1	Cats
2	0,6583049	0,2377849	0,000106674	0,000014071	0	0,000555883	0,004605835	0,000012774	0,233174	2_2	Cats
2	0,9509493	0,01180641	0,000528103	0	0,000496291	0,000311415	0,008176118	0	0,003272361	2_3	Cats
2	0,984688	0	0,000761404	0,003037303	0,001253567	0	0,002306038	0,00048277	-0,004506562	2_4	Cats
2	0,98353	0	0,000285517	0,002760184	0,002245473	0,000309393	0,007418782	0,000332031	-0,007828002	2_5	Cats
2	0,9760544	0	0,001346357	0,000005638	0,008698864	0	0,01244719	0,000091707	-0,01251478	2_6	Cats
2	0,9750112	0	0,000106868	0,000022182	0,001815655	0	0,01364126	0,000305692	-0,01365808	2_7	Cats
2	0,9759024	0	0,000012496	0,000263606	0,000760248	0	0,01422496	0,000002003	-0,01423481	2_8	Cats
2	0,9538609	0,000060012	0,05572913	0,001378934	0,000540764	0,000261076	0,004563229	0,001367953	-0,05718118	3_1	Horses
2	0,9213924	0,000507831	0,0939623	0,003193942	0,00334727	0,00034892	0,01302697	0,003138662	-0,1038298	3_2	Horses
2	0,9760662	0,000005642	0,02678321	0,000091703	0,000343083	0,000523327	0,001320817	0,00130752	-0,02796787	3_3	Horses
2	0,9435555	0	0,09421668	0	0,000000537	0	0,000000009	0,000000067	-0,09421668	3_4	Horses
2	0,9533098	0,000246174	0,05502451	0,000433327	0,000184838	0,000068958	0,003871841	0,002753771	-0,05510562	4_1	Pigs
2	0,9592477	0,001018802	0,05992731	0,000054374	0,000138528	0,000006164	0,000827226	0,000091797	-0,0594908	4_2	Pigs

2	0,9798077	0,000003489	0,01626943	0,00000112	0,000041681	0	0,000424208	0,000331874	-0,01640924	5_1	Sheep
2	0,6027975	0	0,1948961	0	0,000394338	0	0	0,000990717	-0,1948961	5_2	Sheep
**Participant 3**											
**Participant_ID**	**Neutral_Mean**	**Happy_Mean**	**Sad_Mean**	**Angry_Mean**	**Surprised_Mean**	**Scared_Mean**	**Disgusted_Mean**	**Contempt_Mean**	**Valence_Mean**	**Animal_Id**	**Animal**
3	0,9667099	0,000080758	0,02788216	0,000634129	0,000117058	0,000040866	0,002170243	0,000717972	-0,02946363	1_1	Dogs
3	0,9843084	0	0,01959326	0,000000019	0,000177553	0,000030615	0,000005048	0,00003073	-0,01959326	1_2	Dogs
3	0,9875254	0	0,009819509	0	0,005274148	0,000000003	0,001942158	0,000083034	-0,01141404	1_3	Dogs
3	0,9923805	0,000000981	0,006259254	0	0,001700815	0,00000041	0,000093348	0,000693796	-0,006258272	1_4	Dogs
3	0,9935205	0,000063823	0,00269884	0,000226111	0,000699745	0,000055725	0,000055453	0,000280616	-0,002707872	1_5	Dogs
3	0,961893	0,000419848	0,02816252	0,000000869	0,01646867	0,00002868	0,01608812	0,000460804	-0,0407886	1_6	Dogs
3	0,985854	0,000024767	0,02321731	0,000144159	0,000039879	0,000006879	0,000862262	0,000545517	-0,02360996	1_7	Dogs
3	0,9873384	0,00015776	0,01659605	0,000041475	0,000787279	0,000011955	0,00079709	0,000086262	-0,01689586	1_8	Dogs
3	0,9858674	0,000002518	0,02136833	0,00004147	0,000381884	0,000004805	0,000767354	0,000882445	-0,02191399	1_9	Dogs
3	0,9496442	0,00000014	0,0901738	0,000007069	0,001142366	0,000230497	0,00001968	0,001199853	-0,09038556	1_10	Dogs
3	0,9902531	0,000009905	0,01217566	0,000002781	0,000596625	0,000057729	0,000178938	0,000126401	-0,01220539	1_11	Dogs
3	0,9954544	0,000016062	0,00382241	0,000027337	0,001775797	0,000136803	0,000500129	0,000913108	-0,00387925	1_12	Dogs
3	0,9935417	0	0,01021627	0,000069484	0,000125632	0	0,000092222	0,000228221	-0,01037368	1_13	Dogs
3	0,9034258	0,000222921	0	0,0044047	0,0225592	0,004528895	0,001312928	0,001991204	-0,004679419	2_1	Cats
3	0,825505	0,000279731	0,004884248	0,02384828	0,01521108	0,01296707	0,001525828	0,003171906	-0,02448261	2_2	Cats
3	0,8147249	0,000018067	0,1740507	0,003290862	0,000975502	0,005968861	0,000098282	0,0010093	-0,1740326	2_3	Cats
3	0,9647751	0,000001069	0,03917393	0,000194651	0,0000577	0,000391928	0,000005813	0,000634296	-0,03917285	2_4	Cats
3	0,9782628	0,000000025	0,04001252	0,000004555	0,00000135	0,000009172	0,000000136	0,000018175	-0,0400125	2_5	Cats
3	0,903887	0,000000006	0,1894183	0,000001075	0,000000319	0,000002165	0,000000032	0,00005874	-0,1894183	2_6	Cats
3	0,8723387	0,000000002	0,2517718	0,000000383	0,000000114	0,000000772	0,000000011	0,000070646	-0,2517718	2_7	Cats
3	0,8476367	0,000000001	0,3005928	0,000000158	0,000000047	0,000000319	0,000000005	0,000031433	-0,3005928	2_8	Cats
3	0,9814171	0,000033064	0,02278923	0,000140534	0,000546872	0,000661071	0,00457333	0,001072402	-0,02692018	3_1	Horses
3	0,8929052	0,000060306	0,009396609	0,02170107	0,001188071	0,003774578	0,000594458	0,001480232	-0,02767951	3_2	Horses
3	0,8327897	0,001380838	0,03084939	0,0239499	0,003214836	0,002883099	0,002665106	0,003657185	-0,03809629	3_3	Horses
3	0,95363	0	0,08346561	0,000000004	0,001243149	0,000000004	0	0,003586331	-0,08346561	3_4	Horses

3	0,448086	0,2078444	0,02529496	0,005942093	0,001696053	0,005651936	0,01039162	0,005276674	0,1698434	4_1	Pigs
3	0,5166577	0,04405503	0,06603654	0,004751754	0,0269364	0,02038287	0,003045666	0,008126038	-0,03032591	4_2	Pigs
3	0,9869297	0,000004621	0,00271047	0,000053685	0,001329549	0,000820726	0,001545843	0,002250208	-0,004515662	5_1	Sheep
3	0,9715439	0,000000184	0,02584296	0,000106225	0,000140907	0,000075845	0,008165347	0,007377714	-0,0301427	5_2	Sheep
**Participant 4**											
**Participant_ID**	**Neutral_Mean**	**Happy_Mean**	**Sad_Mean**	**Angry_Mean**	**Surprised_Mean**	**Scared_Mean**	**Disgusted_Mean**	**Contempt_Mean**	**Valence_Mean**	**Animal_Id**	**Animal**
4	0,6401392	0,08620657	0,01457104	0,000013028	0,00544125	0,003499772	0,000261221	0,02109378	0,06805263	1_1	Dogs
4	0,6199158	0,000236101	0,1281789	0,005670412	0	0,000301099	0,000823508	0,001068598	-0,1287361	1_2	Dogs
4	0,7060562	0,004869727	0,06267774	0,005532072	0	0	0,002805392	0,005180563	-0,05991543	1_3	Dogs
4	0,7442588	0,000000007	0,04217121	0,03973868	0	0,00000006	0,003010949	0,000240917	-0,06709756	1_4	Dogs
4	0,6142043	0,0006063	0,1781577	0,02701349	0,00095173	0	0,01346761	0,001000937	-0,197801	1_5	Dogs
4	0,6298794	0,0036358	0,1598743	0,003072298	0,001679084	0,004839886	0,00479997	0,002357214	-0,1607798	1_6	Dogs
4	0,6558719	0,00540747	0,2114823	0,00517416	0,000421842	0,000373056	0,009851594	0,004091396	-0,2087624	1_7	Dogs
4	0,6275737	0,01361462	0,1359453	0,01753154	0,001907311	0,000292379	0,000778078	0,008535203	-0,1306884	1_8	Dogs
4	0,7423272	0,000142654	0,1295904	0,004459923	0,000530475	0,007472597	0,003489577	0,007356484	-0,1402811	1_9	Dogs
4	0,7364466	0,002552443	0,09704598	0,009655276	0,003005682	0,007268011	0,02307595	0,007161804	-0,1145938	1_10	Dogs
4	0,7557104	0,001763732	0,08411371	0,01777225	0,001229163	0,001160484	0,00261052	0,006998546	-0,1001812	1_11	Dogs
4	0,7218685	0,004150147	0,1481358	0,001450339	0,001391034	0,000123857	0,001532227	0,000310483	-0,1455151	1_12	Dogs
4	0,5641925	0,04935619	0,2702781	0,007835875	0,002679216	0,000792657	0,003324687	0,000520882	-0,2241002	1_13	Dogs
4	0,9883021	0,000000077	0,0147867	0	0,000001213	0,000000017	0,000000603	0,000030545	-0,01478662	2_1	Cats
4	0,9791657	0,000024253	0,02714146	0	0,000074401	0,000000012	0,000007307	0,000106248	-0,02711721	2_2	Cats
4	0,9934695	0	0,004855644	0,000000228	0,000000126	0,00000055	0,000002701	0,000062985	-0,004855644	2_3	Cats
4	0,9335778	0	0,1196014	0	0	0	0,000000002	0,000178483	-0,1196014	2_4	Cats
4	0,987843	0	0,01636021	0,000019972	0,000022361	0	0,000002355	0,000344881	-0,01636021	2_5	Cats
4	0,998363	0	0,001553428	0,00003361	0,000013991	0	0	0,000466112	-0,001576817	2_6	Cats
4	0,9936473	0	0,007750353	0,001675682	0,000525335	0	0	0,000905161	-0,009372247	2_7	Cats
4	0,9294	0	0,1002536	0	0	0	0,000058533	0,000445302	-0,1002536	2_8	Cats
4	0,8928754	0,001211357	0,01150282	0,01903454	0,002902722	0,002663854	0,002874918	0,002010862	-0,0237416	3_1	Horses
4	0,545894	0,04118307	0,000746317	0,005848111	0,000114773	0,000010549	0,002356705	0,00031779	0,03307771	3_2	Horses

4	0,5055752	0,005103359	0,05994073	0,05031316	0,007080444	0	0,04419283	0,000092333	-0,108207	3_3	Horses
4	0,9466375	0,001843851	0,000073052	0,01960625	0,000371172	0,001159985	0,005142278	0,004240246	-0,02136082	3_4	Horses
4	0,635717	0,002922577	0,03519866	0,007098441	0,003853944	0,000358835	0,06654542	0,000297362	-0,07018884	4_1	Pigs
4	0,5768839	0,002186422	0,04508597	0,0356194	0,001925397	0,000318486	0,0338119	0,000997238	-0,0859805	4_2	Pigs
4	0,8497183	0,001769137	0,04116225	0,01793809	0,001453406	0,002309965	0,00118168	0,007971554	-0,05260422	5_1	Sheep
4	0,8048372	0,000516328	0,09782766	0,01628406	0,000822302	0,001293065	0,001445924	0,009080737	-0,1021145	5_2	Sheep
**Participant 5**											
**Participant_ID**	**Neutral_Mean**	**Happy_Mean**	**Sad_Mean**	**Angry_Mean**	**Surprised_Mean**	**Scared_Mean**	**Disgusted_Mean**	**Contempt_Mean**	**Valence_Mean**	**Animal_Id**	**Animal**
5	0,5912697	0,03048824	0,05227102	0,000000023	0,000000002	0,000444602	0,003369734	0,000122332	-0,02529158	1_1	Dogs
5	0,4961915	0,1765466	0,00819232	0,000019923	0,000136296	0,000183875	0,006602414	0,000176075	0,1642466	1_2	Dogs
5	0,5296482	0,005529772	0,001567662	0,01133692	0,000001609	0	0,01132051	0,000003918	-0,01460772	1_3	Dogs
5	0,4908317	0,05105464	0,007273715	0,002038102	0,00000048	0	0,01974916	0,000040126	0,02568711	1_4	Dogs
5	0,4031264	0,1271052	0,02614017	0,000046041	0,000204316	0,000446041	0,1117597	0,000579397	0,01117523	1_5	Dogs
5	0,4147799	0,284829	0,03157473	0,000700239	0,00089192	0,00033915	0,04528164	0,000158126	0,2380561	1_6	Dogs
5	0,5774177	0,1203386	0,001398954	0,000002258	0,000609695	0,000246699	0,000000267	0,000000201	0,1186945	1_7	Dogs
5	0,544246	0,1102127	0,009468235	0	0	0,000001168	0,004756497	0,000230735	0,09742317	1_8	Dogs
5	0,4887945	0	0,02905094	0,004813273	0,000604726	0,000095349	0,08140226	0,00101834	-0,09114133	1_9	Dogs
5	0,438879	0,04839216	0,1458055	0,00565692	0,00091929	0,000351193	0,1323074	0,004031667	-0,1404006	1_10	Dogs
5	0,4017001	0,0000008	0,1574912	0,000704294	0,000089292	0,00000647	0,1452325	0,000430712	-0,1993708	1_11	Dogs
5	0,5389815	0,0492619	0,04033674	0,000257714	0,000041013	0,000206922	0,019942	0,000363803	0,004356736	1_12	Dogs
5	0,4977951	0,1647631	0,001467949	0,001995032	0,000000003	0	0,000134733	0,000590203	0,1614013	1_13	Dogs
5	0,9913875	0,000007148	0,01080279	0,000040894	0,000102061	0,000046796	0	0,000151142	-0,01080471	2_1	Cats
5	0,9788363	0	0,03691506	0,000022664	0,000110423	0,000009474	0,000076509	0,000820406	-0,036996	2_2	Cats
5	0,9882017	0	0,01512458	0,000003282	0,000817247	0,000010909	0,000302358	0,001730241	-0,01518769	2_3	Cats
5	0,9891027	0	0	0,000000281	0,000003246	0,000000689	0	0,000048809	-0,000000689	2_4	Cats
5	0,978268	0	0,02751254	0,000023064	0,001554735	0	0,00018513	0,00020012	-0,02751254	2_5	Cats
5	0,9949442	0,000004512	0,001797276	0,000000168	0,005344995	0,000468687	0,00084907	0,000208119	-0,002787371	2_6	Cats
5	0,9905131	0	0,00976773	0,000019391	0,000018106	0,000133273	0,000212366	0,004132064	-0,009959634	2_7	Cats
5	0,9653059	0,001576124	0,01949386	0,000920716	0,000865511	0,000221415	0,02690405	0,000796549	-0,04467361	2_8	Cats

5	0,4705164	0,0665796	0,06737389	0,001365058	0,000472806	0,000336755	0,07315967	0,000502977	-0,03093417	3_1	Horses
5	0,930777	0,000915477	0,001213463	0,01639928	0,004699723	0,008129927	0,003674697	0,000730779	-0,02119742	3_2	Horses
5	0,4083647	0,000014095	0,2183634	0,1852092	0,000404442	0,000658331	0,002422067	0,001093281	-0,3765245	3_3	Horses
5	0,6174364	0	0,03070961	0,03209984	0,001801768	0	0,05469052	0,000086686	-0,0941231	3_4	Horses
5	0,8528942	0,001734079	0,03566564	0,03398162	0,002488279	0,005351252	0,004229775	0,002494483	-0,06636565	4_1	Pigs
5	0,8500018	0,001615409	0,05237159	0,007573283	0,02245731	0,000775904	0,003612767	0,00453019	-0,05845735	4_2	Pigs
5	0,5861474	0,006771392	0,03694827	0,02629608	0,000410058	0,0002413	0,05975827	0,00041587	-0,08230197	5_1	Sheep
5	0,7910239	0,0685185	0,03717769	0,001376006	0,000965389	0,000121881	0,01397763	0,002979696	0,01920087	5_2	Sheep
**Participant 6**											
**Participant_ID**	**Neutral_Mean**	**Happy_Mean**	**Sad_Mean**	**Angry_Mean**	**Surprised_Mean**	**Scared_Mean**	**Disgusted_Mean**	**Contempt_Mean**	**Valence_Mean**	**Animal_Id**	**Animal**
6	0,8430544	0,000134787	0,0296535	0,02476146	0,05865685	0,002008101	0,002775202	0,004971676	-0,04438024	1_1	Dogs
6	0,8474074	0,005068785	0,04042478	0,02155445	0,00169028	0,00706044	0,002226831	0,01048797	-0,04877979	1_2	Dogs
6	0,9267796	0,000927173	0,02031498	0,00527568	0,001397097	0,002732948	0,003824151	0,003189897	-0,0226508	1_3	Dogs
6	0,857906	0,00314908	0,04117011	0,002262157	0,01821171	0,004658997	0,01197676	0,00403991	-0,046921	1_4	Dogs
6	0,9547051	0,000400465	0,000259343	0,004114082	0,02564138	0,00010287	0,000678301	0,000723787	-0,004003405	1_5	Dogs
6	0,8689638	0,007170942	0,03962611	0,006263139	0,002240932	0,001209543	0,01134532	0,000689613	-0,03434552	1_6	Dogs
6	0,9598205	0,001104985	0,006117377	0,001294778	0,01090585	0,000531866	0,000907202	0,00030368	-0,006363092	1_7	Dogs
6	0,9335316	0,000908177	0,004163187	0,004973754	0,03630749	0,004501947	0,001667426	0,001982398	-0,008972813	1_8	Dogs
6	0,8881175	0,004191492	0,01027503	0,001404412	0,05682889	0,001719062	0,000598146	0,000658125	-0,007446633	1_9	Dogs
6	0,9083731	0,001407152	0,05146643	0,001965345	0,01498825	0,000788688	0,001184377	0,000444121	-0,05042398	1_10	Dogs
6	0,8669376	0,00151773	0,02770847	0,0243182	0,02458557	0,001861251	0,004303615	0,000778051	-0,04434879	1_11	Dogs
6	0,9236584	0,000610466	0,02226479	0,000216802	0,03261057	0,00011226	0,000497511	0,000569743	-0,0218411	1_12	Dogs
6	0,9249306	0,003451035	0,009295165	0,000790265	0,000694926	0,002288038	0,000176927	0,000201048	-0,006933523	1_13	Dogs
6	0,7521251	0,009989313	0,1425523	0,02383012	0,000834785	0,009122958	0,003977378	0,007853319	-0,1484092	2_1	Cats
6	0,722095	0,01024788	0,1256014	0,01520569	0,00500245	0,004831017	0,002963478	0,002168979	-0,1308225	2_2	Cats
6	0,7802057	0,001020033	0,07585383	0,005931199	0,005758853	0,001933439	0,003012972	0,001490009	-0,07981484	2_3	Cats
6	0,7335003	0,001194079	0,1368876	0,0114305	0,001670148	0,000727	0,003959063	0,001352531	-0,1430184	2_4	Cats
6	0,7800924	0,001881703	0,07179992	0,01192864	0,000230697	0,004401487	0,007744411	0,006494272	-0,08392957	2_5	Cats
6	0,8334482	0,000067428	0,03065511	0,02485425	0,001509817	0,006575555	0,003598939	0,003706236	-0,05024442	2_6	Cats

6	0,809604	0,000314184	0,05454968	0,02275863	0,001099218	0,001114071	0,002345501	0,005458051	-0,06673376	2_7	Cats
6	0,7912132	0,002574293	0,04440903	0,008693662	0,01561961	0,008712292	0,001930003	0,00796748	-0,05089042	2_8	Cats
6	0,8518319	0,01518166	0,06876451	0,02268721	0,01683645	0,000752532	0,002481066	0,001304867	-0,07596546	3_1	Horses
6	0,3655984	0,000352979	0,3958026	0,08196208	0,000780345	0,00787107	0,01682855	0,005207571	-0,457297	3_2	Horses
6	0,6702856	0,000257236	0,1299195	0,000500602	0,000893952	0,01865693	0,002989131	0,000588229	-0,1370448	3_3	Horses
6	0,876678	0,001888889	0,06042458	0,001875602	0,008566717	0,000235535	0,001823456	0,001457007	-0,05862656	3_4	Horses
6	0,598644	0,000631515	0,1200911	0,1210098	0,000851178	0,000201157	0,01609773	0,007266752	-0,230464	4_1	Pigs
6	0,3190891	0,000117586	0,1554425	0,3394276	0,000161593	0,005309894	0,006122536	0,003250312	-0,4825043	4_2	Pigs
6	0,8978245	0,0012639	0,02725286	0,02128548	0,01548539	0,005310327	0,001102774	0,006306157	-0,04318557	5_1	Sheep
6	0,972033	0,000029005	0,004119666	0,000863614	0,0111486	0	0,000407098	0,007269755	-0,004090661	5_2	Sheep
**Participant 7**											
**Participant_ID**	**Neutral_Mean**	**Happy_Mean**	**Sad_Mean**	**Angry_Mean**	**Surprised_Mean**	**Scared_Mean**	**Disgusted_Mean**	**Contempt_Mean**	**Valence_Mean**	**Animal_Id**	**Animal**
7	0,550193	0	0,2976285	0,04893876	0,000545206	0,00099607	0,04631127	0,01365626	-0,3325251	1_1	Dogs
7	0,5913799	0	0,1863125	0,101843	0,000738761	0,003003679	0,006807928	0,007248929	-0,2781874	1_2	Dogs
7	0,7323179	0	0,1347879	0,05020287	0,001043682	0,001675765	0,00022919	0,005887022	-0,1799984	1_3	Dogs
7	0,4480328	0	0,372447	0,04478632	0,000065905	0,005042762	0,00921943	0,01837933	-0,41124	1_4	Dogs
7	0,4788838	0	0,3675102	0,04332457	0,000172536	0,002468199	0,000967412	0,0008233	-0,3961376	1_5	Dogs
7	0,5369281	0	0,229305	0,1205929	0,001024927	0,001352521	0,004022665	0,00182437	-0,331653	1_6	Dogs
7	0,4267247	0	0,3727292	0,1461859	0,001254332	0,000391809	0	0,00285717	-0,4779667	1_7	Dogs
7	0,5131108	0,000031304	0,3069601	0,0824619	0,000567472	0,002916037	0,000613519	0,000115034	-0,3676147	1_8	Dogs
7	0,5817676	0	0,246503	0,07716776	0,000088117	0,000897133	0	0,001256907	-0,3107851	1_9	Dogs
7	0,3984388	0	0,3797045	0,1328054	0,000976547	0,001602389	0,000709215	0,003793049	-0,493825	1_10	Dogs
7	0,3762807	0	0,4122347	0,08381271	0,000078248	0,001637567	0,000203227	0,000449387	-0,4719715	1_11	Dogs
7	0,369322	0	0,3776155	0,1015237	0,000000012	0,000622058	0,01249148	0,003574267	-0,4510341	1_12	Dogs
7	0,4492708	0	0,3186922	0,1248191	0,000258191	0,000920192	0	0	-0,4243963	1_13	Dogs
7	0,4946263	0,04505795	0,08968638	0,002504034	0,000471555	0,000019951	0,1034481	0,00030337	-0,07984438	2_1	Cats
7	0,4076627	0,2181433	0,04317588	0,000779255	0,000150335	0,00032217	0,01142966	0,001268744	0,1669994	2_2	Cats
7	0,4957853	0,04244377	0,01477798	0,001579666	0,000030968	0,000579239	0,002311406	0,000387893	0,02425923	2_3	Cats
7	0,481813	0	0,02161955	0,02429259	0,000240914	0,001338996	0,0486884	0,000369802	-0,0698697	2_4	Cats

7	0,5005144	0,04736612	0,01934092	0,00068208	0,000106066	0,000088218	0,03143555	0,000630624	0,001657552	2_5	Cats
7	0,5148625	0,06389512	0,001041468	0,002710563	0,000000167	0,000169286	0,02157694	0,000515806	0,03876563	2_6	Cats
7	0,4595893	0,01509623	0,01230808	0,008654454	0,00017801	0,000021435	0,09547919	0,000199688	-0,08957002	2_7	Cats
7	0,5541883	0,000005263	0,000007502	0,004138769	0	0,000018966	0,001729754	0,000006617	-0,005805904	2_8	Cats
7	0,5181999	0,000091094	0,1752716	0,1446043	0,00071481	0,006375139	0,008449149	0,001995014	-0,2977023	3_1	Horses
7	0,6508357	0,001416266	0,1788246	0,000198914	0,000414823	0,004420316	0,000200968	0,001990187	-0,1774095	3_2	Horses
7	0,5407046	0,01927478	0,004254689	0,00180842	0,000292529	0,03614461	0,000360364	0,02997946	-0,02074702	3_3	Horses
7	0,5900356	0,000109339	0,1886294	0,05587506	0,000549382	0,000493833	0,006863746	0,00056226	-0,2374025	3_4	Horses
7	0,9010031	0,004030661	0,02471651	0,000665702	0,000950785	0,00197507	0,000287492	0,00186764	-0,02144558	4_1	Pigs
7	0,7270511	0,001512331	0,235275	0,004058025	0,000916642	0,01294235	0,008907776	0,001401195	-0,2354073	4_2	Pigs
7	0,677851	0,00005343	0,1601772	0,09399949	0,000939127	0,00131878	0,004388622	0,004137534	-0,2421472	5_1	Sheep
7	0,5400432	0,000056266	0,3609771	0,03880405	0,001072663	0,005824891	0,003614656	0,001944892	-0,3858913	5_2	Sheep
**Participant 8**											
**Participant_ID**	**Neutral_Mean**	**Happy_Mean**	**Sad_Mean**	**Angry_Mean**	**Surprised_Mean**	**Scared_Mean**	**Disgusted_Mean**	**Contempt_Mean**	**Valence_Mean**	**Animal_Id**	**Animal**
8	0,4815421	0,29421	0,02091096	0,000088477	0,00000005	0,000678117	0,000378455	0,004465042	0,2731892	1_1	Dogs
8	0,5844611	0,1092464	0,01829865	0,000025104	0	0,002456079	0,000187907	0,001353166	0,08990575	1_2	Dogs
8	0,6509289	0,002682423	0,08835526	0,000112838	0,000169402	0,04244396	0,006850021	0,001564983	-0,1236135	1_3	Dogs
8	0,7563101	0,0000001	0,135721	0,003713561	0,000986324	0,01641354	0,005598273	0,000221532	-0,1432529	1_4	Dogs
8	0,6672012	0,00389439	0,2448725	0,000517781	0,000111756	0,00568918	0,02935827	0,002209908	-0,240978	1_5	Dogs
8	0,7515516	0,000000001	0,02739295	0,000003854	0,000020713	0,0181343	0,000000098	0,0034568	-0,04288371	1_6	Dogs
8	0,6515013	0,01881293	0,1260245	0,000583165	0,000606759	0,002766763	0,01767228	0,005538696	-0,1074308	1_7	Dogs
8	0,701651	0,003343267	0,1302862	0,005329337	0,000641825	0,02983394	0,00631988	0,00096816	-0,1367346	1_8	Dogs
8	0,7709302	0,000000002	0,1251585	0,002854384	0,000272321	0,01163847	0,00785743	0,000018445	-0,128205	1_9	Dogs
8	0,7115795	0	0,08872151	0,001770374	0,000721629	0,0274211	0,000000034	0,000897669	-0,1147639	1_10	Dogs
8	0,5095884	0,000808701	0,2970513	0,004295705	0,003472368	0,007534885	0,05166075	0,002648517	-0,2985333	1_11	Dogs
8	0,7450712	0,001855375	0,1449626	0,001433749	0,000200784	0,003200393	0,02397003	0,000425543	-0,1447196	1_12	Dogs
8	0,4150983	0,308402	0,03378483	0,000000022	0,000378186	0,001275388	0,003869197	0,02097137	0,2738878	1_13	Dogs
8	0,7338175	0,005267084	0,05747548	0,01316481	0,01845209	0,01243141	0,002247218	0,002408	-0,06564897	2_1	Cats
8	0,9073441	0	0,001973459	0,01333942	0,02588473	0,001650349	0,000924327	0,000031925	-0,01423086	2_2	Cats

8	0,9199831	0	0,008226885	0,00069016	0,03314409	0,000024962	0,00069651	0,00044763	-0,008437325	2_3	Cats
8	0,9205478	0	0,001271397	0,01202737	0,04470389	0,000148378	0,000590124	0,000844605	-0,01298582	2_4	Cats
8	0,8670109	0	0,02222277	0,03872217	0,02170345	0,002073731	0,000943687	0,001393353	-0,0476754	2_5	Cats
8	0,8840893	0,000291963	0,03752598	0,01201293	0,009508057	0,007181939	0,002324404	0,000884085	-0,04793324	2_6	Cats
8	0,9102209	0,000117564	0,01236953	0,007971329	0,001704804	0,000861802	0,000245223	0,00057445	-0,01603504	2_7	Cats
8	0,9623349	0,000000008	0,004415714	0,000843344	0,009197896	0,00000005	0,000109032	0,000437157	-0,005230096	2_8	Cats
8	0,8591517	0,000709152	0,02975901	0,000642358	0,000501163	0,009687147	0,002813173	0,000978479	-0,03865428	3_1	Horses
8	0,4900821	0,0877734	0,02028534	0,003667027	0,000586971	0,01731246	0,009152604	0,004068279	0,0487376	3_2	Horses
8	0,8300568	0,0111915	0,02840053	0,01569245	0,01298789	0,009531545	0,005265116	0,004315879	-0,03232868	3_3	Horses
8	0,7974039	0,000214212	0,0402345	0,000326269	0,000022198	0,01091158	0,000077176	0,000534644	-0,04977785	3_4	Horses
8	0,5126851	0,07602739	0,01648543	0,000042754	0,01702808	0,008626594	0,003603519	0,004019089	0,05138291	4_1	Pigs
8	0,3743018	0,3419207	0,000059981	0,000013385	0,00490784	0,00137456	0,000000003	0,01864157	0,3405173	4_2	Pigs
8	0,5217096	0,1281285	0,02788757	0	0,002295756	0,01444587	0,000910994	0,005278337	0,09582094	5_1	Sheep
8	0,3951754	0,2350944	0,01570759	0,01165783	0,00483377	0,01812848	0,005530972	0,003097862	0,2105161	5_2	Sheep
**Participant 9**											
**Participant_ID**	**Neutral_Mean**	**Happy_Mean**	**Sad_Mean**	**Angry_Mean**	**Surprised_Mean**	**Scared_Mean**	**Disgusted_Mean**	**Contempt_Mean**	**Valence_Mean**	**Animal_Id**	**Animal**
9	0,3565933	0,4050825	0	0,004996202	0,000236458	0,003035878	0	0,00063473	0,397513	1_1	Dogs
9	0,3227297	0,3353681	0,007902307	0,000259481	0,04251216	0	0,00019952	0,00426568	0,3274428	1_2	Dogs
9	0,4845402	0,03631086	0,003616771	0,000426576	0,00000149	0,01620441	0,000000013	0,003748696	0,01675358	1_3	Dogs
9	0,5321212	0,005114231	0,004056876	0,000002937	0,03007512	0,009243873	0,001737571	0,002654592	-0,008186481	1_4	Dogs
9	0,3180919	0,3675908	0,0030758	0,000632089	0,06942844	0,004550754	0,00072892	0,000511155	0,3599642	1_5	Dogs
9	0,4863006	0,0323547	0,03196368	0,000027474	0,008798865	0,002391003	0,01273015	0,006700751	-0,001483267	1_6	Dogs
9	0,3216945	0,3972374	0,002574495	0,000455775	0,000309841	0,001433055	0,003461413	0,004237147	0,391527	1_7	Dogs
9	0,4773625	0,08010273	0,003035912	0,000598822	0,000002952	0,00875566	0,000189231	0,003281345	0,06913547	1_8	Dogs
9	0,4786673	0	0,0484497	0,003504092	0,000858506	0,01302482	0,009587768	0,03763623	-0,06039209	1_9	Dogs
9	0,4974197	0,000046684	0,00150754	0,000300766	0,000147645	0,01963851	0,000013743	0,003462737	-0,02027389	1_10	Dogs
9	0,5892212	0,005139038	0,0228001	0,004610915	0,01725409	0,06222301	0,008730969	0,01160411	-0,07594065	1_11	Dogs
9	0,5178866	0,002878474	0,000594173	0,000119111	0,004229199	0,002764192	0	0,000208238	-0,000510685	1_12	Dogs
9	0,3712922	0,001057149	0,3415993	0,00535228	0,000038958	0,003910187	0,04693854	0,001240635	-0,3444523	1_13	Dogs

9	0,5964151	0,00066449	0,1073447	0,2262339	0,002005917	0,002561392	0,02016456	0,002463515	-0,3050509	2_1	Cats
9	0,3159453	0	0,4610896	0,1378264	0,000027359	0,000711644	0,003304919	0,003681241	-0,5533034	2_2	Cats
9	0,4598739	0	0,380313	0,09119505	0,000005769	0,000126008	0,000010865	0,00076563	-0,4675032	2_3	Cats
9	0,4664809	0,000005066	0,2831741	0,1516107	0,000569065	0,002237995	0,003886606	0,001533543	-0,4178066	2_4	Cats
9	0,5225692	0,000022512	0,2924013	0,08465709	0,000606019	0,002684847	0,000265043	0,000539001	-0,3521333	2_5	Cats
9	0,4496935	0,00017625	0,3903534	0,01797461	0,001471052	0,003449651	0,009469407	0,00129655	-0,3976544	2_6	Cats
9	0,6003418	0	0,1392288	0,0868215	0,000051242	0,000000025	0	0,000124006	-0,2092696	2_7	Cats
9	0,5730397	0,000006437	0,2601092	0,08489138	0,000727022	0,000646608	0,000131104	0,000802442	-0,3292405	2_8	Cats
9	0,5379652	0,01180437	0,02382009	0,005028236	0,000293476	0,01516327	0,00074801	0,003987163	-0,02387276	3_1	Horses
9	0,8373411	0,0200533	0,05136983	0,000847873	0,01289512	0,01148139	0,006946106	0,001541604	-0,03695073	3_2	Horses
9	0,7378226	0,001354955	0,1150745	0,002589279	0,000018116	0,04289086	0,004759726	0,01113002	-0,1264541	3_3	Horses
9	0,5378377	0,001476705	0,01371414	0,00534533	0,001478389	0,00637319	0,000927814	0,004153282	-0,0219053	3_4	Horses
9	0,8279406	0,06568001	0,01477232	0,005685364	0,002708595	0,008488772	0,001669471	0,001054795	0,04206762	4_1	Pigs
9	0,9418218	0	0,001319026	0,003823196	0,000109697	0,007498096	0,001540188	0,001101298	-0,01026804	4_2	Pigs
9	0,5478441	0,006731531	0,009434612	0,001146916	0,01814912	0,01077492	0,008844564	0,003240868	-0,01632419	5_1	Sheep
9	0,6387722	0	0,005912019	0	0,001770769	0,001064642	0	0,003622939	-0,006861202	5_2	Sheep
**Participant 10**											
**Participant_ID**	**Neutral_Mean**	**Happy_Mean**	**Sad_Mean**	**Angry_Mean**	**Surprised_Mean**	**Scared_Mean**	**Disgusted_Mean**	**Contempt_Mean**	**Valence_Mean**	**Animal_Id**	**Animal**
10	0,4645511	0,3482352	0,000973594	0,000000096	0,000592357	0,01079222	0	0,007584581	0,336882	1_1	Dogs
10	0,5949453	0,1020143	0,01151465	0,002205786	0,0030157	0,01282022	0,008792032	0,02032369	0,0775187	1_2	Dogs
10	0,7663321	0,009495463	0,01264523	0,01476932	0,003123175	0,01186474	0,01107232	0,008038882	-0,02491907	1_3	Dogs
10	0,9111435	0,000000001	0,01645584	0,002993649	0,000277614	0,005798835	0,01437023	0,00318188	-0,03122417	1_4	Dogs
10	0,8831737	0,01073712	0,003505037	0,002174994	0,001580879	0,007591246	0,02286664	0,01655107	-0,02265014	1_5	Dogs
10	0,8707624	0,01092171	0,01394253	0,001415337	0,000627709	0,00847566	0,01096796	0,02420091	-0,01214988	1_6	Dogs
10	0,8982053	0,001708618	0,01811244	0,002130829	0,000706388	0,006972016	0,01474525	0,008033995	-0,02445169	1_7	Dogs
10	0,9202887	0,000007317	0,01060063	0,002368772	0,000430062	0,00327825	0,01078596	0,006292637	-0,01959	1_8	Dogs
10	0,899954	0	0,02220988	0,005992258	0,004735001	0,004005665	0,01100363	0,000265693	-0,02980741	1_9	Dogs
10	0,880129	0	0,02039409	0,003704482	0,000799932	0,004280169	0,01042975	0,0029067	-0,02466594	1_10	Dogs
10	0,8966616	0,01740446	0,00483429	0,001940415	0,000518315	0,005722019	0,003732954	0,01139664	0,006175209	1_11	Dogs

10	0,8401718	0,02705012	0,003803501	0,004647333	0,004308364	0,02355972	0,002863314	0,02119193	-0,004442156	1_12	Dogs
10	0,7648988	0,000580029	0,03476303	0,002303653	0,0163704	0,000170804	0,005981387	0,0583579	-0,03421643	1_13	Dogs
10	0,5377804	0,1177485	0,07975248	0,002867665	0,001155803	0,006918178	0,01474775	0,006257659	0,03244613	2_1	Cats
10	0,7151869	0,000002296	0,1479539	0,007069082	0,000005393	0,009702832	0,01679211	0,000840132	-0,1555036	2_2	Cats
10	0,6743566	0	0,1560076	0,0008807	0,000021797	0,004139994	0,008745927	0,000023435	-0,1584434	2_3	Cats
10	0,7058489	0,000807338	0,1184143	0,004340943	0,000813589	0,03019481	0,003520695	0,001198335	-0,1330339	2_4	Cats
10	0,6367913	0,03493316	0,1365904	0,001214045	0,002020642	0,03726731	0,002312596	0,004941599	-0,1080972	2_5	Cats
10	0,6018218	0,1722966	0,008483028	0,000002472	0,000003011	0,002164513	0,000000753	0,004337007	0,162067	2_6	Cats
10	0,5891535	0,000000007	0,1082673	0,004004038	0,00749821	0,01260611	0,02597661	0,00300548	-0,1208903	2_7	Cats
10	0,6361155	0,01821142	0,1108258	0,005690922	0,000019014	0,009552605	0,004173861	0,00731631	-0,09947389	2_8	Cats
10	0,8911375	0,00458559	0,02150239	0,001973033	0,001201494	0,00729872	0,001620995	0,001743854	-0,02092138	3_1	Horses
10	0,7282686	0,002197647	0,0336591	0,000727986	0,003460024	0,03218918	0,001132286	0,03375719	-0,05836881	3_2	Horses
10	0,7594216	0,000091054	0,1241457	0,001934817	0,002255433	0,004791515	0,001454321	0,001647068	-0,1276364	3_3	Horses
10	0,9356429	0,000192028	0,0232836	0,000091584	0,000000005	0,005800714	0,000247273	0,000023547	-0,02489851	3_4	Horses
10	0,6092201	0,05722251	0,02146268	0,001336153	0,009393499	0,01951755	0,001235757	0,03715166	0,02288397	4_1	Pigs
10	0,7738883	0,000352934	0,05031512	0,00249833	0,002192014	0,01554822	0,000077839	0,006018343	-0,05736818	4_2	Pigs
10	0,895331	0,02113995	0,01834901	0,00140732	0,00744059	0,001440262	0,002317414	0,002125889	0,000589669	5_1	Sheep
10	0,8963683	0,005975899	0,0096847	0,01205463	0,000554921	0,004813369	0,000488115	0,001583225	-0,01454451	5_2	Sheep
**Participant 11**											
**Participant_ID**	**Neutral_Mean**	**Happy_Mean**	**Sad_Mean**	**Angry_Mean**	**Surprised_Mean**	**Scared_Mean**	**Disgusted_Mean**	**Contempt_Mean**	**Valence_Mean**	**Animal_Id**	**Animal**
11	0,7520943	0,003527262	0,01066715	0,01186912	0,01486302	0,02963212	0,003040269	0,01285771	-0,03839079	1_1	Dogs
11	0,5891905	0,1534706	0,005920264	0,001615943	0,0368551	0,01608679	0,000824796	0,0231038	0,1339567	1_2	Dogs
11	0,7985094	0,000000086	0,03763815	0,00030645	0,000016675	0,04235803	0,000123661	0,01275765	-0,06139625	1_3	Dogs
11	0,6540452	0,07706136	0,006378814	0,000557707	0,01165562	0,03372017	0,000584784	0,04737801	0,03866177	1_4	Dogs
11	0,8307977	0	0,01391287	0,002800761	0,004498776	0,01369321	0,001352767	0,006951701	-0,02699235	1_5	Dogs
11	0,7583131	0,000165095	0,03759042	0,007366145	0,01168313	0,01686933	0,008249347	0,01113264	-0,05342365	1_6	Dogs
11	0,7730185	0,003248272	0,05886449	0,02864492	0,002660432	0,0196505	0,02991001	0,002059535	-0,0740177	1_7	Dogs
11	0,8008814	0,000019906	0,05249821	0,01381447	0,002992971	0,8008814	0,000019906	0,05249821	0,01381447	1_8	Dogs
11	0,814886	0	0,07212984	0,03423607	0,001387762	0,01219492	0,02191301	0,000835438	-0,08292378	1_9	Dogs

11	0,7503023	0,007265021	0,05419658	0,01178169	0,01309305	0,02063401	0,02531549	0,003369412	-0,06515867	1_10	Dogs
11	0,7177759	0,009150903	0,06890262	0,02393469	0,007945198	0,0511374	0,02514755	0,006086268	-0,107972	1_11	Dogs
11	0,5293036	0,1201062	0,05880827	0,01957092	0,008258759	0,01753443	0,0285802	0,02451232	0,03771264	1_12	Dogs
11	0,5641473	0,1494642	0,001911384	0,000001499	0,005962686	0,02053013	0,000013382	0,04129729	0,1283724	1_13	Dogs
11	0,4479175	0,2997269	0,000698339	0,001096963	0,009815605	0,002312389	0,000504399	0,003598067	0,2961157	2_1	Cats
11	0,3482138	0,2821962	0,05180796	0,000168917	0,000018406	0,01439348	0,02271823	0,002837941	0,2167313	2_2	Cats
11	0,5606028	0	0,02516159	0,000929994	0,001687904	0,01039481	0,002209192	0,007121133	-0,03531896	2_3	Cats
11	0,5875572	0	0,007355414	0,000564314	0,001858779	0,01293689	0,001326978	0,003331222	-0,02040373	2_4	Cats
11	0,51592	0,004397805	0,01709536	0,003747384	0,0107047	0,01327201	0,003451425	0,001173019	-0,02006619	2_5	Cats
11	0,5322884	0,008015288	0,0213715	0,001082794	0,001555798	0,007195416	0,003610244	0,001424283	-0,02186515	2_6	Cats
11	0,5315039	0,001993255	0,03306273	0,000123799	0,000280811	0,01791516	0,009729542	0,005957523	-0,04866133	2_7	Cats
11	0,5294285	0	0,01108238	0,001725015	0	0,02100144	0,000146937	0,004175384	-0,03152998	2_8	Cats
11	0,8174526	0,05524734	0,009625233	0,000626245	0,01074997	0,01520746	0,000681935	0,02397013	0,03172717	3_1	Horses
11	0,7361839	0,000158715	0,1224613	0,008520385	0,003181296	0,009731938	0,000640129	0,003621102	-0,1307844	3_2	Horses
11	0,7572508	0,000971676	0,07401167	0,0108584	0,008448437	0,01761112	0,02075723	0,002260715	-0,1127468	3_3	Horses
11	0,771415	0,000905378	0,06278059	0,000022325	0,000644946	0,04973902	0,000136836	0,01226637	-0,07910177	3_4	Horses
11	0,8154962	0,000057769	0,08009931	0,007579038	0,004279398	0,002041079	0,000284103	0,00292589	-0,08248959	4_1	Pigs
11	0,7448707	0,000032806	0,1480339	0,003584899	0,00069063	0,01073841	0,00448006	0,000137947	-0,1494474	4_2	Pigs
11	0,7932173	0,004301593	0,01660227	0,000593503	0,003191883	0,02336808	0,000172882	0,04084435	-0,03300615	5_1	Sheep
11	0,6536569	0,02621941	0,01040568	0,000063023	0,004980978	0,02321477	0,0002076	0,04223562	-0,000463052	5_2	Sheep
**Participant 12**											
**Participant_ID**	**Neutral_Mean**	**Happy_Mean**	**Sad_Mean**	**Angry_Mean**	**Surprised_Mean**	**Scared_Mean**	**Disgusted_Mean**	**Contempt_Mean**	**Valence_Mean**	**Animal_Id**	**Animal**
12	0,8237468	0,000032707	0,07364549	0,002558268	0,00560166	0,008961409	0	0,002026351	-0,08358132	1_1	Dogs
12	0,7947896	0,000224286	0,06876845	0,01271289	0,000234167	0,008857518	0	0,006465351	-0,08490163	1_2	Dogs
12	0,7234649	0,00051245	0,152997	0,005347261	0,006335001	0,000807111	0,00060578	0,005888628	-0,1554795	1_3	Dogs
12	0,7339794	0,000019096	0,1215486	0,003255959	0,002453647	0,003140111	0	0,00128929	-0,1241772	1_4	Dogs
12	0,8933617	0,000035765	0,01236004	0,001871924	0,001525703	0,02834131	0,000392889	0,002132806	-0,03919457	1_5	Dogs
12	0,8047926	0,000156633	0,05912506	0,00679189	0,000537417	0,0106675	0,000067773	0,004715445	-0,0700056	1_6	Dogs
12	0,6450184	0,000131389	0,1539485	0,001438254	0,001098173	0,002716157	0,00002353	0,002699693	-0,1560686	1_7	Dogs

12	0,898794	0,000012637	0,02342953	0,003427632	0,009124327	0,004032918	0,000000006	0,000721911	-0,02925965	1_8	Dogs
12	0,8315953	0	0	0,03056824	0,000005146	0,000004302	0	0,000003356	-0,03056824	1_9	Dogs
12	0,6256235	0,000653034	0,221686	0,000749353	0,001768852	0,001302892	0,001635947	0,003676325	-0,2212356	1_10	Dogs
12	0,4418392	0,000002207	0,4657466	0,000191493	0,000243842	0,000842636	0,00020554	0,002802599	-0,4657443	1_11	Dogs
12	0,7158344	0,000051633	0,1624854	0,002545511	0,00114681	0,002999307	0,001591543	0,00054989	-0,1647146	1_12	Dogs
12	0,8565039	0,000167134	0,02788089	0,00111031	0,001400747	0,009089237	0,000109424	0,004077391	-0,03507895	1_13	Dogs
12	0,8705139	0,004855882	0,0517828	0,006301998	0,002436433	0,003790074	0,007964844	0,003217898	-0,04941686	2_1	Cats
12	0,9330363	0,000378309	0,03540554	0,003689084	0,001443578	0,006681032	0,002617657	0,003320127	-0,03783597	2_2	Cats
12	0,9214427	0,001775862	0,009732317	0,000661087	0,004829217	0,003859587	0,001211391	0,000840072	-0,01065106	2_3	Cats
12	0,9073019	0,001171648	0,04666545	0,001183144	0,001642444	0,008502995	0,003028652	0,000362667	-0,05442715	2_4	Cats
12	0,87904	0,005305528	0,01455567	0,01683247	0,000729122	0,002004491	0,003276183	0,002242377	-0,02269458	2_5	Cats
12	0,90564	0,002801801	0,03207692	0,005496301	0,007246553	0,005140775	0,001523984	0,000986534	-0,03552252	2_6	Cats
12	0,8651378	0,000000006	0,02499114	0,008404402	0,000000001	0,005640151	0,00060105	0,000119012	-0,0306037	2_7	Cats
12	0,8827859	0,008086143	0,03081336	0,006986352	0,000415507	0,003771611	0,007872139	0,001274957	-0,03394203	2_8	Cats
12	0,6817679	0,000264723	0,1894715	0,003303756	0,000978097	0,002967529	0,000676244	0,003422294	-0,1912574	3_1	Horses
12	0,8303246	0,000981257	0,03413875	0,008980724	0,03262491	0,0153568	0,000557115	0,00077265	-0,05180298	3_2	Horses
12	0,8619937	0,000154564	0,0650792	0,004741498	0,003455776	0,00605874	0,001132269	0,004374024	-0,07094576	3_3	Horses
12	0,7606108	0,000000401	0,1271435	0,001876425	0,001579386	0,000938763	0,000279103	0,001745801	-0,1285114	3_4	Horses
12	0,7692317	0,001206554	0,04298059	0,01809105	0,01128858	0,007976945	0,003880355	0,00324	-0,0578918	4_1	Pigs
12	0,7363172	0,003602606	0,05152872	0,05242335	0,02009834	0,00453072	0,000111396	0,01636817	-0,09735844	4_2	Pigs
12	0,7524076	0,000155348	0,0713181	0,007378018	0,002173702	0,00504736	0,000381768	0,002214572	-0,07480334	5_1	Sheep
12	0,7661375	0,000084876	0,112756	0,001268698	0,000443552	0,004692584	0,000980114	0,000643736	-0,1126711	5_2	Sheep
**Participant 13**											
**Participant_ID**	**Neutral_Mean**	**Happy_Mean**	**Sad_Mean**	**Angry_Mean**	**Surprised_Mean**	**Scared_Mean**	**Disgusted_Mean**	**Contempt_Mean**	**Valence_Mean**	**Animal_Id**	**Animal**
13	0,8714746	0,000518109	0,009811715	0,01272499	0,02149792	0,004000165	0,000029677	0,008906146	-0,02353411	1_1	Dogs
13	0,8599914	0,000588054	0,01201372	0,009880634	0,05152586	0,005666539	0,000156993	0,003682777	-0,02340517	1_2	Dogs
13	0,8628533	0,00097848	0,005598637	0,04282776	0,04663954	0,001478224	0,000080242	0,006825384	-0,043201	1_3	Dogs
13	0,9095387	0,000564343	0,02486256	0,004220059	0,02072287	0,002111312	0,000226896	0,004495	-0,02936083	1_4	Dogs
13	0,8263147	0,009796313	0,03531528	0,01791107	0,02402106	0,00634401	0,007063314	0,002006386	-0,04635294	1_5	Dogs

13	0,8513436	0,001974645	0,01366057	0,04655803	0,02040694	0,007934526	0,000553121	0,00050101	-0,06236165	1_6	Dogs
13	0,6549515	0,003821778	0,1721066	0,003409087	0,05841447	0,003311292	0,01268999	0,000033666	-0,1702697	1_7	Dogs
13	0,8382221	0,000160102	0,01770499	0,008505802	0,08778084	0,004534816	0,00002161	0,005014434	-0,02606367	1_8	Dogs
13	0,7867263	0,000436111	0,02016742	0,001137446	0,1524933	0,009890804	0,000322381	0,000749789	-0,02882723	1_9	Dogs
13	0,7328036	0,01326685	0,07356716	0,000248323	0,04904209	0,003424738	0,007815753	0,001788866	-0,0626549	1_10	Dogs
13	0,7660181	0,005019162	0,04034862	0,008639655	0,06331681	0,005681857	0,000235083	0,001679742	-0,04302476	1_11	Dogs
13	0,7908401	0,00000092	0,01680668	0,003730604	0,07923524	0,004427664	0,002687413	0,000051686	-0,02514183	1_12	Dogs
13	0,8555164	0	0,008453496	0,005929324	0,07977873	0,000092047	0,008980796	0,000685119	-0,02051669	1_13	Dogs
13	0,7708337	0,007663493	0,001108937	0	0,01460973	0,02107112	0,000000007	0,01358271	-0,01407948	2_1	Cats
13	0,7817359	0,006461134	0,0416734	0,004147942	0,005039406	0,03915596	0,00464917	0,0216812	-0,06611805	2_2	Cats
13	0,7702714	0,001599434	0,0286008	0,000144006	0,00099732	0,008161008	0,000322189	0,008688621	-0,03278343	2_3	Cats
13	0,7014878	0,000431633	0,02391622	0,000310003	0,000462095	0,03581728	0,000711863	0,01987325	-0,05116427	2_4	Cats
13	0,7635034	0,000763479	0,05224169	0,00175993	0,000306696	0,03587239	0,002299299	0,01489734	-0,07023095	2_5	Cats
13	0,5990365	0,0364404	0,02294348	0,01738797	0,002353473	0,02076588	0,01033091	0,03499881	-0,01315045	2_6	Cats
13	0,605885	0,04122451	0,01151924	0,01140326	0,000891758	0,02026438	0,01803441	0,03502815	-0,001189712	2_7	Cats
13	0,6585858	0,000195335	0,0639657	0,000000449	0,001463405	0,04233065	0,000000826	0,03769081	-0,0874038	2_8	Cats
13	0,8259639	0,000987112	0,01616339	0,009319629	0,04195289	0,003511004	0,000168138	0,008725321	-0,02364599	3_1	Horses
13	0,8178415	0,000433444	0,02185155	0,04965195	0,003474586	0,002715645	0,000224808	0,004558565	-0,06736325	3_2	Horses
13	0,4504719	0,00498596	0,07841687	0,3367712	0,005651425	0,000370174	0,002580696	0,001880528	-0,4037203	3_3	Horses
13	0,8472597	0,000000739	0,007631801	0,0261488	0,002219523	0,02091566	0,000042311	0,000176126	-0,04081446	3_4	Horses
13	0,8592329	0,000609352	0,04424797	0,0133701	0,005647919	0,003889981	0,00057913	0,002997972	-0,05326181	4_1	Pigs
13	0,8189777	0,000239957	0,0807928	0,0341046	0,005079483	0,001797247	0,000848071	0,002404535	-0,1100299	4_2	Pigs
13	0,7396643	0,005007027	0,09009718	0,01638986	0,0380289	0,01273888	0,004581702	0,000913476	-0,1081818	5_1	Sheep
13	0,6619654	0,003120498	0,001606853	0,05600264	0,1102297	0,01936552	0,000000015	0,002917365	-0,06575438	5_2	Sheep
**Participant 14**											
**Participant_ID**	**Neutral_Mean**	**Happy_Mean**	**Sad_Mean**	**Angry_Mean**	**Surprised_Mean**	**Scared_Mean**	**Disgusted_Mean**	**Contempt_Mean**	**Valence_Mean**	**Animal_Id**	**Animal**
14	0,6991747	0,003853827	0,02078211	0,0574884	0,009521451	0,007103262	0,001420301	0,01425054	-0,07523926	1_1	Dogs
14	0,7853221	0,000563445	0,04228581	0,02486737	0,01010182	0,002349379	0,002991818	0,001123487	-0,06496064	1_2	Dogs
14	0,6686823	0,001399626	0,1159222	0,0809141	0,003740347	0,000006716	0,01525535	0,000688325	-0,1927376	1_3	Dogs

14	0,7568623	0,000453964	0,05841505	0,04536087	0,0122179	0,00184425	0,000368529	0,00406694	-0,102077	1_4	Dogs
14	0,6137201	0,000046573	0,2032719	0,1006022	0,002901381	0,008188922	0,001509727	0,001097052	-0,2887181	1_5	Dogs
14	0,7448093	0,000820064	0,1078185	0,02315642	0,007044657	0,003833837	0,000312809	0,004532066	-0,1298136	1_6	Dogs
14	0,7991073	0,00018592	0,05545451	0,02046507	0,01713359	0,00618285	0,000360701	0,006989283	-0,07256506	1_7	Dogs
14	0,7809398	0,00003412	0,06183004	0,04335505	0,009821268	0,005803473	0,00024866	0,002884482	-0,09780005	1_8	Dogs
14	0,831126	0	0,04969036	0,01893692	0,003631856	0,001089966	0,000753941	0,003891817	-0,06237027	1_9	Dogs
14	0,7797995	0	0,08499329	0,05996563	0,004192531	0,003156197	0,000311036	0,008975733	-0,1385968	1_10	Dogs
14	0,7208362	0,000453992	0,07230817	0,1028221	0,006653146	0,001928353	0,000985418	0,003053892	-0,1668549	1_11	Dogs
14	0,9078969	0,000038488	0,04383338	0,00041454	0,009679189	0,000295814	0,000061382	0,003317567	-0,04414162	1_12	Dogs
14	0,8055921	0,000298623	0,05408019	0,02125503	0,006608387	0,000275398	0,00122989	0,002061833	-0,06678537	1_13	Dogs
14	0,8864096	0,000031222	0,0163412	0,004657193	0,003874074	0,02127061	0,000762093	0,01187882	-0,03798161	2_1	Cats
14	0,9582339	0	0,000188691	0,000003581	0,01958898	0,0103499	0,000023438	0,01131163	-0,0103499	2_2	Cats
14	0,7735376	0,001419735	0,02639273	0,002481131	0,005629581	0,005130622	0,000770821	0,02380564	-0,02883761	2_3	Cats
14	0,7174806	0,002930796	0,1684385	0,002067073	0,009127782	0,008515499	0,001976318	0,009403217	-0,1700923	2_4	Cats
14	0,8705185	0	0,03001572	0,004698625	0,001290749	0,004978162	0,000117202	0,003866299	-0,03489929	2_5	Cats
14	0,6865554	0	0,1971429	0,005002025	0,004496974	0,001888912	0,00307449	0,000650237	-0,2001721	2_6	Cats
14	0,6522816	0	0,1910568	0,009070233	0,003650012	0,001822828	0,002188162	0,007092715	-0,1956777	2_7	Cats
14	0,7274729	0	0,1214058	0,001010715	0,01524302	0,003893801	0,002647205	0,002125524	-0,1220209	2_8	Cats
14	0,7610019	0,000206763	0,07558936	0,05145972	0,008019362	0,006333827	0,000716487	0,002888223	-0,1161537	3_1	Horses
14	0,5417284	0,008018981	0,03669417	0,1309513	0,006010261	0,02272348	0,000772623	0,02291824	-0,1508863	3_2	Horses
14	0,893284	0,005067855	0,007335306	0,03135845	0,006606027	0,006290595	0,000599181	0,000684881	-0,03613907	3_3	Horses
14	0,8161469	0,000175074	0,04589248	0,03747127	0,001814366	0,000764931	0,001095326	0,01327073	-0,07458784	3_4	Horses
14	0,635669	0,005390031	0,01862116	0,1052531	0,000292009	0,00833122	0,000245568	0,001028766	-0,1095875	4_1	Pigs
14	0,5388489	0	0,09991428	0,117782	0,000235314	0,0113281	0,000702505	0,005955011	-0,210168	4_2	Pigs
14	0,8477051	0,000706918	0,03262231	0,03638572	0,003035921	0,000935218	0,000525562	0,005242715	-0,06626806	5_1	Sheep
14	0,7851753	0,000092692	0,07960679	0,03766784	0,006294764	0,01770961	0,003553297	0,007266481	-0,1012254	5_2	Sheep
**Participant 15**											
**Participant_ID**	**Neutral_Mean**	**Happy_Mean**	**Sad_Mean**	**Angry_Mean**	**Surprised_Mean**	**Scared_Mean**	**Disgusted_Mean**	**Contempt_Mean**	**Valence_Mean**	**Animal_Id**	**Animal**
15	0,6754861	0,000005158	0,04148337	0,02543762	0,00017227	0,01870601	0,003853172	0,000000314	-0,07205988	1_1	Dogs

15	0,6376981	0,05937218	0,05985293	0,01192377	0,02015919	0,002545775	0,001591893	0,07779501	-0,01026788	1_2	Dogs
15	0,6456579	0,003383395	0,007938312	0,149116	0,000144738	0,03359285	0,001052649	0,000004295	-0,160937	1_3	Dogs
15	0,6085405	0	0,008688625	0,1141446	0,03562302	0,006066735	0,000265507	0,02027178	-0,1190498	1_4	Dogs
15	0,5861588	0	0,006451454	0,1125355	0,004166965	0,001947759	0,001645146	0,01585968	-0,1167289	1_5	Dogs
15	0,5878023	0	0,06150599	0,0868815	0,000015815	0,02176974	0,000579401	0,01496817	-0,1394855	1_6	Dogs
15	0,4232361	0,00019207	0,04127733	0,3265972	0	0,001806953	0,000858204	0,000358174	-0,3293354	1_7	Dogs
15	0,4859676	0,004283691	0,033955	0,1730218	0	0,009173243	0,00100145	0,000846108	-0,1866687	1_8	Dogs
15	0,5370997	0,001122329	0,05306841	0,1127952	0	0,02109629	0,002041168	0,007447616	-0,147266	1_9	Dogs
15	0,5431921	0	0,0214141	0,2183649	0,007505455	0,02469544	0,000116996	0,001242353	-0,2359566	1_10	Dogs
15	0,5280066	0,03025585	0,002380657	0,1142952	0,000783312	0,000610791	0,000135493	0,08465793	-0,08403939	1_11	Dogs
15	0,5792353	0,005905399	0,0632369	0,1132824	0	0,009341483	0,00026953	0,01589615	-0,1350819	1_12	Dogs
15	0,7085898	0,004932923	0,03132024	0,01490332	0	0,04625152	0,000079901	0,000488718	-0,05629463	1_13	Dogs
15	0,8348262	0,000017165	0,02147318	0,006845986	0,07508449	0,003298412	0,000832304	0,008218739	-0,02571961	2_1	Cats
15	0,8172576	0,002119864	0,05246885	0,004710361	0,04566687	0,0150347	0,005621044	0,003344002	-0,06332139	2_2	Cats
15	0,8685422	0,000061748	0,01079914	0,001680792	0,01808256	0,003222926	0,000045103	0,01107893	-0,01340559	2_3	Cats
15	0,8016203	0,000858116	0,02612148	0,02175444	0,05043228	0,007581334	0,000175845	0,003501855	-0,04484233	2_4	Cats
15	0,6640955	0,01584967	0,168456	0,01773169	0,07890628	0,002856306	0,003200363	0,003052343	-0,1688685	2_5	Cats
15	0,7303167	0,009321202	0,08493158	0,02589644	0,05598498	0,01168542	0,000621741	0,001371038	-0,1036258	2_6	Cats
15	0,7090209	0,00582132	0,04092195	0,01414198	0,1183496	0,01283341	0,001772775	0,008089353	-0,0589439	2_7	Cats
15	0,8107477	0,000000083	0,000000097	0,0423989	0,07226605	0,005808596	0,000000002	0,000549027	-0,04331915	2_8	Cats
15	0,6866685	0,000270477	0,04654748	0,030208	0,000144293	0,017488	0,000565316	0,000136434	-0,08386384	3_1	Horses
15	0,9042882	0,001793595	0,0224407	0,01756444	0,02369273	0,004858038	0,002081652	0,000716811	-0,03081686	3_2	Horses
15	0,6791194	0,001023489	0,0211755	0,1291261	0,009506272	0,007742377	0,003195575	0,00209087	-0,1483298	3_3	Horses
15	0,660186	0,003098198	0,03403081	0,01949629	0,002370065	0,02428807	0,002371324	0,000080435	-0,0555085	3_4	Horses
15	0,8790163	0,01532393	0,007536383	0,01384579	0,009573259	0,005675628	0,001690485	0,000277807	-0,008183069	4_1	Pigs
15	0,7221181	0,01173157	0,000007743	0,008346546	0,1270911	0,006846613	0,000102597	0,000799173	-0,002522416	4_2	Pigs
15	0,6293866	0,000005409	0,01783643	0,1773207	0,000161124	0,01232227	0,000547512	0,000334174	-0,1806968	5_1	Sheep
15	0,5549679	0	0,07590846	0,1098639	0,01334242	0,01329881	0,00010212	0,000894047	-0,1647931	5_2	Sheep
**Participant 16**											

Participant_ID	Neutral_Mean	Happy_Mean	Sad_Mean	Angry_Mean	Surprised_Mean	Scared_Mean	Disgusted_Mean	Contempt_Mean	Valence_Mean	Animal_Id	Animal
16	0,7014251	0,02709184	0,000169718	0,00842568	0,003480795	0,0573301	0,000006351	0,004718392	-0,03196526	1_1	Dogs
16	0,9071317	0,00027065	0,000923194	0,01260523	0,01722441	0,0413566	0,000107666	0,000754338	-0,04293014	1_2	Dogs
16	0,9426929	0,000380582	0,000660438	0,008254036	0,02540694	0,002478801	0	0,000006116	-0,009906521	1_3	Dogs
16	0,9437324	0,000001044	0,000077287	0,000299529	0,03660035	0,03108089	0	0,000336747	-0,03114939	1_4	Dogs
16	0,9456978	0	0,000861104	0,002421367	0,009634236	0,04859038	0,000412294	0,000308529	-0,04885318	1_5	Dogs
16	0,9549741	0	0,000020533	0,00296095	0,05493766	0,001242817	0	0,000069981	-0,003644102	1_6	Dogs
16	0,9527305	0,005901661	0	0,005144546	0,01765429	0,01073791	0,001214203	0,000337251	-0,009839199	1_7	Dogs
16	0,9591898	0,00019934	0,000187446	0,000272461	0,02728545	0,01776507	0,000022005	0,000446413	-0,01756572	1_8	Dogs
16	0,9286065	0	0,000153199	0,000682868	0,01032836	0,09216266	0,001799324	0,001020596	-0,09409711	1_9	Dogs
16	0,9655085	0	0,000026592	0,004280124	0,02579633	0,01615083	0,000002965	0,000692082	-0,01842777	1_10	Dogs
16	0,9808191	0	0,000000786	0,00574396	0,01228399	0,004796688	0	0,000296394	-0,007091887	1_11	Dogs
16	0,9631382	0	0,003540218	0,007269754	0,007534819	0,007284387	0	0,000032141	-0,01332464	1_12	Dogs
16	0,9598044	0	0,000987167	0,01211673	0,01114764	0,005034599	0	0,000153901	-0,01494036	1_13	Dogs
16	0,6644057	0,000624887	0,04814852	0,1549821	0,004955085	0,003177514	0,000742507	0,000556461	-0,1951059	2_1	Cats
16	0,7617629	0,000402707	0,04177354	0,08064382	0,00581242	0,002995566	0,000914595	0,003227378	-0,1170281	2_2	Cats
16	0,7783686	0,000008237	0,08823273	0,01183785	0,01771451	0,005269994	0,000629124	0,001537851	-0,09717707	2_3	Cats
16	0,8173816	0,000467382	0,03408861	0,01773765	0,007749814	0,01500354	0,00076993	0,006985336	-0,05623318	2_4	Cats
16	0,8440018	0,002039309	0,02796741	0,02738	0,009853124	0,01227548	0,002167794	0,002000595	-0,05231229	2_5	Cats
16	0,8476295	0,000134918	0,03656185	0,02263721	0,01291553	0,000829421	0,00037838	0,002906474	-0,05690612	2_6	Cats
16	0,8301031	0,000103632	0,06498749	0,01166625	0,005528578	0,002996783	0,000273094	0,003292421	-0,07306024	2_7	Cats
16	0,9231092	0,000398838	0,020817	0,001813663	0,00306944	0,000205745	0,000126387	0,0106673	-0,02223736	2_8	Cats
16	0,9016281	0,00027756	0,01370447	0,03293213	0,006791402	0,01137893	0,001646614	0,000698749	-0,04576201	3_1	Horses
16	0,8464682	0,001035532	0,005379648	0,03964297	0,004144358	0,003823306	0,002368868	0,000956616	-0,04375368	3_2	Horses
16	0,4606692	0,1361395	0,002377789	0,04531048	0,01565067	0,000177927	0,000772548	0,03648824	0,08933993	3_3	Horses
16	0,8195344	0,003922583	0,000000384	0,06737366	0,04438362	0,01842768	0,000035504	0,000358978	-0,07496232	3_4	Horses
16	0,7107975	0,001730193	0,02260447	0,03665995	0,004846918	0,01125723	0,000687698	0,000705357	-0,04901426	4_1	Pigs
16	0,8415877	0,000009221	0,01199349	0,04333163	0,003574449	0,01103626	0,000165444	0,001766363	-0,05120934	4_2	Pigs
16	0,9254516	0,000795059	0,008824947	0,01544156	0,006085198	0,002913938	0,000575841	0,000948638	-0,02428106	5_1	Sheep
16	0,8879567	0,001261769	0,001055871	0,01550678	0,001991205	0,006839897	0,001095962	0,00088452	-0,01908218	5_2	Sheep

Participant 17											
**Participant_ID**	**Neutral_Mean**	**Happy_Mean**	**Sad_Mean**	**Angry_Mean**	**Surprised_Mean**	**Scared_Mean**	**Disgusted_Mean**	**Contempt_Mean**	**Valence_Mean**	**Animal_Id**	**Animal**
17	0,6471031	0,008645846	0,007759492	0,08532826	0,03369283	0,01916982	0,001406624	0,000826565	-0,08912184	1_1	Dogs
17	0,5790902	0,005429024	0,009253737	0,04929478	0,005558346	0,01188982	0,004768479	0,000393993	-0,05513512	1_2	Dogs
17	0,6031482	0,000000276	0,01222162	0,06236631	0,02897155	0,018596	0,002342007	0,004975236	-0,07771637	1_3	Dogs
17	0,5853924	0	0,01776437	0,08104654	0,01413031	0,0212449	0,04799021	0,004156023	-0,1467366	1_4	Dogs
17	0,634851	0	0,0238109	0,05641411	0,02965029	0,01194742	0,00023444	0,000546045	-0,08371247	1_5	Dogs
17	0,8343617	0	0,02626662	0,003240497	0,03420109	0,004545714	0,000000001	0,000182115	-0,03364567	1_6	Dogs
17	0,787623	0	0,0408064	0,02289021	0,01757301	0,006924457	0,000501272	0,000026254	-0,06022067	1_7	Dogs
17	0,8464183	0	0,008772507	0,009026392	0,01241464	0,002439607	0,000000017	0,000456977	-0,01854064	1_8	Dogs
17	0,7732146	0	0,02369144	0,03789983	0,02778963	0,006873877	0,000606684	0,000106696	-0,05705176	1_9	Dogs
17	0,8118424	0	0,01183745	0,009369674	0,02072919	0,007321392	0,000135468	0,000548295	-0,0247956	1_10	Dogs
17	0,7123082	0	0,01597022	0,02110215	0,02332332	0,01297106	0	0	-0,03812534	1_11	Dogs
17	0,7399744	0,000592543	0,009483363	0,02931191	0,03758994	0,01875978	0,002785277	0,000061399	-0,05065881	1_12	Dogs
17	0,874885	0,000000259	0,02944758	0,000536525	0,01348376	0,005875216	0,000036695	0,000118171	-0,0349091	1_13	Dogs
17	0,5835643	0,01249422	0,01341566	0,03013525	0,001162487	0,03188833	0,000195575	0,008045579	-0,04411961	2_1	Cats
17	0,5298954	0,02805202	0,05800336	0,05463518	0,000740418	0,0105132	0,000553849	0,010197	-0,07547602	2_2	Cats
17	0,5545917	0,000061503	0,01072223	0,1357235	0,002386993	0,006969161	0,000072658	0,005562208	-0,1462526	2_3	Cats
17	0,7318152	0,000147868	0,01525315	0,03983614	0,000869434	0,01239192	0,000111947	0	-0,05992761	2_4	Cats
17	0,6390362	0,000802666	0,007644576	0,05828894	0,000720212	0,00169925	0,000212208	0,006526024	-0,06285989	2_5	Cats
17	0,5262655	0,00030391	0,07341696	0,09984113	0,001087157	0,00183508	0,000535126	0,002132164	-0,1270073	2_6	Cats
17	0,5466301	0,01459769	0,04622122	0,0587956	0,001066331	0,01235385	0,00022503	0,02575648	-0,08103254	2_7	Cats
17	0,6334385	0,000929278	0,04032793	0,03998052	0,002249069	0,02030915	0,000084521	0,02054062	-0,09049532	2_8	Cats
17	0,7581545	0,001159739	0,007455499	0,01260034	0,01226715	0,02087777	0,005274056	0,001004438	-0,03792642	3_1	Horses
17	0,4850462	0,1128554	0,008229819	0,01770689	0,004220865	0,000223333	0,002012905	0,009746145	0,08898123	3_2	Horses
17	0,589882	0,004746506	0,02528058	0,01308459	0,005592262	0,06652318	0,001173419	0,001403636	-0,07977969	3_3	Horses
17	0,7567627	0,00053371	0,01691953	0,1195817	0,00234457	0,008533484	0,000210064	0,000787606	-0,1328683	3_4	Horses
17	0,5193202	0,1974715	0,006762715	0,008597164	0,008297286	0,000185536	0,000278025	0,002534225	0,1840099	4_1	Pigs
17	0,4488599	0,2393197	0,000407826	0,01210866	0,005405427	0,000250998	0,000245884	0,01163881	0,2270166	4_2	Pigs
17	0,7388416	0,000920725	0,02456242	0,1012852	0,002890149	0,01135477	0,002366537	0,001988948	-0,1134249	5_1	Sheep

17	0,8369551	0,000670553	0,008481497	0,02697971	0,005048925	0,0229003	0,000222473	0,000515236	-0,04200706	5_2	Sheep
**Participant 18**											
**Participant_ID**	**Neutral_Mean**	**Happy_Mean**	**Sad_Mean**	**Angry_Mean**	**Surprised_Mean**	**Scared_Mean**	**Disgusted_Mean**	**Contempt_Mean**	**Valence_Mean**	**Animal_Id**	**Animal**
18	0,375843	0,4028452	0,001792376	0,000029535	0,006581889	0,000004412	0,000501576	0,01209369	0,4010238	1_1	Dogs
18	0,6103739	0,04102434	0,05412788	0,001057303	0,004036088	0,03530553	0,01657734	0,008769198	-0,02379177	1_2	Dogs
18	0,4422057	0,09475604	0,1568342	0,01411486	0,009481758	0,01692533	0,0134984	0,006620438	-0,07158601	1_3	Dogs
18	0,6112754	0,1213456	0,01445735	0,02378714	0,001909906	0,003948712	0,001475792	0,005509918	0,08362088	1_4	Dogs
18	0,5528525	0,1301046	0,01723651	0,005774555	0,01177361	0,000040911	0,008070188	0,004818286	0,1073793	1_5	Dogs
18	0,5397165	0,1043057	0,09051297	0,005834567	0,004525387	0,002803461	0,01699238	0,0169636	0,008756927	1_6	Dogs
18	0,4772247	0,1590578	0,1433156	0,02555414	0,01481486	0,01109982	0,02809398	0,009544011	-0,008013413	1_7	Dogs
18	0,5311638	0,1185072	0,03535128	0,02610319	0,00959386	0,01746038	0,01793928	0,008761581	0,05793868	1_8	Dogs
18	0,6122465	0,000092258	0,08691117	0,02639928	0,00144756	0,02714403	0,07635791	0,00416488	-0,1192931	1_9	Dogs
18	0,4982704	0,07753531	0,05223284	0,03917567	0,000722805	0,02757487	0,01867162	0,003338913	-0,02638241	1_10	Dogs
18	0,4698028	0,1779412	0,002937141	0,01475313	0,008242346	0,01584744	0,005143569	0,002646607	0,1470694	1_11	Dogs
18	0,514286	0,100028	0,000014235	0,08961152	0,007673347	0,000046767	0,000077255	0,002886779	0,01036268	1_12	Dogs
18	0,6439494	0,1031241	0,0003782	0,07115096	0,0205128	0,00010074	0,000463791	0,008616792	0,031512	1_13	Dogs
18	0,9067274	0,000813054	0,000082119	0,03514818	0,002687478	0,006251157	0,000480513	0,000394065	-0,03834633	2_1	Cats
18	0,9373125	0,000143449	0,00176441	0,01062089	0,01306624	0,01130446	0,000723848	0,000190228	-0,0186085	2_2	Cats
18	0,9574972	0,000044262	0,002749141	0,000067817	0,00331533	0,002590166	0,000425105	0,000604026	-0,005613936	2_3	Cats
18	0,9617554	0	0,000197944	0,004294838	0,007497659	0,01912377	0,000135017	0,000648534	-0,02270711	2_4	Cats
18	0,9660162	0,000013826	0,000121922	0,002371005	0,02561555	0,007208704	0,000309538	0,000353896	-0,009308611	2_5	Cats
18	0,9319618	0,000170464	0,000738393	0,0162763	0,03140436	0,002220075	0,000416549	0,000374947	-0,01840165	2_6	Cats
18	0,9360797	0,000000027	0,000576799	0,001946429	0,06760769	0,01407805	0,000204312	0,000468255	-0,01575542	2_7	Cats
18	0,9482391	0	0,000875286	0,001536197	0,02138845	0,004149989	0,000091872	0,000078256	-0,005968885	2_8	Cats
18	0,4170316	0,005796231	0,02257526	0,2272574	0,00942535	0,00053429	0,000359617	0,04005543	-0,2214612	3_1	Horses
18	0,6097084	0,03512446	0,02935744	0,01940664	0,01594619	0,00503424	0,00178383	0,04635759	-0,01517492	3_2	Horses
18	0,6698866	0,01896334	0,1088977	0,02760114	0,007769221	0,006956621	0,01244825	0,08961126	-0,1157709	3_3	Horses
18	0,8308029	0	0,004314467	0,03631179	0,02228263	0,0003303	0,000317162	0,005395662	-0,03904847	3_4	Horses
18	0,6168585	0,01820447	0,01424809	0,03174704	0,005418742	0,0356274	0,001159588	0,009725394	-0,05327518	4_1	Pigs

18	0,6310705	0,03080118	0,002846724	0,01435373	0,000077146	0,01880661	0,000857731	0,008780413	0,002130591	4_2	Pigs
18	0,6788478	0,00152268	0,01191884	0,001461053	0,008061911	0,00005086	0,001045681	0,01313855	-0,0115909	5_1	Sheep
18	0,754362	0,05393724	0,000616187	0,03019378	0,004542708	0,000069363	0,000017251	0,003477307	0,02374294	5_2	Sheep
**Participant 19**											
**Participant_ID**	**Neutral_Mean**	**Happy_Mean**	**Sad_Mean**	**Angry_Mean**	**Surprised_Mean**	**Scared_Mean**	**Disgusted_Mean**	**Contempt_Mean**	**Valence_Mean**	**Animal_Id**	**Animal**
19	0,4725201	0,1270259	0,03067006	0,007926704	0,01747275	0,0306303	0,03436749	0,009968332	0,04189806	1_1	Dogs
19	0,4928372	0,06714517	0,02625712	0,01399298	0,003020783	0,02498676	0,000279575	0,01535542	0,01962946	1_2	Dogs
19	0,462373	0,125201	0,01523299	0,008481925	0,005831033	0,01768069	0,000582415	0,02663609	0,09037876	1_3	Dogs
19	0,5378467	0,03112269	0,01442309	0,007791789	0,02108735	0,005047824	0,000905829	0,04299161	0,01058399	1_4	Dogs
19	0,5083619	0,04367828	0,003411738	0,03215959	0,000877028	0,01235206	0,000333086	0,01055011	0,007894666	1_5	Dogs
19	0,5385014	0,02694817	0,01154581	0,01478262	0,00122571	0,02357309	0,001414416	0	-0,009062835	1_6	Dogs
19	0,5306656	0,03567262	0,02511517	0,03981025	0,01235086	0,01637822	0,001618531	0,01466367	-0,02934936	1_7	Dogs
19	0,508696	0,02364596	0,02228012	0,01661624	0,004445337	0,02815551	0,002096852	0,01186574	-0,01932488	1_8	Dogs
19	0,5048617	0,05709323	0,06540539	0,005391695	0,0024931	0,002271865	0,004114787	0,04563659	-0,0105086	1_9	Dogs
19	0,5389788	0,01543799	0,04401048	0,02644225	0,000524282	0,02484069	0,003523126	0,010562	-0,05359985	1_10	Dogs
19	0,568463	0,01842877	0,02494509	0,0213343	0,000109021	0,01103814	0,001891855	0,03517233	-0,02998165	1_11	Dogs
19	0,5316129	0,0060703	0,04004959	0,06170994	0,000134055	0,05392551	0,001761592	0,004448097	-0,09616034	1_12	Dogs
19	0,5745437	0,004535552	0,01282861	0,04534106	0,000488526	0,04659692	0,000247424	0,0005051	-0,06436065	1_13	Dogs
19	0,7977205	0,003059645	0,02092659	0,01235651	0,01316994	0,003765701	0,004030906	0,000582443	-0,02570563	2_1	Cats
19	0,767404	0,001596285	0,01471501	0,02545483	0,007058488	0,007634966	0,001514964	0,000874227	-0,0378109	2_2	Cats
19	0,7398428	0,000520497	0,04614318	0,02165102	0,02630781	0,01757403	0,000367212	0,001304531	-0,07377809	2_3	Cats
19	0,7719116	0,000687819	0,03977088	0,0130557	0,01742085	0,009217602	0,001776266	0,001307682	-0,05379624	2_4	Cats
19	0,8096904	0,000479975	0,00260229	0,01199637	0,0167489	0,006018891	0,000695292	0,000962041	-0,01798803	2_5	Cats
19	0,8284864	0,000413162	0,005939747	0,0126812	0,01103743	0,002694361	0,001611274	0,000605168	-0,0170942	2_6	Cats
19	0,7995092	0,003075154	0,007328287	0,08548085	0,00419424	0,006810284	0,002299808	0,001040968	-0,08454879	2_7	Cats
19	0,8095707	0,000104139	0,04519115	0,005574542	0,01598471	0,01227541	0,000745914	0,000362205	-0,0567822	2_8	Cats
19	0,6131887	0,005070554	0,01209322	0,01842496	0,02759623	0,05593359	0,001194134	0,03234703	-0,06128199	3_1	Horses
19	0,627718	0,06494541	0,04177452	0,01415758	0,000686567	0,01636586	0,01419938	0,002421828	-0,000497321	3_2	Horses
19	0,4852149	0,000004596	0,2241427	0,03172313	0	0,01865566	0,00003524	0,03251176	-0,2319841	3_3	Horses

19	0,5964614	0,002917882	0,03086635	0,009967376	0,000194775	0,04113663	0,000615995	0,007150568	-0,06696273	3_4	Horses
19	0,6489799	0,146909	0,01852834	0,007757639	0,005899517	0,002761047	0,02190651	0,002091234	0,1101841	4_1	Pigs
19	0,7943497	0,000142202	0,002215998	0,02913538	0,02964024	0,003947045	0,000001417	0,001673577	-0,03174745	4_2	Pigs
19	0,600484	0,02023509	0,01692867	0,06620842	0,000008573	0,03458501	0,005378898	0,000316161	-0,06663547	5_1	Sheep
19	0,6232818	0,04620355	0,02630451	0,0105044	0,004937647	0,04458416	0,004146059	0,02586785	-0,01625904	5_2	Sheep
**Participant 20**											
**Participant_ID**	**Neutral_Mean**	**Happy_Mean**	**Sad_Mean**	**Angry_Mean**	**Surprised_Mean**	**Scared_Mean**	**Disgusted_Mean**	**Contempt_Mean**	**Valence_Mean**	**Animal_Id**	**Animal**
20	0,5198922	0,2312355	0,02057447	0,0450342	0,001559612	0,000919118	0,01269274	0,01081349	0,1599156	1_1	Dogs
20	0,3436046	0,4084572	0,007534678	0,02175285	0,001079942	0,001781587	0,0485394	0,0222651	0,3396668	1_2	Dogs
20	0,4972196	0,2509035	0,01399869	0,0662665	0,000654282	0,002511139	0,009504967	0,03524588	0,174856	1_3	Dogs
20	0,712931	0,02224184	0,06715605	0,02588846	0,01223839	0,001539326	0,01154161	0,04104114	-0,0643099	1_4	Dogs
20	0,6528996	0,1386903	0,02997658	0,0218855	0,006269555	0,000804056	0,003276238	0,1068045	0,09025318	1_5	Dogs
20	0,7318243	0,000000789	0,08925516	0,03539402	0,001167895	0,002969153	0,003504632	0,05312101	-0,111593	1_6	Dogs
20	0,6487811	0,03143112	0,08244207	0,03131808	0,008242914	0,001614607	0,01076406	0,01315102	-0,07802455	1_7	Dogs
20	0,5012439	0,2476933	0,02905595	0,07432766	0,001086259	0,004513843	0,001933696	0,02008312	0,1477677	1_8	Dogs
20	0,6978908	0,00677502	0,01577374	0,001277505	0,02088125	0,006438288	0,006525943	0,003315033	-0,01385914	1_9	Dogs
20	0,7377893	0,006166678	0,02643486	0,002870085	0,006021756	0,00548005	0,004317203	0,01169586	-0,02702192	1_10	Dogs
20	0,4723884	0,2832565	0,02348511	0,008959591	0,01089287	0,000577259	0,000000732	0,02716924	0,2529501	1_11	Dogs
20	0,6195309	0,001269444	0,01045013	0,000000146	0,08804551	0,01539439	0,000485341	0,00657316	-0,02087436	1_12	Dogs
20	0,6501788	0,002207984	0,000098264	0,003647373	0,004353042	0,02125814	0	0,04877429	-0,02263877	1_13	Dogs
20	0,4927313	0,04107105	0,1584918	0,01344826	0,006086996	0,01560002	0,04444028	0,00930151	-0,1258116	2_1	Cats
20	0,7279641	0	0,003682152	0,08117861	0,00329865	0,001064679	0,001183853	0,01429825	-0,08438753	2_2	Cats
20	0,7806817	0,001265939	0,01221176	0,02548438	0,01609727	0,000644599	0,003598625	0,006132335	-0,0327896	2_3	Cats
20	0,8296406	0,00013527	0,01928808	0,0151914	0,01403783	0,00114331	0,008526352	0,002874525	-0,0327955	2_4	Cats
20	0,6806495	0,1005758	0,003432874	0,01706886	0,00570728	0,000989339	0,00068968	0,008803912	0,08122753	2_5	Cats
20	0,4395146	0,3201582	0,01257896	0,000061151	0,003508351	0,002304748	0,003307288	0,007051096	0,3074725	2_6	Cats
20	0,6662918	0,01458668	0,08749907	0,01849603	0,007451662	0,000621281	0,0189561	0,006377099	-0,08856212	2_7	Cats
20	0,8117499	0	0,000072009	0,02570397	0,01139012	0,000406511	0,000016282	0,00552464	-0,02584649	2_8	Cats
20	0,6586232	0,008490896	0,1074502	0,02111971	0,001205591	0,00283	0,04287172	0,007942843	-0,1414085	3_1	Horses

20	0,6397091	0,001608408	0,1081841	0,0181663	0,01395689	0,000476408	0,010223	0,001620712	-0,1150116	3_2	Horses
20	0,921658	0,000029223	0,03111351	0,001207343	0,000232456	0,001572127	0,000006737	0,01160514	-0,03120564	3_3	Horses
20	0,6935517	0,000150936	0,1129167	0,002588497	0,009036242	0,002013882	0,01261539	0	-0,1145518	3_4	Horses
20	0,5037065	0,000471385	0,2250335	0,01118993	0,01299074	0,000093646	0,005231842	0,002040332	-0,2291359	4_1	Pigs
20	0,62425	0,000624443	0,09960613	0,01912002	0,04107959	0,000003198	0,001048672	0,003519721	-0,1153223	4_2	Pigs
20	0,7866426	0,002332126	0,03274802	0,00386687	0,004205824	0,003975264	0,004920273	0,004351908	-0,03710993	5_1	Sheep
20	0,5389923	0,01552639	0,05606631	0,002398199	0,001444209	0,002973609	0,2425909	0,0008055	-0,2498913	5_2	Sheep
**Participant 21**											
**Participant_ID**	**Neutral_Mean**	**Happy_Mean**	**Sad_Mean**	**Angry_Mean**	**Surprised_Mean**	**Scared_Mean**	**Disgusted_Mean**	**Contempt_Mean**	**Valence_Mean**	**Animal_Id**	**Animal**
21	0,6414334	0,001505421	0,09188625	0,007237269	0,01654867	0,001716966	0,007092207	0,01264246	-0,09282844	1_1	Dogs
21	0,752352	0,000162697	0,005568886	0,02793639	0	0,0119623	0,000027143	0,0699767	-0,03684804	1_2	Dogs
21	0,7980854	0,000401343	0,03594694	0,002270245	0,001917226	0,000440692	0,004589691	0,000165079	-0,03780269	1_3	Dogs
21	0,6019063	0,000801947	0,04454298	0,04406695	0,000000037	0,02360848	0,000374443	0,01813228	-0,08321993	1_4	Dogs
21	0,5758024	0,000008503	0,06751678	0,01104982	0	0,02214908	0,000001171	0,01018555	-0,07624093	1_5	Dogs
21	0,7022524	0,000054593	0,08409981	0,000376294	0,00879211	0	0	0,00025147	-0,08408696	1_6	Dogs
21	0,4312496	0,000007122	0,2877618	0,000786284	0,002176661	0,000009789	0,000103414	0	-0,2877547	1_7	Dogs
21	0,08081216	0,003292989	0,8463666	0,000000001	0	0,03351823	0,06905901	0	-0,8430736	1_8	Dogs
21	0,4900939	0,00161078	0,2059865	0,01391412	0,00214552	0,004769219	0,004315853	0,01633024	-0,204415	1_9	Dogs
21	0,5194559	0	0,2141486	0,000431151	0,006349649	0	0	0,000000003	-0,2141486	1_10	Dogs
21	0,276368	0,000360956	0,5045604	0	0,006877528	0	0,001739522	0	-0,5041994	1_11	Dogs
21	0,4294624	0,00210527	0,2778811	0,000157568	0,03001482	0,000081636	0,007487727	0,000134112	-0,2757758	1_12	Dogs
21	0,4690717	0,000001251	0,2294518	0,00179824	0,006431988	0,000000065	0,000008456	0,000475251	-0,2300068	1_13	Dogs
21	0,5484914	0,03591977	0,02365776	0,009399701	0,008168933	0,00772624	0,003445721	0,02075938	-0,001278739	2_1	Cats
21	0,5537447	0,07236928	0,005654913	0,02732823	0,00089108	0,03080032	0,001471228	0,007251223	0,02712638	2_2	Cats
21	0,4167652	0,279919	0,01093252	0,0172535	0,000532653	0,002857813	0,007791827	0,03814347	0,2502157	2_3	Cats
21	0,5768645	0,04225726	0,03000556	0,01588717	0,0202977	0,004673593	0,001708005	0,014055	0,000945582	2_4	Cats
21	0,6001255	0,009205692	0,03120765	0,01465524	0,00055058	0,02686401	0,002088272	0,003947781	-0,04442494	2_5	Cats
21	0,5728511	0,01987392	0,02057903	0,01427415	0,00488694	0,01580875	0,000879918	0,01900287	-0,01279611	2_6	Cats
21	0,6288533	0,000704765	0,01499489	0,08865417	0,000672314	0,04116397	0,004949745	0,009680743	-0,1105479	2_7	Cats

21	0,5789363	0,002036799	0,07349783	0,01222672	0,000408918	0,06419941	0,001621725	0,0430024	-0,1099185	2_8	Cats
21	0,6722413	0,000005333	0,07199032	0,004780111	0,01139286	0,001919363	0,000533649	0,001403428	-0,07736602	3_1	Horses
21	0,5570068	0,003362764	0,07615028	0,009695215	0,006876035	0,04236561	0,01320593	0,000559429	-0,1033359	3_2	Horses
21	0,9416173	0,000066331	0,0103554	0,001093624	0,004982581	0,000526595	0,000715294	0,003930469	-0,01069413	3_3	Horses
21	0,5170792	0,000069513	0,2323349	0,001651647	0,01303378	0,000144527	0	0,000132614	-0,232606	3_4	Horses
21	0,5097826	0,1078497	0,01092587	0,02606173	0,00534078	0,003229232	0,01452315	0,004394582	0,06403807	4_1	Pigs
21	0,7724756	0,001689852	0,004537896	0,0545223	0,004355296	0,001750133	0,005807535	0,007575257	-0,06001374	4_2	Pigs
21	0,8060508	0,000035224	0,05329061	0,02234993	0,001044008	0,000510838	0,00097961	0,001070414	-0,06831834	5_1	Sheep
21	0,5944844	0,000263135	0,1143985	0,007996117	0,02871291	0,001706115	0,000042596	0,002868734	-0,1146891	5_2	Sheep
**Participant 22**											
**Participant_ID**	**Neutral_Mean**	**Happy_Mean**	**Sad_Mean**	**Angry_Mean**	**Surprised_Mean**	**Scared_Mean**	**Disgusted_Mean**	**Contempt_Mean**	**Valence_Mean**	**Animal_Id**	**Animal**
22	0,4332064	0,1706011	0,02193378	0,00362863	0,04881182	0,001982718	0,01866272	0,001115536	0,1420102	1_1	Dogs
22	0,4327596	0,284146	0,001406597	0,00137308	0,004094776	0,000065271	0,007157806	0,00016947	0,2750487	1_2	Dogs
22	0,3649437	0,2798493	0,000503799	0,006925391	0,001914104	0,00215361	0,00082731	0,000349074	0,2716104	1_3	Dogs
22	0,7008869	0,000000219	0,02208642	0,02762642	0,02701481	0,004123397	0,127289	0,000431681	-0,1532234	1_4	Dogs
22	0,3182952	0,4577909	0,01117541	0,002508772	0,01248972	0,01243909	0,02410538	0,0035016	0,4237902	1_5	Dogs
22	0,4379587	0,2208247	0,03225388	0,000693963	0,009334833	0,004868452	0,07258196	0,00369422	0,1202318	1_6	Dogs
22	0,4612268	0,06087631	0,01718068	0,003079013	0,03924193	0,04954124	0,0569377	0,00670567	-0,05248798	1_7	Dogs
22	0,2668701	0,4875143	0,00608696	0,008813193	0,000025206	0,003316313	0,0665057	0,002025498	0,409499	1_8	Dogs
22	0,5069331	0,000036384	0,107575	0,005555153	0,009167517	0,03052334	0,1421352	0,005810619	-0,2263373	1_9	Dogs
22	0,7070575	0	0,02741324	0,008236112	0,000309667	0,03533109	0,05488264	0,000246253	-0,09377078	1_10	Dogs
22	0,5956136	0,144692	0,001361498	0,00472806	0,000000585	0,00737531	0,1007714	0,000275536	0,0439206	1_11	Dogs
22	0,5606407	0,06621126	0,0393539	0,01239499	0,01734259	0,01425744	0,1240167	0,002000772	-0,08237717	1_12	Dogs
22	0,1217654	0,7497817	0,01776704	0,002027199	0,0110729	0,002533008	0,01564923	0,002087364	0,7193924	1_13	Dogs
22	0,4433809	0,1130736	0,000847209	0,01044269	0,006877638	0,01090883	0,000566848	0,00630148	0,09685322	2_1	Cats
22	0,6483713	0,06636523	0,006793924	0,001685139	0,002035027	0,000011335	0,00014593	0,003151651	0,05844537	2_2	Cats
22	0,7629798	0	0,005735233	0,000588757	0,003816308	0,000435711	0,001777628	0,008774268	-0,007472436	2_3	Cats
22	0,7860446	0	0,004491122	0,002592235	0,008449812	0,000061386	0,000720151	0,01142248	-0,005885448	2_4	Cats
22	0,6886525	0	0,02164216	0,000370681	0,01198882	0,000788478	0,000754046	0,001352468	-0,02231505	2_5	Cats

22	0,7755683	0	0,007385879	0,000986534	0,04609588	0,02102936	0,001324159	0,003372264	-0,02581513	2_6	Cats
22	0,8194966	0	0,010718	0,02691025	0,004742059	0,02298794	0,003156288	0,001354871	-0,04484989	2_7	Cats
22	0,8322144	0	0,01565029	0,003855671	0,000767337	0,03096886	0,005869913	0,000002438	-0,04477926	2_8	Cats
22	0,48816	0,01867976	0,05460991	0,001886268	0,01502473	0,09115639	0,01792872	0,003531498	-0,1133203	3_1	Horses
22	0,9439914	0,000945682	0,001827546	0,000000263	0,001317071	0,001124534	0,000004785	0,01062264	-0,001718397	3_2	Horses
22	0,5496504	0,01249567	0,004703239	0	0,03586249	0,03507199	0,005986085	0,009275368	-0,02647188	3_3	Horses
22	0,5224737	0,009979245	0,03122013	0,007201718	0,003017615	0,1489398	0,02408898	0,003117109	-0,1616242	3_4	Horses
22	0,7594536	0,128684	0,01051982	0,006236443	0,001433296	0,000913998	0,001519747	0,005468949	0,1122373	4_1	Pigs
22	0,7963067	0,1150272	0,01177026	0,000678929	0,000110792	0,00089143	0,000368559	0,004762955	0,1027346	4_2	Pigs
22	0,9122023	0,003665985	0,02796068	0,000026995	0,007177148	0,001954658	0,00008002	0,005453097	-0,02571609	5_1	Sheep
22	0,6060074	0,001101579	0,01704086	0,05176993	0,003671039	0,01873272	0,03778537	0,005717716	-0,06180532	5_2	Sheep
**Participant 23**											
**Participant_ID**	**Neutral_Mean**	**Happy_Mean**	**Sad_Mean**	**Angry_Mean**	**Surprised_Mean**	**Scared_Mean**	**Disgusted_Mean**	**Contempt_Mean**	**Valence_Mean**	**Animal_Id**	**Animal**
23	0,6711955	0,03514565	0,009086832	0,03066872	0,02976523	0,005673516	0,000884858	0,002118012	-0,003774746	1_1	Dogs
23	0,8364941	0,01172882	0,01039577	0,01947323	0,004532975	0,003342047	0,000117366	0,004761958	-0,01909728	1_2	Dogs
23	0,9092762	0,0002637	0,003281362	0,003152991	0,001944043	0,002575222	0,000670579	0,001108326	-0,006730206	1_3	Dogs
23	0,9022407	0	0,02797108	0,000351671	0,003186947	0,001405733	0	0,002183563	-0,02831048	1_4	Dogs
23	0,8413985	0,02026929	0,01703249	0,000916897	0	0,000007889	0,000311754	0,002923372	0,003057019	1_5	Dogs
23	0,5560054	0,3399207	0,004570976	0,001669452	0	0,001138017	0,000002234	0,006604288	0,333204	1_6	Dogs
23	0,8476528	0,000329581	0,01178429	0,000457562	0,00204959	0,000033955	0	0,006678185	-0,01191191	1_7	Dogs
23	0,8776569	0,000092771	0,0201562	0,001335922	0,002653672	0,005465086	0,000105499	0,001586784	-0,02319592	1_8	Dogs
23	0,8828655	0,004856238	0,06381477	0	0,001182717	0,001428311	0	0,004732931	-0,05895854	1_9	Dogs
23	0,8354594	0	0,1034496	0,00297812	0,001682398	0	0,000552492	0,004006814	-0,1034496	1_10	Dogs
23	0,8728506	0,000227899	0,08001334	0,000018983	0,000201428	0,000316094	0	0,001847773	-0,07978544	1_11	Dogs
23	0,769509	0	0,1548768	0	0,008681905	0	0	0,005024532	-0,1548768	1_12	Dogs
23	0,8930678	0	0,009133724	0,008599123	0,003818184	0,00143538	0,003161379	0,006032691	-0,0188171	1_13	Dogs
23	0,5413323	0,01857508	0,1771165	0,04598964	0,0102447	0,00984132	0,006671012	0,03003037	-0,1991056	2_1	Cats
23	0,5740451	0,000010965	0,1293662	0,03206431	0,001034597	0,006637673	0,005426684	0,001037503	-0,1445619	2_2	Cats
23	0,4662554	0,000423405	0,3157504	0,02669863	0,000260869	0,0225148	0,006116973	0,001029149	-0,315327	2_3	Cats

23	0,4801758	0	0,3144344	0,000087207	0,03572658	0,000534302	0,01240182	0,000004137	-0,3144344	2_4	Cats
23	0,6031494	0,0235355	0,1298141	0,000674948	0,01290366	0,000460657	0,000839807	0,01223317	-0,1069436	2_5	Cats
23	0,5278609	0,000000239	0,0949731	0,01080442	0,01264801	0,001187531	0,000000001	0,003638567	-0,1007705	2_6	Cats
23	0,4319914	0,002159528	0,2662211	0,003755806	0,001749113	0,000554421	0,01036856	0,000000006	-0,2642105	2_7	Cats
23	0,7267068	0,000000363	0,000009318	0,000154291	0,05812064	0,000002506	0,00000019	0,001242807	-0,000161638	2_8	Cats
23	0,895708	0,003520046	0,01868617	0,002989473	0,008243673	0,00247757	0,000226887	0,002888788	-0,01804855	3_1	Horses
23	0,9372345	0,001581418	0,002837203	0,001920223	0,007051135	0,001722823	0,000614635	0,002092204	-0,003267881	3_2	Horses
23	0,3869983	0,1809105	0,08547521	0,000137697	0,05836591	0,00000001	0,1729311	0	-0,04659674	3_3	Horses
23	0,9350546	0,005288322	0,001464684	0,000424845	0,000743402	0,000759044	0,000004847	0,002586248	0,0028294	3_4	Horses
23	0,8062308	0,05905212	0,008642829	0,000960364	0,009406315	0,004464445	0,001086452	0,007051324	0,04589766	4_1	Pigs
23	0,9357204	0	0,01689756	0,003644363	0,000774135	0,000385522	0,000007238	0,001602138	-0,0172141	4_2	Pigs
23	0,8973063	0,000535498	0,04273888	0,004221967	0,004853969	0,000713751	0,000491843	0,000783556	-0,04489173	5_1	Sheep
23	0,9116932	0	0,02753829	0,001298142	0,007030445	0,000650532	0	0,004649037	-0,02818912	5_2	Sheep
**Participant 24**											
**Participant_ID**	**Neutral_Mean**	**Happy_Mean**	**Sad_Mean**	**Angry_Mean**	**Surprised_Mean**	**Scared_Mean**	**Disgusted_Mean**	**Contempt_Mean**	**Valence_Mean**	**Animal_Id**	**Animal**
24	0,9053642	0,001112541	0,009353619	0,004116457	0,002193565	0,002043961	0,003259288	0,00101668	-0,01191946	1_1	Dogs
24	0,4338632	0,4035233	0,00141413	0,0103165	0,004752219	0,004778752	0,006581409	0,01193247	0,3859506	1_2	Dogs
24	0,5139701	0,1865368	0,000385114	0,1496512	0,002697302	0,01324283	0,01012791	0,000756958	0,03619917	1_3	Dogs
24	0,8196329	0,07933629	0,01294837	0,007069949	0,002989387	0,001452202	0,000657547	0,001666898	0,05987582	1_4	Dogs
24	0,843133	0,08106846	0,000984062	0,003105324	0,001231019	0,002428135	0,001475803	0,004433446	0,07503443	1_5	Dogs
24	0,855661	0,000037136	0,02755134	0,000707458	0,007429638	0,000588823	0,00112774	0,002353151	-0,02834286	1_6	Dogs
24	0,9729288	0	0,000112112	0,000018135	0,0030157	0,000320202	0,000000079	0,000070897	-0,000396148	1_7	Dogs
24	0,9686908	0	0,001157219	0,000523832	0,000906889	0,001176672	0,000062605	0,003925738	-0,002311171	1_8	Dogs
24	0,9352647	0	0,01260571	0,000584609	0,003681433	0,002889026	0,000450153	0,000952471	-0,01365956	1_9	Dogs
24	0,9493701	0	0,01638995	0,000204344	0,001538905	0,000882674	0,000109307	0,000432844	-0,01639407	1_10	Dogs
24	0,9165197	0	0,02335323	0,000017142	0,001377079	0,001037101	0,000613879	0,001471198	-0,02396681	1_11	Dogs
24	0,9447249	0	0,005312036	0,001820446	0,002620505	0,000230766	0,000000115	0,00134969	-0,006866616	1_12	Dogs
24	0,9261297	0	0,02311344	0,001209453	0,000976111	0,00135596	0,000246786	0,000809144	-0,02365667	1_13	Dogs
24	0,6734703	0,01277304	0,01312105	0,00003262	0,001852282	0,008657167	0,09451968	0,002125229	-0,08279347	2_1	Cats

24	0,7180157	0,02574727	0,03099349	0,002708555	0,002402906	0,01259694	0,02475192	0,001112425	-0,02613364	2_2	Cats
24	0,4722793	0,1599387	0,09077832	0,01002064	0,02024817	0,007296324	0,01544528	0,03237256	0,06260445	2_3	Cats
24	0,4569597	0,08242653	0,1439247	0,009993281	0,002163459	0,005675002	0,002787716	0,08430643	-0,06746908	2_4	Cats
24	0,7520583	0,007582311	0,0309559	0,003832872	0,005939824	0,02172031	0,03994161	0,000000002	-0,06471039	2_5	Cats
24	0,8412735	0,01633527	0,02832112	0,001170378	0,000017978	0,02309633	0,01837168	0,003057704	-0,0371624	2_6	Cats
24	0,6878978	0,000217118	0,02041717	0,01973271	0,005014723	0,01652395	0,05831649	0,000189104	-0,0966116	2_7	Cats
24	0,6719561	0	0,02019403	0,01249475	0,008386818	0,06239998	0,05069743	0	-0,09163885	2_8	Cats
24	0,9376281	0,000722545	0,01670331	0,006321394	0,006067373	0,002098356	0,000622813	0,001414236	-0,01943714	3_1	Horses
24	0,4909074	0,107389	0,001011122	0,04459421	0,008998333	0,0148551	0,003765818	0,01143683	0,05926644	3_2	Horses
24	0,5070872	0,007527385	0,0426308	0,02713347	0,000444469	0,04075801	0,04355375	0,003426416	-0,09249496	3_3	Horses
24	0,9135269	0,002703089	0,009219851	0,001363435	0,004915676	0,001067485	0,000637709	0,00145112	-0,007299196	3_4	Horses
24	0,4402557	0,1200098	0,001143315	0,01354053	0,002743268	0,008753656	0,03832859	0,002370114	0,07483264	4_1	Pigs
24	0,5126827	0,06021573	0,001127095	0,002915089	0,02141648	0,005224315	0,006651981	0,01576031	0,04763506	4_2	Pigs
24	0,5580102	0,1246719	0,002495386	0,05796861	0,0048023	0,005925447	0,009099633	0,0648492	0,06342132	5_1	Sheep
24	0,7866817	0,06818815	0,009062693	0,000875222	0,01201309	0,007880537	0,03368745	0,005028669	0,02872468	5_2	Sheep
**Participant 25**											
**Participant_ID**	**Neutral_Mean**	**Happy_Mean**	**Sad_Mean**	**Angry_Mean**	**Surprised_Mean**	**Scared_Mean**	**Disgusted_Mean**	**Contempt_Mean**	**Valence_Mean**	**Animal_Id**	**Animal**
25	0,5772938	0,03786476	0,02880519	0,02397928	0,002745393	0,006299268	0,009189866	0,03406955	-0,01168651	1_1	Dogs
25	0,3435513	0,4034266	0,000343402	0,08240118	0,00886861	0,01990059	0,04620283	0,00003639	0,2834468	1_2	Dogs
25	0,5000691	0,06470414	0,06561416	0,005251828	0,002474422	0,01342876	0,0127799	0,005098526	-0,02621746	1_3	Dogs
25	0,487171	0,1116583	0,00041914	0,1229181	0,001966781	0,003296597	0,004542677	0,00054282	-0,01256363	1_4	Dogs
25	0,6083295	0,0481147	0,000378713	0,02600238	0,0294487	0,000253877	0,007732676	0,009353397	0,01599883	1_5	Dogs
25	0,565731	0,1113464	0,00018553	0,06873858	0,01077994	0,002170029	0,004868005	0,000296135	0,04076371	1_6	Dogs
25	0,5694646	0,044143	0,01358724	0,000001389	0,05495134	0,01007786	0,004755042	0,07334627	0,02570597	1_7	Dogs
25	0,5924647	0,04196834	0,001589223	0,01815149	0,03288276	0,01598041	0,008274109	0,01124812	0,006968893	1_8	Dogs
25	0,5737002	0,02675112	0,000625951	0,06420435	0,008413906	0,000649752	0,000872774	0,003821679	-0,03807668	1_9	Dogs
25	0,5108548	0,08201852	0,000563239	0,05905233	0,01787377	0,00271585	0,005725132	0,000891495	0,01881728	1_10	Dogs
25	0,6041809	0,02736343	0,002909414	0,0244812	0,04086053	0,00664991	0,001278116	0,002932777	-0,005125111	1_11	Dogs
25	0,6313467	0,000000055	0,000888758	0,03729944	0,01536723	0,009844	0	0,002735607	-0,04048899	1_12	Dogs

25	0,6064942	0,007075513	0,004829403	0,1095966	0,002651668	0,008592741	0,004738823	0,000046951	-0,1053019	1_13	Dogs
25	0,8913001	0	0,0270875	0,0101654	0,001930519	0,000188434	0,000379215	0,003198141	-0,03512015	2_1	Cats
25	0,8234869	0	0,1141715	0,00058662	0,001983511	0	0,000032055	0,03244966	-0,1141715	2_2	Cats
25	0,8832117	0,000303408	0,06295423	0,000862651	0,000015346	0,000133437	0,000461485	0,002817159	-0,06284723	2_3	Cats
25	0,8693857	0,003560822	0,05202026	0,001249754	0,007561925	0,001541125	0,000397587	0,027006	-0,04997233	2_4	Cats
25	0,9246689	0,000054958	0,02694425	0,002400887	0,002067404	0,00101618	0,000003552	0,005227478	-0,02826473	2_5	Cats
25	0,8966782	0,000658288	0,0283267	0,002991901	0,005887192	0,000299959	0,000467125	0,009613039	-0,0286209	2_6	Cats
25	0,8842999	0,000407226	0,02503644	0,00126453	0,002611956	0,001939169	0,000006658	0,001832711	-0,02502666	2_7	Cats
25	0,8107441	0	0,1158988	0,006631556	0,0004347	0,000487475	0	0,003270979	-0,1163122	2_8	Cats
25	0,5311587	0,01481668	0,006447348	0,01206812	0,002929788	0,01543622	0,05265067	0,01614064	-0,04732502	3_1	Horses
25	0,6008939	0,01825919	0,01999278	0,01937138	0,005117607	0,008219187	0,04532366	0,002268441	-0,05888416	3_2	Horses
25	0,5460601	0,001276618	0,01985485	0,000091994	0,000009551	0,03212526	0,000089704	0,001572722	-0,04234342	3_3	Horses
25	0,6403065	0	0,01829921	0,0309954	0,02824172	0,05578892	0,01016151	0,005583275	-0,07390747	3_4	Horses
25	0,5452951	0,08780684	0,003788216	0,04218319	0,02819125	0,0159357	0,07531884	0,004365333	-0,02224966	4_1	Pigs
25	0,4588588	0,01800813	0,01881274	0,06492031	0,000842222	0,000755121	0,1279522	0,002713317	-0,1688938	4_2	Pigs
25	0,6264467	0,03503466	0,04380574	0,01242129	0,005626704	0,003694089	0,01666181	0,01350044	-0,02289087	5_1	Sheep
25	0,5930243	0,006969748	0,007088408	0,05490177	0,01660056	0,0179061	0,02160377	0,014904	-0,06472873	5_2	Sheep
**Participant 26**											
**Participant_ID**	**Neutral_Mean**	**Happy_Mean**	**Sad_Mean**	**Angry_Mean**	**Surprised_Mean**	**Scared_Mean**	**Disgusted_Mean**	**Contempt_Mean**	**Valence_Mean**	**Animal_Id**	**Animal**
26	0,5551447	0,02271118	0,01490708	0,01620278	0,01600593	0,02299884	0,04102456	0,003156013	-0,05210407	1_1	Dogs
26	0,487127	0,1050929	0,03813526	0,05369737	0,02737212	0,007136229	0,0301925	0,008065373	0,004110217	1_2	Dogs
26	0,4386446	0,001441556	0,0921255	0,208177	0,000879767	0	0,000000564	0,00567895	-0,2848571	1_3	Dogs
26	0,5763856	0,02195255	0,04038059	0,02912637	0,00437461	0,03458786	0,00385155	0,009479164	-0,07378808	1_4	Dogs
26	0,5733711	0,05049359	0,001877559	0,08207913	0,003458751	0,001980701	0,07804095	0,000820492	-0,07400235	1_5	Dogs
26	0,5444202	0,05534491	0,01729677	0,02613876	0,001306991	0,03548334	0,03819	0,007734936	-0,02538976	1_6	Dogs
26	0,553421	0,09247652	0,03072601	0,006014676	0,000001094	0,000484681	0,01668759	0,02364939	0,04739114	1_7	Dogs
26	0,4942165	0,1676354	0,000026995	0,0149277	0,002402438	0,003613536	0,09345721	0,01557626	0,0681032	1_8	Dogs
26	0,6028033	0,08950605	0,02327884	0,01018773	0,000959335	0,005142528	0,01964932	0,005038699	0,05380033	1_9	Dogs
26	0,4942966	0,01067608	0,03943693	0,004801529	0	0,001272832	0,08966351	0,00249065	-0,1087647	1_10	Dogs

26	0,6168309	0,07055829	0,02445951	0,0336266	0,003460075	0,01345482	0,0190492	0,00904694	0,009944339	1_11	Dogs
26	0,6811673	0,00810132	0,001711981	0,02521347	0,04433132	0,02360473	0,000351144	0,000029667	-0,03572296	1_12	Dogs
26	0,5055718	0,1056269	0,02249388	0,04347843	0,01380162	0,05123972	0,02377081	0,002075656	0,02334475	1_13	Dogs
26	0,8032381	0,1145785	0,01707565	0,005842417	0,003625197	0,002415327	0,005415767	0,0036391	0,08729798	2_1	Cats
26	0,8090242	0,1105926	0,001931156	0,001944757	0,008753882	0,009224407	0,003378705	0,003008955	0,09769338	2_2	Cats
26	0,9176548	0,000000082	0,01024541	0,000788295	0,004466908	0,004765115	0,000150215	0,004477192	-0,0141053	2_3	Cats
26	0,9147899	0	0,01886371	0,001085236	0,000023863	0,00048851	0,002999406	0,000809338	-0,02024824	2_4	Cats
26	0,901493	0	0,03337313	0,000596716	0,002040851	0,000653451	0,001407963	0,000888396	-0,03385032	2_5	Cats
26	0,9367017	0	0,02114481	0,005114857	0,000955089	0,001240264	0,000198484	0,000678729	-0,02408969	2_6	Cats
26	0,9725699	0	0,000041675	0,02696291	0,000343463	0,000172937	0	0,002056257	-0,02696291	2_7	Cats
26	0,9300433	0	0,02395163	0,005644499	0,000306283	0,000317885	0,000023279	0,002921152	-0,02671629	2_8	Cats
26	0,6171364	0,000337336	0,009591271	0,03087166	0,01050723	0,01788287	0,008311339	0,00212388	-0,04657223	3_1	Horses
26	0,399466	0,001875857	0,1633783	0,04403682	0,01190074	0,04745914	0,1082746	0,0071607	-0,2225107	3_2	Horses
26	0,745564	0,003594674	0,001014495	0,02305032	0,01768984	0,004033005	0,006090478	0,000266344	-0,02373358	3_3	Horses
26	0,7599077	0,002286955	0,01927952	0,001315517	0,000654639	0,01500924	0,000000043	0,002014714	-0,02942877	3_4	Horses
26	0,4617134	0,02776722	0,09272361	0,04972292	0,001029223	0,000000136	0,053752	0,02069879	-0,115588	4_1	Pigs
26	0,3842153	0,1287659	0,003772455	0,003113712	0,1061196	0,04069605	0,000272687	0,004460437	0,0824549	4_2	Pigs
26	0,4668138	0,006830878	0,06694964	0,007320342	0,002531502	0,03820632	0,09077754	0,00024895	-0,1230245	5_1	Sheep
26	0,4409801	0,1410067	0	0,08873829	0,004164882	0	0,03547204	0,000000009	0,05226842	5_2	Sheep
**Participant 27**											
**Participant_ID**	**Neutral_Mean**	**Happy_Mean**	**Sad_Mean**	**Angry_Mean**	**Surprised_Mean**	**Scared_Mean**	**Disgusted_Mean**	**Contempt_Mean**	**Valence_Mean**	**Animal_Id**	**Animal**
27	0,4158637	0,004757363	0,02681251	0,2359657	0,001589358	0,000615507	0,017856	0,002010474	-0,2473504	1_1	Dogs
27	0,6639507	0,03480386	0,02926883	0,05690832	0,000310003	0,01048662	0,00029846	0,001467101	-0,05211138	1_2	Dogs
27	0,4472853	0,06464185	0,005788314	0,01183959	0,06863484	0,003833417	0,02594928	0,002524839	0,03672362	1_3	Dogs
27	0,4094128	0,005837563	0,1876149	0,1195664	0,000859814	0,000585763	0,01328376	0,001366398	-0,2959114	1_4	Dogs
27	0,4505169	0,000063432	0,1114346	0,06539153	0,00130619	0,008437431	0,02183136	0,02952248	-0,1760818	1_5	Dogs
27	0,4813596	0,001268238	0,06332638	0,03102094	0,001082364	0,02750718	0,04919745	0,01274644	-0,1166716	1_6	Dogs
27	0,5667568	0,02200224	0,1470519	0,005465602	0,003253195	0,000000015	0,05476786	0,006085346	-0,1288413	1_7	Dogs
27	0,4539979	0,01196557	0,1180198	0,02798251	0,00713358	0,01614159	0,1328982	0,0048061	-0,1860612	1_8	Dogs

27	0,4745895	0,002025247	0,1015467	0,0694356	0	0,007830166	0,06551035	0,004499028	-0,174045	1_9	Dogs
27	0,415215	0,000021431	0,1067294	0,06607667	0,000018411	0,03417408	0,1101903	0,04530293	-0,2093746	1_10	Dogs
27	0,4034149	0,004741326	0,1998404	0,000913026	0,00069913	0,05601195	0,1183421	0,004667274	-0,2571955	1_11	Dogs
27	0,4458641	0,005969393	0,0555605	0,08170063	0,000209118	0,04328908	0,06196688	0,05297904	-0,1700264	1_12	Dogs
27	0,5756763	0,01265334	0,04644503	0,0543476	0,007531403	0,006135568	0,05257625	0,01608828	-0,09669846	1_13	Dogs
27	0,5986767	0,000017703	0,00273431	0,003621479	0,04554017	0,01859997	0,02956794	0,007261546	-0,04107766	2_1	Cats
27	0,5003623	0,09766841	0,000499818	0,00864089	0,04093567	0,00810191	0,001407798	0,003320489	0,08398625	2_2	Cats
27	0,5075885	0,03831384	0,000416401	0,01427017	0,07923123	0,007305696	0,02268421	0,002296961	0,004506176	2_3	Cats
27	0,6246814	0,01103404	0,001990177	0,003077198	0,03763163	0,006996547	0,02352221	0,01082385	-0,01644685	2_4	Cats
27	0,5445933	0,08218258	0,001894673	0,01450974	0,03754926	0,006166658	0,01259207	0,001023547	0,05675429	2_5	Cats
27	0,4971312	0,05563738	0,000265035	0,04981192	0,03944737	0,000440475	0,01884417	0,001699029	0,00471937	2_6	Cats
27	0,4634785	0,120657	0,000807671	0,06581406	0,00181089	0,000000847	0,00711913	0,000535507	0,05265739	2_7	Cats
27	0,5071105	0,09083641	0,000777074	0,03670315	0	0,004511929	0,06267859	0,005299679	0,01441099	2_8	Cats
27	0,3995485	0,001877937	0,1733868	0,08571798	0,003088148	0,009283671	0,0256751	0,005961779	-0,252087	3_1	Horses
27	0,4630622	0,08261029	0,02302875	0,003005883	0,04124363	0,02363213	0,000646526	0,002031137	0,04142327	3_2	Horses
27	0,6328946	0,02098934	0,02707008	0,007669293	0,003250685	0,01092498	0,000652893	0,01309026	-0,0188796	3_3	Horses
27	0,4655551	0,01597357	0,05342245	0,06291631	0,002848664	0,02536803	0,03904569	0,000822043	-0,1315933	3_4	Horses
27	0,3474919	0,3087891	0,004476686	0,005216834	0,000914808	0,000807838	0,00183675	0,002748268	0,2993656	4_1	Pigs
27	0,7044623	0,005478549	0	0,00964471	0,00861538	0,00049963	0,001348951	0,000696281	-0,005587576	4_2	Pigs
27	0,5355825	0,0172185	0,04056615	0,003301798	0,01857355	0,000765038	0,005346612	0,003395198	-0,02401817	5_1	Sheep
27	0,3914652	0,01339831	0,1744476	0,06111403	0,01617993	0,01113131	0,09588359	0,003390814	-0,2216705	5_2	Sheep
**Participant 28**											
**Participant_ID**	**Neutral_Mean**	**Happy_Mean**	**Sad_Mean**	**Angry_Mean**	**Surprised_Mean**	**Scared_Mean**	**Disgusted_Mean**	**Contempt_Mean**	**Valence_Mean**	**Animal_Id**	**Animal**
28	0,4641806	0,05210785	0,01608057	0,00350618	0,007396691	0,006470065	0,000842453	0,000871212	0,02844162	1_1	Dogs
28	0,2813351	0,4284431	0,03130809	0,000103254	0,002815546	0	0	0,00080492	0,397135	1_2	Dogs
28	0,6322061	0,01405221	0,000008187	0,02618684	0,009787987	0,002073504	0,0112607	0,000021083	-0,02126054	1_3	Dogs
28	0,3709129	0,2411934	0,02338535	0,001656026	0,002971274	0,00413102	0,001181138	0,000721109	0,2167089	1_4	Dogs
28	0,363073	0,216876	0,07367754	0,00349066	0,00455721	0,002270564	0,000380931	0,002738185	0,1422591	1_5	Dogs
28	0,4149134	0,08213446	0,1165186	0,001562733	0,01672035	0,01277648	0,002695451	0,002099314	-0,04118187	1_6	Dogs

28	0,4379726	0,008723224	0,1182733	0,004279559	0,001372123	0,04309439	0,000074967	0,004489345	-0,125165	1_7	Dogs
28	0,4259833	0,1449056	0,007553116	0,002449118	0,004494309	0,01207932	0,000094417	0,001793311	0,1257739	1_8	Dogs
28	0,487738	0,0132976	0,02415833	0,000959539	0,000194022	0,003870398	0,00162341	0,007051551	-0,01292871	1_9	Dogs
28	0,4311693	0,003337564	0,1908158	0,000333376	0,04178712	0,01039693	0,000415027	0,001460439	-0,1915164	1_10	Dogs
28	0,448557	0,002141023	0,07653284	0,005369314	0,1356219	0,04006594	0,001211457	0,000163877	-0,09685309	1_11	Dogs
28	0,4818234	0	0,08447711	0,001412407	0,005561384	0,007107971	0,000002102	0,005707589	-0,08667723	1_12	Dogs
28	0,4046797	0,1814691	0,06417082	0,01272547	0,00671368	0,000677253	0,000300083	0,008007788	0,1110756	1_13	Dogs
28	0,5549143	0,0249535	0,03298629	0,02099126	0,00007585	0,000751804	0,06234021	0,01324723	-0,06305315	2_1	Cats
28	0,6462378	0,09713744	0,01205057	0,07342748	0,000079181	0,000155192	0,01607515	0,001838574	0,01482075	2_2	Cats
28	0,7586628	0,01893543	0,02268072	0,003043244	0,004234835	0,007515668	0,00338203	0,004245784	-0,01089218	2_3	Cats
28	0,4966223	0,04768418	0,01875453	0,0729595	0,000180305	0,003068716	0,01127262	0,00161499	-0,0343541	2_4	Cats
28	0,415529	0,004249396	0,02094488	0,2230441	0,000133328	0,000003342	0,07263454	0,00535986	-0,2405401	2_5	Cats
28	0,5325021	0,05777277	0,01867507	0,1092906	0,002217604	0,007464101	0,02341217	0,002937299	-0,07685115	2_6	Cats
28	0,7456571	0,02161548	0,000356241	0,003119292	0,02902921	0,01499232	0,0153692	0,005536639	-0,00798598	2_7	Cats
28	0,6384966	0,09038137	0,006845307	0,008673185	0,000838757	0,01188284	0,04497544	0,01139943	0,02952256	2_8	Cats
28	0,758481	0,000150143	0,03192771	0,001824802	0,001024353	0,002395425	0,000789586	0,003905469	-0,03395715	3_1	Horses
28	0,7130786	0	0,004485088	0,09680315	0,002786925	0,001148291	0,0140384	0,000767976	-0,1058769	3_2	Horses
28	0,8856322	0,000000723	0,007026766	0,08482172	0,000012554	0,000148606	0,001138262	0,00071561	-0,08816434	3_3	Horses
28	0,4751681	0,04849835	0,02739706	0,01699077	0,02421818	0,006916225	0,001055113	0,003507808	0,002698556	3_4	Horses
28	0,4869825	0	0,00132793	0,2994404	0,01062748	0,003531664	0,02036733	0,000495412	-0,3074087	4_1	Pigs
28	0,6184352	0,01987195	0,003444606	0,03029131	0,01062267	0,04181118	0,004994183	0,003874585	-0,04033363	4_2	Pigs
28	0,5761612	0,00667294	0,000131596	0,07772619	0,002182961	0,001307685	0,03212478	0,00100119	-0,09416582	5_1	Sheep
28	0,4863351	0,08589486	0,01423536	0,01712647	0,00941465	0,01065719	0,002043405	0,000472236	0,05594597	5_2	Sheep
**Participant 29**											
**Participant_ID**	**Neutral_Mean**	**Happy_Mean**	**Sad_Mean**	**Angry_Mean**	**Surprised_Mean**	**Scared_Mean**	**Disgusted_Mean**	**Contempt_Mean**	**Valence_Mean**	**Animal_Id**	**Animal**
29	0,4381295	0,001258317	0,0127936	0,2131163	0,003620347	0,002641563	0,1086788	0,002989729	-0,3107556	1_1	Dogs
29	0,4333325	0,004226767	0,000005628	0,01647228	0,001484326	0,003073007	0,2277029	0,000000084	-0,2248844	1_2	Dogs
29	0,6832396	0,05369576	0,06125836	0,01292409	0,02617031	0,000639791	0,004549657	0,001062087	-0,01322359	1_3	Dogs
29	0,62669	0,01121378	0,001095634	0,07623393	0,003015133	0,000854074	0,06397295	0,002483095	-0,1270395	1_4	Dogs

29	0,5354488	0,01991996	0,002138112	0,1491303	0,00722797	0,001339641	0,006176575	0,000003428	-0,13542	1_5	Dogs
29	0,5280076	0,07960829	0,005935256	0,01596371	0,004388851	0,001713542	0,03625474	0,000241434	0,02216115	1_6	Dogs
29	0,5984828	0,01249481	0,02293189	0,000030183	0,01116816	0,003863126	0,01040872	0,000253055	-0,01851659	1_7	Dogs
29	0,5397599	0,04926435	0,004097009	0,02998686	0,008828493	0,006273607	0,02905799	0,001496138	-0,005808456	1_8	Dogs
29	0,6554704	0,001397839	0,001085321	0,005770092	0,002020348	0,002592713	0,02643052	0,001468013	-0,03070023	1_9	Dogs
29	0,6254777	0,006825343	0,005797047	0,002218541	0,02490217	0,005888793	0,02531686	0,000267343	-0,02343457	1_10	Dogs
29	0,6932164	0	0,000678768	0,01152048	0,006084007	0,002988898	0,008907855	0,000072711	-0,02063886	1_11	Dogs
29	0,6447285	0,01270686	0,000039192	0,08092996	0,009079813	0,00401198	0,01853584	0,000070133	-0,07025482	1_12	Dogs
29	0,6017559	0,03722796	0,01778323	0,04496381	0,005012681	0,005648254	0,01308575	0,007913218	-0,03126956	1_13	Dogs
29	0,4471456	0,02083073	0,09417631	0,008162289	0,002506013	0,02194677	0,1356411	0,01060422	-0,1918369	2_1	Cats
29	0,506	0,004860817	0,000000464	0,03520028	0	0,006988943	0,2206603	0,02063184	-0,223002	2_2	Cats
29	0,4123498	0,000011121	0,1595091	0,03776936	0,000002774	0,008426967	0,1353197	0,008595861	-0,2116219	2_3	Cats
29	0,3988525	0	0,1507925	0,001380649	0,0014187	0,07766773	0,1148821	0,00159503	-0,2337221	2_4	Cats
29	0,4860218	0,008212539	0,135887	0,04512604	0,004546877	0,007967293	0,07666329	0,000880453	-0,1712394	2_5	Cats
29	0,3769507	0,00023202	0,2357348	0,01128066	0,000177641	0,01564383	0,04728164	0,001807397	-0,268938	2_6	Cats
29	0,4202864	0,000318373	0,1217615	0,02156685	0	0,006274409	0,08074383	0,002189941	-0,1969908	2_7	Cats
29	0,4394618	0,0149487	0,1211258	0,04706762	0,008564143	0,03439206	0,09123887	0,001448898	-0,2007552	2_8	Cats
29	0,5903594	0,00210883	0,003698948	0,1553151	0,005213286	0,001063524	0,02203238	0,000541762	-0,1696831	3_1	Horses
29	0,7067899	0,003942416	0,0403388	0,006226738	0,009324904	0,01549183	0,000000017	0,006174787	-0,04595998	3_2	Horses
29	0,9082042	0,000313866	0,02357621	0,01901511	0,002643725	0,00641118	0,000517244	0,003262315	-0,03375385	3_3	Horses
29	0,675025	0,002273605	0,005369196	0,02317622	0,01913703	0,01047928	0,007662298	0,000846272	-0,03121622	3_4	Horses
29	0,5339869	0,06731299	0,009755609	0,01895713	0,002939277	0,03709755	0,02511775	0,01362024	0,001818449	4_1	Pigs
29	0,9331901	0	0,003967169	0,03188835	0,001477801	0,001011543	0,005647504	0,006208551	-0,03964394	4_2	Pigs
29	0,6221336	0,03518612	0,005057268	0,04659867	0,01228893	0,01986942	0,02345961	0,009367136	-0,04975799	5_1	Sheep
29	0,6308724	0,03707938	0,000763799	0,002401259	0,0139306	0,003559596	0,01709151	0,000471991	0,01822041	5_2	Sheep
**Participant 30**											
**Participant_ID**	**Neutral_Mean**	**Happy_Mean**	**Sad_Mean**	**Angry_Mean**	**Surprised_Mean**	**Scared_Mean**	**Disgusted_Mean**	**Contempt_Mean**	**Valence_Mean**	**Animal_Id**	**Animal**
30	0,5811527	0,07144124	0,01261641	0,000663072	0,005998944	0,05987326	0,03334682	0,004145948	-0,02418905	1_1	Dogs
30	0,6607224	0,1168262	0,000252623	0,009957039	0,011711	0,008945066	0,000000011	0,01515943	0,1019664	1_2	Dogs

30	0,7828517	0	0,000564906	0,2089873	0,003601527	0	0,000208801	0,004753417	-0,2091949	1_3	Dogs
30	0,6312823	0,03474527	0,0767431	0,004935161	0,02499761	0,00647064	0,01158333	0,001687145	-0,05671471	1_4	Dogs
30	0,5641578	0,004429804	0,07677796	0,001506014	0,01044651	0,04741623	0,008588925	0,006193227	-0,1177898	1_5	Dogs
30	0,7906356	0,000000147	0,000002318	0,005688902	0,001606075	0,03666227	0,00484924	0,000000153	-0,03866135	1_6	Dogs
30	0,8297222	0,00001373	0,000030977	0,0154614	0,0180286	0,000232504	0,005176195	0,000144146	-0,01678685	1_7	Dogs
30	0,663873	0,02126852	0,05780555	0,00315691	0,03327482	0,01631049	0,02440891	0,005357183	-0,07225543	1_8	Dogs
30	0,646323	0,01145077	0,001167389	0,01121259	0,02080237	0,0585878	0,01851106	0,003009596	-0,0670177	1_9	Dogs
30	0,5338477	0,1292312	0,01342997	0,02736311	0,01752654	0,04687195	0,001543532	0,006100771	0,06654915	1_10	Dogs
30	0,5649899	0,02884734	0,01116835	0,01925703	0,04763183	0,06743932	0,001229819	0,004694635	-0,05348513	1_11	Dogs
30	0,6358753	0,02347982	0,000000199	0,01200095	0,006407696	0,0496305	0,00190117	0,000947209	-0,02615069	1_12	Dogs
30	0,6160854	0,000414575	0,000623784	0,06577358	0,0594863	0,05103155	0,000004523	0,007474455	-0,0878377	1_13	Dogs
30	0,4448546	0,1760593	0,005371438	0,002022886	0,001099369	0,004469557	0,003836584	0,002048641	0,1640941	2_1	Cats
30	0,4937447	0,05238159	0,06956431	0,01976507	0,005153491	0,005503231	0,006635311	0,004645042	-0,03412579	2_2	Cats
30	0,4997745	0,1043734	0,07739681	0,03499167	0,009290513	0,02537535	0,004350959	0,00172757	-0,02913628	2_3	Cats
30	0,4137779	0,01417916	0,1549897	0,000837801	0,03665664	0,01255068	0,000024846	0,001087828	-0,1452227	2_4	Cats
30	0,5414154	0,001710374	0,03877507	0,006183562	0,01953258	0,001610325	0,000015275	0,004564283	-0,04281023	2_5	Cats
30	0,4711544	0,000416955	0,1229101	0,006894595	0,01968027	0,01261692	0,000095796	0,001281017	-0,1379372	2_6	Cats
30	0,4738361	0,0393768	0,0359793	0,00765816	0,01268038	0,000587265	0,000031704	0,000511923	-0,004260278	2_7	Cats
30	0,4625868	0,05020848	0,003508356	0,001729745	0,04653374	0,000977668	0,000849818	0,000643701	0,04432646	2_8	Cats
30	0,5619291	0,03854591	0,007106273	0,009438458	0,002397979	0,04155017	0,04471306	0,007338639	-0,05227436	3_1	Horses
30	0,8628116	0,000019151	0,008009645	0,1454669	0,002862396	0,000837615	0,000152513	0,002276576	-0,1458699	3_2	Horses
30	0,3336233	0,001633368	0,4812395	0,000636967	0,000344285	0,001019175	0,000787029	0,000131791	-0,4799164	3_3	Horses
30	0,6000185	0,001252447	0,01660155	0,03913438	0,005869932	0,02294259	0,03293822	0,008672985	-0,08913308	3_4	Horses
30	0,7904674	0,000035165	0,004772549	0,1916141	0,000014661	0,001875333	0,000332844	0,001282968	-0,1937799	4_1	Pigs
30	0,633761	0,000000009	0,08528259	0	0,000248905	0,01187786	0,000723439	0	-0,09326958	4_2	Pigs
30	0,8970472	0,000088606	0,002524122	0,07324369	0,002988775	0,001264046	0,000001986	0,005817451	-0,07409278	5_1	Sheep
30	0,5230988	0,1334407	0,00450316	0,007918074	0,008301234	0,0571931	0,04062552	0,003501143	0,03055552	5_2	Sheep
**Participant 31**											
**Participant_ID**	**Neutral_Mean**	**Happy_Mean**	**Sad_Mean**	**Angry_Mean**	**Surprised_Mean**	**Scared_Mean**	**Disgusted_Mean**	**Contempt_Mean**	**Valence_Mean**	**Animal_Id**	**Animal**

31	0,7791847	0,000422244	0,000017834	0,06754921	0,009042682	0,005697077	0,01382415	0,001998571	-0,07678717	1_1	Dogs
31	0,8172264	0	0,001651165	0,1852184	0,00673621	0	0,009102617	0,003750314	-0,1880076	1_2	Dogs
31	0,2052069	0,000194261	0,7112227	0,001463351	0,000646507	0,000930217	0,007668504	0,000832064	-0,7161372	1_3	Dogs
31	0,7905734	0	0,001507448	0,2392958	0,003354762	0	0,000578614	0,01309441	-0,2405505	1_4	Dogs
31	0,793429	0	0,004287024	0,2430343	0,000000013	0	0,001413492	0,002561271	-0,2437623	1_5	Dogs
31	0,8153237	0	0,00001962	0,1853783	0,000484837	0,001420407	0,000190417	0,003223731	-0,1855641	1_6	Dogs
31	0,7129456	0	0,01267796	0,2942348	0,001638291	0,000037081	0,005796813	0,000176391	-0,2944768	1_7	Dogs
31	0,7424768	0	0,002076154	0,2722232	0,000553318	0,001063364	0,002786842	0,002103145	-0,2739502	1_8	Dogs
31	0,8903052	0	0,005518731	0,06035631	0,0007647	0,000812711	0,01667241	0,003650968	-0,0739093	1_9	Dogs
31	0,8991473	0	0,00569763	0,06459395	0,000044365	0	0,002210607	0,00142818	-0,06983641	1_10	Dogs
31	0,9181719	0	0,001528764	0,04927241	0,000122338	0,001115876	0,00038855	0,008458553	-0,04995073	1_11	Dogs
31	0,9486444	0	0,003785481	0,04363525	0,002053618	0,000548949	0,001771945	0,008330509	-0,04623079	1_12	Dogs
31	0,9091439	0	0,005233886	0,02024026	0,004690486	0,00006338	0,001416223	0,01060356	-0,02238867	1_13	Dogs
31	0,4398677	0,05898705	0,000252478	0,1616244	0,008319519	0,00182587	0,007877728	0,000126409	-0,1026373	2_1	Cats
31	0,6706111	0,001834905	0,001322348	0,190312	0,001789321	0,000129889	0,01345621	0,001394934	-0,1936004	2_2	Cats
31	0,6862945	0,000675893	0,000558461	0,02814963	0,02115579	0,009832185	0,004310142	0,000046196	-0,03402855	2_3	Cats
31	0,6219283	0,002461038	0,00686386	0,1163027	0,005630677	0,000538302	0,005601173	0,003734702	-0,1187354	2_4	Cats
31	0,4884851	0,01241324	0,000646446	0,2521158	0,008561876	0,004401898	0,05371832	0,000034892	-0,2499004	2_5	Cats
31	0,5435196	0,01388244	0,002577957	0,03656634	0,006748364	0,005593268	0,04613532	0,000829502	-0,06034516	2_6	Cats
31	0,3422687	0,000000001	0,000872982	0,4783164	0,000404947	0,000363598	0,02786478	0,000000001	-0,4859098	2_7	Cats
31	0,2621066	0	0,004049	0,6904324	0,001156404	0	0,006113155	0,000041816	-0,6904324	2_8	Cats
31	0,9206391	0,000577014	0,001411955	0,0286769	0,000000021	0,000313426	0,01073894	0,000762078	-0,0338051	3_1	Horses
31	0,8321466	0,000472191	0,0296252	0,02982443	0,01101658	0,007683758	0,001462922	0,003663791	-0,04064172	3_2	Horses
31	0,9488481	0,000002525	0,00405197	0,008980598	0,01669247	0,002417955	0,001386928	0,001895856	-0,01157779	3_3	Horses
31	0,9306161	0,000044111	0,0055698	0,0612214	0,00223979	0,000667102	0,001067838	0,004649212	-0,06236788	3_4	Horses
31	0,5713495	0,006888072	0,1333678	0,00017116	0,001524421	0,01889306	0,003076918	0,002036226	-0,1454941	4_1	Pigs
31	0,8828942	0,000191572	0,04863348	0,000006706	0,005776266	0,001102474	0,02124785	0,000061358	-0,06312241	4_2	Pigs
31	0,395798	0,001105797	0,4691291	0,000687759	0,000000085	0,001839616	0,006511362	0,00021408	-0,4683482	5_1	Sheep
31	0,900567	0,000252796	0,003491061	0,0461206	0,00385596	0,001027854	0,001307648	0,004755193	-0,04685488	5_2	Sheep
**Participant 32**											

Participant_ID	Neutral_Mean	Happy_Mean	Sad_Mean	Angry_Mean	Surprised_Mean	Scared_Mean	Disgusted_Mean	Contempt_Mean	Valence_Mean	Animal_Id	Animal
32	0,3677468	0,000125376	0,5614724	0,000464154	0	0,000049074	0,000028029	0,000068654	-0,561347	1_1	Dogs
32	0,3489694	0,000224872	0,5299605	0,001351746	0	0,000453265	0,00085691	0,000850591	-0,5309486	1_2	Dogs
32	0,7223571	0,000187584	0,1824394	0,02103949	0,01024486	0,009055407	0,004474857	0,001595172	-0,2033332	1_3	Dogs
32	0,3982829	0,05681974	0,3210786	0,000047882	0,007649154	0,02429702	0,01137718	0,002023228	-0,2868513	1_4	Dogs
32	0,6817173	0,0004012	0,01990424	0,01047216	0,008348472	0,006104384	0,000696017	0,001372449	-0,02862002	1_5	Dogs
32	0,6488327	0,000000001	0,1785029	0,000122688	0,001425427	0,01009654	0,000710768	0,000368428	-0,1880361	1_6	Dogs
32	0,3920209	0	0,4721144	0,000000766	0,000007247	0,001429719	0,000571979	0,000023101	-0,4721144	1_7	Dogs
32	0,341696	0	0,5606989	0,00003396	0	0,000478698	0,000595856	0,000179276	-0,560884	1_8	Dogs
32	0,3195702	0,01580215	0,4984225	0,002643492	0,000423517	0,02432193	0,01237364	0,01600916	-0,5020752	1_9	Dogs
32	0,4228744	0,07764328	0,03019604	0,000116923	0,01347391	0,1096238	0,007382091	0,000040403	-0,05064695	1_10	Dogs
32	0,3747424	0,00170226	0,4130338	0,001575386	0,00234729	0,014108	0,005589752	0,000217275	-0,423856	1_11	Dogs
32	0,6158431	0	0,1293304	0,006312083	0,00020771	0,008881713	0,0039324	0,000052096	-0,1376559	1_12	Dogs
32	0,7011364	0	0,00026354	0,03821827	0	0,000000001	0,02853335	0,001139459	-0,05683179	1_13	Dogs
32	0,5869426	0,1314039	0,01169046	0,02653144	0,009978836	0,02768492	0,01116614	0,00391754	0,07482833	2_1	Cats
32	0,5944008	0,07523162	0,001694915	0,0454318	0,0187604	0,03152869	0,01163351	0,005645411	0,00993962	2_2	Cats
32	0,5048163	0,06602421	0,01913045	0,001471044	0,004142132	0,000652654	0,04247212	0,009874525	0,02173417	2_3	Cats
32	0,554601	0,01676613	0,002901424	0,07909253	0,004801164	0,06949429	0,04169352	0,002112578	-0,1154393	2_4	Cats
32	0,52929	0,002190697	0,006205303	0,02627075	0,01407141	0,0424023	0,02019187	0,004642125	-0,07767033	2_5	Cats
32	0,5838882	0,03415605	0,005187277	0,04592922	0,00897869	0,003635688	0,05127582	0,00773056	-0,06240242	2_6	Cats
32	0,5570138	0,09861878	0,007167108	0,0379403	0,02890525	0,01380302	0,01336576	0,006520858	0,03799287	2_7	Cats
32	0,537724	0,007868821	0,001421372	0,07884593	0,009489807	0,02065616	0,05034664	0,002506994	-0,1218823	2_8	Cats
32	0,8800501	0,000257302	0,03666632	0,01157601	0,000037965	0,01234376	0,000777823	0,003650045	-0,03640902	3_1	Horses
32	0,4373995	0,001230549	0,2611148	0,002300506	0,000215828	0,002160879	0,009456568	0,002802775	-0,263008	3_2	Horses
32	0,5123011	0,000057662	0,0891069	0,008787557	0,000147556	0,08692933	0,002378786	0,000828046	-0,1525113	3_3	Horses
32	0,8945052	0,000442346	0,01707829	0,01033424	0,000002378	0,003173901	0,001445816	0,005658539	-0,01663595	3_4	Horses
32	0,8971717	0,01061428	0,0531472	0,000706342	0,006348015	0,001125676	0,01228844	0,000356682	-0,04382862	4_1	Pigs
32	0,5757061	0,000000697	0,001765178	0,002051265	0,000180509	0,1801036	0,008013474	0,003850395	-0,1906868	4_2	Pigs
32	0,7873801	0,002589227	0,08617194	0,007259828	0,03348288	0,004245346	0,01021838	0,002185081	-0,0882481	5_1	Sheep
32	0,2792362	0,000281057	0,5733579	0,00098176	0,004136163	0,002878228	0,003474957	0,000109693	-0,5758247	5_2	Sheep

**Participant 33**											
**Participant_ID**	**Neutral_Mean**	**Happy_Mean**	**Sad_Mean**	**Angry_Mean**	**Surprised_Mean**	**Scared_Mean**	**Disgusted_Mean**	**Contempt_Mean**	**Valence_Mean**	**Animal_Id**	**Animal**
33	0,7481698	0,04200117	0,08189581	0,01646828	0,00150852	0,005247117	0,01515951	0,004066254	-0,06660298	1_1	Dogs
33	0,7486917	0	0,1792307	0,0200351	0,004758085	0,00886986	0,004873137	0,009003176	-0,1964786	1_2	Dogs
33	0,307235	0	0,03460411	0,04769903	0,000017939	0,3488184	0,002390745	0,000205996	-0,390812	1_3	Dogs
33	0,7589601	0,000001536	0,1722023	0,002675517	0,00255606	0,006084819	0,01425795	0,001103474	-0,1779262	1_4	Dogs
33	0,7195283	0	0,1435693	0,07254269	0,002042624	0,001211875	0,01886532	0,000382426	-0,2072472	1_5	Dogs
33	0,7919181	0	0,09753828	0,02032951	0,007895132	0,001641926	0,005698778	0,007980009	-0,1176798	1_6	Dogs
33	0,7585888	0,003044697	0,1251881	0,02122913	0,00493578	0,006833136	0,01499438	0,002259999	-0,1423849	1_7	Dogs
33	0,2806454	0,001010648	0,5358586	0,000298984	0,01717106	0,006633011	0,02909513	0,000599412	-0,534848	1_8	Dogs
33	0,7799951	0,000000132	0,1203427	0,02062031	0,003996919	0,002148064	0,01735203	0,001093127	-0,1402362	1_9	Dogs
33	0,8024654	0	0,06753297	0,007349402	0,002515658	0,00312721	0,009564529	0,002425052	-0,07454003	1_10	Dogs
33	0,698966	0,001608998	0,1628313	0,007440436	0,007219322	0,008540942	0,02502271	0,002017913	-0,1655076	1_11	Dogs
33	0,8321401	0,000032994	0,002212381	0,0163943	0,01909604	0,02124425	0,000134684	0,002311889	-0,02982437	1_12	Dogs
33	0,9608151	0	0,000676784	0,000002272	0,000013975	0,000097637	0,000000274	0,000332833	-0,000763753	1_13	Dogs
33	0,8135868	0,000245332	0,004375468	0,2072183	0,002324963	0,000560241	0,00207756	0,002712068	-0,209476	2_1	Cats
33	0,8847156	0,000034824	0,004220444	0,02682232	0,004213781	0,000329156	0,00473324	0,003743579	-0,03233823	2_2	Cats
33	0,8702149	0,002198644	0,01266072	0,07654206	0,007310236	0,001437426	0,001423512	0,004471373	-0,0806362	2_3	Cats
33	0,9163402	0,007530801	0,008184393	0,0257324	0,002522186	0,002587516	0,008494609	0,001689467	-0,02988417	2_4	Cats
33	0,8719721	0,000007122	0,01024129	0,078738	0,01010956	0,001056624	0,002756086	0,00564811	-0,08609377	2_5	Cats
33	0,8853432	0,000159804	0,000000537	0,122383	0,01507219	0,001307539	0,006037856	0,001910447	-0,1229296	2_6	Cats
33	0,8808173	0,000039267	0,002176248	0,08647081	0,004283937	0,001715409	0,003903728	0,000401204	-0,08884136	2_7	Cats
33	0,8459976	0,003311285	0,003610122	0,02325874	0,009544404	0,000517606	0,000121262	0,0208138	-0,02247822	2_8	Cats
33	0,4123074	0,000240034	0,3491627	0,000038139	0,000023244	0,000145088	0,000452985	0,000038669	-0,3489227	3_1	Horses
33	0,8375939	0,000587095	0,1181934	0,001470752	0,008353429	0,002113234	0,03342851	0,000305953	-0,1183369	3_2	Horses
33	0,5133261	0,02729881	0,01263624	0,001420314	0,01528374	0	0,000581089	0,03418265	0,01302934	3_3	Horses
33	0,32988	0,000168307	0,4652447	0,001652622	0,000177813	0,003168993	0,002389594	0,000231438	-0,4675021	3_4	Horses
33	0,5805373	0,05533319	0,02636201	0,02075987	0,003520559	0,1136065	0,001579823	0,000928429	-0,09084509	4_1	Pigs
33	0,5629542	0,005183246	0,004490849	0,005854599	0,009847085	0,000605955	0,00247351	0,005631622	-0,005149629	4_2	Pigs

33	0,5252612	0,000000005	0,09565109	0,005859563	0,00003484	0,0180886	0,006553246	0,000077878	-0,1094112	5_1	Sheep
33	0,8915612	0,001304393	0,05655043	0,003356362	0,000336234	0,000479525	0,01206187	0,000843582	-0,05870299	5_2	Sheep
**Participant 34**											
**Participant_ID**	**Neutral_Mean**	**Happy_Mean**	**Sad_Mean**	**Angry_Mean**	**Surprised_Mean**	**Scared_Mean**	**Disgusted_Mean**	**Contempt_Mean**	**Valence_Mean**	**Animal_Id**	**Animal**
34	0,3419881	0,004228618	0,1757275	0,01180746	0,007355466	0,2368076	0,007856862	0,001159303	-0,3996395	1_1	Dogs
34	0,3396981	0,06199542	0,05442652	0,02620935	0,01542378	0,280098	0,01435289	0,001092999	-0,2640427	1_2	Dogs
34	0,4563137	0,07864635	0,03669536	0,0124624	0,009036414	0,02724436	0,000242293	0,01069706	0,01102399	1_3	Dogs
34	0,5764438	0,001844098	0,02188463	0,02571531	0,005039189	0,08734416	0,001911394	0,000074391	-0,1185652	1_4	Dogs
34	0,416798	0	0,0781223	0,001724967	0,0690318	0,1259369	0,001257335	0,000018442	-0,1775502	1_5	Dogs
34	0,2921599	0,01406278	0	0,000091757	0,002909028	0,444378	0,001472103	0,000002964	-0,4303153	1_6	Dogs
34	0,3938924	0,000000157	0,05281905	0,06146129	0,000004993	0,2262072	0,004644516	0,000786626	-0,2898122	1_7	Dogs
34	0,3750607	0	0,1124008	0,1961886	0	0,00273265	0,01079045	0,000292729	-0,2534079	1_8	Dogs
34	0,2343941	0	0,2438409	0,1007978	0,000091713	0,2619827	0,000015848	0,000862361	-0,555347	1_9	Dogs
34	0,2934712	0	0,09033308	0,00776669	0,002258843	0,378332	0,000997065	0,000917102	-0,4680438	1_10	Dogs
34	0,2921683	0	0,001816138	0,0469971	0,002982033	0,4453162	0,003298478	0,000140126	-0,4809062	1_11	Dogs
34	0,3697076	0	0,002100413	0,1068167	0,000056134	0,2183089	0,01757417	0,000064899	-0,3005623	1_12	Dogs
34	0,6535068	0,04423978	0	0,000233162	0	0,03415665	0,01484045	0,000723748	-0,004596282	1_13	Dogs
34	0,4340603	0,000979381	0,37218	0,000680445	0,000048292	0,002229568	0,00164418	0,0046806	-0,3729514	2_1	Cats
34	0,2798548	0,001302248	0,6031678	0,00112734	0,000402722	0,00005593	0,001926043	0,000003692	-0,6020318	2_2	Cats
34	0,1953173	0	0,7545753	0,00459214	0,000030513	0,000725957	0,003127876	0,000017309	-0,7591296	2_3	Cats
34	0,4640386	0,07320745	0,05890508	0,000444266	0,002261468	0,04553059	0,007485451	0,000404865	-0,02060633	2_4	Cats
34	0,6163159	0	0,001739595	0,000001819	0,000000037	0,00009673	0,000000989	0,000002258	-0,001816862	2_5	Cats
34	0,2608865	0	0,5463333	0,000027842	0,000005669	0,000414665	0,009745303	0,00060202	-0,5463333	2_6	Cats
34	0,2851198	0	0,5256097	0,000019077	0,000966867	0,004661521	0,009094206	0,00034765	-0,5326015	2_7	Cats
34	0,1917502	0,000000001	0,7282436	0	0	0	0,000489431	0	-0,7282436	2_8	Cats
34	0,8670951	0,002952166	0,0555106	0,01732509	0,004423719	0,00092105	0,01820602	0,000360706	-0,06904465	3_1	Horses
34	0,5598844	0,000027111	0,05806378	0,01132727	0,000176866	0,06421627	0,0330485	0,000041149	-0,1247479	3_2	Horses
34	0,7574894	0,003437916	0,03928037	0,07740857	0,000652284	0,0504611	0,005273176	0,001251449	-0,121012	3_3	Horses
34	0,8983188	0,000229357	0,06657106	0,001488563	0,002071697	0,000360525	0,01673997	0,000209672	-0,06788972	3_4	Horses

34	0,401995	0,09380244	0,03127683	0,008367142	0,05473785	0,05196433	0,01739718	0,004452161	0,005171668	4_1	Pigs
34	0,530356	0,02942686	0,07885267	0,02237074	0,000392853	0,000975211	0,002599053	0,001719707	-0,05853497	4_2	Pigs
34	0,6718297	0,000301746	0,004003002	0,000515202	0,005171142	0,001098929	0,00143198	0,000004412	-0,004890657	5_1	Sheep
34	0,4091158	0,000030927	0,01742424	0,002459074	0,000360896	0,3653843	0,000586352	0,000174168	-0,3671394	5_2	Sheep
**Participant 35**											
**Participant_ID**	**Neutral_Mean**	**Happy_Mean**	**Sad_Mean**	**Angry_Mean**	**Surprised_Mean**	**Scared_Mean**	**Disgusted_Mean**	**Contempt_Mean**	**Valence_Mean**	**Animal_Id**	**Animal**
35	0,4314148	0,09154702	0,002638947	0,0104902	0,003792328	0,039238	0,03163552	0,004557069	0,03299825	1_1	Dogs
35	0,4533996	0,07150029	0,02999247	0,01124124	0,009246659	0,03678007	0,01102468	0,01154877	-0,003097283	1_2	Dogs
35	0,5245878	0,02597192	0,07371528	0,01662552	0,00031911	0,03900173	0,0100862	0,01135287	-0,07855769	1_3	Dogs
35	0,5403652	0,01262986	0,0207652	0,002989749	0,02367014	0,04988254	0,000803499	0,001223445	-0,05332286	1_4	Dogs
35	0,445521	0,01193806	0,04559675	0,0356078	0,03318596	0,05422075	0,002366393	0,01470886	-0,09777416	1_5	Dogs
35	0,4319134	0,06381962	0,07115857	0,002532844	0,02770485	0,02314495	0,01044767	0,002217475	-0,03059364	1_6	Dogs
35	0,4287273	0,08227608	0,01041453	0,00300045	0,02339793	0,05763431	0,000458659	0,000943817	0,0191912	1_7	Dogs
35	0,3788368	0,2393875	0,03425504	0,006969997	0,01442949	0,03662487	0,001968034	0,001263353	0,170221	1_8	Dogs
35	0,5227163	0,000000004	0,01242105	0,000650538	0,00510712	0,01479835	0,000109677	0,000000056	-0,02721411	1_9	Dogs
35	0,3965929	0,1606047	0,007372907	0,02032002	0,01740346	0,03455575	0,03860489	0,03960338	0,08429318	1_10	Dogs
35	0,3617985	0,2545729	0,03050549	0,01015241	0,00672408	0,05170159	0,00493616	0,001850202	0,180667	1_11	Dogs
35	0,4238415	0,05457306	0,02192299	0,001510524	0,02504229	0,07120147	0,01977183	0,005243527	-0,03828843	1_12	Dogs
35	0,4339873	0,09739343	0,01861607	0,02289318	0,0278741	0,02227256	0,0209651	0,003798764	0,03577451	1_13	Dogs
35	0,8680768	0,002412485	0,07764984	0,01107072	0,005973606	0,004712385	0,0232888	0,001290115	-0,08224332	2_1	Cats
35	0,7833899	0,01737585	0,08197165	0,01529942	0,02115806	0,001707047	0,01820494	0,001197055	-0,07948557	2_2	Cats
35	0,7905316	0,01591215	0,1253653	0,0103308	0,000942989	0,005041629	0,01866901	0,000144698	-0,1187158	2_3	Cats
35	0,8825895	0,002841613	0,02578284	0,01037855	0,008864718	0,01136018	0,01645452	0,001274346	-0,03506076	2_4	Cats
35	0,8008887	0,01193265	0,05119986	0,02713217	0,01137246	0,007076047	0,01956286	0,007516272	-0,06304378	2_5	Cats
35	0,8784981	0,007067158	0,02060487	0,01498314	0,005335758	0,00248445	0,02338182	0,000909911	-0,03534571	2_6	Cats
35	0,7561006	0,01458082	0,0972627	0,002102602	0,009909209	0,01319527	0,05777837	0,000042967	-0,09222857	2_7	Cats
35	0,8696024	0,000530837	0,000585879	0,007561978	0,03262322	0,000485293	0,003100344	0,00061926	-0,008879675	2_8	Cats
35	0,6215022	0,000000074	0,01192915	0,01456299	0,005326732	0,1025928	0,00489051	0,00003306	-0,1128567	3_1	Horses
35	0,4703824	0,005732794	0,07040356	0,0001484	0,02055844	0,000071676	0,03325538	0,004458264	-0,0775521	3_2	Horses

35	0,4631716	0,02416773	0,07355372	0,00000005	0,004682403	0,01664611	0,000000964	0,004641572	-0,0623805	3_3	Horses
35	0,3975881	0,000208178	0,002270021	0,00456822	0,000144424	0,3144184	0,004173255	0,001126145	-0,3192261	3_4	Horses
35	0,5011492	0,1141137	0,03444183	0,005110118	0,000067137	0,0266053	0,000086938	0,006045938	0,05969035	4_1	Pigs
35	0,4622013	0,02146557	0,05790107	0,000205204	0,000169383	0,04241316	0,001831532	0,008463815	-0,06915364	4_2	Pigs
35	0,5650474	0,05972485	0,04980489	0,01088154	0,000107402	0,01910841	0,00006152	0,000672392	0,002914786	5_1	Sheep
35	0,6503027	0,003131462	0,006127004	0,001256549	0,007576685	0,01106099	0,002524651	0,003678599	-0,01523655	5_2	Sheep
**Participant 36**											
**Participant_ID**	**Neutral_Mean**	**Happy_Mean**	**Sad_Mean**	**Angry_Mean**	**Surprised_Mean**	**Scared_Mean**	**Disgusted_Mean**	**Contempt_Mean**	**Valence_Mean**	**Animal_Id**	**Animal**
36	0,4998812	0,02191772	0,1311048	0,01370472	0,000556136	0,00547855	0,02626948	0,004095753	-0,1110279	1_1	Dogs
36	0,5487472	0,04044496	0,1076402	0,0135291	0,000776962	0,007557688	0,03469154	0,004844157	-0,07672993	1_2	Dogs
36	0,3059603	0,04042433	0,05089581	0,001067085	0,000000261	0,3581222	0,00011021	0,000359561	-0,3418612	1_3	Dogs
36	0,586553	0,02426444	0,1242879	0,05720011	0,001272332	0,0294914	0,000774965	0,001107994	-0,1395131	1_4	Dogs
36	0,5096158	0,04918957	0,02401348	0,01039188	0,000101873	0,06194119	0,00141016	0,007755528	-0,03170532	1_5	Dogs
36	0,5908378	0,02024227	0,04069649	0,1228022	0,000460631	0,03609788	0,003604765	0,003565452	-0,1317071	1_6	Dogs
36	0,5192626	0,004297402	0,2167937	0,008945124	0,003443713	0,004672826	0,01429059	0,02855486	-0,2168594	1_7	Dogs
36	0,6333196	0,000820422	0,04253876	0,02397573	0,000171778	0,00917022	0,000041418	0,002575313	-0,06498809	1_8	Dogs
36	0,5020432	0,00424109	0,2216516	0,07582381	0,000509397	0,004497026	0,007258837	0,00000039	-0,2810539	1_9	Dogs
36	0,3419104	0	0,5740847	0,006182812	0,000034774	0,001350994	0,007941279	0,000124714	-0,5740847	1_10	Dogs
36	0,506853	0	0,287995	0,000536412	0,000001985	0,002396539	0,000153308	0,000151254	-0,287995	1_11	Dogs
36	0,461893	0,000843864	0,3399478	0,01797253	0,01053725	0,01117696	0,01335438	0,001531816	-0,3479136	1_12	Dogs
36	0,4270684	0,000872705	0,4310882	0,001627055	0,000002941	0,000577737	0,006156648	0,000094482	-0,4302154	1_13	Dogs
36	0,4994674	0,006124235	0,02667881	0,02086954	0,008059695	0,07923528	0,007111662	0,00113617	-0,09401464	2_1	Cats
36	0,6059344	0,000385441	0,01617622	0,000917484	0,002130922	0,1066667	0,003332916	0,000522463	-0,108407	2_2	Cats
36	0,5693098	0	0,02453416	0,000017818	0,03869351	0,04856362	0,008042059	0	-0,06787272	2_3	Cats
36	0,4330633	0,000039708	0,135096	0,00431465	0,005483491	0,1207561	0,000551891	0,007177953	-0,2173618	2_4	Cats
36	0,5017555	0,00005944	0,1141357	0,004480934	0,02305301	0,06664997	0,000335162	0,001266549	-0,1258707	2_5	Cats
36	0,5109825	0	0,1395967	0,08628803	0,000026283	0,007750202	0,005218331	0,001446472	-0,1920925	2_6	Cats
36	0,5331086	0	0,1200535	0,01106149	0	0	0,000253463	0,00000832	-0,1200535	2_7	Cats
36	0,4110805	0,000164032	0,000000034	0,006124238	0,000059889	0,4232897	0,001885638	0,000073988	-0,4249251	2_8	Cats

36	0,4470057	0,002848309	0,03913466	0,04378388	0,03921523	0,03964004	0,003136977	0,012743	-0,09752303	3_1	Horses
36	0,4920216	0,01128831	0,2844638	0,01313828	0,000230958	0,002624215	0,01187702	0,003064771	-0,2855602	3_2	Horses
36	0,6507769	0,05269738	0,001905425	0,000043837	0,007426332	0,005514658	0,001544041	0,002918355	0,04454725	3_3	Horses
36	0,5396033	0,004135417	0,000965248	0,01274509	0,01284729	0,06037412	0,000011663	0,003221287	-0,0602845	3_4	Horses
36	0,4611436	0,001549851	0,1106292	0,000010375	0,000028643	0,02171895	0,01264431	0,001995088	-0,121737	4_1	Pigs
36	0,5770361	0,003087173	0,01760908	0,000492129	0,01933777	0,003145481	0,002964248	0,003100779	-0,01748814	4_2	Pigs
36	0,4905255	0,02067115	0,07835531	0,001554406	0,000482852	0,04917378	0,005378932	0,00483762	-0,0926227	5_1	Sheep
36	0,5253485	0,02034494	0,07222593	0,01959096	0,000563383	0,06597404	0,01921529	0,000746804	-0,1168056	5_2	Sheep
**Participant 37**											
**Participant_ID**	**Neutral_Mean**	**Happy_Mean**	**Sad_Mean**	**Angry_Mean**	**Surprised_Mean**	**Scared_Mean**	**Disgusted_Mean**	**Contempt_Mean**	**Valence_Mean**	**Animal_Id**	**Animal**
37	0,4120225	0,02750562	0,08202302	0,01377409	0,00042773	0,1528007	0,004426601	0,003073626	-0,209076	1_1	Dogs
37	0,3325789	0,1917266	0,03261719	0,000079585	0,003511937	0,2139903	0,001436009	0,00195055	-0,05167025	1_2	Dogs
37	0,526787	0,1645782	0,004497356	0,000134698	0,006927362	0,02558545	0,00019257	0,003763522	0,1345073	1_3	Dogs
37	0,4077637	0,000005515	0,06491075	0,00060453	0	0,1486674	0,000410908	0,000000006	-0,1791947	1_4	Dogs
37	0,2476123	0,06845137	0,07375932	0,000190018	0,01612806	0,3928582	0,003916733	0,002180052	-0,3692955	1_5	Dogs
37	0,3036404	0,07469119	0,0292482	0,000622238	0,01121786	0,3196188	0,001816609	0,006850265	-0,2718855	1_6	Dogs
37	0,3197338	0,008295122	0,086095	0,00717437	0,002854057	0,3213899	0,000217228	0,001420634	-0,3757116	1_7	Dogs
37	0,3299868	0,1005968	0,09064489	0,000568966	0,001518584	0,1963896	0,0003312	0,003823457	-0,1858641	1_8	Dogs
37	0,4532431	0,002512671	0,06643773	0,000000026	0,000000216	0,0504853	0,004005534	0,002512695	-0,09244937	1_9	Dogs
37	0,4121066	0	0,09418314	0	0,000030085	0,0923258	0,000412653	0,001075731	-0,1664973	1_10	Dogs
37	0,307967	0,07978335	0,04517012	0	0,000000749	0,312573	0,000122394	0,002356077	-0,2762288	1_11	Dogs
37	0,4418328	0,01437358	0,05776743	0	0	0,1332739	0,000028678	0,000072151	-0,1191803	1_12	Dogs
37	0,3462317	0,2049318	0,02399097	0,000936355	0,01009376	0,1775483	0,000387688	0,00205877	0,00358468	1_13	Dogs
37	0,4773783	0,05092663	0,000074391	0,000284041	0,03596009	0,02781365	0,0100281	0,001291839	0,01382851	2_1	Cats
37	0,3789412	0,06439353	0,1349618	0,04164816	0,02186633	0,05857819	0,07477513	0,0354157	-0,1263073	2_2	Cats
37	0,4787855	0,00649163	0,0185164	0,01175767	0,007578541	0,00214169	0,02575637	0,01746745	-0,04066635	2_3	Cats
37	0,4824334	0,000004149	0,0331615	0,02627005	0,03870511	0,04251325	0,01454546	0,004240097	-0,08848777	2_4	Cats
37	0,4714694	0,003288801	0,02162224	0,03542032	0,03827338	0,02208853	0,02524086	0,0330327	-0,07415112	2_5	Cats
37	0,5112857	0,000182409	0,05521726	0,000000005	0,03775478	0,000006504	0,000031876	0,02991273	-0,05506672	2_6	Cats

37	0,4937488	0,00386716	0,09395976	0,001021529	0,01724589	0,004616589	0,00393719	0,005251031	-0,09377645	2_7	Cats
37	0,4838389	0,000859717	0,01502745	0,02182771	0,07343468	0,01718088	0,000155425	0,002748765	-0,04188044	2_8	Cats
37	0,547682	0,003065058	0,226721	0,006134049	0,000135839	0,00299408	0,01238263	0,002605268	-0,2289041	3_1	Horses
37	0,3832168	0,01364771	0,08755514	0,008049536	0,000002998	0,1521098	0,002987866	0,007177295	-0,217963	3_2	Horses
37	0,6307222	0,1430113	0,04263661	0,007070634	0,002335117	0,001545948	0,01790415	0,000026147	0,08661022	3_3	Horses
37	0,6112509	0,003551803	0,07485715	0,006083946	0,00028262	0,0251141	0,00489825	0,004030492	-0,09457523	3_4	Horses
37	0,5014012	0,01976494	0,003246567	0,000001651	0,002458044	0,001475463	0,000134241	0,004400086	0,01552765	4_1	Pigs
37	0,7474211	0,04989576	0,0104146	0,000184172	0,000192594	0,001472038	0,001044765	0,000528666	0,03731454	4_2	Pigs
37	0,6323347	0,02065614	0,003947934	0,001044501	0,009478237	0,004616554	0,000916047	0,001389858	0,01262549	5_1	Sheep
37	0,2246082	0,01917766	0,01238273	0,000052869	0,00034365	0,5511166	0,001751514	0,00759856	-0,5379538	5_2	Sheep
**Participant 38**											
**Participant_ID**	**Neutral_Mean**	**Happy_Mean**	**Sad_Mean**	**Angry_Mean**	**Surprised_Mean**	**Scared_Mean**	**Disgusted_Mean**	**Contempt_Mean**	**Valence_Mean**	**Animal_Id**	**Animal**
38	0,7266304	0,007944646	0,02628416	0,002730446	0,001300266	0,01248788	0,0203162	0,000497675	-0,03539973	1_1	Dogs
38	0,5895355	0,0235078	0,08200315	0,00279647	0,001462634	0,04057427	0,04210066	0,000990261	-0,1068144	1_2	Dogs
38	0,7092273	0,03233467	0,01666362	0,01620243	0,003577645	0,000001368	0,01307022	0,004744275	-0,01089473	1_3	Dogs
38	0,6588978	0,001828983	0,06419579	0,01071712	0,004198077	0,03276923	0,007756587	0,000780036	-0,09876613	1_4	Dogs
38	0,5560996	0,08365053	0,02286506	0,000000469	0,001843873	0,02564286	0,005443434	0,001051268	0,03145451	1_5	Dogs
38	0,6700119	0	0,03497843	0,005157396	0,004274563	0,00398527	0,000836598	0,00005115	-0,0393766	1_6	Dogs
38	0,6211039	0,001276856	0,05315837	0,002054091	0,005875641	0,008778582	0,01034405	0,003077098	-0,06526983	1_7	Dogs
38	0,6119982	0,04768898	0,007649742	0,000980636	0,01150518	0,006998681	0,002146225	0,00200214	0,03152137	1_8	Dogs
38	0,6621221	0,02605081	0,07508473	0,003716297	0,005067384	0,01912645	0,004597959	0,03271918	-0,06750594	1_9	Dogs
38	0,6338591	0,004101482	0,06799985	0,004977545	0,003304619	0,0227269	0,00635909	0,003468462	-0,08763691	1_10	Dogs
38	0,6626225	0,000205698	0,04367579	0,005492008	0,002004875	0,01030497	0,01078874	0,000144741	-0,05555224	1_11	Dogs
38	0,635569	0,000009581	0,00001067	0,000035527	0,001409422	0,001074529	0,02250176	0,000046695	-0,02326244	1_12	Dogs
38	0,7138208	0,06885196	0,002204452	0,001121521	0,003459302	0,00290255	0,02985011	0,002023693	0,03739703	1_13	Dogs
38	0,461084	0,005750222	0,3053274	0,009527689	0,000329339	0,001899739	0,004663039	0,01204051	-0,3001975	2_1	Cats
38	0,5756471	0,02975131	0,1178404	0,003194353	0,000191857	0,01374241	0,01427975	0,007701541	-0,0972677	2_2	Cats
38	0,7026236	0,008710341	0,07598986	0,004722478	0,000037967	0,000917768	0,000422194	0,001405711	-0,06917673	2_3	Cats
38	0,6182218	0,001690435	0,1040182	0,04263783	0,00003321	0,01351444	0,000666069	0,007273614	-0,138397	2_4	Cats

38	0,4847719	0,008400733	0,1480224	0,01243466	0,000026508	0,02819259	0,009557329	0,004344174	-0,158762	2_5	Cats
38	0,632904	0,006219123	0,02082829	0,04028143	0,002723532	0,001976486	0,01685573	0,007194259	-0,05095216	2_6	Cats
38	0,4847376	0,1494033	0,04573689	0,01653581	0,001023949	0,02568131	0,009470991	0,006833794	0,0685509	2_7	Cats
38	0,5030577	0,003059394	0,2879663	0,001305397	0,000018089	0,004386014	0,000006717	0,002368463	-0,2849069	2_8	Cats
38	0,3808014	0,004400846	0,09708072	0,001951403	0,006918117	0,1664947	0,01003694	0,001404757	-0,2262657	3_1	Horses
38	0,5421858	0,00466075	0,002112978	0,000000005	0,02078432	0,004889608	0,004724887	0,001597985	-0,005260321	3_2	Horses
38	0,6541963	0,00351523	0,007556758	0,0021329	0,03921056	0,009767478	0,000412931	0,000467715	-0,01114377	3_3	Horses
38	0,5110577	0,002548029	0,05340151	0	0,000000476	0,01916774	0	0,005916765	-0,06800766	3_4	Horses
38	0,7924323	0,04749777	0,02340085	0,007510894	0,001587405	0,002006153	0,001071897	0,001072337	0,01701743	4_1	Pigs
38	0,4516881	0,1112717	0,03380708	0,02534399	0,0645094	0,0307645	0,01982882	0,005932306	0,03709865	4_2	Pigs
38	0,8342485	0,06063509	0,008885328	0,002303727	0,01187082	0,000342851	0,007210928	0,000425347	0,04616061	5_1	Sheep
38	0,6534234	0,01122188	0,09152808	0,004468034	0,003553761	0,000043722	0,009832219	0,009972218	-0,08037785	5_2	Sheep
**Participant 39**											
**Participant_ID**	**Neutral_Mean**	**Happy_Mean**	**Sad_Mean**	**Angry_Mean**	**Surprised_Mean**	**Scared_Mean**	**Disgusted_Mean**	**Contempt_Mean**	**Valence_Mean**	**Animal_Id**	**Animal**
39	0,6773689	0,001740692	0,02092797	0,01359775	0,000942989	0,01784197	0,05586376	0,001241316	-0,07988189	1_1	Dogs
39	0,8228242	0,02323765	0,001608994	0,03995652	0,007221539	0,0152285	0,000536011	0,000764361	-0,0305543	1_2	Dogs
39	0,9208708	0	0,000427086	0,000221367	0	0	0,000576204	0	-0,000576215	1_3	Dogs
39	0,6133956	0,0507509	0,06816193	0,03492367	0,000340217	0	0,05333436	0,003086281	-0,0850925	1_4	Dogs
39	0,801437	0,03349653	0,02855876	0,0025203	0,000953142	0,000628588	0,02321887	0,000450061	-0,01015063	1_5	Dogs
39	0,7052159	0,01968995	0,02144185	0,01533563	0,001641641	0,000142669	0,02685454	0,000055622	-0,03172033	1_6	Dogs
39	0,7593748	0,06499314	0,02385073	0,000001721	0,006171534	0,001809031	0,000066258	0,006989804	0,03968257	1_7	Dogs
39	0,6422693	0,003856393	0,07004119	0,000377861	0,000779833	0,000004102	0,001179768	0,000836993	-0,06760839	1_8	Dogs
39	0,6217123	0,0166716	0,02763577	0,000000837	0,004866885	0,000206809	0,002883764	0,000400311	-0,01403337	1_9	Dogs
39	0,6984777	0,02188415	0,01091451	0	0,004703975	0,000001329	0,000000002	0,00054584	0,01096965	1_10	Dogs
39	0,6879722	0,02938093	0,02098364	0,02042542	0,007578833	0,00064977	0,03966323	0,004175442	-0,02973638	1_11	Dogs
39	0,8851025	0,008168753	0,000069096	0,009654325	0,01765157	0,000034187	0,000412469	0,000171552	-0,001872277	1_12	Dogs
39	0,9546047	0,003351136	0,006931106	0,000000456	0,000153634	0,000000006	0,000000043	0,001368341	-0,003580042	1_13	Dogs
39	0,4295429	0,01611554	0,1080748	0,008988786	0,004059873	0,09228504	0,002601704	0,000811929	-0,1793208	2_1	Cats
39	0,4064732	0,05152932	0,02592783	0,002116605	0,001972877	0,1665517	0,001186327	0,003430102	-0,1400816	2_2	Cats

39	0,3470212	0,001164341	0,1758184	0,004180991	0,000312296	0,1619557	0,000716466	0,01717193	-0,3188218	2_3	Cats
39	0,3791493	0,01138182	0,1133239	0,000087437	0,000000838	0,1456602	0,000032345	0,01372792	-0,2393115	2_4	Cats
39	0,464095	0,009150166	0,06442393	0,000661458	0,002068081	0,01547058	0,00213821	0,000749233	-0,06899567	2_5	Cats
39	0,5438218	0,01363784	0,02613431	0,000160084	0,01857929	0,01335871	0,004292242	0,000891646	-0,02354478	2_6	Cats
39	0,4881959	0,002499925	0,03374225	0	0,000004039	0,02852688	0,000000541	0,002760238	-0,05800158	2_7	Cats
39	0,4617199	0	0,04488312	0	0	0,04233366	0,001265812	0,001691005	-0,08639156	2_8	Cats
39	0,5074298	0,1290358	0,05762817	0,003358713	0,02333887	0,001274229	0,004223449	0,003960075	0,07079887	3_1	Horses
39	0,839551	0,04169571	0,01276387	0,0004698	0,001595833	0,000098507	0,001151292	0,002237119	0,02866839	3_2	Horses
39	0,9719663	0,000008885	0	0,003824575	0,004810772	0	0,000522604	0,001117559	-0,004338184	3_3	Horses
39	0,5397959	0,09030402	0,005824425	0,000217857	0,04749119	0,000457789	0,00786835	0,001744628	0,07898328	3_4	Horses
39	0,4277356	0,1802154	0,02078389	0,011747	0,06351621	0,03104106	0,01636874	0,001516438	0,1237557	4_1	Pigs
39	0,4639194	0,1614905	0,004123963	0,03403158	0,02583974	0	0,005505079	0,005890006	0,1268334	4_2	Pigs
39	0,5485014	0,07329124	0,02894582	0,01012947	0,02151304	0,03847783	0,002399332	0,004435488	0,007286495	5_1	Sheep
39	0,8493183	0,01674578	0,01305027	0,009463216	0	0,000936692	0,000695885	0,000126882	-0,002032043	5_2	Sheep
**Participant 40**											
**Participant_ID**	**Neutral_Mean**	**Happy_Mean**	**Sad_Mean**	**Angry_Mean**	**Surprised_Mean**	**Scared_Mean**	**Disgusted_Mean**	**Contempt_Mean**	**Valence_Mean**	**Animal_Id**	**Animal**
40	0,6375163	0,008888939	0,00254793	0,000603488	0,03165674	0,02580571	0,002664104	0,000072266	-0,02185494	1_1	Dogs
40	0,6066949	0,000008284	0,000186946	0,000092176	0,04656377	0,05834195	0,00000672	0,001847574	-0,05844914	1_2	Dogs
40	0,3248785	0,4666235	0	0,000000005	0	0,000211642	0,002857808	0,005631087	0,4637658	1_3	Dogs
40	0,3382362	0,1567663	0,01162849	0,001101277	0,05160837	0,2520562	0,00145966	0,000445137	-0,1072751	1_4	Dogs
40	0,3858203	0,4119359	0,002677246	0,003275845	0,006693697	0,005323953	0,02378159	0,006362676	0,3827626	1_5	Dogs
40	0,4127228	0,1862008	0,000308237	0,001466904	0,000757266	0,1525463	0,03543895	0,001278242	-0,001244019	1_6	Dogs
40	0,4815333	0,0607147	0,0102975	0,001613778	0,1062582	0,02401819	0,02708885	0,000046012	0,008290959	1_7	Dogs
40	0,5872332	0,06938676	0,006194431	0,01033435	0,03238441	0,005504054	0,01094658	0,001876078	0,04351675	1_8	Dogs
40	0,7010623	0,007924646	0,006147623	0,000129873	0,004393182	0,0232613	0,02304759	0,000286438	-0,03836684	1_9	Dogs
40	0,6417938	0,01470088	0,01774225	0,000088942	0,004416397	0,01268183	0,02238332	0,000883783	-0,02814306	1_10	Dogs
40	0,69608	0,04232052	0,0727231	0,005551518	0,02722244	0,002908506	0,04701417	0,007730934	-0,06173406	1_11	Dogs
40	0,740796	0,04200838	0,005375667	0,01513482	0,007380914	0	0,004011707	0,001133194	0,02530402	1_12	Dogs
40	0,7348077	0,03211137	0,01266968	0,001514976	0,02208442	0,001199845	0,05265409	0,00098117	-0,02172979	1_13	Dogs

40	0,5461824	0,01132177	0,03634815	0,008712004	0,007903388	0,01535719	0,03434293	0,00518852	-0,04767682	2_1	Cats
40	0,6890047	0,000224399	0,03086809	0,004069693	0,00283699	0,006427365	0,001687826	0,000954242	-0,03577506	2_2	Cats
40	0,6601943	0	0,03645831	0,002988108	0,005389082	0,01044322	0,03000863	0,001042963	-0,04864779	2_3	Cats
40	0,6492379	0,002613661	0,03482094	0,01286341	0,01095181	0,004061782	0,005513692	0,005583751	-0,04302202	2_4	Cats
40	0,8097876	0,000728215	0,03275608	0,001439656	0,006372178	0,004303182	0,002586658	0,002554422	-0,03566181	2_5	Cats
40	0,6196817	0,004418033	0,05356539	0,001733334	0,0081129	0,001558847	0,000738624	0,01070902	-0,04932753	2_6	Cats
40	0,6467386	0,007449502	0,0187999	0,003309448	0,008308376	0,00341625	0,002217415	0,009588392	-0,01464442	2_7	Cats
40	0,7096313	0,000342975	0,02039715	0,002690989	0,000354447	0,000634767	0,005610829	0,000382218	-0,02496521	2_8	Cats
40	0,8143707	0,05457823	0,008734528	0,003404901	0,000728346	0,000755511	0,006264039	0,001101134	0,04152232	3_1	Horses
40	0,5572495	0,05838479	0,1032566	0,02183072	0,04214877	0,02211745	0,003517715	0,001665167	-0,06222599	3_2	Horses
40	0,6987239	0,002787839	0,000183513	0,004372655	0,00001407	0	0,1418184	0,000108008	-0,1399785	3_3	Horses
40	0,7931204	0,01400735	0,003490054	0,01069933	0,005341214	0,000070114	0,004772151	0,00010274	-0,004130933	3_4	Horses
40	0,6462122	0,03196567	0,005818506	0,02495195	0,00524212	0,01510181	0,009827371	0,001682174	-0,01535272	4_1	Pigs
40	0,4949099	0,258261	0,000039853	0,01499909	0,03293375	0,002427884	0,06441043	0,002003438	0,183545	4_2	Pigs
40	0,7925392	0,000000311	0,02985214	0,01452997	0,001111656	0,000032781	0,03061996	0,007957295	-0,03453348	5_1	Sheep
40	0,5706675	0,09316575	0,05253693	0,002793321	0,02998449	0,1298346	0,00711142	0,01236819	-0,0845288	5_2	Sheep
**Participant 41**											
**Participant_ID**	**Neutral_Mean**	**Happy_Mean**	**Sad_Mean**	**Angry_Mean**	**Surprised_Mean**	**Scared_Mean**	**Disgusted_Mean**	**Contempt_Mean**	**Valence_Mean**	**Animal_Id**	**Animal**
41	0,9661516	0	0,01262056	0,009870111	0,02883828	0	0	0,01045051	-0,01926084	1_1	Dogs
41	0,9023346	0	0,000014939	0,02188338	0	0	0,01088049	0,000006526	-0,02188338	1_2	Dogs
41	0,8429505	0,002078482	0,03336763	0,01039208	0,03188198	0,01229115	0,06733057	0,01100224	-0,07514815	1_3	Dogs
41	0,8592423	0	0,01163139	0,002452509	0	0,007943171	0,001189108	0,000107552	-0,0202193	1_4	Dogs
41	0,9197713	0	0,002710933	0,0054452	0,01611543	0	0	0,000051534	-0,005549914	1_5	Dogs
41	0,9571779	0	0,001828614	0,002219042	0,005118217	0	0	0,003716213	-0,003548496	1_6	Dogs
41	0,9468292	0,000114727	0,000276524	0,005379238	0,002149205	0	0	0,000000102	-0,005528635	1_7	Dogs
41	0,9508986	0,000010747	0,002158592	0,01345359	0,00247582	0,000139315	0,00001575	0,000772493	-0,0147667	1_8	Dogs
41	0,8693788	0,000038422	0,03486582	0,001186876	0,000000036	0,000000375	0,001450052	0,000000861	-0,03687845	1_9	Dogs
41	0,8677388	0,000030766	0,05819846	0,000184441	0,007226388	0,002231183	0,002177527	0	-0,05835199	1_10	Dogs
41	0,924946	0	0,02164159	0,00233155	0,000977087	0,000001422	0	0	-0,0233456	1_11	Dogs

41	0,917996	0,000066166	0,02564589	0,029037	0,006608895	0,000024335	0,001513803	0,000510917	-0,03659423	1_12	Dogs
41	0,9625083	0,00014286	0	0,01489493	0,01320951	0,000000051	0,000250256	0,003244784	-0,01475207	1_13	Dogs
41	0,8161052	0,03523975	0,01892089	0,011343	0,000725742	0,000650985	0,02783422	0,000889692	-0,01170627	2_1	Cats
41	0,9246541	0,003476591	0,01165347	0,008473266	0,003660055	0,000171179	0,004430176	0,001017887	-0,01923224	2_2	Cats
41	0,768683	0,03570953	0,00853239	0,0431072	0,002082026	0,001550579	0,04000872	0,001095437	-0,03643886	2_3	Cats
41	0,7778118	0,02077656	0,04315554	0,006212174	0,001076695	0,000270928	0,002539072	0,00045855	-0,02797721	2_4	Cats
41	0,9163533	0,009224425	0,01394884	0,01723509	0,001468325	0,000411035	0	0,001504623	-0,02010353	2_5	Cats
41	0,8435377	0,00546666	0,02280992	0,008281949	0,002130279	0,000287508	0,007596318	0,001969492	-0,03204355	2_6	Cats
41	0,6672322	0,1009599	0,03540003	0,01589249	0,002394638	0,001235985	0,02505172	0,000523377	0,04993483	2_7	Cats
41	0,8978068	0,03679561	0,001758052	0	0,002592267	0,000051335	0,006984028	0,001021073	0,02835818	2_8	Cats
41	0,5287573	0,09367062	0,02835146	0,0131244	0,03848177	0,006210228	0,004767701	0,003912815	0,05354035	3_1	Horses
41	0,9213998	0,00006753	0,0602787	0,01103806	0,001527919	0	0,003006922	0,000328167	-0,07411467	3_2	Horses
41	0,8504564	0,02142113	0,0155066	0,000560806	0,006038963	0,0313051	0,006234629	0,002120251	-0,02492259	3_3	Horses
41	0,735751	0,006386634	0,007700879	0,000003004	0,01065486	0,02441166	0,001134006	0,00521197	-0,02195941	3_4	Horses
41	0,3441806	0,3784509	0,000536477	0,006972777	0,008975468	0,004352298	0,03522758	0,000942601	0,3378375	4_1	Pigs
41	0,6756392	0,01474854	0,02372901	0,01281663	0,04346627	0,000047616	0,05677487	0,01509169	-0,05438044	4_2	Pigs
41	0,7331547	0,002593021	0,00571215	0,003817182	0,000031719	0,006602162	0,03635022	0,006867802	-0,0400225	5_1	Sheep
41	0,9054837	0,000667731	0,01244596	0,01482233	0,001856702	0,000220864	0,000178689	0,000111775	-0,02660197	5_2	Sheep
**Participant 42**											
**Participant_ID**	**Neutral_Mean**	**Happy_Mean**	**Sad_Mean**	**Angry_Mean**	**Surprised_Mean**	**Scared_Mean**	**Disgusted_Mean**	**Contempt_Mean**	**Valence_Mean**	**Animal_Id**	**Animal**
42	0,6923997	0,001270846	0,006014684	0,03217321	0,000000002	0,000000001	0,05666899	0,000919722	-0,08642989	1_1	Dogs
42	0,7533838	0,008193222	0,01301377	0,00008425	0	0,001090266	0,04965159	0,0023044	-0,04407737	1_2	Dogs
42	0,4926443	0,000000563	0,002695011	0,004048433	0,05146931	0,00812453	0,000137946	0,000063203	-0,008252039	1_3	Dogs
42	0,5668992	0,01068678	0,01309577	0	0	0	0,1951331	0,000114577	-0,1844462	1_4	Dogs
42	0,651553	0	0,01303648	0,02842363	0	0	0,04607156	0,000099438	-0,07160726	1_5	Dogs
42	0,5577777	0,09446807	0,000000863	0,000001383	0,00451023	0	0,01729801	0	0,07717006	1_6	Dogs
42	0,6857081	0,05889529	0,000000001	0,006643858	0,000825021	0,000902681	0,000039851	0,001946277	0,0521434	1_7	Dogs
42	0,7453409	0	0,000663573	0,0636525	0,002443966	0,00207201	0,01008034	0,001034405	-0,06384159	1_8	Dogs
42	0,5787196	0	0,02433051	0,05106381	0,000021257	0,000029562	0,01930186	0,001988697	-0,06996483	1_9	Dogs

42	0,6637097	0	0,02922721	0,05684796	0,000166605	0	0,03131096	0,004064153	-0,0877747	1_10	Dogs
42	0,5906602	0	0,0124858	0,05947727	0,000026769	0	0,03209006	0,000046786	-0,09034809	1_11	Dogs
42	0,4837009	0	0,03401581	0,04708935	0	0	0,05390104	0,000101361	-0,1040465	1_12	Dogs
42	0,5063311	0,07416453	0,01271841	0,06653858	0,00011942	0	0,0882852	0,000899226	-0,07297791	1_13	Dogs
42	0,6232982	0,02877071	0,01090152	0,0242543	0,04532237	0,00598744	0,0189312	0,002042267	-0,01583897	2_1	Cats
42	0,5418406	0,1025482	0,05322956	0,00056398	0,02416874	0,000247207	0,02026812	0,003937005	0,03621378	2_2	Cats
42	0,6794257	0,01889406	0,005336022	0,002851164	0,01016388	0,004719719	0,000561161	0,004055194	0,007027978	2_3	Cats
42	0,5224063	0,1537592	0,002677265	0,007481386	0,02265701	0,00785871	0,02054626	0,01128418	0,127432	2_4	Cats
42	0,6004227	0,1137641	0,01851674	0,000588932	0,01306794	0,00301737	0,004250855	0,009010973	0,09262998	2_5	Cats
42	0,4642929	0,1978285	0,06868012	0,0296876	0,04338956	0,003140463	0,006038006	0,006482849	0,1190729	2_6	Cats
42	0,4678259	0,08424798	0,09764181	0,01529219	0,05846256	0,01062304	0,01307387	0,008879856	-0,02322901	2_7	Cats
42	0,4737318	0,1514807	0,09538401	0,02038352	0,01315168	0,02145586	0,01242617	0,008370254	0,0353996	2_8	Cats
42	0,5562099	0,004400846	0,09708072	0,001951403	0,006918117	0,1664947	0,01003694	0,001404757	-0,2262657	3_1	Horses
42	0,6240586	0	0,05085605	0,03151803	0,01223618	0,000001646	0,005921386	0,000207225	-0,07407705	3_2	Horses
42	0,8832483	0,01450025	0,000939372	0	0,001222511	0,004650216	0,004099933	0,005600113	0,009392049	3_3	Horses
42	0,9537741	0,000000023	0	0,003634993	0,000199291	0	0,009911082	0,00156823	-0,01354406	3_4	Horses
42	0,646454	0,1602235	0,01033024	0,001586123	0,008294354	0,005489878	0,01878133	0,02047476	0,1288702	4_1	Pigs
42	0,2819321	0,6316614	0,000044688	0,02750966	0,008567697	0,00044853	0,000014833	0,002422032	0,6041517	4_2	Pigs
42	0,8320255	0,001770814	0,06132456	0,000602613	0,02356358	0,01068305	0,01609494	0,006628588	-0,06297204	5_1	Sheep
42	0,7053549	0	0,00815582	0,02520973	0,000086397	0	0,006022257	0,001492296	-0,0276302	5_2	Sheep
**Participant 43**											
**Participant_ID**	**Neutral_Mean**	**Happy_Mean**	**Sad_Mean**	**Angry_Mean**	**Surprised_Mean**	**Scared_Mean**	**Disgusted_Mean**	**Contempt_Mean**	**Valence_Mean**	**Animal_Id**	**Animal**
43	0,870079	0,008437376	0,04483892	0,01335839	0,02454746	0,004096273	0,06459156	0,01701937	-0,0838869	1_1	Dogs
43	0,8430713	0,000948917	0,09489036	0,003385194	0,009603183	0,001509817	0,07753098	0,0060624	-0,1162367	1_2	Dogs
43	0,3517778	0,3100983	0,01894906	0	0,02028196	0,00012644	0,005793157	0,004048301	0,2909853	1_3	Dogs
43	0,8764474	0,01696005	0,037643	0,002319457	0,01217521	0,01966557	0,01410268	0,002685393	-0,04660895	1_4	Dogs
43	0,8735449	0,000990988	0,02266571	0,009305792	0,003637872	0,009253335	0,05287587	0,000011935	-0,06612863	1_5	Dogs
43	0,8708439	0,006994939	0,007425783	0,01531059	0,01055408	0,006390464	0,01077062	0,004248511	-0,02307473	1_6	Dogs
43	0,8615445	0,03236477	0,01624951	0,001438905	0,00522462	0,000001563	0,01032174	0,007294406	0,01594653	1_7	Dogs

43	0,9374861	0,00033676	0,002490539	0,001094138	0,02109486	0	0,004135089	0,004076179	-0,006272632	1_8	Dogs
43	0,9311745	0,000004733	0,002629112	0,003306672	0,001813205	0,000518342	0,01299699	0,000228029	-0,01369893	1_9	Dogs
43	0,8949643	0	0,000000403	0,000778198	0,000398532	0,00971208	0,02663792	0,00025158	-0,03002538	1_10	Dogs
43	0,8603889	0,0119588	0,04172326	0,000432971	0,00056138	0,002575464	0,04965293	0,00072483	-0,06569505	1_11	Dogs
43	0,914297	0,000956459	0,0104397	0,000342918	0,00183193	0,00232378	0,02168148	0,000850591	-0,02417344	1_12	Dogs
43	0,8086299	0,000089738	0,01197217	0,01120612	0,000060327	0,000536631	0,1092162	0,00002477	-0,1093361	1_13	Dogs
43	0,924053	0,000001298	0,01803819	0,004782011	0,000342534	0,001163019	0,01279322	0,000403651	-0,02989171	2_1	Cats
43	0,8691293	0	0,03618176	0,001998646	0,000874608	0,01333212	0,08033367	0,000053994	-0,0909394	2_2	Cats
43	0,8285231	0,000028137	0,003215622	0,02398844	0,003340595	0,01584669	0,02480513	0,000008287	-0,05177224	2_3	Cats
43	0,8411456	0,00000001	0,01961959	0,007085536	0,005194207	0,003666847	0,000000132	0,000869098	-0,02548541	2_4	Cats
43	0,9167149	0,000404201	0,02838493	0,000488388	0,020324	0,000579194	0	0,000004105	-0,02834021	2_5	Cats
43	0,9504866	0	0,01346502	0,007052152	0,000485337	0	0	0,000461894	-0,02013888	2_6	Cats
43	0,8014577	0	0,09662763	0,009845795	0,000010103	0	0	0,00013304	-0,09954195	2_7	Cats
43	0,9078969	0	0,07709135	0,000019422	0	0	0	0	-0,07709135	2_8	Cats
43	0,6289405	0,001288362	0,0256945	0,1125584	0,000394303	0,001695424	0,01169405	0	-0,1327337	3_1	Horses
43	0,7366905	0,008246417	0,01016524	0,000909725	0,004426188	0,007226814	0,04877407	0,01586022	-0,05089411	3_2	Horses
43	0,7748238	0,001657231	0,000014281	0,000000006	0,0194476	0,004881817	0,000000007	0,01770474	-0,003224587	3_3	Horses
43	0,7681859	0,007695788	0,00043765	0,009032539	0,000070373	0	0,03049509	0,007214777	-0,02637208	3_4	Horses
43	0,6286585	0,004905628	0,06207374	0,1230737	0,00681014	0,007199959	0,02009805	0,01721425	-0,134416	4_1	Pigs
43	0,2879355	0,4275373	0,001651993	0,002509637	0,004497338	0,000016057	0,000720745	0,01533095	0,4248852	4_2	Pigs
43	0,8584481	0,001620125	0,007655025	0,001345393	0,01766943	0,00847003	0,005472667	0,004967502	-0,01357982	5_1	Sheep
43	0,8278096	0,02232276	0,02197482	0,006599025	0,006344367	0,006681784	0,06504629	0,01181871	-0,05934992	5_2	Sheep
**Participant 44**											
**Participant_ID**	**Neutral_Mean**	**Happy_Mean**	**Sad_Mean**	**Angry_Mean**	**Surprised_Mean**	**Scared_Mean**	**Disgusted_Mean**	**Contempt_Mean**	**Valence_Mean**	**Animal_Id**	**Animal**
44	0,5998149	0,00007074	0,005765817	0,02371093	0,02830548	0,024612	0,01133925	0,002026538	-0,03187439	1_1	Dogs
44	0,5014191	0,000002814	0,001468536	0,02023227	0,07180279	0,0178841	0,000689392	0,000315859	-0,02022945	1_2	Dogs
44	0,8789653	0,02587309	0,001049196	0,0325469	0,000201272	0,001293105	0,01978674	0,0148021	-0,006673818	1_3	Dogs
44	0,4995978	0,000000146	0,01202797	0,001049946	0,01444399	0,04227715	0,000035776	0,000016391	-0,04227701	1_4	Dogs
44	0,5083812	0,000000048	0,01215228	0,000346814	0,004771075	0,08099189	0,000011817	0,000005414	-0,08099183	1_5	Dogs

44	0,5200439	0,00009444	0,01004564	0,000090158	0,001240299	0,1265277	0,000003072	0,000001408	-0,1264332	1_6	Dogs
44	0,503332	0,001499215	0,003793077	0,000018426	0,000253485	0,1619477	0,000000628	0,000000288	-0,1604484	1_7	Dogs
44	0,4641268	0,002866456	0,01551803	0,000004415	0,000060734	0,1732146	0,00000015	0,000000069	-0,1703481	1_8	Dogs
44	0,4596816	0,003375174	0,07149827	0,000001442	0,000019834	0,1736535	0,000000049	0,000000023	-0,1702783	1_9	Dogs
44	0,4441916	0,004687813	0,0623553	0,00000056	0,0000077	0,1997617	0,000000019	0,000000009	-0,1950739	1_10	Dogs
44	0,4514883	0,004024276	0,05016075	0,000000221	0,000003037	0,1464608	0,01536357	0,000000003	-0,1424366	1_11	Dogs
44	0,4626621	0,003526974	0,04765731	0,00000007	0,000000967	0,1111725	0,01358142	0,000000001	-0,1076455	1_12	Dogs
44	0,4622215	0,002726272	0,02401499	0,004572801	0,000000355	0,1208303	0,005184102	0	-0,118104	1_13	Dogs
44	0,5271444	0,005946626	0,000020569	0,1023889	0,03014263	0,00334604	0,01473574	0,000315028	-0,1086553	2_1	Cats
44	0,6748083	0,0444295	0,002780501	0,02733077	0,02308119	0,001935685	0,08299775	0,000515984	-0,06334837	2_2	Cats
44	0,7187244	0,01366488	0,000761544	0,02284687	0,01447701	0,003927251	0,03060618	0,000930141	-0,03385078	2_3	Cats
44	0,8205107	0,03447834	0,000175504	0,029333	0,000129131	0,01052667	0,01126805	0,000640659	-0,009760868	2_4	Cats
44	0,7796218	0,008696534	0,000012673	0,06436215	0,003082691	0,005562402	0,02891196	0,00121611	-0,081628	2_5	Cats
44	0,8639113	0,01014991	0,001075953	0,07153577	0,005050942	0,009758526	0,02977998	0,000748817	-0,08496747	2_6	Cats
44	0,6485458	0,000000049	0,02239628	0,09316716	0,000191583	0,007517846	0,002498666	0,000323141	-0,1034608	2_7	Cats
44	0,5556628	0	0,02295197	0,1174624	0	0,001899725	0	0,000357892	-0,132018	2_8	Cats
44	0,7327055	0,004187901	0,02374953	0,00124303	0,0113494	0,002607431	0,06247269	0,02758776	-0,06168327	3_1	Horses
44	0,7403915	0,06992791	0,00674206	0	0,008510141	0,007649238	0,02869583	0,01104062	0,04123208	3_2	Horses
44	0,590933	0,000582962	0,02615371	0,009031864	0,000000878	0,03403991	0,002698515	0,0147005	-0,0396864	3_3	Horses
44	0,8234845	0,000065964	0,01652316	0,001095791	0,003143284	0,01980326	0,02877643	0,002537379	-0,04487857	3_4	Horses
44	0,3848997	0,2304216	0,006703324	0,002119958	0,000715012	0,000349937	0,001954255	0,003133424	0,2236376	4_1	Pigs
44	0,8808704	0,000853425	0,05052973	0,002852897	0,1222464	0	0,000047606	0,001318133	-0,0496763	4_2	Pigs
44	0,8360922	0	0,02832437	0,01426957	0,000008338	0,000001576	0,000807593	0,000057234	-0,03463931	5_1	Sheep
44	0,7995803	0,001433117	0,03265708	0,003634446	0,001320359	0,006788636	0,009743797	0,000969609	-0,03481792	5_2	Sheep
**Participant 45**											
**Participant_ID**	**Neutral_Mean**	**Happy_Mean**	**Sad_Mean**	**Angry_Mean**	**Surprised_Mean**	**Scared_Mean**	**Disgusted_Mean**	**Contempt_Mean**	**Valence_Mean**	**Animal_Id**	**Animal**
45	0,4424161	0,1079113	0,000616247	0	0,000000226	0,000002736	0,02430948	0,000844808	0,0836018	1_1	Dogs
45	0,1753653	0,6481202	0,000019046	0	0,001619342	0,000000076	0,007048517	0,000491359	0,6410717	1_2	Dogs
45	0,5094852	0,000004822	0,2487836	0,00022335	0,000062688	0,0001012	0,000216089	0,000028994	-0,2487788	1_3	Dogs

45	0,3469769	0,4216268	0,001442643	0	0,002585286	0,000240269	0,000357746	0,008367841	0,4201841	1_4	Dogs
45	0,3152108	0,4705173	0,000246857	0	0,000442379	0,000095887	0,000061215	0,005930364	0,4702705	1_5	Dogs
45	0,3468949	0,3509203	0,009798757	0,01317149	0,003715446	0,000264206	0,001759063	0,004253053	0,3283149	1_6	Dogs
45	0,4816491	0,08800434	0,000772226	0,04532399	0,001716134	0,000824033	0,000317671	0,003158675	0,04268034	1_7	Dogs
45	0,5104327	0,02981871	0,000255037	0,01660617	0,000567693	0,000916268	0,000104915	0,001117697	0,01321253	1_8	Dogs
45	0,5035737	0,03494174	0,000088194	0,005742537	0,000198564	0,000561176	0,00003628	0,000566822	0,02919921	1_9	Dogs
45	0,4426092	0,1433017	0,000027347	0,001780622	0,000107178	0,000211093	0,00001125	0,000718454	0,1415211	1_10	Dogs
45	0,497869	0,08751295	0,000009758	0,000635358	0,000049172	0,000107768	0,000004014	0,00317878	0,08687759	1_11	Dogs
45	0,5355127	0,03511046	0,000002663	0,000173419	0,000013421	0,000031768	0,000001096	0,002121538	0,03493704	1_12	Dogs
45	0,5020772	0,06167651	0,001066563	0,000041138	0,000003184	0,000012959	0,00000026	0,000559488	0,06057917	1_13	Dogs
45	0,850086	0,003202807	0,01356743	0,009854307	0,01716475	0,006356148	0,09048064	0,001002871	-0,09301606	2_1	Cats
45	0,8382295	0,00045881	0,01903565	0,003921792	0,001638878	0,01021514	0,01894455	0,003921001	-0,03685415	2_2	Cats
45	0,8875446	0,005747438	0,01538461	0,000002948	0,002819736	0,00365118	0,03078813	0,001451887	-0,03149843	2_3	Cats
45	0,9102771	0,00618803	0,009765885	0,001060452	0,001629517	0,000328623	0,00487388	0,002935263	-0,005463729	2_4	Cats
45	0,8821499	0,000562842	0,05891195	0,00134903	0,02238281	0,000207826	0,01539973	0,000452121	-0,06023956	2_5	Cats
45	0,9228994	0,000208887	0,005930291	0,000682846	0,00072229	0,002614748	0,002795287	0,004787777	-0,008055476	2_6	Cats
45	0,8160439	0,01697915	0,03549055	0,008963413	0,02002013	0,003605926	0,005997408	0,003755857	-0,02034209	2_7	Cats
45	0,7822872	0,003286921	0,05533499	0,000435266	0,009600461	0,007400432	0,05545663	0,003428818	-0,08440766	2_8	Cats
45	0,8783004	0,005482832	0,01363154	0	0,004280273	0,003595599	0,01569761	0,003908223	-0,01349852	3_1	Horses
45	0,8964978	0,000069997	0,000011221	0,000001204	0,03441506	0,02857861	0,000001475	0,009176034	-0,02850861	3_2	Horses
45	0,8082268	0,000009127	0,000000132	0,00003094	0,07435543	0,001803495	0,000000078	0,000006554	-0,001794368	3_3	Horses
45	0,9242231	0,003394661	0,000219917	0	0,000286203	0,006755457	0,000959838	0,005607245	-0,003360796	3_4	Horses
45	0,8182834	0,0110843	0,01192123	0,04088124	0,02991867	0,003693588	0,03468317	0,001685707	-0,06539005	4_1	Pigs
45	0,6661429	0,04349912	0,02482316	0,02823023	0,000134439	0,000448069	0,000091529	0,003196536	-0,006651589	4_2	Pigs
45	0,6423839	0,1318436	0	0,00460588	0,004164415	0,000520039	0,000549837	0,002372951	0,1272377	5_1	Sheep
45	0,5809541	0,001917238	0,2486832	0,03143648	0,000000145	0,003388401	0,02556897	0,000726316	-0,2531836	5_2	Sheep
**Participant 46**											
**Participant_ID**	**Neutral_Mean**	**Happy_Mean**	**Sad_Mean**	**Angry_Mean**	**Surprised_Mean**	**Scared_Mean**	**Disgusted_Mean**	**Contempt_Mean**	**Valence_Mean**	**Animal_Id**	**Animal**
46	0,8117003	0,003498096	0,008554964	0,01044958	0,000188733	0,001234739	0,01532272	0,006942299	-0,01416407	1_1	Dogs

46	0,8581988	0,01444396	0,002586625	0,019215	0,000442823	0,002797496	0,005704215	0,005991267	-0,004771041	1_2	Dogs
46	0,2391928	0,5312862	0,004282306	0,000010597	0,000310922	0,000335137	0	0,003909822	0,5266952	1_3	Dogs
46	0,8868525	0,04347685	0,000396015	0,03729009	0,000075969	0,000488078	0,03098584	0,01522568	0,006186764	1_4	Dogs
46	0,6964169	0,01496733	0,02427556	0,06448429	0,000013786	0,000088554	0,03618203	0,008513425	-0,04999961	1_5	Dogs
46	0,8518404	0,01041092	0,000050207	0,05281527	0,000009631	0,000061878	0,004537944	0,002352078	-0,04240434	1_6	Dogs
46	0,6761245	0,002929854	0,03125094	0,03837435	0,000058829	0,000014308	0,05166173	0,01163065	-0,0636827	1_7	Dogs
46	0,7274058	0,00145611	0,01236278	0,01628499	0,000056295	0,000006642	0,04134036	0,008033061	-0,04021966	1_8	Dogs
46	0,7653255	0,001607629	0,008602991	0,009859126	0,000018026	0,000002127	0,03042959	0,003652449	-0,02882196	1_9	Dogs
46	0,7122161	0,01839198	0,002788607	0,04174707	0,000003789	0,000000447	0,04221943	0,01777321	-0,02811996	1_10	Dogs
46	0,759981	0,04750619	0,01050331	0,008944303	0,000002103	0,000000248	0,01048438	0,03653676	0,0324443	1_11	Dogs
46	0,7147797	0,04665499	0,002663825	0,04073702	0,000000528	0,000000062	0,04247371	0,02786419	-0,009589661	1_12	Dogs
46	0,5586715	0,01072106	0,01591444	0,08437087	0,000000093	0,000000011	0,1141643	0,01830701	-0,1051214	1_13	Dogs
46	0,7215348	0,000296517	0,08952566	0,004069859	0,000698956	0,009731476	0,006121946	0,004096726	-0,09232414	2_1	Cats
46	0,7569848	0,07617028	0,18512	0,002747049	0,000004394	0,000160198	0,003043302	0,0643221	-0,1089498	2_2	Cats
46	0,9072294	0,08395731	0,02009407	0,00091659	0,000000063	0,000002298	0,000075836	0,01162392	0,06386324	2_3	Cats
46	0,9276145	0,06677862	0,004500085	0,000205271	0,000000014	0,000000515	0,000016984	0,002603188	0,06227855	2_4	Cats
46	0,9577133	0,03038903	0,000892138	0,000040695	0,000000003	0,000000102	0,000003367	0,00051608	0,02949689	2_5	Cats
46	0,9687541	0,01282047	0,00035775	0,000016319	0,000000001	0,000000041	0,00000135	0,000206949	0,01246272	2_6	Cats
46	0,975039	0,005765624	0,000160887	0,000007339	0,000000001	0,000000018	0,000000607	0,000093069	0,005604737	2_7	Cats
46	0,9807863	0,001523144	0,000042503	0,000001939	0	0,000000005	0,00000016	0,000024587	0,001480641	2_8	Cats
46	0,9673972	0,000969767	0,001366352	0,000409565	0,02097361	0,008213771	0,000159138	0,00208418	-0,007244004	3_1	Horses
46	0,5773504	0,000330692	0,01112955	0,01003801	0,000000498	0,03596695	0,001530765	0,02484392	-0,03563626	3_2	Horses
46	0,675422	0,05407748	0,001194983	0,006877028	0,01198256	0,04506114	0,004914159	0,006666999	0,009016337	3_3	Horses
46	0,6982494	0,01730467	0,01527037	0,000611059	0,04033034	0,04744102	0,0002383	0,02154198	-0,03131927	3_4	Horses
46	0,5732026	0,000823016	0,01903401	0,01910956	0,004956955	0,000560913	0,00328545	0,000961707	-0,02918506	4_1	Pigs
46	0,7636469	0,001213784	0,03689954	0,03148869	0,001358975	0,000528127	0,01118594	0,000823969	-0,06613365	4_2	Pigs
46	0,5396475	0,004944073	0,008662687	0,06019699	0,07114249	0,00546333	0,005180777	0,01648838	-0,05818675	5_1	Sheep
46	0,5088036	0,2839246	0,00767644	0,000124045	0,001279225	0,0000317	0,0186361	0,002145008	0,259057	5_2	Sheep
**Participant 47**											

Participant_ID	Neutral_Mean	Happy_Mean	Sad_Mean	Angry_Mean	Surprised_Mean	Scared_Mean	Disgusted_Mean	Contempt_Mean	Valence_Mean	Animal_Id	Animal
47	0,7525948	0,001291543	0,01676396	0,001376355	0,000028853	0,00332804	0,000007585	0,004879281	-0,01812446	1_1	Dogs
47	0,5847142	0,000022117	0,1849018	0,001024371	0,000052689	0,000464144	0,000084359	0,000132977	-0,1848796	1_2	Dogs
47	0,5763856	0,02195255	0,04038059	0,02912637	0,00437461	0,03458786	0,00385155	0,009479164	-0,07378808	1_3	Dogs
47	0,3863706	0,000001773	0,4380232	0,000082131	0,000026217	0,000037214	0,00166255	0,000466842	-0,4380215	1_4	Dogs
47	0,3575343	0,000000803	0,4666695	0,000037169	0,000011865	0,000016842	0,002255107	0,000378359	-0,4666687	1_5	Dogs
47	0,3718122	0,000000479	0,4134123	0,000022162	0,000007074	0,000010042	0,002226782	0,000225597	-0,4134118	1_6	Dogs
47	0,3988962	0,000000281	0,3439825	0,000013033	0,000004694	0,000005905	0,001737165	0,000132668	-0,3439822	1_7	Dogs
47	0,4849887	0,000000087	0,2054767	0,000004007	0,000034928	0,000001816	0,00060263	0,000040791	-0,2054766	1_8	Dogs
47	0,4534437	0,000000002	0,3050077	0,0000001	0,000018665	0,000891039	0,000077826	0,002338284	-0,3050077	1_9	Dogs
47	0,4360229	0,004176683	0,3070158	0,008853513	0,000663656	0,002259906	0,002181067	0,002345661	-0,3028392	1_10	Dogs
47	0,2239928	0,007170233	0,5921116	0,03791393	0,000217338	0,002932827	0,01454609	0,004682678	-0,5849414	1_11	Dogs
47	0,2471267	0,006320262	0,5521178	0,04538472	0,000282358	0,004010952	0,01341996	0,003623785	-0,5457976	1_12	Dogs
47	0,3251845	0,003122335	0,4303558	0,04907374	0,001919299	0,01581366	0,009930035	0,001657613	-0,4272334	1_13	Dogs
47	0,4958917	0,1167381	0,001269269	0,000090498	0	0,000000065	0,000196681	0,006020594	0,115454	2_1	Cats
47	0,3947294	0,2396222	0,000200228	0,001043112	0	0,000000001	0,000153022	0,001362722	0,2385791	2_2	Cats
47	0,4747016	0,01861496	0,01658046	0,004932	0	0,000766003	0,06529301	0,007172265	-0,04667806	2_3	Cats
47	0,4647253	0,005672682	0,002606456	0,01032962	0,001171089	0,000661658	0,09157323	0,002494947	-0,08590055	2_4	Cats
47	0,4953213	0,01315019	0,000150402	0,001479138	0,000112164	0,00003818	0,01034263	0,000178251	0,002793378	2_5	Cats
47	0,4477027	0,1192988	0,000889327	0,008043416	0,000127788	0,00225838	0,0223063	0,01113854	0,09678843	2_6	Cats
47	0,5192239	0,1461296	0,000044987	0,002663509	0,000111188	0,001831968	0,005008238	0,04159946	0,1411214	2_7	Cats
47	0,6973981	0,02239165	0,000006287	0,000372251	0,00001554	0,000273108	0,000699949	0,008221381	0,0216917	2_8	Cats
47	0,4828174	0,1369059	0,004549259	0,05311839	0,001328721	0,007616317	0,04400864	0,004349303	0,06910051	3_1	Horses
47	0,7312459	0,00010295	0,001220562	0,02864564	0,09851761	0,006431903	0,000175156	0,009459541	-0,03055593	3_2	Horses
47	0,5346069	0,06426129	0	0,006516148	0,02277563	0,03146086	0,000053287	0,001582524	0,02827333	3_3	Horses
47	0,5942141	0,01647296	0,01453451	0,05762406	0,000024809	0,01604344	0,03300171	0,02366288	-0,05655506	3_4	Horses
47	0,8128996	0,000463213	0,04793194	0,01587516	0,005948209	0,00157199	0,002112304	0,000096234	-0,05544228	4_1	Pigs
47	0,803563	0,000234344	0,01566715	0,002675975	0,02470264	0,01862512	0,00148369	0,005888036	-0,02348963	4_2	Pigs
47	0,6251045	0,08689056	0,01263474	0,006354641	0,008108497	0,002184433	0,03772394	0,000172717	0,04790465	5_1	Sheep
47	0,4658701	0,05212562	0,01688612	0,03143765	0,05892361	0,000879666	0,00736959	0,01230451	0,009470279	5_2	Sheep

**Participant 48**											
**Participant_ID**	**Neutral_Mean**	**Happy_Mean**	**Sad_Mean**	**Angry_Mean**	**Surprised_Mean**	**Scared_Mean**	**Disgusted_Mean**	**Contempt_Mean**	**Valence_Mean**	**Animal_Id**	**Animal**
48	0,6323503	0,001670764	0	0,000456096	0,004031168	0,007972206	0	0,002513527	-0,006301443	1_1	Dogs
48	0,4273356	0,2143689	0	0,000102667	0,00301235	0,003246954	0	0,00609	0,211122	1_2	Dogs
48	0,3517778	0,005837563	0,1876149	0,1195664	0,000859814	0,000585763	0,01328376	0,001366398	0,2959114	1_3	Dogs
48	0,202348	0,5969716	0,02726517	0,000001465	0,00004297	0,000046317	0	0,000590119	0,5697064	1_4	Dogs
48	0,2494474	0,5017152	0,01667753	0,000000449	0,000013167	0,000014192	0	0,000180821	0,4850377	1_5	Dogs
48	0,3127009	0,3750507	0,0134015	0,000000113	0,000003306	0,000003563	0	0,000636037	0,3616492	1_6	Dogs
48	0,3946239	0,2114507	0,002558106	0,000005479	0,00000063	0,000000679	0	0,001396733	0,2088926	1_7	Dogs
48	0,4202888	0,1598742	0,0005286	0,000003265	0,000447308	0,00000014	0	0,000481943	0,1593456	1_8	Dogs
48	0,3917624	0,2168054	0,000414459	0,000001008	0,000710537	0,000000043	0	0,001174828	0,2163909	1_9	Dogs
48	0,4142441	0,1720003	0,01914026	0,000000611	0,000824692	0,000000011	0,000001453	0,002339062	0,15286	1_10	Dogs
48	0,3914445	0,2175916	0,03682583	0,000082011	0,000559197	0,000000004	0,000001614	0,001327718	0,1807658	1_11	Dogs
48	0,3880626	0,2243922	0,01851145	0,000129455	0,001087185	0,000000002	0,000002313	0,001338265	0,2058808	1_12	Dogs
48	0,410884	0,1788861	0,009042365	0,000020696	0,001796662	0,000000001	0,000000323	0,0017299	0,1698437	1_13	Dogs
48	0,801661	0,008029593	0,004350093	0,009627166	0,02253557	0,01517141	0,007561365	0,007676392	-0,01712	2_1	Cats
48	0,7140439	0,005388008	0,003326318	0,01665381	0,0376599	0,01968715	0,000832669	0,004287506	-0,02304789	2_2	Cats
48	0,7806468	0,003550718	0,000530346	0,001912886	0,04816454	0,01542416	0,009443082	0,004684735	-0,01623477	2_3	Cats
48	0,943413	0,005474449	0,000071927	0,000259429	0,007393412	0,00217242	0,01342741	0,01358149	-0,007952957	2_4	Cats
48	0,9720278	0,002532302	0,000013137	0,000047382	0,001350326	0,000396769	0,0279475	0,005685758	-0,02541519	2_5	Cats
48	0,9679521	0,000486483	0,00000204	0,000007358	0,000209707	0,000061619	0,057469	0,02987297	-0,05698252	2_6	Cats
48	0,9615416	0,000224255	0,000000162	0,000000584	0,000016656	0,000004894	0,07494177	0,03508233	-0,07471751	2_7	Cats
48	0,9606969	0,00006763	0,000000049	0,000000176	0,000005023	0,000001476	0,07611282	0,02019115	-0,07604519	2_8	Cats
48	0,6588	0,003792213	0,006121418	0,000000123	0,000088007	0,000000001	0,03440528	0,01221508	-0,03061306	3_1	Horses
48	0,675656	0,00834087	0,007642059	0,05185721	0,000072027	0,01715718	0,0168449	0,001452539	-0,04351634	3_2	Horses
48	0,5011492	0,1141137	0,03444183	0,003824575	0,000067137	0,0266053	0,000086938	0,006045938	-0,05969035	3_3	Horses
48	0,7995329	0,002447251	0,01467336	0,000141643	0,07643263	0,008025656	0,000439068	0,002222769	-0,02026873	3_4	Horses
48	0,7185959	0,006344692	0,06124632	0,000459886	0,000479238	0,001345308	0,04309373	0,001477867	-0,07947437	4_1	Pigs
48	0,9536425	0,000129549	0,003493435	0,009231944	0,000263206	0,000376014	0,000040552	0,000016765	-0,01234534	4_2	Pigs
48	0,6495132	0,01951443	0,009872558	0,003503199	0,000000693	0,000023471	0,002329502	0,000482656	0,008758953	5_1	Sheep

48	0,3616246	0,2359688	0,001959132	0,01207424	0,0237563	0,004604336	0,1952201	0,001738044	0,04074866	5_2	Sheep
**Participant 49**											
**Participant_ID**	**Neutral_Mean**	**Happy_Mean**	**Sad_Mean**	**Angry_Mean**	**Surprised_Mean**	**Scared_Mean**	**Disgusted_Mean**	**Contempt_Mean**	**Valence_Mean**	**Animal_Id**	**Animal**
49	0,5321212	0,008437376	0,004056876	0,004096273	0,03007512	0,009243873	0,05666899	0,005098526	0,144856	1_1	Dogs
49	0,4000691	0,09470414	0,06561416	0,005251828	0,002474422	0,01342876	0,04210066	0,005098526	-0,02621746	1_2	Dogs
49	0,3709129	0,2411934	0,01894906	0,001656026	0,002971274	0,00413102	0,001181138	0,000721109	0,2167089	1_3	Dogs
49	0,4572247	0,1990578	0,1433156	0,02555414	0,01481486	0,01109982	0,02809398	0,004544011	-0,008013413	1_4	Dogs
49	0,6488327	0,7119359	0,1785029	0,000122688	0,001425427	0,01009654	0,000710768	0,000368428	0,1880361	1_5	Dogs
49	0,4232361	0,00019207	0,04127733	0,3265972	0	0,000246699	0,000858204	0,000358174	0,1074308	1_6	Dogs
49	0,6008814	0,000019906	0,05249821	0,01381447	0,002992971	0,004032918	0,008143005	0,01435662	0,06213888	1_7	Dogs
49	0,8881175	0,004191492	0,02136833	0,001404412	0,05682889	0,001719062	0,000598146	0,001256907	0,007446633	1_8	Dogs
49	0,4974197	0,000653034	0,00150754	0,000300766	0,01309305	0,01963851	0,000013743	0,003462737	-0,02027389	1_9	Dogs
49	0,7560181	0,005019162	0,04034862	0,008639655	0,06331681	0,005681857	0,008730969	0,001679742	0,4718715	1_10	Dogs
49	0,8321401	0,000032994	0,002212381	0,0163943	0,01909604	0,003200393	0,1240167	0,002311889	-0,02982437	1_11	Dogs
49	0,4690717	0,000023906	0,2294518	0,00179824	0,006431988	0,000000065	0,000008456	0,000475251	0,2300068	1_12	Dogs
49	0,1857807	0,6310219	0,000760235	0,000879432	0,000121503	0,02227256	0,000456222	0,001661759	0,6298648	1_13	Dogs
49	0,1857807	0,6310219	0,000760235	0,000879432	0,000121503	0,000083105	0,000456222	0,001661759	0,6298648	2_1	Cats
49	0,4611452	0,09240409	0,00204739	0,00386135	0,009509287	0,000002516	0,002497328	0,02534704	0,08773366	2_2	Cats
49	0,6014357	0,002650975	0,009154864	0,006324963	0,002746863	0,000074738	0,003362697	0,01062478	-0,01004212	2_3	Cats
49	0,6464167	0,000318723	0,115242	0,02023947	0,000358062	0,00138199	0,00581597	0,01110321	-0,1149232	2_4	Cats
49	0,5233886	0,000063892	0,2063747	0,01300742	0,000071778	0,001673246	0,01889136	0,003613031	-0,2063108	2_5	Cats
49	0,4432016	0,000015348	0,2567274	0,003291681	0,000017242	0,000940174	0,05333208	0,000867921	-0,2567121	2_6	Cats
49	0,4607628	0,000004847	0,1779577	0,001039535	0,000005445	0,001098257	0,1085678	0,000274095	-0,1779528	2_7	Cats
49	0,4450154	0,00000135	0,1898285	0,000289536	0,000001517	0,004879378	0,1666583	0,000076342	-0,1898272	2_8	Cats
49	0,6744562	0,1417228	0,001026684	0,0203577	0,005111705	0,01792396	0,002118102	0,01501569	0,1098877	3_1	Horses
49	0,5826881	0,004393469	0	0,00953617	0,04594313	0,01130029	0,000526518	0,001448962	-0,01349199	3_2	Horses
49	0,6092201	0,05722251	0,02146268	0,001336153	0,009393499	0,01951755	0,001235757	0,002120251	0,02288397	3_3	Horses
49	0,6643038	0,08019581	0,00013205	0,00336277	0,008947523	0,06761426	0,000486667	0,005516057	0,01258157	3_4	Horses
49	0,5883154	0,002986957	0,1113173	0,09851685	0,000601971	0,008148942	0,01251486	0,000351774	-0,1801766	4_1	Pigs

49	0,9550316	0,01330976	0,009525466	0,001131178	0,002107618	0,000006046	0,004312942	0,008769788	0,001482782	4_2	Pigs
49	0,4061491	0,1813138	0,000525465	0	0,000018326	0,000002331	0,02205925	0,000817612	0,1592546	5_1	Sheep
49	0,5417651	0,01943229	0,02157806	0,007251637	0	0,000014845	0,000019964	0,000231887	-0,00340216	5_2	Sheep
**Participant 50**											
**Participant_ID**	**Neutral_Mean**	**Happy_Mean**	**Sad_Mean**	**Angry_Mean**	**Surprised_Mean**	**Scared_Mean**	**Disgusted_Mean**	**Contempt_Mean**	**Valence_Mean**	**Animal_Id**	**Animal**
50	0,75104473	-0,034414439	-0,03802122	0,19976713	-0,036173908	-0,00494081	-0,038950056	-0,02643426	0,20102183	1_1	Dogs
50	0,6540452	0,07406136	0,006378814	0,000557707	0,01165562	0,03372017	0,000584784	0,04737801	-0,03866177	1_2	Dogs
50	0,7905734	0,005114231	0,001507448	0,2392958	0,003354762	0,03458786	0,000578614	0,01309441	0,2405505	1_3	Dogs
50	0,6583131	0,070165095	0,03759042	0,007366145	0,01168313	0,01686933	0,008249347	0,01113264	-0,05342365	1_4	Dogs
50	0,5558719	0,00840747	0,2114823	0,00517416	0,000421842	0,000373056	0,009851594	0,004091396	-0,2087624	1_5	Dogs
50	0,5577777	0,01981293	0,000000863	0,000001383	0,00451023	0	0,01729801	0,00485217	0,07717006	1_6	Dogs
50	0,8008814	0,000019906	0,033955	0,01381447	0,002992971	0,8008814	0,000019906	0,05249821	0,01381447	1_7	Dogs
50	0,4786673	0,000053402	0,0484497	0,003504092	0,000858506	0,01302482	0,009587768	0,03763623	-0,06039209	1_8	Dogs
50	0,6256235	0,000653034	0,221686	0,000749353	0,001768852	0,001302892	0,001635947	0,003676325	-0,2212356	1_9	Dogs
50	0,4723884	0,000808701	0,02348511	0,008959591	0,01089287	0,000577259	0,000000732	0,02716924	0,2529501	1_10	Dogs
50	0,4837009	0	0,03401581	0,04708935	0	0	0,05390104	0,000101361	-0,1040465	1_11	Dogs
50	0,7011364	0,1814691	0,00026354	0,03821827	0,000000967	0,000000001	0,02853335	0,001139459	-0,05683179	1_12	Dogs
50	0,5549143	0,0249535	0,03298629	0,02099126	0,00007585	0,000751804	0,06234021	0,000559488	-0,06305315	1_13	Dogs
50	0,9047397	0,000101762	0,001770329	0,000001328	0,000059496	0,000016978	0,000001875	0,000410209	-0,001668567	2_1	Cats
50	0,8904513	0,001804926	0,005442807	0,000072786	0,000990143	0,000033879	0,00001792	0,002519096	-0,003637881	2_2	Cats
50	0,8364255	0,000216735	0,07026097	0,000005336	0,000109159	0,000023117	0,000066788	0,000228146	-0,07004423	2_3	Cats
50	0,8321677	0,000041439	0,06338474	0,00000102	0,000139923	0,000102701	0,000079619	0,000043621	-0,0633433	2_4	Cats
50	0,7892068	0,000012474	0,1058308	0,000243718	0,000067747	0,000143894	0,000184415	0,00001313	-0,1058183	2_5	Cats
50	0,7918047	0,000002584	0,1038887	0,000223877	0,000019922	0,0001393	0,000165751	0,00000272	-0,1038861	2_6	Cats
50	0,8444512	0,000000939	0,04931175	0,000081336	0,000007238	0,000119902	0,000067298	0,000096558	-0,04931081	2_7	Cats
50	0,8472815	0,000745887	0,03113398	0,00014846	0,000357908	0,000035542	0,000120851	0,000808521	-0,0303881	2_8	Cats
50	0,543691	0,00266554	0	0,0149946	0,007441001	0,002363977	0,01036526	0,01632802	-0,01736237	3_1	Horses
50	0,7594216	0,000091054	0,1150745	0,001934817	0,002255433	0,004791515	0,001454321	0,001647068	0,1246364	3_2	Horses
50	0,6541963	0,000007124	0,007556758	0,0021329	0,03921056	0,009767478	0,000412931	0,000467715	0,01114377	3_3	Horses

50	0,5281817	0,0670314	0	0,07180584	0,003433307	0,01074599	0,001323103	0,000946275	-0,005258315	3_4	Horses
50	0,7997316	0,03433292	0,005633313	0,05011907	0,002395167	0,003038798	0,02029378	0,006991077	-0,02084085	4_1	Pigs
50	0,5861474	0,6316614	0,03694827	0,02629608	0,000410058	0,0002413	0,05975827	0,00041587	0,08230197	4_2	Pigs
50	0,7925392	0,0685185	0,02985214	0,01452997	0,001111656	0,000032781	0,03061996	0,007957295	0,03453348	5_1	Sheep
50	0,3248357	0,3997368	0,01022376	0	0,01165212	0,00014694	0,003633631	0,005051285	0,388895	5_2	Sheep
