# Supplementary material for: Personalized Strategy for Animal-Assisted Therapy for Individuals Based on the Emotions Induced by the Images of Different Animal Species and Breeds
Source: Animals (Basel). 2022 Feb 27;12(5):597. doi: 10.3390/ani12050597 (PMC8909388; doi:10.3390/ani12050597)
Supplement: Supplementary file 1 [file animals-12-00597-s001.zip › Supplementary File 3. Multivariate ANOVA results.pdf]

Multivariate ANOVA results

Source		Type III Sum of Squares	df	Mean Square	F	Sig.
species	neutral	1,169	4	,292	7,953	,000
	happy	,438	4	,110	12,893	,000
	sad	,017	4	,004	,446	,776
	angry	,005	4	,001	,543	,705
	surprised	,001	4	,000	,759	,552
	scared	,040	4	,010	3,366	,009
	disgusted	,002	4	,000	,651	,626
	contempt	,000	4	7,483E-05	,735	,568
	valence	,349	4	,087	3,581	,006
breed	neutral	,237	12	,020	,537	,891
	happy	,418	12	,035	4,097	,000
	sad	,117	12	,010	1,026	,422
	angry	,014	12	,001	,544	,887
	surprised	,002	12	,000	,496	,918
	scared	,020	12	,002	,580	,860
	disgusted	,007	12	,001	,878	,570
	contempt	,001	12	5,323E-05	,523	,901
	valence	,782	12	,065	2,677	,001
species * breed	neutral	,141	12	,012	,320	,986
	happy	,084	12	,007	,829	,621
	sad	,038	12	,003	,330	,984
	angry	,011	12	,001	,416	,958
	surprised	,002	12	,000	,575	,864
	scared	,023	12	,002	,656	,795
	disgusted	,005	12	,000	,663	,788
	contempt	,001	12	7,239E-05	,711	,742
	valence	,121	12	,010	,416	,958

Analysed factors and theyr Interaction		Emotions induced for persons								Valence
species	breed	neutral	happy	sad	angry	surprised	scared	disgusted	contempt	
1	1	0,609369	0,046475	0,020066	0,023292	0,016036	0,01031	0,001368	0,00771	0,015735
1	1	0,609369	0,347424	4,67E-07	0,001317	0,000594	0,002165	0,006233	0,000164	0,339354
1	1	0,96671	8,08E-05	0,027882	0,000634	0,000117	4,09E-05	0,00217	0,000718	-0,02946
1	1	0,640139	0,086207	0,014571	1,3E-05	0,005441	0,0035	0,000261	0,021094	0,068053
1	1	0,59127	0,030488	0,052271	2,3E-08	2E-09	0,000445	0,00337	0,000122	-0,02529
1	1	0,843054	0,000135	0,029654	0,024761	0,058657	0,002008	0,002775	0,004972	-0,04438
1	1	0,550193	0	0,297629	0,048939	0,000545	0,000996	0,046311	0,013656	-0,33253
1	1	0,481542	0,29421	0,020911	8,85E-05	5E-08	0,000678	0,000378	0,004465	0,273189
1	1	0,356593	0,405083	0	0,004996	0,000236	0,003036	0	0,000635	0,397513

1	1	0,464551	0,348235	0,000974	9,6E-08	0,000592	0,010792	0	0,007585	0,336882
1	1	0,752094	0,003527	0,010667	0,011869	0,014863	0,029632	0,00304	0,012858	-0,03839
1	1	0,823747	3,27E-05	0,073645	0,002558	0,005602	0,008961	0	0,002026	-0,08358
1	1	0,871475	0,000518	0,009812	0,012725	0,021498	0,004	2,97E-05	0,008906	-0,02353
1	1	0,699175	0,003854	0,020782	0,057488	0,009521	0,007103	0,00142	0,014251	-0,07524
1	1	0,675486	5,16E-06	0,041483	0,025438	0,000172	0,018706	0,003853	3,14E-07	-0,07206
1	1	0,701425	0,027092	0,00017	0,008426	0,003481	0,05733	6,35E-06	0,004718	-0,03197
1	1	0,647103	0,008646	0,007759	0,085328	0,033693	0,01917	0,001407	0,000827	-0,08912
1	1	0,375843	0,402845	0,001792	2,95E-05	0,006582	4,41E-06	0,000502	0,012094	0,401024
1	1	0,47252	0,127026	0,03067	0,007927	0,017473	0,03063	0,034367	0,009968	0,041898
1	1	0,519892	0,231236	0,020574	0,045034	0,00156	0,000919	0,012693	0,010813	0,159916
1	1	0,641433	0,001505	0,091886	0,007237	0,016549	0,001717	0,007092	0,012642	-0,09283
1	1	0,433206	0,170601	0,021934	0,003629	0,048812	0,001983	0,018663	0,001116	0,14201
1	1	0,671196	0,035146	0,009087	0,030669	0,029765	0,005674	0,000885	0,002118	-0,00377
1	1	0,905364	0,001113	0,009354	0,004116	0,002194	0,002044	0,003259	0,001017	-0,01192
1	1	0,577294	0,037865	0,028805	0,023979	0,002745	0,006299	0,00919	0,03407	-0,01169
1	1	0,555145	0,022711	0,014907	0,016203	0,016006	0,022999	0,041025	0,003156	-0,0521
1	1	0,415864	0,004757	0,026813	0,235966	0,001589	0,000616	0,017856	0,00201	-0,24735
1	1	0,464181	0,052108	0,016081	0,003506	0,007397	0,00647	0,000842	0,000871	0,028442
1	1	0,43813	0,001258	0,012794	0,213116	0,00362	0,002642	0,108679	0,00299	-0,31076
1	1	0,581153	0,071441	0,012616	0,000663	0,005999	0,059873	0,033347	0,004146	-0,02419
1	1	0,779185	0,000422	1,78E-05	0,067549	0,009043	0,005697	0,013824	0,001999	-0,07679
1	1	0,367747	0,000125	0,561472	0,000464	0	4,91E-05	2,8E-05	6,87E-05	-0,56135
1	1	0,74817	0,042001	0,081896	0,016468	0,001509	0,005247	0,01516	0,004066	-0,0666
1	1	0,341988	0,004229	0,175728	0,011807	0,007355	0,236808	0,007857	0,001159	-0,39964
1	1	0,431415	0,091547	0,002639	0,01049	0,003792	0,039238	0,031636	0,004557	0,032998
1	1	0,499881	0,021918	0,131105	0,013705	0,000556	0,005479	0,026269	0,004096	-0,11103
1	1	0,412023	0,027506	0,082023	0,013774	0,000428	0,152801	0,004427	0,003074	-0,20908
1	1	0,72663	0,007945	0,026284	0,00273	0,0013	0,012488	0,020316	0,000498	-0,0354
1	1	0,677369	0,001741	0,020928	0,013598	0,000943	0,017842	0,055864	0,001241	-0,07988
1	1	0,637516	0,008889	0,002548	0,000603	0,031657	0,025806	0,002664	7,23E-05	-0,02185
1	1	0,966152	0	0,012621	0,00987	0,028838	0	0	0,010451	-0,01926
1	1	0,6924	0,001271	0,006015	0,032173	2E-09	1E-09	0,056669	0,00092	-0,08643
1	1	0,870079	0,008437	0,044839	0,013358	0,024547	0,004096	0,064592	0,017019	-0,08389
1	1	0,599815	7,07E-05	0,005766	0,023711	0,028305	0,024612	0,011339	0,002027	-0,03187
1	1	0,442416	0,107911	0,000616	0	2,26E-07	2,74E-06	0,024309	0,000845	0,083602
1	1	0,8117	0,003498	0,008555	0,01045	0,000189	0,001235	0,015323	0,006942	-0,01416
1	1	0,752595	0,001292	0,016764	0,001376	2,89E-05	0,003328	7,59E-06	0,004879	-0,01812
1	1	0,63235	0,001671	0	0,000456	0,004031	0,007972	0	0,002514	-0,0063
1	1	0,532121	0,008437	0,004057	0,004096	0,030075	0,009244	0,056669	0,005099	0,144856
1	1	0,751045	-0,03441	-0,03802	0,199767	-0,03617	-0,00494	-0,03895	-0,02643	0,201022
1	2	0,491098	0,230845	0,010621	0,006427	0,01011	0,025192	0,000228	0,004806	0,195235
1	2	0,707557	0,273352	1E-09	0,001942	3E-09	0,000644	0,00306	0,000139	0,268908
1	2	0,984308	0	0,019593	1,9E-08	0,000178	3,06E-05	5,05E-06	3,07E-05	-0,01959
1	2	0,619916	0,000236	0,128179	0,00567	0	0,000301	0,000824	0,001069	-0,12874
1	2	0,496192	0,176547	0,008192	1,99E-05	0,000136	0,000184	0,006602	0,000176	0,164247
1	2	0,847407	0,005069	0,040425	0,021554	0,00169	0,00706	0,002227	0,010488	-0,04878
1	2	0,59138	0	0,186313	0,101843	0,000739	0,003004	0,006808	0,007249	-0,27819

1	2	0,584461	0,109246	0,018299	2,51E-05	0	0,002456	0,000188	0,001353	0,089906
1	2	0,32273	0,335368	0,007902	0,000259	0,042512	0	0,0002	0,004266	0,327443
1	2	0,594945	0,102014	0,011515	0,002206	0,003016	0,01282	0,008792	0,020324	0,077519
1	2	0,589191	0,153471	0,00592	0,001616	0,036855	0,016087	0,000825	0,023104	0,133957
1	2	0,79479	0,000224	0,068768	0,012713	0,000234	0,008858	0	0,006465	-0,0849
1	2	0,859991	0,000588	0,012014	0,009881	0,051526	0,005667	0,000157	0,003683	-0,02341
1	2	0,785322	0,000563	0,042286	0,024867	0,010102	0,002349	0,002992	0,001123	-0,06496
1	2	0,637698	0,059372	0,059853	0,011924	0,020159	0,002546	0,001592	0,077795	-0,01027
1	2	0,907132	0,000271	0,000923	0,012605	0,017224	0,041357	0,000108	0,000754	-0,04293
1	2	0,57909	0,005429	0,009254	0,049295	0,005558	0,01189	0,004768	0,000394	-0,05514
1	2	0,610374	0,041024	0,054128	0,001057	0,004036	0,035306	0,016577	0,008769	-0,02379
1	2	0,492837	0,067145	0,026257	0,013993	0,003021	0,024987	0,00028	0,015355	0,019629
1	2	0,343605	0,408457	0,007535	0,021753	0,00108	0,001782	0,048539	0,022265	0,339667
1	2	0,752352	0,000163	0,005569	0,027936	0	0,011962	2,71E-05	0,069977	-0,03685
1	2	0,43276	0,284146	0,001407	0,001373	0,004095	6,53E-05	0,007158	0,000169	0,275049
1	2	0,836494	0,011729	0,010396	0,019473	0,004533	0,003342	0,000117	0,004762	-0,0191
1	2	0,433863	0,403523	0,001414	0,010317	0,004752	0,004779	0,006581	0,011932	0,385951
1	2	0,343551	0,403427	0,000343	0,082401	0,008869	0,019901	0,046203	3,64E-05	0,283447
1	2	0,487127	0,105093	0,038135	0,053697	0,027372	0,007136	0,030193	0,008065	0,00411
1	2	0,663951	0,034804	0,029269	0,056908	0,00031	0,010487	0,000298	0,001467	-0,05211
1	2	0,281335	0,428443	0,031308	0,000103	0,002816	0	0	0,000805	0,397135
1	2	0,433333	0,004227	5,63E-06	0,016472	0,001484	0,003073	0,227703	8,4E-08	-0,22488
1	2	0,660722	0,116826	0,000253	0,009957	0,011711	0,008945	1,1E-08	0,015159	0,101966
1	2	0,817226	0	0,001651	0,185218	0,006736	0	0,009103	0,00375	-0,18801
1	2	0,348969	0,000225	0,529961	0,001352	0	0,000453	0,000857	0,000851	-0,53095
1	2	0,748692	0	0,179231	0,020035	0,004758	0,00887	0,004873	0,009003	-0,19648
1	2	0,339698	0,061995	0,054427	0,026209	0,015424	0,280098	0,014353	0,001093	-0,26404
1	2	0,4534	0,0715	0,029992	0,011241	0,009247	0,03678	0,011025	0,011549	-0,0031
1	2	0,548747	0,040445	0,10764	0,013529	0,000777	0,007558	0,034692	0,004844	-0,07673
1	2	0,332579	0,191727	0,032617	7,96E-05	0,003512	0,21399	0,001436	0,001951	-0,05167
1	2	0,589536	0,023508	0,082003	0,002796	0,001463	0,040574	0,042101	0,00099	-0,10681
1	2	0,822824	0,023238	0,001609	0,039957	0,007222	0,015229	0,000536	0,000764	-0,03055
1	2	0,606695	8,28E-06	0,000187	9,22E-05	0,046564	0,058342	6,72E-06	0,001848	-0,05845
1	2	0,902335	0	1,49E-05	0,021883	0	0	0,01088	6,53E-06	-0,02188
1	2	0,753384	0,008193	0,013014	8,43E-05	0	0,00109	0,049652	0,002304	-0,04408
1	2	0,843071	0,000949	0,09489	0,003385	0,009603	0,00151	0,077531	0,006062	-0,11624
1	2	0,83222	2,81E-06	0,001469	0,020232	0,071803	0,017884	0,000689	0,000316	-0,02023
1	2	0,175365	0,64812	1,9E-05	0	0,001619	7,6E-08	0,007049	0,000491	0,641072
1	2	0,858199	0,014444	0,002587	0,019215	0,000443	0,002797	0,005704	0,005991	-0,00477
1	2	0,584714	2,21E-05	0,184902	0,001024	5,27E-05	0,000464	8,44E-05	0,000133	-0,18488
1	2	0,427336	0,214369	0	0,000103	0,003012	0,003247	0	0,00609	0,211122
1	2	0,400069	0,094704	0,065614	0,005252	0,002474	0,013429	0,042101	0,005099	-0,02622
1	2	0,654045	0,074061	0,006379	0,000558	0,011656	0,03372	0,000585	0,047378	-0,03866
1	3	0,472024	0,160668	0,03805	0,011215	0,00954	0,02128	1,52E-05	0,000792	0,10164
1	3	0,804616	0,117978	0,000792	1,85E-05	2,3E-06	0,000154	0,002223	1,43E-07	0,115571
1	3	0,987525	0	0,00982	0	0,005274	3E-09	0,001942	8,3E-05	-0,01141
1	3	0,706056	0,00487	0,062678	0,005532	0	0	0,002805	0,005181	-0,05992

1	3	0,529648	0,00553	0,001568	0,011337	1,61E-06	0	0,011321	3,92E-06	-0,01461
1	3	0,92678	0,000927	0,020315	0,005276	0,001397	0,002733	0,003824	0,00319	-0,02265
1	3	0,732318	0	0,134788	0,050203	0,001044	0,001676	0,000229	0,005887	-0,18
1	3	0,650929	0,002682	0,088355	0,000113	0,000169	0,042444	0,00685	0,001565	-0,12361
1	3	0,48454	0,036311	0,003617	0,000427	1,49E-06	0,016204	1,3E-08	0,003749	0,016754
1	3	0,766332	0,009495	0,012645	0,014769	0,003123	0,011865	0,011072	0,008039	-0,02492
1	3	0,798509	8,6E-08	0,037638	0,000306	1,67E-05	0,042358	0,000124	0,012758	-0,0614
1	3	0,723465	0,000512	0,152997	0,005347	0,006335	0,000807	0,000606	0,005889	-0,15548
1	3	0,862853	0,000978	0,005599	0,042828	0,04664	0,001478	8,02E-05	0,006825	-0,0432
1	3	0,668682	0,0014	0,115922	0,080914	0,00374	6,72E-06	0,015255	0,000688	-0,19274
1	3	0,645658	0,003383	0,007938	0,149116	0,000145	0,033593	0,001053	4,3E-06	-0,16094
1	3	0,942693	0,000381	0,00066	0,008254	0,025407	0,002479	0	6,12E-06	-0,00991
1	3	0,603148	2,76E-07	0,012222	0,062366	0,028972	0,018596	0,002342	0,004975	-0,07772
1	3	0,442206	0,094756	0,156834	0,014115	0,009482	0,016925	0,013498	0,00662	-0,07159
1	3	0,462373	0,125201	0,015233	0,008482	0,005831	0,017681	0,000582	0,026636	0,090379
1	3	0,49722	0,250904	0,013999	0,066267	0,000654	0,002511	0,009505	0,035246	0,174856
1	3	0,798085	0,000401	0,035947	0,00227	0,001917	0,000441	0,00459	0,000165	-0,0378
1	3	0,364944	0,279849	0,000504	0,006925	0,001914	0,002154	0,000827	0,000349	0,27161
1	3	0,909276	0,000264	0,003281	0,003153	0,001944	0,002575	0,000671	0,001108	-0,00673
1	3	0,51397	0,186537	0,000385	0,149651	0,002697	0,013243	0,010128	0,000757	0,036199
1	3	0,500069	0,064704	0,065614	0,005252	0,002474	0,013429	0,01278	0,005099	-0,02622
1	3	0,438645	0,001442	0,092126	0,208177	0,00088	0	5,64E-07	0,005679	-0,28486
1	3	0,447285	0,064642	0,005788	0,01184	0,068635	0,003833	0,025949	0,002525	0,036724
1	3	0,632206	0,014052	8,19E-06	0,026187	0,009788	0,002074	0,011261	2,11E-05	-0,02126
1	3	0,68324	0,053696	0,061258	0,012924	0,02617	0,00064	0,00455	0,001062	-0,01322
1	3	0,782852	0	0,000565	0,208987	0,003602	0	0,000209	0,004753	-0,20919
1	3	0,205207	0,000194	0,711223	0,001463	0,000647	0,00093	0,007669	0,000832	-0,71614
1	3	0,722357	0,000188	0,182439	0,021039	0,010245	0,009055	0,004475	0,001595	-0,20333
1	3	0,307235	0	0,034604	0,047699	1,79E-05	0,348818	0,002391	0,000206	-0,39081
1	3	0,456314	0,078646	0,036695	0,012462	0,009036	0,027244	0,000242	0,010697	0,011024
1	3	0,524588	0,025972	0,073715	0,016626	0,000319	0,039002	0,010086	0,011353	-0,07856
1	3	0,30596	0,040424	0,050896	0,001067	2,61E-07	0,358122	0,00011	0,00036	-0,34186
1	3	0,526787	0,164578	0,004497	0,000135	0,006927	0,025585	0,000193	0,003764	0,134507
1	3	0,709227	0,032335	0,016664	0,016202	0,003578	1,37E-06	0,01307	0,004744	-0,01089
1	3	0,920871	0	0,000427	0,000221	0	0	0,000576	0	-0,00058
1	3	0,324879	0,466624	0	5E-09	0	0,000212	0,002858	0,005631	0,463766
1	3	0,842951	0,002078	0,033368	0,010392	0,031882	0,012291	0,067331	0,011002	-0,07515
1	3	0,492644	5,63E-07	0,002695	0,004048	0,051469	0,008125	0,000138	6,32E-05	-0,00825
1	3	0,351778	0,310098	0,018949	0	0,020282	0,000126	0,005793	0,004048	0,290985
1	3	0,878965	0,025873	0,001049	0,032547	0,000201	0,001293	0,019787	0,014802	-0,00667
1	3	0,509485	4,82E-06	0,248784	0,000223	6,27E-05	0,000101	0,000216	2,9E-05	-0,24878
1	3	0,239193	0,531286	0,004282	1,06E-05	0,000311	0,000335	0	0,00391	0,526695
1	3	0,576386	0,021953	0,040381	0,029126	0,004375	0,034588	0,003852	0,009479	-0,07379
1	3	0,351778	0,005838	0,187615	0,119566	0,00086	0,000586	0,013284	0,001366	0,295911
1	3	0,370913	0,241193	0,018949	0,001656	0,002971	0,004131	0,001181	0,000721	0,216709
1	3	0,790573	0,005114	0,001507	0,239296	0,003355	0,034588	0,000579	0,013094	0,240551
1	4	0,557986	0,067064	0,032605	0,008656	0,005447	0,012203	5,75E-06	0,001438	0,022728
1	4	0,987632	0	0	0,001508	0,000521	0	0	4,03E-05	-0,00151

1	4	0,992381	9,81E-07	0,006259	0	0,001701	4,1E-07	9,33E-05	0,000694	-0,00626
1	4	0,744259	7E-09	0,042171	0,039739	0	6E-08	0,003011	0,000241	-0,0671
1	4	0,490832	0,051055	0,007274	0,002038	4,8E-07	0	0,019749	4,01E-05	0,025687
1	4	0,857906	0,003149	0,04117	0,002262	0,018212	0,004659	0,011977	0,00404	-0,04692
1	4	0,448033	0	0,372447	0,044786	6,59E-05	0,005043	0,009219	0,018379	-0,41124
1	4	0,75631	1E-07	0,135721	0,003714	0,000986	0,016414	0,005598	0,000222	-0,14325
1	4	0,532121	0,005114	0,004057	2,94E-06	0,030075	0,009244	0,001738	0,002655	-0,00819
1	4	0,911144	1E-09	0,016456	0,002994	0,000278	0,005799	0,01437	0,003182	-0,03122
1	4	0,654045	0,077061	0,006379	0,000558	0,011656	0,03372	0,000585	0,047378	0,038662
1	4	0,733979	1,91E-05	0,121549	0,003256	0,002454	0,00314	0	0,001289	-0,12418
1	4	0,909539	0,000564	0,024863	0,00422	0,020723	0,002111	0,000227	0,004495	-0,02936
1	4	0,756862	0,000454	0,058415	0,045361	0,012218	0,001844	0,000369	0,004067	-0,10208
1	4	0,608541	0	0,008689	0,114145	0,035623	0,006067	0,000266	0,020272	-0,11905
1	4	0,943732	1,04E-06	7,73E-05	0,0003	0,0366	0,031081	0	0,000337	-0,03115
1	4	0,585392	0	0,017764	0,081047	0,01413	0,021245	0,04799	0,004156	-0,14674
1	4	0,611275	0,121346	0,014457	0,023787	0,00191	0,003949	0,001476	0,00551	0,083621
1	4	0,537847	0,031123	0,014423	0,007792	0,021087	0,005048	0,000906	0,042992	0,010584
1	4	0,712931	0,022242	0,067156	0,025888	0,012238	0,001539	0,011542	0,041041	-0,06431
1	4	0,601906	0,000802	0,044543	0,044067	3,7E-08	0,023608	0,000374	0,018132	-0,08322
1	4	0,700887	2,19E-07	0,022086	0,027626	0,027015	0,004123	0,127289	0,000432	-0,15322
1	4	0,902241	0	0,027971	0,000352	0,003187	0,001406	0	0,002184	-0,02831
1	4	0,819633	0,079336	0,012948	0,00707	0,002989	0,001452	0,000658	0,001667	0,059876
1	4	0,487171	0,111658	0,000419	0,122918	0,001967	0,003297	0,004543	0,000543	-0,01256
1	4	0,576386	0,021953	0,040381	0,029126	0,004375	0,034588	0,003852	0,009479	-0,07379
1	4	0,409413	0,005838	0,187615	0,119566	0,00086	0,000586	0,013284	0,001366	-0,29591
1	4	0,370913	0,241193	0,023385	0,001656	0,002971	0,004131	0,001181	0,000721	0,216709
1	4	0,62669	0,011214	0,001096	0,076234	0,003015	0,000854	0,063973	0,002483	-0,12704
1	4	0,631282	0,034745	0,076743	0,004935	0,024998	0,006471	0,011583	0,001687	-0,05671
1	4	0,790573	0	0,001507	0,239296	0,003355	0	0,000579	0,013094	-0,24055
1	4	0,398283	0,05682	0,321079	4,79E-05	0,007649	0,024297	0,011377	0,002023	-0,28685
1	4	0,75896	1,54E-06	0,172202	0,002676	0,002556	0,006085	0,014258	0,001103	-0,17793
1	4	0,576444	0,001844	0,021885	0,025715	0,005039	0,087344	0,001911	7,44E-05	-0,11857
1	4	0,540365	0,01263	0,020765	0,00299	0,02367	0,049883	0,000803	0,001223	-0,05332
1	4	0,586553	0,024264	0,124288	0,0572	0,001272	0,029491	0,000775	0,001108	-0,13951
1	4	0,407764	5,52E-06	0,064911	0,000605	0	0,148667	0,000411	6E-09	-0,17919
1	4	0,658898	0,001829	0,064196	0,010717	0,004198	0,032769	0,007757	0,00078	-0,09877
1	4	0,613396	0,050751	0,068162	0,034924	0,00034	0	0,053334	0,003086	-0,08509
1	4	0,338236	0,156766	0,011628	0,001101	0,051608	0,252056	0,00146	0,000445	-0,10728
1	4	0,859242	0	0,011631	0,002453	0	0,007943	0,001189	0,000108	-0,02022
1	4	0,566899	0,010687	0,013096	0	0	0	0,195133	0,000115	-0,18445
1	4	0,876447	0,01696	0,037643	0,002319	0,012175	0,019666	0,014103	0,002685	-0,04661
1	4	0,499598	1,46E-07	0,012028	0,00105	0,014444	0,042277	3,58E-05	1,64E-05	-0,04228
1	4	0,346977	0,421627	0,001443	0	0,002585	0,00024	0,000358	0,008368	0,420184
1	4	0,886853	0,043477	0,000396	0,03729	7,6E-05	0,000488	0,030986	0,015226	0,006187
1	4	0,386371	1,77E-06	0,438023	8,21E-05	2,62E-05	3,72E-05	0,001663	0,000467	-0,43802
1	4	0,202348	0,596972	0,027265	1,47E-06	4,3E-05	4,63E-05	0	0,00059	0,569706
1	4	0,457225	0,199058	0,143316	0,025554	0,014815	0,0111	0,028094	0,004544	-0,00801
1	4	0,658313	0,070165	0,03759	0,007366	0,011683	0,016869	0,008249	0,011133	-0,05342

1	5	0,611356	0,013424	0,044423	0,004901	0,00521	0,004713	0,001378	0,002179	-0,03231
1	5	0,893869	0,063036	6E-05	6,11E-06	0,0003	6,08E-05	0,002466	4,52E-05	0,060518
1	5	0,993521	6,38E-05	0,002699	0,000226	0,0007	5,57E-05	5,55E-05	0,000281	-0,00271
1	5	0,614204	0,000606	0,178158	0,027013	0,000952	0	0,013468	0,001001	-0,1978
1	5	0,403126	0,127105	0,02614	4,6E-05	0,000204	0,000446	0,11176	0,000579	0,011175
1	5	0,954705	0,0004	0,000259	0,004114	0,025641	0,000103	0,000678	0,000724	-0,004
1	5	0,478884	0	0,36751	0,043325	0,000173	0,002468	0,000967	0,000823	-0,39614
1	5	0,667201	0,003894	0,244873	0,000518	0,000112	0,005689	0,029358	0,00221	-0,24098
1	5	0,318092	0,367591	0,003076	0,000632	0,069428	0,004551	0,000729	0,000511	0,359964
1	5	0,883174	0,010737	0,003505	0,002175	0,001581	0,007591	0,022867	0,016551	-0,02265
1	5	0,830798	0	0,013913	0,002801	0,004499	0,013693	0,001353	0,006952	-0,02699
1	5	0,893362	3,58E-05	0,01236	0,001872	0,001526	0,028341	0,000393	0,002133	-0,03919
1	5	0,826315	0,009796	0,035315	0,017911	0,024021	0,006344	0,007063	0,002006	-0,04635
1	5	0,61372	4,66E-05	0,203272	0,100602	0,002901	0,008189	0,00151	0,001097	-0,28872
1	5	0,586159	0	0,006451	0,112536	0,004167	0,001948	0,001645	0,01586	-0,11673
1	5	0,945698	0	0,000861	0,002421	0,009634	0,04859	0,000412	0,000309	-0,04885
1	5	0,634851	0	0,023811	0,056414	0,02965	0,011947	0,000234	0,000546	-0,08371
1	5	0,552853	0,130105	0,017237	0,005775	0,011774	4,09E-05	0,00807	0,004818	0,107379
1	5	0,508362	0,043678	0,003412	0,03216	0,000877	0,012352	0,000333	0,01055	0,007895
1	5	0,6529	0,13869	0,029977	0,021886	0,00627	0,000804	0,003276	0,106805	0,090253
1	5	0,575802	8,5E-06	0,067517	0,01105	0	0,022149	1,17E-06	0,010186	-0,07624
1	5	0,318295	0,457791	0,011175	0,002509	0,01249	0,012439	0,024105	0,003502	0,42379
1	5	0,841399	0,020269	0,017032	0,000917	0	7,89E-06	0,000312	0,002923	0,003057
1	5	0,843133	0,081068	0,000984	0,003105	0,001231	0,002428	0,001476	0,004433	0,075034
1	5	0,60833	0,048115	0,000379	0,026002	0,029449	0,000254	0,007733	0,009353	0,015999
1	5	0,573371	0,050494	0,001878	0,082079	0,003459	0,001981	0,078041	0,00082	-0,074
1	5	0,450517	6,34E-05	0,111435	0,065392	0,001306	0,008437	0,021831	0,029522	-0,17608
1	5	0,363073	0,216876	0,073678	0,003491	0,004557	0,002271	0,000381	0,002738	0,142259
1	5	0,535449	0,01992	0,002138	0,14913	0,007228	0,00134	0,006177	3,43E-06	-0,13542
1	5	0,564158	0,00443	0,076778	0,001506	0,010447	0,047416	0,008589	0,006193	-0,11779
1	5	0,793429	0	0,004287	0,243034	1,3E-08	0	0,001413	0,002561	-0,24376
1	5	0,681717	0,000401	0,019904	0,010472	0,008348	0,006104	0,000696	0,001372	-0,02862
1	5	0,719528	0	0,143569	0,072543	0,002043	0,001212	0,018865	0,000382	-0,20725
1	5	0,416798	0	0,078122	0,001725	0,069032	0,125937	0,001257	1,84E-05	-0,17755
1	5	0,445521	0,011938	0,045597	0,035608	0,033186	0,054221	0,002366	0,014709	-0,09777
1	5	0,509616	0,04919	0,024013	0,010392	0,000102	0,061941	0,00141	0,007756	-0,03171
1	5	0,247612	0,068451	0,073759	0,00019	0,016128	0,392858	0,003917	0,00218	-0,3693
1	5	0,5561	0,083651	0,022865	4,69E-07	0,001844	0,025643	0,005443	0,001051	0,031455
1	5	0,801437	0,033497	0,028559	0,00252	0,000953	0,000629	0,023219	0,00045	-0,01015
1	5	0,38582	0,411936	0,002677	0,003276	0,006694	0,005324	0,023782	0,006363	0,382763
1	5	0,919771	0	0,002711	0,005445	0,016115	0	0	5,15E-05	-0,00555
1	5	0,651553	0	0,013036	0,028424	0	0	0,046072	9,94E-05	-0,07161
1	5	0,873545	0,000991	0,022666	0,009306	0,003638	0,009253	0,052876	1,19E-05	-0,06613
1	5	0,508381	4,8E-08	0,012152	0,000347	0,004771	0,080992	1,18E-05	5,41E-06	-0,08099
1	5	0,315211	0,470517	0,000247	0	0,000442	9,59E-05	6,12E-05	0,00593	0,470271
1	5	0,696417	0,014967	0,024276	0,064484	1,38E-05	8,86E-05	0,036182	0,008513	-0,05
1	5	0,357534	8,03E-07	0,46667	3,72E-05	1,19E-05	1,68E-05	0,002255	0,000378	-0,46667
1	5	0,249447	0,501715	0,016678	4,49E-07	1,32E-05	1,42E-05	0	0,000181	0,485038

1	5	0,648833	0,711936	0,178503	0,000123	0,001425	0,010097	0,000711	0,000368	0,188036
1	5	0,555872	0,008407	0,211482	0,005174	0,000422	0,000373	0,009852	0,004091	-0,20876
1	6	0,572486	0,002993	0,067361	0,0035	0,004314	0,003543	0,03874	0,000789	-0,09566
1	6	0,961007	0,00072	0,000194	1,5E-05	0,000504	0	0,032143	0,000555	-0,03153
1	6	0,961893	0,00042	0,028163	8,69E-07	0,016469	2,87E-05	0,016088	0,000461	-0,04079
1	6	0,629879	0,003636	0,159874	0,003072	0,001679	0,00484	0,0048	0,002357	-0,16078
1	6	0,41478	0,284829	0,031575	0,0007	0,000892	0,000339	0,045282	0,000158	0,238056
1	6	0,868964	0,007171	0,039626	0,006263	0,002241	0,00121	0,011345	0,00069	-0,03435
1	6	0,536928	0	0,229305	0,120593	0,001025	0,001353	0,004023	0,001824	-0,33165
1	6	0,751552	1E-09	0,027393	3,85E-06	2,07E-05	0,018134	9,8E-08	0,003457	-0,04288
1	6	0,486301	0,032355	0,031964	2,75E-05	0,008799	0,002391	0,01273	0,006701	-0,00148
1	6	0,870762	0,010922	0,013943	0,001415	0,000628	0,008476	0,010968	0,024201	-0,01215
1	6	0,758313	0,000165	0,03759	0,007366	0,011683	0,016869	0,008249	0,011133	-0,05342
1	6	0,804793	0,000157	0,059125	0,006792	0,000537	0,010668	6,78E-05	0,004715	-0,07001
1	6	0,851344	0,001975	0,013661	0,046558	0,020407	0,007935	0,000553	0,000501	-0,06236
1	6	0,744809	0,00082	0,107819	0,023156	0,007045	0,003834	0,000313	0,004532	-0,12981
1	6	0,587802	0	0,061506	0,086882	1,58E-05	0,02177	0,000579	0,014968	-0,13949
1	6	0,954974	0	2,05E-05	0,002961	0,054938	0,001243	0	7E-05	-0,00364
1	6	0,834362	0	0,026267	0,00324	0,034201	0,004546	1E-09	0,000182	-0,03365
1	6	0,539717	0,104306	0,090513	0,005835	0,004525	0,002803	0,016992	0,016964	0,008757
1	6	0,538501	0,026948	0,011546	0,014783	0,001226	0,023573	0,001414	0	-0,00906
1	6	0,731824	7,89E-07	0,089255	0,035394	0,001168	0,002969	0,003505	0,053121	-0,11159
1	6	0,702252	5,46E-05	0,0841	0,000376	0,008792	0	0	0,000251	-0,08409
1	6	0,437959	0,220825	0,032254	0,000694	0,009335	0,004868	0,072582	0,003694	0,120232
1	6	0,556005	0,339921	0,004571	0,001669	0	0,001138	2,23E-06	0,006604	0,333204
1	6	0,855661	3,71E-05	0,027551	0,000707	0,00743	0,000589	0,001128	0,002353	-0,02834
1	6	0,565731	0,111346	0,000186	0,068739	0,01078	0,00217	0,004868	0,000296	0,040764
1	6	0,54442	0,055345	0,017297	0,026139	0,001307	0,035483	0,03819	0,007735	-0,02539
1	6	0,48136	0,001268	0,063326	0,031021	0,001082	0,027507	0,049197	0,012746	-0,11667
1	6	0,414913	0,082134	0,116519	0,001563	0,01672	0,012776	0,002695	0,002099	-0,04118
1	6	0,528008	0,079608	0,005935	0,015964	0,004389	0,001714	0,036255	0,000241	0,022161
1	6	0,790636	1,47E-07	2,32E-06	0,005689	0,001606	0,036662	0,004849	1,53E-07	-0,03866
1	6	0,815324	0	1,96E-05	0,185378	0,000485	0,00142	0,00019	0,003224	-0,18556
1	6	0,648833	1E-09	0,178503	0,000123	0,001425	0,010097	0,000711	0,000368	-0,18804
1	6	0,791918	0	0,097538	0,02033	0,007895	0,001642	0,005699	0,00798	-0,11768
1	6	0,29216	0,014063	0	9,18E-05	0,002909	0,444378	0,001472	2,96E-06	-0,43032
1	6	0,431913	0,06382	0,071159	0,002533	0,027705	0,023145	0,010448	0,002217	-0,03059
1	6	0,590838	0,020242	0,040696	0,122802	0,000461	0,036098	0,003605	0,003565	-0,13171
1	6	0,30364	0,074691	0,029248	0,000622	0,011218	0,319619	0,001817	0,00685	-0,27189
1	6	0,670012	0	0,034978	0,005157	0,004275	0,003985	0,000837	5,12E-05	-0,03938
1	6	0,705216	0,01969	0,021442	0,015336	0,001642	0,000143	0,026855	5,56E-05	-0,03172
1	6	0,412723	0,186201	0,000308	0,001467	0,000757	0,152546	0,035439	0,001278	-0,00124
1	6	0,957178	0	0,001829	0,002219	0,005118	0	0	0,003716	-0,00355
1	6	0,557778	0,094468	8,63E-07	1,38E-06	0,00451	0	0,017298	0	0,07717
1	6	0,870844	0,006995	0,007426	0,015311	0,010554	0,00639	0,010771	0,004249	-0,02307
1	6	0,520044	9,44E-05	0,010046	9,02E-05	0,00124	0,126528	3,07E-06	1,41E-06	-0,12643
1	6	0,346895	0,35092	0,009799	0,013171	0,003715	0,000264	0,001759	0,004253	0,328315
1	6	0,85184	0,010411	5,02E-05	0,052815	9,63E-06	6,19E-05	0,004538	0,002352	-0,0424

1	6	0,371812	4,79E-07	0,413412	2,22E-05	7,07E-06	1E-05	0,002227	0,000226	-0,41341
1	6	0,312701	0,375051	0,013402	1,13E-07	3,31E-06	3,56E-06	0	0,000636	0,361649
1	6	0,423236	0,000192	0,041277	0,326597	0	0,000247	0,000858	0,000358	0,107431
1	6	0,557778	0,019813	8,63E-07	1,38E-06	0,00451	0	0,017298	0,004852	0,07717
1	7	0,524599	0,000438	0,033012	0,000948	0,000839	0,003015	0,076039	0,000402	-0,09404
1	7	0,79255	0,162071	0,000248	0,002148	0,002348	0,000291	0,03846	0,000815	0,122601
1	7	0,985854	2,48E-05	0,023217	0,000144	3,99E-05	6,88E-06	0,000862	0,000546	-0,02361
1	7	0,655872	0,005407	0,211482	0,005174	0,000422	0,000373	0,009852	0,004091	-0,20876
1	7	0,577418	0,120339	0,001399	2,26E-06	0,00061	0,000247	2,67E-07	2,01E-07	0,118695
1	7	0,959821	0,001105	0,006117	0,001295	0,010906	0,000532	0,000907	0,000304	-0,00636
1	7	0,426725	0	0,372729	0,146186	0,001254	0,000392	0	0,002857	-0,47797
1	7	0,651501	0,018813	0,126025	0,000583	0,000607	0,002767	0,017672	0,005539	-0,10743
1	7	0,321695	0,397237	0,002574	0,000456	0,00031	0,001433	0,003461	0,004237	0,391527
1	7	0,898205	0,001709	0,018112	0,002131	0,000706	0,006972	0,014745	0,008034	-0,02445
1	7	0,773019	0,003248	0,058864	0,028645	0,00266	0,019651	0,02991	0,00206	-0,07402
1	7	0,645018	0,000131	0,153949	0,001438	0,001098	0,002716	2,35E-05	0,0027	-0,15607
1	7	0,654952	0,003822	0,172107	0,003409	0,058414	0,003311	0,01269	3,37E-05	-0,17027
1	7	0,799107	0,000186	0,055455	0,020465	0,017134	0,006183	0,000361	0,006989	-0,07257
1	7	0,423236	0,000192	0,041277	0,326597	0	0,001807	0,000858	0,000358	-0,32934
1	7	0,952731	0,005902	0	0,005145	0,017654	0,010738	0,001214	0,000337	-0,00984
1	7	0,787623	0	0,040806	0,02289	0,017573	0,006924	0,000501	2,63E-05	-0,06022
1	7	0,477225	0,159058	0,143316	0,025554	0,014815	0,0111	0,028094	0,009544	-0,00801
1	7	0,530666	0,035673	0,025115	0,03981	0,012351	0,016378	0,001619	0,014664	-0,02935
1	7	0,648781	0,031431	0,082442	0,031318	0,008243	0,001615	0,010764	0,013151	-0,07802
1	7	0,43125	7,12E-06	0,287762	0,000786	0,002177	9,79E-06	0,000103	0	-0,28775
1	7	0,461227	0,060876	0,017181	0,003079	0,039242	0,049541	0,056938	0,006706	-0,05249
1	7	0,847653	0,00033	0,011784	0,000458	0,00205	3,4E-05	0	0,006678	-0,01191
1	7	0,972929	0	0,000112	1,81E-05	0,003016	0,00032	7,9E-08	7,09E-05	-0,0004
1	7	0,569465	0,044143	0,013587	1,39E-06	0,054951	0,010078	0,004755	0,073346	0,025706
1	7	0,553421	0,092477	0,030726	0,006015	1,09E-06	0,000485	0,016688	0,023649	0,047391
1	7	0,566757	0,022002	0,147052	0,005466	0,003253	1,5E-08	0,054768	0,006085	-0,12884
1	7	0,437973	0,008723	0,118273	0,00428	0,001372	0,043094	7,5E-05	0,004489	-0,12517
1	7	0,598483	0,012495	0,022932	3,02E-05	0,011168	0,003863	0,010409	0,000253	-0,01852
1	7	0,829722	1,37E-05	3,1E-05	0,015461	0,018029	0,000233	0,005176	0,000144	-0,01679
1	7	0,712946	0	0,012678	0,294235	0,001638	3,71E-05	0,005797	0,000176	-0,29448
1	7	0,392021	0	0,472114	7,66E-07	7,25E-06	0,00143	0,000572	2,31E-05	-0,47211
1	7	0,758589	0,003045	0,125188	0,021229	0,004936	0,006833	0,014994	0,00226	-0,14238
1	7	0,393892	1,57E-07	0,052819	0,061461	4,99E-06	0,226207	0,004645	0,000787	-0,28981
1	7	0,428727	0,082276	0,010415	0,003	0,023398	0,057634	0,000459	0,000944	0,019191
1	7	0,519263	0,004297	0,216794	0,008945	0,003444	0,004673	0,014291	0,028555	-0,21686
1	7	0,319734	0,008295	0,086095	0,007174	0,002854	0,32139	0,000217	0,001421	-0,37571
1	7	0,621104	0,001277	0,053158	0,002054	0,005876	0,008779	0,010344	0,003077	-0,06527
1	7	0,759375	0,064993	0,023851	1,72E-06	0,006172	0,001809	6,63E-05	0,00699	0,039683
1	7	0,481533	0,060715	0,010298	0,001614	0,106258	0,024018	0,027089	4,6E-05	0,008291
1	7	0,946829	0,000115	0,000277	0,005379	0,002149	0	0	1,02E-07	-0,00553
1	7	0,685708	0,058895	1E-09	0,006644	0,000825	0,000903	3,99E-05	0,001946	0,052143
1	7	0,861545	0,032365	0,01625	0,001439	0,005225	1,56E-06	0,010322	0,007294	0,015947
1	7	0,503332	0,001499	0,003793	1,84E-05	0,000253	0,161948	6,28E-07	2,88E-07	-0,16045

1	7	0,481649	0,088004	0,000772	0,045324	0,001716	0,000824	0,000318	0,003159	0,04268
1	7	0,676125	0,00293	0,031251	0,038374	5,88E-05	1,43E-05	0,051662	0,011631	-0,06368
1	7	0,398896	2,81E-07	0,343983	1,3E-05	4,69E-06	5,91E-06	0,001737	0,000133	-0,34398
1	7	0,394624	0,211451	0,002558	5,48E-06	6,3E-07	6,79E-07	0	0,001397	0,208893
1	7	0,600881	1,99E-05	0,052498	0,013814	0,002993	0,004033	0,008143	0,014357	0,062139
1	7	0,800881	1,99E-05	0,033955	0,013814	0,002993	0,800881	1,99E-05	0,052498	0,013814
1	8	0,567861	1,46E-06	0,088661	0,004273	0,001523	9,14E-05	0,025398	0,000186	-0,09918
1	8	0,989197	0	0,001107	0,00235	0,000459	6,96E-06	0,003774	0,000351	-0,00589
1	8	0,987338	0,000158	0,016596	4,15E-05	0,000787	1,2E-05	0,000797	8,63E-05	-0,0169
1	8	0,627574	0,013615	0,135945	0,017532	0,001907	0,000292	0,000778	0,008535	-0,13069
1	8	0,544246	0,110213	0,009468	0	0	1,17E-06	0,004756	0,000231	0,097423
1	8	0,933532	0,000908	0,004163	0,004974	0,036307	0,004502	0,001667	0,001982	-0,00897
1	8	0,513111	3,13E-05	0,30696	0,082462	0,000567	0,002916	0,000614	0,000115	-0,36761
1	8	0,701651	0,003343	0,130286	0,005329	0,000642	0,029834	0,00632	0,000968	-0,13673
1	8	0,477363	0,080103	0,003036	0,000599	2,95E-06	0,008756	0,000189	0,003281	0,069135
1	8	0,920289	7,32E-06	0,010601	0,002369	0,00043	0,003278	0,010786	0,006293	-0,01959
1	8	0,800881	1,99E-05	0,052498	0,013814	0,002993	0,800881	1,99E-05	0,052498	0,013814
1	8	0,898794	1,26E-05	0,02343	0,003428	0,009124	0,004033	6E-09	0,000722	-0,02926
1	8	0,838222	0,00016	0,017705	0,008506	0,087781	0,004535	2,16E-05	0,005014	-0,02606
1	8	0,78094	3,41E-05	0,06183	0,043355	0,009821	0,005803	0,000249	0,002884	-0,0978
1	8	0,485968	0,004284	0,033955	0,173022	0	0,009173	0,001001	0,000846	-0,18667
1	8	0,95919	0,000199	0,000187	0,000272	0,027285	0,017765	2,2E-05	0,000446	-0,01757
1	8	0,846418	0	0,008773	0,009026	0,012415	0,00244	1,7E-08	0,000457	-0,01854
1	8	0,531164	0,118507	0,035351	0,026103	0,009594	0,01746	0,017939	0,008762	0,057939
1	8	0,508696	0,023646	0,02228	0,016616	0,004445	0,028156	0,002097	0,011866	-0,01932
1	8	0,501244	0,247693	0,029056	0,074328	0,001086	0,004514	0,001934	0,020083	0,147768
1	8	0,080812	0,003293	0,846367	1E-09	0	0,033518	0,069059	0	-0,84307
1	8	0,26687	0,487514	0,006087	0,008813	2,52E-05	0,003316	0,066506	0,002025	0,409499
1	8	0,877657	9,28E-05	0,020156	0,001336	0,002654	0,005465	0,000105	0,001587	-0,0232
1	8	0,968691	0	0,001157	0,000524	0,000907	0,001177	6,26E-05	0,003926	-0,00231
1	8	0,592465	0,041968	0,001589	0,018151	0,032883	0,01598	0,008274	0,011248	0,006969
1	8	0,494217	0,167635	2,7E-05	0,014928	0,002402	0,003614	0,093457	0,015576	0,068103
1	8	0,453998	0,011966	0,11802	0,027983	0,007134	0,016142	0,132898	0,004806	-0,18606
1	8	0,425983	0,144906	0,007553	0,002449	0,004494	0,012079	9,44E-05	0,001793	0,125774
1	8	0,53976	0,049264	0,004097	0,029987	0,008828	0,006274	0,029058	0,001496	-0,00581
1	8	0,663873	0,021269	0,057806	0,003157	0,033275	0,01631	0,024409	0,005357	-0,07226
1	8	0,742477	0	0,002076	0,272223	0,000553	0,001063	0,002787	0,002103	-0,27395
1	8	0,341696	0	0,560699	3,4E-05	0	0,000479	0,000596	0,000179	-0,56088
1	8	0,280645	0,001011	0,535859	0,000299	0,017171	0,006633	0,029095	0,000599	-0,53485
1	8	0,375061	0	0,112401	0,196189	0	0,002733	0,01079	0,000293	-0,25341
1	8	0,378837	0,239388	0,034255	0,00697	0,014429	0,036625	0,001968	0,001263	0,170221
1	8	0,63332	0,00082	0,042539	0,023976	0,000172	0,00917	4,14E-05	0,002575	-0,06499
1	8	0,329987	0,100597	0,090645	0,000569	0,001519	0,19639	0,000331	0,003823	-0,18586
1	8	0,611998	0,047689	0,00765	0,000981	0,011505	0,006999	0,002146	0,002002	0,031521
1	8	0,642269	0,003856	0,070041	0,000378	0,00078	4,1E-06	0,00118	0,000837	-0,06761
1	8	0,587233	0,069387	0,006194	0,010334	0,032384	0,005504	0,010947	0,001876	0,043517
1	8	0,950899	1,07E-05	0,002159	0,013454	0,002476	0,000139	1,58E-05	0,000772	-0,01477
1	8	0,745341	0	0,000664	0,063653	0,002444	0,002072	0,01008	0,001034	-0,06384

1	8	0,937486	0,000337	0,002491	0,001094	0,021095	0	0,004135	0,004076	-0,00627
1	8	0,464127	0,002866	0,015518	4,42E-06	6,07E-05	0,173215	1,5E-07	6,9E-08	-0,17035
1	8	0,510433	0,029819	0,000255	0,016606	0,000568	0,000916	0,000105	0,001118	0,013213
1	8	0,727406	0,001456	0,012363	0,016285	5,63E-05	6,64E-06	0,04134	0,008033	-0,04022
1	8	0,484989	8,7E-08	0,205477	4,01E-06	3,49E-05	1,82E-06	0,000603	4,08E-05	-0,20548
1	8	0,420289	0,159874	0,000529	3,27E-06	0,000447	1,4E-07	0	0,000482	0,159346
1	8	0,888118	0,004191	0,021368	0,001404	0,056829	0,001719	0,000598	0,001257	0,007447
1	8	0,478667	5,34E-05	0,04845	0,003504	0,000859	0,013025	0,009588	0,037636	-0,06039
1	9	0,576081	2,57E-07	0,102212	0,001843	0,004268	0,008026	0,019168	0,002958	-0,10631
1	9	0,980931	0	8E-09	0,004284	0,000921	0	0,017794	4,8E-05	-0,01997
1	9	0,985867	2,52E-06	0,021368	4,15E-05	0,000382	4,81E-06	0,000767	0,000882	-0,02191
1	9	0,742327	0,000143	0,12959	0,00446	0,00053	0,007473	0,00349	0,007356	-0,14028
1	9	0,488795	0	0,029051	0,004813	0,000605	9,53E-05	0,081402	0,001018	-0,09114
1	9	0,888118	0,004191	0,010275	0,001404	0,056829	0,001719	0,000598	0,000658	-0,00745
1	9	0,581768	0	0,246503	0,077168	8,81E-05	0,000897	0	0,001257	-0,31079
1	9	0,77093	2E-09	0,125159	0,002854	0,000272	0,011638	0,007857	1,84E-05	-0,12821
1	9	0,478667	0	0,04845	0,003504	0,000859	0,013025	0,009588	0,037636	-0,06039
1	9	0,899954	0	0,02221	0,005992	0,004735	0,004006	0,011004	0,000266	-0,02981
1	9	0,814886	0	0,07213	0,034236	0,001388	0,012195	0,021913	0,000835	-0,08292
1	9	0,831595	0	0	0,030568	5,15E-06	4,3E-06	0	3,36E-06	-0,03057
1	9	0,786726	0,000436	0,020167	0,001137	0,152493	0,009891	0,000322	0,00075	-0,02883
1	9	0,831126	0	0,04969	0,018937	0,003632	0,00109	0,000754	0,003892	-0,06237
1	9	0,5371	0,001122	0,053068	0,112795	0	0,021096	0,002041	0,007448	-0,14727
1	9	0,928607	0	0,000153	0,000683	0,010328	0,092163	0,001799	0,001021	-0,0941
1	9	0,773215	0	0,023691	0,0379	0,02779	0,006874	0,000607	0,000107	-0,05705
1	9	0,612247	9,23E-05	0,086911	0,026399	0,001448	0,027144	0,076358	0,004165	-0,11929
1	9	0,504862	0,057093	0,065405	0,005392	0,002493	0,002272	0,004115	0,045637	-0,01051
1	9	0,697891	0,006775	0,015774	0,001278	0,020881	0,006438	0,006526	0,003315	-0,01386
1	9	0,490094	0,001611	0,205987	0,013914	0,002146	0,004769	0,004316	0,01633	-0,20442
1	9	0,506933	3,64E-05	0,107575	0,005555	0,009168	0,030523	0,142135	0,005811	-0,22634
1	9	0,882866	0,004856	0,063815	0	0,001183	0,001428	0	0,004733	-0,05896
1	9	0,935265	0	0,012606	0,000585	0,003681	0,002889	0,00045	0,000952	-0,01366
1	9	0,5737	0,026751	0,000626	0,064204	0,008414	0,00065	0,000873	0,003822	-0,03808
1	9	0,602803	0,089506	0,023279	0,010188	0,000959	0,005143	0,019649	0,005039	0,0538
1	9	0,47459	0,002025	0,101547	0,069436	0	0,00783	0,06551	0,004499	-0,17405
1	9	0,487738	0,013298	0,024158	0,00096	0,000194	0,00387	0,001623	0,007052	-0,01293
1	9	0,65547	0,001398	0,001085	0,00577	0,00202	0,002593	0,026431	0,001468	-0,0307
1	9	0,646323	0,011451	0,001167	0,011213	0,020802	0,058588	0,018511	0,00301	-0,06702
1	9	0,890305	0	0,005519	0,060356	0,000765	0,000813	0,016672	0,003651	-0,07391
1	9	0,31957	0,015802	0,498423	0,002643	0,000424	0,024322	0,012374	0,016009	-0,50208
1	9	0,779995	1,32E-07	0,120343	0,02062	0,003997	0,002148	0,017352	0,001093	-0,14024
1	9	0,234394	0	0,243841	0,100798	9,17E-05	0,261983	1,58E-05	0,000862	-0,55535
1	9	0,522716	4E-09	0,012421	0,000651	0,005107	0,014798	0,00011	5,6E-08	-0,02721
1	9	0,502043	0,004241	0,221652	0,075824	0,000509	0,004497	0,007259	3,9E-07	-0,28105
1	9	0,453243	0,002513	0,066438	2,6E-08	2,16E-07	0,050485	0,004006	0,002513	-0,09245
1	9	0,662122	0,026051	0,075085	0,003716	0,005067	0,019126	0,004598	0,032719	-0,06751
1	9	0,621712	0,016672	0,027636	8,37E-07	0,004867	0,000207	0,002884	0,0004	-0,01403
1	9	0,701062	0,007925	0,006148	0,00013	0,004393	0,023261	0,023048	0,000286	-0,03837

1	9	0,869379	3,84E-05	0,034866	0,001187	3,6E-08	3,75E-07	0,00145	8,61E-07	-0,03688
1	9	0,57872	0	0,024331	0,051064	2,13E-05	2,96E-05	0,019302	0,001989	-0,06996
1	9	0,931175	4,73E-06	0,002629	0,003307	0,001813	0,000518	0,012997	0,000228	-0,0137
1	9	0,459682	0,003375	0,071498	1,44E-06	1,98E-05	0,173654	4,9E-08	2,3E-08	-0,17028
1	9	0,503574	0,034942	8,82E-05	0,005743	0,000199	0,000561	3,63E-05	0,000567	0,029199
1	9	0,765326	0,001608	0,008603	0,009859	1,8E-05	2,13E-06	0,03043	0,003652	-0,02882
1	9	0,453444	2E-09	0,305008	1E-07	1,87E-05	0,000891	7,78E-05	0,002338	-0,30501
1	9	0,391762	0,216805	0,000414	1,01E-06	0,000711	4,3E-08	0	0,001175	0,216391
1	9	0,49742	0,000653	0,001508	0,000301	0,013093	0,019639	1,37E-05	0,003463	-0,02027
1	9	0,625624	0,000653	0,221686	0,000749	0,001769	0,001303	0,001636	0,003676	-0,22124
1	10	0,670888	3,2E-08	0,053781	0,000496	0,055935	0,040351	0,029865	0,000987	-0,08581
1	10	0,96603	0	0,00142	6,21E-05	0,00075	0	0,034936	0,000679	-0,03503
1	10	0,949644	1,4E-07	0,090174	7,07E-06	0,001142	0,00023	1,97E-05	0,0012	-0,09039
1	10	0,736447	0,002552	0,097046	0,009655	0,003006	0,007268	0,023076	0,007162	-0,11459
1	10	0,438879	0,048392	0,145806	0,005657	0,000919	0,000351	0,132307	0,004032	-0,1404
1	10	0,908373	0,001407	0,051466	0,001965	0,014988	0,000789	0,001184	0,000444	-0,05042
1	10	0,398439	0	0,379705	0,132805	0,000977	0,001602	0,000709	0,003793	-0,49383
1	10	0,71158	0	0,088722	0,00177	0,000722	0,027421	3,4E-08	0,000898	-0,11476
1	10	0,49742	4,67E-05	0,001508	0,000301	0,000148	0,019639	1,37E-05	0,003463	-0,02027
1	10	0,880129	0	0,020394	0,003704	0,0008	0,00428	0,01043	0,002907	-0,02467
1	10	0,750302	0,007265	0,054197	0,011782	0,013093	0,020634	0,025315	0,003369	-0,06516
1	10	0,625624	0,000653	0,221686	0,000749	0,001769	0,001303	0,001636	0,003676	-0,22124
1	10	0,732804	0,013267	0,073567	0,000248	0,049042	0,003425	0,007816	0,001789	-0,06265
1	10	0,7798	0	0,084993	0,059966	0,004193	0,003156	0,000311	0,008976	-0,1386
1	10	0,543192	0	0,021414	0,218365	0,007505	0,024695	0,000117	0,001242	-0,23596
1	10	0,965509	0	2,66E-05	0,00428	0,025796	0,016151	2,97E-06	0,000692	-0,01843
1	10	0,811842	0	0,011837	0,00937	0,020729	0,007321	0,000135	0,000548	-0,0248
1	10	0,49827	0,077535	0,052233	0,039176	0,000723	0,027575	0,018672	0,003339	-0,02638
1	10	0,538979	0,015438	0,04401	0,026442	0,000524	0,024841	0,003523	0,010562	-0,0536
1	10	0,737789	0,006167	0,026435	0,00287	0,006022	0,00548	0,004317	0,011696	-0,02702
1	10	0,519456	0	0,214149	0,000431	0,00635	0	0	3E-09	-0,21415
1	10	0,707058	0	0,027413	0,008236	0,00031	0,035331	0,054883	0,000246	-0,09377
1	10	0,835459	0	0,10345	0,002978	0,001682	0	0,000552	0,004007	-0,10345
1	10	0,94937	0	0,01639	0,000204	0,001539	0,000883	0,000109	0,000433	-0,01639
1	10	0,510855	0,082019	0,000563	0,059052	0,017874	0,002716	0,005725	0,000891	0,018817
1	10	0,494297	0,010676	0,039437	0,004802	0	0,001273	0,089664	0,002491	-0,10876
1	10	0,415215	2,14E-05	0,106729	0,066077	1,84E-05	0,034174	0,11019	0,045303	-0,20937
1	10	0,431169	0,003338	0,190816	0,000333	0,041787	0,010397	0,000415	0,00146	-0,19152
1	10	0,625478	0,006825	0,005797	0,002219	0,024902	0,005889	0,025317	0,000267	-0,02343
1	10	0,533848	0,129231	0,01343	0,027363	0,017527	0,046872	0,001544	0,006101	0,066549
1	10	0,899147	0	0,005698	0,064594	4,44E-05	0	0,002211	0,001428	-0,06984
1	10	0,422874	0,077643	0,030196	0,000117	0,013474	0,109624	0,007382	4,04E-05	-0,05065
1	10	0,802465	0	0,067533	0,007349	0,002516	0,003127	0,009565	0,002425	-0,07454
1	10	0,293471	0	0,090333	0,007767	0,002259	0,378332	0,000997	0,000917	-0,46804
1	10	0,396593	0,160605	0,007373	0,02032	0,017403	0,034556	0,038605	0,039603	0,084293
1	10	0,34191	0	0,574085	0,006183	3,48E-05	0,001351	0,007941	0,000125	-0,57408
1	10	0,412107	0	0,094183	0	3,01E-05	0,092326	0,000413	0,001076	-0,1665
1	10	0,633859	0,004101	0,068	0,004978	0,003305	0,022727	0,006359	0,003468	-0,08764

1	10	0,698478	0,021884	0,010915	0	0,004704	1,33E-06	2E-09	0,000546	0,01097
1	10	0,641794	0,014701	0,017742	8,89E-05	0,004416	0,012682	0,022383	0,000884	-0,02814
1	10	0,867739	3,08E-05	0,058198	0,000184	0,007226	0,002231	0,002178	0	-0,05835
1	10	0,66371	0	0,029227	0,056848	0,000167	0	0,031311	0,004064	-0,08777
1	10	0,894964	0	4,03E-07	0,000778	0,000399	0,009712	0,026638	0,000252	-0,03003
1	10	0,444192	0,004688	0,062355	5,6E-07	7,7E-06	0,199762	1,9E-08	9E-09	-0,19507
1	10	0,442609	0,143302	2,73E-05	0,001781	0,000107	0,000211	1,13E-05	0,000718	0,141521
1	10	0,712216	0,018392	0,002789	0,041747	3,79E-06	4,47E-07	0,042219	0,017773	-0,02812
1	10	0,436023	0,004177	0,307016	0,008854	0,000664	0,00226	0,002181	0,002346	-0,30284
1	10	0,414244	0,172	0,01914	6,11E-07	0,000825	1,1E-08	1,45E-06	0,002339	0,15286
1	10	0,756018	0,005019	0,040349	0,00864	0,063317	0,005682	0,008731	0,00168	0,471872
1	10	0,472388	0,000809	0,023485	0,00896	0,010893	0,000577	7,32E-07	0,027169	0,25295
1	11	0,604908	9E-09	0,066626	0,000165	0,059479	0,046428	0,054841	0,000461	-0,1055
1	11	0,966542	0	0,018372	0,000201	0,014032	0	0,017301	1,56E-06	-0,0329
1	11	0,990253	9,91E-06	0,012176	2,78E-06	0,000597	5,77E-05	0,000179	0,000126	-0,01221
1	11	0,75571	0,001764	0,084114	0,017772	0,001229	0,00116	0,002611	0,006999	-0,10018
1	11	0,4017	8E-07	0,157491	0,000704	8,93E-05	6,47E-06	0,145233	0,000431	-0,19937
1	11	0,866938	0,001518	0,027708	0,024318	0,024586	0,001861	0,004304	0,000778	-0,04435
1	11	0,376281	0	0,412235	0,083813	7,82E-05	0,001638	0,000203	0,000449	-0,47197
1	11	0,509588	0,000809	0,297051	0,004296	0,003472	0,007535	0,051661	0,002649	-0,29853
1	11	0,589221	0,005139	0,0228	0,004611	0,017254	0,062223	0,008731	0,011604	-0,07594
1	11	0,896662	0,017404	0,004834	0,00194	0,000518	0,005722	0,003733	0,011397	0,006175
1	11	0,717776	0,009151	0,068903	0,023935	0,007945	0,051137	0,025148	0,006086	-0,10797
1	11	0,441839	2,21E-06	0,465747	0,000191	0,000244	0,000843	0,000206	0,002803	-0,46574
1	11	0,766018	0,005019	0,040349	0,00864	0,063317	0,005682	0,000235	0,00168	-0,04302
1	11	0,720836	0,000454	0,072308	0,102822	0,006653	0,001928	0,000985	0,003054	-0,16685
1	11	0,528007	0,030256	0,002381	0,114295	0,000783	0,000611	0,000135	0,084658	-0,08404
1	11	0,980819	0	7,86E-07	0,005744	0,012284	0,004797	0	0,000296	-0,00709
1	11	0,712308	0	0,01597	0,021102	0,023323	0,012971	0	0	-0,03813
1	11	0,469803	0,177941	0,002937	0,014753	0,008242	0,015847	0,005144	0,002647	0,147069
1	11	0,568463	0,018429	0,024945	0,021334	0,000109	0,011038	0,001892	0,035172	-0,02998
1	11	0,472388	0,283257	0,023485	0,00896	0,010893	0,000577	7,32E-07	0,027169	0,25295
1	11	0,276368	0,000361	0,50456	0	0,006878	0	0,00174	0	-0,5042
1	11	0,595614	0,144692	0,001361	0,004728	5,85E-07	0,007375	0,100771	0,000276	0,043921
1	11	0,872851	0,000228	0,080013	1,9E-05	0,000201	0,000316	0	0,001848	-0,07979
1	11	0,91652	0	0,023353	1,71E-05	0,001377	0,001037	0,000614	0,001471	-0,02397
1	11	0,604181	0,027363	0,002909	0,024481	0,040861	0,00665	0,001278	0,002933	-0,00513
1	11	0,616831	0,070558	0,02446	0,033627	0,00346	0,013455	0,019049	0,009047	0,009944
1	11	0,403415	0,004741	0,19984	0,000913	0,000699	0,056012	0,118342	0,004667	-0,2572
1	11	0,448557	0,002141	0,076533	0,005369	0,135622	0,040066	0,001211	0,000164	-0,09685
1	11	0,693216	0	0,000679	0,01152	0,006084	0,002989	0,008908	7,27E-05	-0,02064
1	11	0,56499	0,028847	0,011168	0,019257	0,047632	0,067439	0,00123	0,004695	-0,05349
1	11	0,918172	0	0,001529	0,049272	0,000122	0,001116	0,000389	0,008459	-0,04995
1	11	0,374742	0,001702	0,413034	0,001575	0,002347	0,014108	0,00559	0,000217	-0,42386
1	11	0,698966	0,001609	0,162831	0,00744	0,007219	0,008541	0,025023	0,002018	-0,16551
1	11	0,292168	0	0,001816	0,046997	0,002982	0,445316	0,003298	0,00014	-0,48091
1	11	0,361799	0,254573	0,030505	0,010152	0,006724	0,051702	0,004936	0,00185	0,180667
1	11	0,506853	0	0,287995	0,000536	1,99E-06	0,002397	0,000153	0,000151	-0,288

1	11	0,307967	0,079783	0,04517	0	7,49E-07	0,312573	0,000122	0,002356	-0,27623
1	11	0,662623	0,000206	0,043676	0,005492	0,002005	0,010305	0,010789	0,000145	-0,05555
1	11	0,687972	0,029381	0,020984	0,020425	0,007579	0,00065	0,039663	0,004175	-0,02974
1	11	0,69608	0,042321	0,072723	0,005552	0,027222	0,002909	0,047014	0,007731	-0,06173
1	11	0,924946	0	0,021642	0,002332	0,000977	1,42E-06	0	0	-0,02335
1	11	0,59066	0	0,012486	0,059477	2,68E-05	0	0,03209	4,68E-05	-0,09035
1	11	0,860389	0,011959	0,041723	0,000433	0,000561	0,002575	0,049653	0,000725	-0,0657
1	11	0,451488	0,004024	0,050161	2,21E-07	3,04E-06	0,146461	0,015364	3E-09	-0,14244
1	11	0,497869	0,087513	9,76E-06	0,000635	4,92E-05	0,000108	4,01E-06	0,003179	0,086878
1	11	0,759981	0,047506	0,010503	0,008944	2,1E-06	2,48E-07	0,010484	0,036537	0,032444
1	11	0,223993	0,00717	0,592112	0,037914	0,000217	0,002933	0,014546	0,004683	-0,58494
1	11	0,391445	0,217592	0,036826	8,2E-05	0,000559	4E-09	1,61E-06	0,001328	0,180766
1	11	0,83214	3,3E-05	0,002212	0,016394	0,019096	0,0032	0,124017	0,002312	-0,02982
1	11	0,483701	0	0,034016	0,047089	0	0	0,053901	0,000101	-0,10405
1	12	0,620258	2E-09	0,043656	0,043302	0,021529	0,018705	0,025043	0,001387	-0,07413
1	12	0,776175	0,173668	0,000372	5,12E-05	0,001435	6,66E-05	0,022496	7,81E-05	0,15087
1	12	0,995454	1,61E-05	0,003822	2,73E-05	0,001776	0,000137	0,0005	0,000913	-0,00388
1	12	0,721869	0,00415	0,148136	0,00145	0,001391	0,000124	0,001532	0,00031	-0,14552
1	12	0,538982	0,049262	0,040337	0,000258	4,1E-05	0,000207	0,019942	0,000364	0,004357
1	12	0,923658	0,00061	0,022265	0,000217	0,032611	0,000112	0,000498	0,00057	-0,02184
1	12	0,369322	0	0,377616	0,101524	1,2E-08	0,000622	0,012491	0,003574	-0,45103
1	12	0,745071	0,001855	0,144963	0,001434	0,000201	0,0032	0,02397	0,000426	-0,14472
1	12	0,517887	0,002878	0,000594	0,000119	0,004229	0,002764	0	0,000208	-0,00051
1	12	0,840172	0,02705	0,003804	0,004647	0,004308	0,02356	0,002863	0,021192	-0,00444
1	12	0,529304	0,120106	0,058808	0,019571	0,008259	0,017534	0,02858	0,024512	0,037713
1	12	0,715834	5,16E-05	0,162485	0,002546	0,001147	0,002999	0,001592	0,00055	-0,16471
1	12	0,79084	9,2E-07	0,016807	0,003731	0,079235	0,004428	0,002687	5,17E-05	-0,02514
1	12	0,907897	3,85E-05	0,043833	0,000415	0,009679	0,000296	6,14E-05	0,003318	-0,04414
1	12	0,579235	0,005905	0,063237	0,113282	0	0,009341	0,00027	0,015896	-0,13508
1	12	0,963138	0	0,00354	0,00727	0,007535	0,007284	0	3,21E-05	-0,01332
1	12	0,739974	0,000593	0,009483	0,029312	0,03759	0,01876	0,002785	6,14E-05	-0,05066
1	12	0,514286	0,100028	1,42E-05	0,089612	0,007673	4,68E-05	7,73E-05	0,002887	0,010363
1	12	0,531613	0,00607	0,04005	0,06171	0,000134	0,053926	0,001762	0,004448	-0,09616
1	12	0,619531	0,001269	0,01045	1,46E-07	0,088046	0,015394	0,000485	0,006573	-0,02087
1	12	0,429462	0,002105	0,277881	0,000158	0,030015	8,16E-05	0,007488	0,000134	-0,27578
1	12	0,560641	0,066211	0,039354	0,012395	0,017343	0,014257	0,124017	0,002001	-0,08238
1	12	0,769509	0	0,154877	0	0,008682	0	0	0,005025	-0,15488
1	12	0,944725	0	0,005312	0,00182	0,002621	0,000231	1,15E-07	0,00135	-0,00687
1	12	0,631347	5,5E-08	0,000889	0,037299	0,015367	0,009844	0	0,002736	-0,04049
1	12	0,681167	0,008101	0,001712	0,025213	0,044331	0,023605	0,000351	2,97E-05	-0,03572
1	12	0,445864	0,005969	0,055561	0,081701	0,000209	0,043289	0,061967	0,052979	-0,17003
1	12	0,481823	0	0,084477	0,001412	0,005561	0,007108	2,1E-06	0,005708	-0,08668
1	12	0,644729	0,012707	3,92E-05	0,08093	0,00908	0,004012	0,018536	7,01E-05	-0,07025
1	12	0,635875	0,02348	1,99E-07	0,012001	0,006408	0,049631	0,001901	0,000947	-0,02615
1	12	0,948644	0	0,003785	0,043635	0,002054	0,000549	0,001772	0,008331	-0,04623
1	12	0,615843	0	0,12933	0,006312	0,000208	0,008882	0,003932	5,21E-05	-0,13766
1	12	0,83214	3,3E-05	0,002212	0,016394	0,019096	0,021244	0,000135	0,002312	-0,02982
1	12	0,369708	0	0,0021	0,106817	5,61E-05	0,218309	0,017574	6,49E-05	-0,30056

1	12	0,423842	0,054573	0,021923	0,001511	0,025042	0,071201	0,019772	0,005244	-0,03829
1	12	0,461893	0,000844	0,339948	0,017973	0,010537	0,011177	0,013354	0,001532	-0,34791
1	12	0,441833	0,014374	0,057767	0	0	0,133274	2,87E-05	7,22E-05	-0,11918
1	12	0,635569	9,58E-06	1,07E-05	3,55E-05	0,001409	0,001075	0,022502	4,67E-05	-0,02326
1	12	0,885103	0,008169	6,91E-05	0,009654	0,017652	3,42E-05	0,000412	0,000172	-0,00187
1	12	0,740796	0,042008	0,005376	0,015135	0,007381	0	0,004012	0,001133	0,025304
1	12	0,917996	6,62E-05	0,025646	0,029037	0,006609	2,43E-05	0,001514	0,000511	-0,03659
1	12	0,483701	0	0,034016	0,047089	0	0	0,053901	0,000101	-0,10405
1	12	0,914297	0,000956	0,01044	0,000343	0,001832	0,002324	0,021681	0,000851	-0,02417
1	12	0,462662	0,003527	0,047657	7E-08	9,67E-07	0,111173	0,013581	1E-09	-0,10765
1	12	0,535513	0,03511	2,66E-06	0,000173	1,34E-05	3,18E-05	1,1E-06	0,002122	0,034937
1	12	0,71478	0,046655	0,002664	0,040737	5,28E-07	6,2E-08	0,042474	0,027864	-0,00959
1	12	0,247127	0,00632	0,552118	0,045385	0,000282	0,004011	0,01342	0,003624	-0,5458
1	12	0,388063	0,224392	0,018511	0,000129	0,001087	2E-09	2,31E-06	0,001338	0,205881
1	12	0,469072	2,39E-05	0,229452	0,001798	0,006432	6,5E-08	8,46E-06	0,000475	0,230007
1	12	0,701136	0,181469	0,000264	0,038218	9,67E-07	1E-09	0,028533	0,001139	-0,05683
1	13	0,653612	2,39E-05	0,010626	0,028338	0,003355	0,002686	0,093729	0,002665	-0,11513
1	13	0,953655	0	0,000147	4E-09	0,022352	0,000135	0,001864	0,000649	-0,00206
1	13	0,993542	0	0,010216	6,95E-05	0,000126	0	9,22E-05	0,000228	-0,01037
1	13	0,564193	0,049356	0,270278	0,007836	0,002679	0,000793	0,003325	0,000521	-0,2241
1	13	0,497795	0,164763	0,001468	0,001995	3E-09	0	0,000135	0,00059	0,161401
1	13	0,924931	0,003451	0,009295	0,00079	0,000695	0,002288	0,000177	0,000201	-0,00693
1	13	0,449271	0	0,318692	0,124819	0,000258	0,00092	0	0	-0,4244
1	13	0,415098	0,308402	0,033785	2,2E-08	0,000378	0,001275	0,003869	0,020971	0,273888
1	13	0,371292	0,001057	0,341599	0,005352	3,9E-05	0,00391	0,046939	0,001241	-0,34445
1	13	0,764899	0,00058	0,034763	0,002304	0,01637	0,000171	0,005981	0,058358	-0,03422
1	13	0,564147	0,149464	0,001911	1,5E-06	0,005963	0,02053	1,34E-05	0,041297	0,128372
1	13	0,856504	0,000167	0,027881	0,00111	0,001401	0,009089	0,000109	0,004077	-0,03508
1	13	0,855516	0	0,008453	0,005929	0,079779	9,2E-05	0,008981	0,000685	-0,02052
1	13	0,805592	0,000299	0,05408	0,021255	0,006608	0,000275	0,00123	0,002062	-0,06679
1	13	0,70859	0,004933	0,03132	0,014903	0	0,046252	7,99E-05	0,000489	-0,05629
1	13	0,959804	0	0,000987	0,012117	0,011148	0,005035	0	0,000154	-0,01494
1	13	0,874885	2,59E-07	0,029448	0,000537	0,013484	0,005875	3,67E-05	0,000118	-0,03491
1	13	0,643949	0,103124	0,000378	0,071151	0,020513	0,000101	0,000464	0,008617	0,031512
1	13	0,574544	0,004536	0,012829	0,045341	0,000489	0,046597	0,000247	0,000505	-0,06436
1	13	0,650179	0,002208	9,83E-05	0,003647	0,004353	0,021258	0	0,048774	-0,02264
1	13	0,469072	1,25E-06	0,229452	0,001798	0,006432	6,5E-08	8,46E-06	0,000475	-0,23001
1	13	0,121765	0,749782	0,017767	0,002027	0,011073	0,002533	0,015649	0,002087	0,719392
1	13	0,893068	0	0,009134	0,008599	0,003818	0,001435	0,003161	0,006033	-0,01882
1	13	0,92613	0	0,023113	0,001209	0,000976	0,001356	0,000247	0,000809	-0,02366
1	13	0,606494	0,007076	0,004829	0,109597	0,002652	0,008593	0,004739	4,7E-05	-0,1053
1	13	0,505572	0,105627	0,022494	0,043478	0,013802	0,05124	0,023771	0,002076	0,023345
1	13	0,575676	0,012653	0,046445	0,054348	0,007531	0,006136	0,052576	0,016088	-0,0967
1	13	0,40468	0,181469	0,064171	0,012725	0,006714	0,000677	0,0003	0,008008	0,111076
1	13	0,601756	0,037228	0,017783	0,044964	0,005013	0,005648	0,013086	0,007913	-0,03127
1	13	0,616085	0,000415	0,000624	0,065774	0,059486	0,051032	4,52E-06	0,007474	-0,08784
1	13	0,909144	0	0,005234	0,02024	0,00469	6,34E-05	0,001416	0,010604	-0,02239
1	13	0,701136	0	0,000264	0,038218	0	1E-09	0,028533	0,001139	-0,05683

1	13	0,960815	0	0,000677	2,27E-06	1,4E-05	9,76E-05	2,74E-07	0,000333	-0,00076
1	13	0,653507	0,04424	0	0,000233	0	0,034157	0,01484	0,000724	-0,0046
1	13	0,433987	0,097393	0,018616	0,022893	0,027874	0,022273	0,020965	0,003799	0,035775
1	13	0,427068	0,000873	0,431088	0,001627	2,94E-06	0,000578	0,006157	9,45E-05	-0,43022
1	13	0,346232	0,204932	0,023991	0,000936	0,010094	0,177548	0,000388	0,002059	0,003585
1	13	0,713821	0,068852	0,002204	0,001122	0,003459	0,002903	0,02985	0,002024	0,037397
1	13	0,954605	0,003351	0,006931	4,56E-07	0,000154	6E-09	4,3E-08	0,001368	-0,00358
1	13	0,734808	0,032111	0,01267	0,001515	0,022084	0,0012	0,052654	0,000981	-0,02173
1	13	0,962508	0,000143	0	0,014895	0,01321	5,1E-08	0,00025	0,003245	-0,01475
1	13	0,506331	0,074165	0,012718	0,066539	0,000119	0	0,088285	0,000899	-0,07298
1	13	0,80863	8,97E-05	0,011972	0,011206	6,03E-05	0,000537	0,109216	2,48E-05	-0,10934
1	13	0,462222	0,002726	0,024015	0,004573	3,55E-07	0,12083	0,005184	0	-0,1181
1	13	0,502077	0,061677	0,001067	4,11E-05	3,18E-06	1,3E-05	2,6E-07	0,000559	0,060579
1	13	0,558672	0,010721	0,015914	0,084371	9,3E-08	1,1E-08	0,114164	0,018307	-0,10512
1	13	0,325185	0,003122	0,430356	0,049074	0,001919	0,015814	0,00993	0,001658	-0,42723
1	13	0,410884	0,178886	0,009042	2,07E-05	0,001797	1E-09	3,23E-07	0,00173	0,169844
1	13	0,185781	0,631022	0,00076	0,000879	0,000122	0,022273	0,000456	0,001662	0,629865
1	13	0,554914	0,024954	0,032986	0,020991	7,59E-05	0,000752	0,06234	0,000559	-0,06305
2	1	0,535564	0,158003	0,051011	0,101385	0,00655	0,006132	0,016516	0,014151	0,043272
2	1	0,98239	0	0,001468	2,5E-08	0,000237	0	0,003161	0,000148	-0,00451
2	1	0,903426	0,000223	0	0,004405	0,022559	0,004529	0,001313	0,001991	-0,00468
2	1	0,988302	7,7E-08	0,014787	0	1,21E-06	1,7E-08	6,03E-07	3,05E-05	-0,01479
2	1	0,991388	7,15E-06	0,010803	4,09E-05	0,000102	4,68E-05	0	0,000151	-0,0108
2	1	0,752125	0,009989	0,142552	0,02383	0,000835	0,009123	0,003977	0,007853	-0,14841
2	1	0,494626	0,045058	0,089686	0,002504	0,000472	2E-05	0,103448	0,000303	-0,07984
2	1	0,733818	0,005267	0,057475	0,013165	0,018452	0,012431	0,002247	0,002408	-0,06565
2	1	0,596415	0,000664	0,107345	0,226234	0,002006	0,002561	0,020165	0,002464	-0,30505
2	1	0,53778	0,117749	0,079752	0,002868	0,001156	0,006918	0,014748	0,006258	0,032446
2	1	0,447918	0,299727	0,000698	0,001097	0,009816	0,002312	0,000504	0,003598	0,296116
2	1	0,870514	0,004856	0,051783	0,006302	0,002436	0,00379	0,007965	0,003218	-0,04942
2	1	0,770834	0,007663	0,001109	0	0,01461	0,021071	7E-09	0,013583	-0,01408
2	1	0,88641	3,12E-05	0,016341	0,004657	0,003874	0,021271	0,000762	0,011879	-0,03798
2	1	0,834826	1,72E-05	0,021473	0,006846	0,075084	0,003298	0,000832	0,008219	-0,02572
2	1	0,664406	0,000625	0,048149	0,154982	0,004955	0,003178	0,000743	0,000556	-0,19511
2	1	0,583564	0,012494	0,013416	0,030135	0,001162	0,031888	0,000196	0,008046	-0,04412
2	1	0,906727	0,000813	8,21E-05	0,035148	0,002687	0,006251	0,000481	0,000394	-0,03835
2	1	0,797721	0,00306	0,020927	0,012357	0,01317	0,003766	0,004031	0,000582	-0,02571
2	1	0,492731	0,041071	0,158492	0,013448	0,006087	0,0156	0,04444	0,009302	-0,12581
2	1	0,548491	0,03592	0,023658	0,0094	0,008169	0,007726	0,003446	0,020759	-0,00128
2	1	0,443381	0,113074	0,000847	0,010443	0,006878	0,010909	0,000567	0,006301	0,096853
2	1	0,541332	0,018575	0,177117	0,04599	0,010245	0,009841	0,006671	0,03003	-0,19911
2	1	0,67347	0,012773	0,013121	3,26E-05	0,001852	0,008657	0,09452	0,002125	-0,08279
2	1	0,8913	0	0,027088	0,010165	0,001931	0,000188	0,000379	0,003198	-0,03512
2	1	0,803238	0,114579	0,017076	0,005842	0,003625	0,002415	0,005416	0,003639	0,087298
2	1	0,598677	1,77E-05	0,002734	0,003621	0,04554	0,0186	0,029568	0,007262	-0,04108
2	1	0,554914	0,024954	0,032986	0,020991	7,59E-05	0,000752	0,06234	0,013247	-0,06305
2	1	0,447146	0,020831	0,094176	0,008162	0,002506	0,021947	0,135641	0,010604	-0,19184
2	1	0,444855	0,176059	0,005371	0,002023	0,001099	0,00447	0,003837	0,002049	0,164094

2	1	0,439868	0,058987	0,000252	0,161624	0,00832	0,001826	0,007878	0,000126	-0,10264
2	1	0,586943	0,131404	0,01169	0,026531	0,009979	0,027685	0,011166	0,003918	0,074828
2	1	0,813587	0,000245	0,004375	0,207218	0,002325	0,00056	0,002078	0,002712	-0,20948
2	1	0,43406	0,000979	0,37218	0,00068	4,83E-05	0,00223	0,001644	0,004681	-0,37295
2	1	0,868077	0,002412	0,07765	0,011071	0,005974	0,004712	0,023289	0,00129	-0,08224
2	1	0,499467	0,006124	0,026679	0,02087	0,00806	0,079235	0,007112	0,001136	-0,09401
2	1	0,477378	0,050927	7,44E-05	0,000284	0,03596	0,027814	0,010028	0,001292	0,013829
2	1	0,461084	0,00575	0,305327	0,009528	0,000329	0,0019	0,004663	0,012041	-0,3002
2	1	0,429543	0,016116	0,108075	0,008989	0,00406	0,092285	0,002602	0,000812	-0,17932
2	1	0,546182	0,011322	0,036348	0,008712	0,007903	0,015357	0,034343	0,005189	-0,04768
2	1	0,816105	0,03524	0,018921	0,011343	0,000726	0,000651	0,027834	0,00089	-0,01171
2	1	0,623298	0,028771	0,010902	0,024254	0,045322	0,005987	0,018931	0,002042	-0,01584
2	1	0,924053	1,3E-06	0,018038	0,004782	0,000343	0,001163	0,012793	0,000404	-0,02989
2	1	0,527144	0,005947	2,06E-05	0,102389	0,030143	0,003346	0,014736	0,000315	-0,10866
2	1	0,850086	0,003203	0,013567	0,009854	0,017165	0,006356	0,090481	0,001003	-0,09302
2	1	0,721535	0,000297	0,089526	0,00407	0,000699	0,009731	0,006122	0,004097	-0,09232
2	1	0,495892	0,116738	0,001269	9,05E-05	0	6,5E-08	0,000197	0,006021	0,115454
2	1	0,801661	0,00803	0,00435	0,009627	0,022536	0,015171	0,007561	0,007676	-0,01712
2	1	0,185781	0,631022	0,00076	0,000879	0,000122	8,31E-05	0,000456	0,001662	0,629865
2	1	0,90474	0,000102	0,00177	1,33E-06	5,95E-05	1,7E-05	1,88E-06	0,00041	-0,00167
2	2	0,193631	0,736684	3,25E-05	0,008116	0,015243	0,000326	1,08E-05	0,004193	0,728568
2	2	0,658305	0,237785	0,000107	1,41E-05	0	0,000556	0,004606	1,28E-05	0,233174
2	2	0,825505	0,00028	0,004884	0,023848	0,015211	0,012967	0,001526	0,003172	-0,02448
2	2	0,979166	2,43E-05	0,027141	0	7,44E-05	1,2E-08	7,31E-06	0,000106	-0,02712
2	2	0,978836	0	0,036915	2,27E-05	0,00011	9,47E-06	7,65E-05	0,00082	-0,037
2	2	0,722095	0,010248	0,125601	0,015206	0,005002	0,004831	0,002963	0,002169	-0,13082
2	2	0,407663	0,218143	0,043176	0,000779	0,00015	0,000322	0,01143	0,001269	0,166999
2	2	0,907344	0	0,001973	0,013339	0,025885	0,00165	0,000924	3,19E-05	-0,01423
2	2	0,315945	0	0,46109	0,137826	2,74E-05	0,000712	0,003305	0,003681	-0,5533
2	2	0,715187	2,3E-06	0,147954	0,007069	5,39E-06	0,009703	0,016792	0,00084	-0,1555
2	2	0,348214	0,282196	0,051808	0,000169	1,84E-05	0,014393	0,022718	0,002838	0,216731
2	2	0,933036	0,000378	0,035406	0,003689	0,001444	0,006681	0,002618	0,00332	-0,03784
2	2	0,781736	0,006461	0,041673	0,004148	0,005039	0,039156	0,004649	0,021681	-0,06612
2	2	0,958234	0	0,000189	3,58E-06	0,019589	0,01035	2,34E-05	0,011312	-0,01035
2	2	0,817258	0,00212	0,052469	0,00471	0,045667	0,015035	0,005621	0,003344	-0,06332
2	2	0,761763	0,000403	0,041774	0,080644	0,005812	0,002996	0,000915	0,003227	-0,11703
2	2	0,529895	0,028052	0,058003	0,054635	0,00074	0,010513	0,000554	0,010197	-0,07548
2	2	0,937313	0,000143	0,001764	0,010621	0,013066	0,011304	0,000724	0,00019	-0,01861
2	2	0,767404	0,001596	0,014715	0,025455	0,007058	0,007635	0,001515	0,000874	-0,03781
2	2	0,727964	0	0,003682	0,081179	0,003299	0,001065	0,001184	0,014298	-0,08439
2	2	0,553745	0,072369	0,005655	0,027328	0,000891	0,0308	0,001471	0,007251	0,027126
2	2	0,648371	0,066365	0,006794	0,001685	0,002035	1,13E-05	0,000146	0,003152	0,058445
2	2	0,574045	1,1E-05	0,129366	0,032064	0,001035	0,006638	0,005427	0,001038	-0,14456
2	2	0,718016	0,025747	0,030993	0,002709	0,002403	0,012597	0,024752	0,001112	-0,02613
2	2	0,823487	0	0,114172	0,000587	0,001984	0	3,21E-05	0,03245	-0,11417
2	2	0,809024	0,110593	0,001931	0,001945	0,008754	0,009224	0,003379	0,003009	0,097693
2	2	0,500362	0,097668	0,0005	0,008641	0,040936	0,008102	0,001408	0,00332	0,083986
2	2	0,646238	0,097137	0,012051	0,073427	7,92E-05	0,000155	0,016075	0,001839	0,014821

2	2	0,506	0,004861	4,64E-07	0,0352	0	0,006989	0,22066	0,020632	-0,223
2	2	0,493745	0,052382	0,069564	0,019765	0,005153	0,005503	0,006635	0,004645	-0,03413
2	2	0,670611	0,001835	0,001322	0,190312	0,001789	0,00013	0,013456	0,001395	-0,1936
2	2	0,594401	0,075232	0,001695	0,045432	0,01876	0,031529	0,011634	0,005645	0,00994
2	2	0,884716	3,48E-05	0,00422	0,026822	0,004214	0,000329	0,004733	0,003744	-0,03234
2	2	0,279855	0,001302	0,603168	0,001127	0,000403	5,59E-05	0,001926	3,69E-06	-0,60203
2	2	0,78339	0,017376	0,081972	0,015299	0,021158	0,001707	0,018205	0,001197	-0,07949
2	2	0,605934	0,000385	0,016176	0,000917	0,002131	0,106667	0,003333	0,000522	-0,10841
2	2	0,378941	0,064394	0,134962	0,041648	0,021866	0,058578	0,074775	0,035416	-0,12631
2	2	0,575647	0,029751	0,11784	0,003194	0,000192	0,013742	0,01428	0,007702	-0,09727
2	2	0,406473	0,051529	0,025928	0,002117	0,001973	0,166552	0,001186	0,00343	-0,14008
2	2	0,689005	0,000224	0,030868	0,00407	0,002837	0,006427	0,001688	0,000954	-0,03578
2	2	0,924654	0,003477	0,011653	0,008473	0,00366	0,000171	0,00443	0,001018	-0,01923
2	2	0,541841	0,102548	0,05323	0,000564	0,024169	0,000247	0,020268	0,003937	0,036214
2	2	0,869129	0	0,036182	0,001999	0,000875	0,013332	0,080334	5,4E-05	-0,09094
2	2	0,674808	0,04443	0,002781	0,027331	0,023081	0,001936	0,082998	0,000516	-0,06335
2	2	0,83823	0,000459	0,019036	0,003922	0,001639	0,010215	0,018945	0,003921	-0,03685
2	2	0,756985	0,07617	0,18512	0,002747	4,39E-06	0,00016	0,003043	0,064322	-0,10895
2	2	0,394729	0,239622	0,0002	0,001043	0	1E-09	0,000153	0,001363	0,238579
2	2	0,714044	0,005388	0,003326	0,016654	0,03766	0,019687	0,000833	0,004288	-0,02305
2	2	0,461145	0,092404	0,002047	0,003861	0,009509	2,52E-06	0,002497	0,025347	0,087734
2	2	0,890451	0,001805	0,005443	7,28E-05	0,00099	3,39E-05	1,79E-05	0,002519	-0,00364
2	3	0,397069	0,489779	9,45E-06	0,064933	0,006065	9,49E-05	3,14E-06	0,001933	0,424846
2	3	0,950949	0,011806	0,000528	0	0,000496	0,000311	0,008176	0	0,003272
2	3	0,814725	1,81E-05	0,174051	0,003291	0,000976	0,005969	9,83E-05	0,001009	-0,17403
2	3	0,99347	0	0,004856	2,28E-07	1,26E-07	5,5E-07	2,7E-06	6,3E-05	-0,00486
2	3	0,988202	0	0,015125	3,28E-06	0,000817	1,09E-05	0,000302	0,00173	-0,01519
2	3	0,780206	0,00102	0,075854	0,005931	0,005759	0,001933	0,003013	0,00149	-0,07981
2	3	0,495785	0,042444	0,014778	0,00158	3,1E-05	0,000579	0,002311	0,000388	0,024259
2	3	0,919983	0	0,008227	0,00069	0,033144	2,5E-05	0,000697	0,000448	-0,00844
2	3	0,459874	0	0,380313	0,091195	5,77E-06	0,000126	1,09E-05	0,000766	-0,4675
2	3	0,674357	0	0,156008	0,000881	2,18E-05	0,00414	0,008746	2,34E-05	-0,15844
2	3	0,560603	0	0,025162	0,00093	0,001688	0,010395	0,002209	0,007121	-0,03532
2	3	0,921443	0,001776	0,009732	0,000661	0,004829	0,00386	0,001211	0,00084	-0,01065
2	3	0,770271	0,001599	0,028601	0,000144	0,000997	0,008161	0,000322	0,008689	-0,03278
2	3	0,773538	0,00142	0,026393	0,002481	0,00563	0,005131	0,000771	0,023806	-0,02884
2	3	0,868542	6,17E-05	0,010799	0,001681	0,018083	0,003223	4,51E-05	0,011079	-0,01341
2	3	0,778369	8,24E-06	0,088233	0,011838	0,017715	0,00527	0,000629	0,001538	-0,09718
2	3	0,554592	6,15E-05	0,010722	0,135724	0,002387	0,006969	7,27E-05	0,005562	-0,14625
2	3	0,957497	4,43E-05	0,002749	6,78E-05	0,003315	0,00259	0,000425	0,000604	-0,00561
2	3	0,739843	0,00052	0,046143	0,021651	0,026308	0,017574	0,000367	0,001305	-0,07378
2	3	0,780682	0,001266	0,012212	0,025484	0,016097	0,000645	0,003599	0,006132	-0,03279
2	3	0,416765	0,279919	0,010933	0,017254	0,000533	0,002858	0,007792	0,038143	0,250216
2	3	0,76298	0	0,005735	0,000589	0,003816	0,000436	0,001778	0,008774	-0,00747
2	3	0,466255	0,000423	0,31575	0,026699	0,000261	0,022515	0,006117	0,001029	-0,31533
2	3	0,472279	0,159939	0,090778	0,010021	0,020248	0,007296	0,015445	0,032373	0,062604
2	3	0,883212	0,000303	0,062954	0,000863	1,53E-05	0,000133	0,000461	0,002817	-0,06285
2	3	0,917655	8,2E-08	0,010245	0,000788	0,004467	0,004765	0,00015	0,004477	-0,01411

2	3	0,507589	0,038314	0,000416	0,01427	0,079231	0,007306	0,022684	0,002297	0,004506
2	3	0,758663	0,018935	0,022681	0,003043	0,004235	0,007516	0,003382	0,004246	-0,01089
2	3	0,41235	1,11E-05	0,159509	0,037769	2,77E-06	0,008427	0,13532	0,008596	-0,21162
2	3	0,499775	0,104373	0,077397	0,034992	0,009291	0,025375	0,004351	0,001728	-0,02914
2	3	0,686295	0,000676	0,000558	0,02815	0,021156	0,009832	0,00431	4,62E-05	-0,03403
2	3	0,504816	0,066024	0,01913	0,001471	0,004142	0,000653	0,042472	0,009875	0,021734
2	3	0,870215	0,002199	0,012661	0,076542	0,00731	0,001437	0,001424	0,004471	-0,08064
2	3	0,195317	0	0,754575	0,004592	3,05E-05	0,000726	0,003128	1,73E-05	-0,75913
2	3	0,790532	0,015912	0,125365	0,010331	0,000943	0,005042	0,018669	0,000145	-0,11872
2	3	0,56931	0	0,024534	1,78E-05	0,038694	0,048564	0,008042	0	-0,06787
2	3	0,478786	0,006492	0,018516	0,011758	0,007579	0,002142	0,025756	0,017467	-0,04067
2	3	0,702624	0,00871	0,07599	0,004722	3,8E-05	0,000918	0,000422	0,001406	-0,06918
2	3	0,347021	0,001164	0,175818	0,004181	0,000312	0,161956	0,000716	0,017172	-0,31882
2	3	0,660194	0	0,036458	0,002988	0,005389	0,010443	0,030009	0,001043	-0,04865
2	3	0,768683	0,03571	0,008532	0,043107	0,002082	0,001551	0,040009	0,001095	-0,03644
2	3	0,679426	0,018894	0,005336	0,002851	0,010164	0,00472	0,000561	0,004055	0,007028
2	3	0,828523	2,81E-05	0,003216	0,023988	0,003341	0,015847	0,024805	8,29E-06	-0,05177
2	3	0,718724	0,013665	0,000762	0,022847	0,014477	0,003927	0,030606	0,00093	-0,03385
2	3	0,887545	0,005747	0,015385	2,95E-06	0,00282	0,003651	0,030788	0,001452	-0,0315
2	3	0,907229	0,083957	0,020094	0,000917	6,3E-08	2,3E-06	7,58E-05	0,011624	0,063863
2	3	0,474702	0,018615	0,01658	0,004932	0	0,000766	0,065293	0,007172	-0,04668
2	3	0,780647	0,003551	0,00053	0,001913	0,048165	0,015424	0,009443	0,004685	-0,01623
2	3	0,601436	0,002651	0,009155	0,006325	0,002747	7,47E-05	0,003363	0,010625	-0,01004
2	3	0,836426	0,000217	0,070261	5,34E-06	0,000109	2,31E-05	6,68E-05	0,000228	-0,07004
2	4	0,488439	0,349594	2,92E-06	0,040188	0,013118	0,012229	9,68E-07	0,00228	0,297235
2	4	0,984688	0	0,000761	0,003037	0,001254	0	0,002306	0,000483	-0,00451
2	4	0,964775	1,07E-06	0,039174	0,000195	5,77E-05	0,000392	5,81E-06	0,000634	-0,03917
2	4	0,933578	0	0,119601	0	0	0	2E-09	0,000178	-0,1196
2	4	0,989103	0	0	2,81E-07	3,25E-06	6,89E-07	0	4,88E-05	-6,9E-07
2	4	0,7335	0,001194	0,136888	0,011431	0,00167	0,000727	0,003959	0,001353	-0,14302
2	4	0,481813	0	0,02162	0,024293	0,000241	0,001339	0,048688	0,00037	-0,06987
2	4	0,920548	0	0,001271	0,012027	0,044704	0,000148	0,00059	0,000845	-0,01299
2	4	0,466481	5,07E-06	0,283174	0,151611	0,000569	0,002238	0,003887	0,001534	-0,41781
2	4	0,705849	0,000807	0,118414	0,004341	0,000814	0,030195	0,003521	0,001198	-0,13303
2	4	0,587557	0	0,007355	0,000564	0,001859	0,012937	0,001327	0,003331	-0,0204
2	4	0,907302	0,001172	0,046665	0,001183	0,001642	0,008503	0,003029	0,000363	-0,05443
2	4	0,701488	0,000432	0,023916	0,00031	0,000462	0,035817	0,000712	0,019873	-0,05116
2	4	0,717481	0,002931	0,168439	0,002067	0,009128	0,008515	0,001976	0,009403	-0,17009
2	4	0,80162	0,000858	0,026121	0,021754	0,050432	0,007581	0,000176	0,003502	-0,04484
2	4	0,817382	0,000467	0,034089	0,017738	0,00775	0,015004	0,00077	0,006985	-0,05623
2	4	0,731815	0,000148	0,015253	0,039836	0,000869	0,012392	0,000112	0	-0,05993
2	4	0,961755	0	0,000198	0,004295	0,007498	0,019124	0,000135	0,000649	-0,02271
2	4	0,771912	0,000688	0,039771	0,013056	0,017421	0,009218	0,001776	0,001308	-0,0538
2	4	0,829641	0,000135	0,019288	0,015191	0,014038	0,001143	0,008526	0,002875	-0,0328
2	4	0,576865	0,042257	0,030006	0,015887	0,020298	0,004674	0,001708	0,014055	0,000946
2	4	0,786045	0	0,004491	0,002592	0,00845	6,14E-05	0,00072	0,011422	-0,00589
2	4	0,480176	0	0,314434	8,72E-05	0,035727	0,000534	0,012402	4,14E-06	-0,31443
2	4	0,45696	0,082427	0,143925	0,009993	0,002163	0,005675	0,002788	0,084306	-0,06747

2	4	0,869386	0,003561	0,05202	0,00125	0,007562	0,001541	0,000398	0,027006	-0,04997
2	4	0,91479	0	0,018864	0,001085	2,39E-05	0,000489	0,002999	0,000809	-0,02025
2	4	0,624681	0,011034	0,00199	0,003077	0,037632	0,006997	0,023522	0,010824	-0,01645
2	4	0,496622	0,047684	0,018755	0,07296	0,00018	0,003069	0,011273	0,001615	-0,03435
2	4	0,398853	0	0,150793	0,001381	0,001419	0,077668	0,114882	0,001595	-0,23372
2	4	0,413778	0,014179	0,15499	0,000838	0,036657	0,012551	2,48E-05	0,001088	-0,14522
2	4	0,621928	0,002461	0,006864	0,116303	0,005631	0,000538	0,005601	0,003735	-0,11874
2	4	0,554601	0,016766	0,002901	0,079093	0,004801	0,069494	0,041694	0,002113	-0,11544
2	4	0,91634	0,007531	0,008184	0,025732	0,002522	0,002588	0,008495	0,001689	-0,02988
2	4	0,464039	0,073207	0,058905	0,000444	0,002261	0,045531	0,007485	0,000405	-0,02061
2	4	0,88259	0,002842	0,025783	0,010379	0,008865	0,01136	0,016455	0,001274	-0,03506
2	4	0,433063	3,97E-05	0,135096	0,004315	0,005483	0,120756	0,000552	0,007178	-0,21736
2	4	0,482433	4,15E-06	0,033162	0,02627	0,038705	0,042513	0,014545	0,00424	-0,08849
2	4	0,618222	0,00169	0,104018	0,042638	3,32E-05	0,013514	0,000666	0,007274	-0,1384
2	4	0,379149	0,011382	0,113324	8,74E-05	8,38E-07	0,14566	3,23E-05	0,013728	-0,23931
2	4	0,649238	0,002614	0,034821	0,012863	0,010952	0,004062	0,005514	0,005584	-0,04302
2	4	0,777812	0,020777	0,043156	0,006212	0,001077	0,000271	0,002539	0,000459	-0,02798
2	4	0,522406	0,153759	0,002677	0,007481	0,022657	0,007859	0,020546	0,011284	0,127432
2	4	0,841146	1E-08	0,01962	0,007086	0,005194	0,003667	1,32E-07	0,000869	-0,02549
2	4	0,820511	0,034478	0,000176	0,029333	0,000129	0,010527	0,011268	0,000641	-0,00976
2	4	0,910277	0,006188	0,009766	0,00106	0,00163	0,000329	0,004874	0,002935	-0,00546
2	4	0,927615	0,066779	0,0045	0,000205	1,4E-08	5,15E-07	1,7E-05	0,002603	0,062279
2	4	0,464725	0,005673	0,002606	0,01033	0,001171	0,000662	0,091573	0,002495	-0,0859
2	4	0,943413	0,005474	7,19E-05	0,000259	0,007393	0,002172	0,013427	0,013581	-0,00795
2	4	0,646417	0,000319	0,115242	0,020239	0,000358	0,001382	0,005816	0,011103	-0,11492
2	4	0,832168	4,14E-05	0,063385	1,02E-06	0,00014	0,000103	7,96E-05	4,36E-05	-0,06334
2	5	0,188046	0,68771	6E-09	8,24E-05	0,047687	0,045265	2E-09	7,47E-06	0,642446
2	5	0,98353	0	0,000286	0,00276	0,002245	0,000309	0,007419	0,000332	-0,00783
2	5	0,978263	2,5E-08	0,040013	4,56E-06	1,35E-06	9,17E-06	1,36E-07	1,82E-05	-0,04001
2	5	0,987843	0	0,01636	2E-05	2,24E-05	0	2,36E-06	0,000345	-0,01636
2	5	0,978268	0	0,027513	2,31E-05	0,001555	0	0,000185	0,0002	-0,02751
2	5	0,780092	0,001882	0,0718	0,011929	0,000231	0,004401	0,007744	0,006494	-0,08393
2	5	0,500514	0,047366	0,019341	0,000682	0,000106	8,82E-05	0,031436	0,000631	0,001658
2	5	0,867011	0	0,022223	0,038722	0,021703	0,002074	0,000944	0,001393	-0,04768
2	5	0,522569	2,25E-05	0,292401	0,084657	0,000606	0,002685	0,000265	0,000539	-0,35213
2	5	0,636791	0,034933	0,13659	0,001214	0,002021	0,037267	0,002313	0,004942	-0,1081
2	5	0,51592	0,004398	0,017095	0,003747	0,010705	0,013272	0,003451	0,001173	-0,02007
2	5	0,87904	0,005306	0,014556	0,016832	0,000729	0,002004	0,003276	0,002242	-0,02269
2	5	0,763503	0,000763	0,052242	0,00176	0,000307	0,035872	0,002299	0,014897	-0,07023
2	5	0,870519	0	0,030016	0,004699	0,001291	0,004978	0,000117	0,003866	-0,0349
2	5	0,664096	0,01585	0,168456	0,017732	0,078906	0,002856	0,0032	0,003052	-0,16887
2	5	0,844002	0,002039	0,027967	0,02738	0,009853	0,012275	0,002168	0,002001	-0,05231
2	5	0,639036	0,000803	0,007645	0,058289	0,00072	0,001699	0,000212	0,006526	-0,06286
2	5	0,966016	1,38E-05	0,000122	0,002371	0,025616	0,007209	0,00031	0,000354	-0,00931
2	5	0,80969	0,00048	0,002602	0,011996	0,016749	0,006019	0,000695	0,000962	-0,01799
2	5	0,68065	0,100576	0,003433	0,017069	0,005707	0,000989	0,00069	0,008804	0,081228
2	5	0,600126	0,009206	0,031208	0,014655	0,000551	0,026864	0,002088	0,003948	-0,04442
2	5	0,688653	0	0,021642	0,000371	0,011989	0,000788	0,000754	0,001352	-0,02232

2	5	0,603149	0,023536	0,129814	0,000675	0,012904	0,000461	0,00084	0,012233	-0,10694
2	5	0,752058	0,007582	0,030956	0,003833	0,00594	0,02172	0,039942	2E-09	-0,06471
2	5	0,924669	5,5E-05	0,026944	0,002401	0,002067	0,001016	3,55E-06	0,005227	-0,02826
2	5	0,901493	0	0,033373	0,000597	0,002041	0,000653	0,001408	0,000888	-0,03385
2	5	0,544593	0,082183	0,001895	0,01451	0,037549	0,006167	0,012592	0,001024	0,056754
2	5	0,415529	0,004249	0,020945	0,223044	0,000133	3,34E-06	0,072635	0,00536	-0,24054
2	5	0,486022	0,008213	0,135887	0,045126	0,004547	0,007967	0,076663	0,00088	-0,17124
2	5	0,541415	0,00171	0,038775	0,006184	0,019533	0,00161	1,53E-05	0,004564	-0,04281
2	5	0,488485	0,012413	0,000646	0,252116	0,008562	0,004402	0,053718	3,49E-05	-0,2499
2	5	0,52929	0,002191	0,006205	0,026271	0,014071	0,042402	0,020192	0,004642	-0,07767
2	5	0,871972	7,12E-06	0,010241	0,078738	0,01011	0,001057	0,002756	0,005648	-0,08609
2	5	0,616316	0	0,00174	1,82E-06	3,7E-08	9,67E-05	9,89E-07	2,26E-06	-0,00182
2	5	0,800889	0,011933	0,0512	0,027132	0,011372	0,007076	0,019563	0,007516	-0,06304
2	5	0,501756	5,94E-05	0,114136	0,004481	0,023053	0,06665	0,000335	0,001267	-0,12587
2	5	0,471469	0,003289	0,021622	0,03542	0,038273	0,022089	0,025241	0,033033	-0,07415
2	5	0,484772	0,008401	0,148022	0,012435	2,65E-05	0,028193	0,009557	0,004344	-0,15876
2	5	0,464095	0,00915	0,064424	0,000661	0,002068	0,015471	0,002138	0,000749	-0,069
2	5	0,809788	0,000728	0,032756	0,00144	0,006372	0,004303	0,002587	0,002554	-0,03566
2	5	0,916353	0,009224	0,013949	0,017235	0,001468	0,000411	0	0,001505	-0,0201
2	5	0,600423	0,113764	0,018517	0,000589	0,013068	0,003017	0,004251	0,009011	0,09263
2	5	0,916715	0,000404	0,028385	0,000488	0,020324	0,000579	0	4,11E-06	-0,02834
2	5	0,779622	0,008697	1,27E-05	0,064362	0,003083	0,005562	0,028912	0,001216	-0,08163
2	5	0,88215	0,000563	0,058912	0,001349	0,022383	0,000208	0,0154	0,000452	-0,06024
2	5	0,957713	0,030389	0,000892	4,07E-05	3E-09	1,02E-07	3,37E-06	0,000516	0,029497
2	5	0,495321	0,01315	0,00015	0,001479	0,000112	3,82E-05	0,010343	0,000178	0,002793
2	5	0,972028	0,002532	1,31E-05	4,74E-05	0,00135	0,000397	0,027948	0,005686	-0,02542
2	5	0,523389	6,39E-05	0,206375	0,013007	7,18E-05	0,001673	0,018891	0,003613	-0,20631
2	5	0,789207	1,25E-05	0,105831	0,000244	6,77E-05	0,000144	0,000184	1,31E-05	-0,10582
2	6	0,215055	0,631786	5E-09	6,26E-05	0,065373	0,042382	2E-09	2,3E-05	0,589403
2	6	0,976054	0	0,001346	5,64E-06	0,008699	0	0,012447	9,17E-05	-0,01251
2	6	0,903887	6E-09	0,189418	1,08E-06	3,19E-07	2,17E-06	3,2E-08	5,87E-05	-0,18942
2	6	0,998363	0	0,001553	3,36E-05	1,4E-05	0	0	0,000466	-0,00158
2	6	0,994944	4,51E-06	0,001797	1,68E-07	0,005345	0,000469	0,000849	0,000208	-0,00279
2	6	0,833448	6,74E-05	0,030655	0,024854	0,00151	0,006576	0,003599	0,003706	-0,05024
2	6	0,514863	0,063895	0,001041	0,002711	1,67E-07	0,000169	0,021577	0,000516	0,038766
2	6	0,884089	0,000292	0,037526	0,012013	0,009508	0,007182	0,002324	0,000884	-0,04793
2	6	0,449694	0,000176	0,390353	0,017975	0,001471	0,00345	0,009469	0,001297	-0,39765
2	6	0,601822	0,172297	0,008483	2,47E-06	3,01E-06	0,002165	7,53E-07	0,004337	0,162067
2	6	0,532288	0,008015	0,021372	0,001083	0,001556	0,007195	0,00361	0,001424	-0,02187
2	6	0,90564	0,002802	0,032077	0,005496	0,007247	0,005141	0,001524	0,000987	-0,03552
2	6	0,599037	0,03644	0,022943	0,017388	0,002353	0,020766	0,010331	0,034999	-0,01315
2	6	0,686555	0	0,197143	0,005002	0,004497	0,001889	0,003074	0,00065	-0,20017
2	6	0,730317	0,009321	0,084932	0,025896	0,055985	0,011685	0,000622	0,001371	-0,10363
2	6	0,84763	0,000135	0,036562	0,022637	0,012916	0,000829	0,000378	0,002906	-0,05691
2	6	0,526266	0,000304	0,073417	0,099841	0,001087	0,001835	0,000535	0,002132	-0,12701
2	6	0,931962	0,00017	0,000738	0,016276	0,031404	0,00222	0,000417	0,000375	-0,0184
2	6	0,828486	0,000413	0,00594	0,012681	0,011037	0,002694	0,001611	0,000605	-0,01709
2	6	0,439515	0,320158	0,012579	6,12E-05	0,003508	0,002305	0,003307	0,007051	0,307473

2	6	0,572851	0,019874	0,020579	0,014274	0,004887	0,015809	0,00088	0,019003	-0,0128
2	6	0,775568	0	0,007386	0,000987	0,046096	0,021029	0,001324	0,003372	-0,02582
2	6	0,527861	2,39E-07	0,094973	0,010804	0,012648	0,001188	1E-09	0,003639	-0,10077
2	6	0,841274	0,016335	0,028321	0,00117	1,8E-05	0,023096	0,018372	0,003058	-0,03716
2	6	0,896678	0,000658	0,028327	0,002992	0,005887	0,0003	0,000467	0,009613	-0,02862
2	6	0,936702	0	0,021145	0,005115	0,000955	0,00124	0,000198	0,000679	-0,02409
2	6	0,497131	0,055637	0,000265	0,049812	0,039447	0,00044	0,018844	0,001699	0,004719
2	6	0,532502	0,057773	0,018675	0,109291	0,002218	0,007464	0,023412	0,002937	-0,07685
2	6	0,376951	0,000232	0,235735	0,011281	0,000178	0,015644	0,047282	0,001807	-0,26894
2	6	0,471154	0,000417	0,12291	0,006895	0,01968	0,012617	9,58E-05	0,001281	-0,13794
2	6	0,54352	0,013882	0,002578	0,036566	0,006748	0,005593	0,046135	0,00083	-0,06035
2	6	0,583888	0,034156	0,005187	0,045929	0,008979	0,003636	0,051276	0,007731	-0,0624
2	6	0,885343	0,00016	5,37E-07	0,122383	0,015072	0,001308	0,006038	0,00191	-0,12293
2	6	0,260887	0	0,546333	2,78E-05	5,67E-06	0,000415	0,009745	0,000602	-0,54633
2	6	0,878498	0,007067	0,020605	0,014983	0,005336	0,002484	0,023382	0,00091	-0,03535
2	6	0,510983	0	0,139597	0,086288	2,63E-05	0,00775	0,005218	0,001446	-0,19209
2	6	0,511286	0,000182	0,055217	5E-09	0,037755	6,5E-06	3,19E-05	0,029913	-0,05507
2	6	0,632904	0,006219	0,020828	0,040281	0,002724	0,001976	0,016856	0,007194	-0,05095
2	6	0,543822	0,013638	0,026134	0,00016	0,018579	0,013359	0,004292	0,000892	-0,02354
2	6	0,619682	0,004418	0,053565	0,001733	0,008113	0,001559	0,000739	0,010709	-0,04933
2	6	0,843538	0,005467	0,02281	0,008282	0,00213	0,000288	0,007596	0,001969	-0,03204
2	6	0,464293	0,197829	0,06868	0,029688	0,04339	0,00314	0,006038	0,006483	0,119073
2	6	0,950487	0	0,013465	0,007052	0,000485	0	0	0,000462	-0,02014
2	6	0,863911	0,01015	0,001076	0,071536	0,005051	0,009759	0,02978	0,000749	-0,08497
2	6	0,922899	0,000209	0,00593	0,000683	0,000722	0,002615	0,002795	0,004788	-0,00806
2	6	0,968754	0,01282	0,000358	1,63E-05	1E-09	4,1E-08	1,35E-06	0,000207	0,012463
2	6	0,447703	0,119299	0,000889	0,008043	0,000128	0,002258	0,022306	0,011139	0,096788
2	6	0,967952	0,000486	2,04E-06	7,36E-06	0,00021	6,16E-05	0,057469	0,029873	-0,05698
2	6	0,443202	1,53E-05	0,256727	0,003292	1,72E-05	0,00094	0,053332	0,000868	-0,25671
2	6	0,791805	2,58E-06	0,103889	0,000224	1,99E-05	0,000139	0,000166	2,72E-06	-0,10389
2	7	0,348784	0,370584	2E-09	0,000198	0,112307	0,029678	1E-09	3,16E-05	0,340906
2	7	0,975011	0	0,000107	2,22E-05	0,001816	0	0,013641	0,000306	-0,01366
2	7	0,872339	2E-09	0,251772	3,83E-07	1,14E-07	7,72E-07	1,1E-08	7,06E-05	-0,25177
2	7	0,993647	0	0,00775	0,001676	0,000525	0	0	0,000905	-0,00937
2	7	0,990513	0	0,009768	1,94E-05	1,81E-05	0,000133	0,000212	0,004132	-0,00996
2	7	0,809604	0,000314	0,05455	0,022759	0,001099	0,001114	0,002346	0,005458	-0,06673
2	7	0,459589	0,015096	0,012308	0,008654	0,000178	2,14E-05	0,095479	0,0002	-0,08957
2	7	0,910221	0,000118	0,01237	0,007971	0,001705	0,000862	0,000245	0,000574	-0,01604
2	7	0,600342	0	0,139229	0,086822	5,12E-05	2,5E-08	0	0,000124	-0,20927
2	7	0,589154	7E-09	0,108267	0,004004	0,007498	0,012606	0,025977	0,003005	-0,12089
2	7	0,531504	0,001993	0,033063	0,000124	0,000281	0,017915	0,00973	0,005958	-0,04866
2	7	0,865138	6E-09	0,024991	0,008404	1E-09	0,00564	0,000601	0,000119	-0,0306
2	7	0,605885	0,041225	0,011519	0,011403	0,000892	0,020264	0,018034	0,035028	-0,00119
2	7	0,652282	0	0,191057	0,00907	0,00365	0,001823	0,002188	0,007093	-0,19568
2	7	0,709021	0,005821	0,040922	0,014142	0,11835	0,012833	0,001773	0,008089	-0,05894
2	7	0,830103	0,000104	0,064987	0,011666	0,005529	0,002997	0,000273	0,003292	-0,07306
2	7	0,54663	0,014598	0,046221	0,058796	0,001066	0,012354	0,000225	0,025756	-0,08103
2	7	0,93608	2,7E-08	0,000577	0,001946	0,067608	0,014078	0,000204	0,000468	-0,01576

2	7	0,799509	0,003075	0,007328	0,085481	0,004194	0,00681	0,0023	0,001041	-0,08455
2	7	0,666292	0,014587	0,087499	0,018496	0,007452	0,000621	0,018956	0,006377	-0,08856
2	7	0,628853	0,000705	0,014995	0,088654	0,000672	0,041164	0,00495	0,009681	-0,11055
2	7	0,819497	0	0,010718	0,02691	0,004742	0,022988	0,003156	0,001355	-0,04485
2	7	0,431991	0,00216	0,266221	0,003756	0,001749	0,000554	0,010369	6E-09	-0,26421
2	7	0,687898	0,000217	0,020417	0,019733	0,005015	0,016524	0,058316	0,000189	-0,09661
2	7	0,8843	0,000407	0,025036	0,001265	0,002612	0,001939	6,66E-06	0,001833	-0,02503
2	7	0,97257	0	4,17E-05	0,026963	0,000343	0,000173	0	0,002056	-0,02696
2	7	0,463479	0,120657	0,000808	0,065814	0,001811	8,47E-07	0,007119	0,000536	0,052657
2	7	0,745657	0,021615	0,000356	0,003119	0,029029	0,014992	0,015369	0,005537	-0,00799
2	7	0,420286	0,000318	0,121762	0,021567	0	0,006274	0,080744	0,00219	-0,19699
2	7	0,473836	0,039377	0,035979	0,007658	0,01268	0,000587	3,17E-05	0,000512	-0,00426
2	7	0,342269	1E-09	0,000873	0,478316	0,000405	0,000364	0,027865	1E-09	-0,48591
2	7	0,557014	0,098619	0,007167	0,03794	0,028905	0,013803	0,013366	0,006521	0,037993
2	7	0,880817	3,93E-05	0,002176	0,086471	0,004284	0,001715	0,003904	0,000401	-0,08884
2	7	0,28512	0	0,52561	1,91E-05	0,000967	0,004662	0,009094	0,000348	-0,5326
2	7	0,756101	0,014581	0,097263	0,002103	0,009909	0,013195	0,057778	4,3E-05	-0,09223
2	7	0,533109	0	0,120054	0,011061	0	0	0,000253	8,32E-06	-0,12005
2	7	0,493749	0,003867	0,09396	0,001022	0,017246	0,004617	0,003937	0,005251	-0,09378
2	7	0,484738	0,149403	0,045737	0,016536	0,001024	0,025681	0,009471	0,006834	0,068551
2	7	0,488196	0,0025	0,033742	0	4,04E-06	0,028527	5,41E-07	0,00276	-0,058
2	7	0,646739	0,00745	0,0188	0,003309	0,008308	0,003416	0,002217	0,009588	-0,01464
2	7	0,667232	0,10096	0,0354	0,015892	0,002395	0,001236	0,025052	0,000523	0,049935
2	7	0,467826	0,084248	0,097642	0,015292	0,058463	0,010623	0,013074	0,00888	-0,02323
2	7	0,801458	0	0,096628	0,009846	1,01E-05	0	0	0,000133	-0,09954
2	7	0,648546	4,9E-08	0,022396	0,093167	0,000192	0,007518	0,002499	0,000323	-0,10346
2	7	0,816044	0,016979	0,035491	0,008963	0,02002	0,003606	0,005997	0,003756	-0,02034
2	7	0,975039	0,005766	0,000161	7,34E-06	1E-09	1,8E-08	6,07E-07	9,31E-05	0,005605
2	7	0,519224	0,14613	4,5E-05	0,002664	0,000111	0,001832	0,005008	0,041599	0,141121
2	7	0,961542	0,000224	1,62E-07	5,84E-07	1,67E-05	4,89E-06	0,074942	0,035082	-0,07472
2	7	0,460763	4,85E-06	0,177958	0,00104	5,45E-06	0,001098	0,108568	0,000274	-0,17795
2	7	0,844451	9,39E-07	0,049312	8,13E-05	7,24E-06	0,00012	6,73E-05	9,66E-05	-0,04931
2	8	0,361536	0,371941	1E-09	0,021961	0,062008	0,014309	0	0,007652	0,3435
2	8	0,975902	0	1,25E-05	0,000264	0,00076	0	0,014225	2E-06	-0,01423
2	8	0,847637	1E-09	0,300593	1,58E-07	4,7E-08	3,19E-07	5E-09	3,14E-05	-0,30059
2	8	0,9294	0	0,100254	0	0	0	5,85E-05	0,000445	-0,10025
2	8	0,965306	0,001576	0,019494	0,000921	0,000866	0,000221	0,026904	0,000797	-0,04467
2	8	0,791213	0,002574	0,044409	0,008694	0,01562	0,008712	0,00193	0,007967	-0,05089
2	8	0,554188	5,26E-06	7,5E-06	0,004139	0	1,9E-05	0,00173	6,62E-06	-0,00581
2	8	0,962335	8E-09	0,004416	0,000843	0,009198	5E-08	0,000109	0,000437	-0,00523
2	8	0,57304	6,44E-06	0,260109	0,084891	0,000727	0,000647	0,000131	0,000802	-0,32924
2	8	0,636116	0,018211	0,110826	0,005691	1,9E-05	0,009553	0,004174	0,007316	-0,09947
2	8	0,529429	0	0,011082	0,001725	0	0,021001	0,000147	0,004175	-0,03153
2	8	0,882786	0,008086	0,030813	0,006986	0,000416	0,003772	0,007872	0,001275	-0,03394
2	8	0,658586	0,000195	0,063966	4,49E-07	0,001463	0,042331	8,26E-07	0,037691	-0,0874
2	8	0,727473	0	0,121406	0,001011	0,015243	0,003894	0,002647	0,002126	-0,12202
2	8	0,810748	8,3E-08	9,7E-08	0,042399	0,072266	0,005809	2E-09	0,000549	-0,04332
2	8	0,923109	0,000399	0,020817	0,001814	0,003069	0,000206	0,000126	0,010667	-0,02224

2	8	0,633439	0,000929	0,040328	0,039981	0,002249	0,020309	8,45E-05	0,020541	-0,0905
2	8	0,948239	0	0,000875	0,001536	0,021388	0,00415	9,19E-05	7,83E-05	-0,00597
2	8	0,809571	0,000104	0,045191	0,005575	0,015985	0,012275	0,000746	0,000362	-0,05678
2	8	0,81175	0	7,2E-05	0,025704	0,01139	0,000407	1,63E-05	0,005525	-0,02585
2	8	0,578936	0,002037	0,073498	0,012227	0,000409	0,064199	0,001622	0,043002	-0,10992
2	8	0,832214	0	0,01565	0,003856	0,000767	0,030969	0,00587	2,44E-06	-0,04478
2	8	0,726707	3,63E-07	9,32E-06	0,000154	0,058121	2,51E-06	1,9E-07	0,001243	-0,00016
2	8	0,671956	0	0,020194	0,012495	0,008387	0,0624	0,050697	0	-0,09164
2	8	0,810744	0	0,115899	0,006632	0,000435	0,000487	0	0,003271	-0,11631
2	8	0,930043	0	0,023952	0,005644	0,000306	0,000318	2,33E-05	0,002921	-0,02672
2	8	0,507111	0,090836	0,000777	0,036703	0	0,004512	0,062679	0,0053	0,014411
2	8	0,638497	0,090381	0,006845	0,008673	0,000839	0,011883	0,044975	0,011399	0,029523
2	8	0,439462	0,014949	0,121126	0,047068	0,008564	0,034392	0,091239	0,001449	-0,20076
2	8	0,462587	0,050208	0,003508	0,00173	0,046534	0,000978	0,00085	0,000644	0,044326
2	8	0,262107	0	0,004049	0,690432	0,001156	0	0,006113	4,18E-05	-0,69043
2	8	0,537724	0,007869	0,001421	0,078846	0,00949	0,020656	0,050347	0,002507	-0,12188
2	8	0,845998	0,003311	0,00361	0,023259	0,009544	0,000518	0,000121	0,020814	-0,02248
2	8	0,19175	1E-09	0,728244	0	0	0	0,000489	0	-0,72824
2	8	0,869602	0,000531	0,000586	0,007562	0,032623	0,000485	0,0031	0,000619	-0,00888
2	8	0,411081	0,000164	3,4E-08	0,006124	5,99E-05	0,42329	0,001886	7,4E-05	-0,42493
2	8	0,483839	0,00086	0,015027	0,021828	0,073435	0,017181	0,000155	0,002749	-0,04188
2	8	0,503058	0,003059	0,287966	0,001305	1,81E-05	0,004386	6,72E-06	0,002368	-0,28491
2	8	0,46172	0	0,044883	0	0	0,042334	0,001266	0,001691	-0,08639
2	8	0,709631	0,000343	0,020397	0,002691	0,000354	0,000635	0,005611	0,000382	-0,02497
2	8	0,897807	0,036796	0,001758	0	0,002592	5,13E-05	0,006984	0,001021	0,028358
2	8	0,473732	0,151481	0,095384	0,020384	0,013152	0,021456	0,012426	0,00837	0,0354
2	8	0,907897	0	0,077091	1,94E-05	0	0	0	0	-0,07709
2	8	0,555663	0	0,022952	0,117462	0	0,0019	0	0,000358	-0,13202
2	8	0,782287	0,003287	0,055335	0,000435	0,0096	0,0074	0,055457	0,003429	-0,08441
2	8	0,980786	0,001523	4,25E-05	1,94E-06	0	5E-09	1,6E-07	2,46E-05	0,001481
2	8	0,697398	0,022392	6,29E-06	0,000372	1,55E-05	0,000273	0,0007	0,008221	0,021692
2	8	0,960697	6,76E-05	4,9E-08	1,76E-07	5,02E-06	1,48E-06	0,076113	0,020191	-0,07605
2	8	0,445015	1,35E-06	0,189829	0,00029	1,52E-06	0,004879	0,166658	7,63E-05	-0,18983
2	8	0,847282	0,000746	0,031134	0,000148	0,000358	3,55E-05	0,000121	0,000809	-0,03039
3	1	0,774973	0,077271	0,028467	0,013898	0,007792	0,001594	0,027953	0,000619	0,0159
3	1	0,953861	6E-05	0,055729	0,001379	0,000541	0,000261	0,004563	0,001368	-0,05718
3	1	0,981417	3,31E-05	0,022789	0,000141	0,000547	0,000661	0,004573	0,001072	-0,02692
3	1	0,892875	0,001211	0,011503	0,019035	0,002903	0,002664	0,002875	0,002011	-0,02374
3	1	0,470516	0,06658	0,067374	0,001365	0,000473	0,000337	0,07316	0,000503	-0,03093
3	1	0,851832	0,015182	0,068765	0,022687	0,016836	0,000753	0,002481	0,001305	-0,07597
3	1	0,5182	9,11E-05	0,175272	0,144604	0,000715	0,006375	0,008449	0,001995	-0,2977
3	1	0,859152	0,000709	0,029759	0,000642	0,000501	0,009687	0,002813	0,000978	-0,03865
3	1	0,537965	0,011804	0,02382	0,005028	0,000293	0,015163	0,000748	0,003987	-0,02387
3	1	0,891138	0,004586	0,021502	0,001973	0,001201	0,007299	0,001621	0,001744	-0,02092
3	1	0,817453	0,055247	0,009625	0,000626	0,01075	0,015207	0,000682	0,02397	0,031727
3	1	0,681768	0,000265	0,189472	0,003304	0,000978	0,002968	0,000676	0,003422	-0,19126
3	1	0,825964	0,000987	0,016163	0,00932	0,041953	0,003511	0,000168	0,008725	-0,02365
3	1	0,761002	0,000207	0,075589	0,05146	0,008019	0,006334	0,000716	0,002888	-0,11615

3	1	0,686669	0,00027	0,046547	0,030208	0,000144	0,017488	0,000565	0,000136	-0,08386
3	1	0,901628	0,000278	0,013704	0,032932	0,006791	0,011379	0,001647	0,000699	-0,04576
3	1	0,758155	0,00116	0,007455	0,0126	0,012267	0,020878	0,005274	0,001004	-0,03793
3	1	0,417032	0,005796	0,022575	0,227257	0,009425	0,000534	0,00036	0,040055	-0,22146
3	1	0,613189	0,005071	0,012093	0,018425	0,027596	0,055934	0,001194	0,032347	-0,06128
3	1	0,658623	0,008491	0,10745	0,02112	0,001206	0,00283	0,042872	0,007943	-0,14141
3	1	0,672241	5,33E-06	0,07199	0,00478	0,011393	0,001919	0,000534	0,001403	-0,07737
3	1	0,48816	0,01868	0,05461	0,001886	0,015025	0,091156	0,017929	0,003531	-0,11332
3	1	0,895708	0,00352	0,018686	0,002989	0,008244	0,002478	0,000227	0,002889	-0,01805
3	1	0,937628	0,000723	0,016703	0,006321	0,006067	0,002098	0,000623	0,001414	-0,01944
3	1	0,531159	0,014817	0,006447	0,012068	0,00293	0,015436	0,052651	0,016141	-0,04733
3	1	0,617136	0,000337	0,009591	0,030872	0,010507	0,017883	0,008311	0,002124	-0,04657
3	1	0,399549	0,001878	0,173387	0,085718	0,003088	0,009284	0,025675	0,005962	-0,25209
3	1	0,758481	0,00015	0,031928	0,001825	0,001024	0,002395	0,00079	0,003905	-0,03396
3	1	0,590359	0,002109	0,003699	0,155315	0,005213	0,001064	0,022032	0,000542	-0,16968
3	1	0,561929	0,038546	0,007106	0,009438	0,002398	0,04155	0,044713	0,007339	-0,05227
3	1	0,920639	0,000577	0,001412	0,028677	2,1E-08	0,000313	0,010739	0,000762	-0,03381
3	1	0,88005	0,000257	0,036666	0,011576	3,8E-05	0,012344	0,000778	0,00365	-0,03641
3	1	0,412307	0,00024	0,349163	3,81E-05	2,32E-05	0,000145	0,000453	3,87E-05	-0,34892
3	1	0,867095	0,002952	0,055511	0,017325	0,004424	0,000921	0,018206	0,000361	-0,06904
3	1	0,621502	7,4E-08	0,011929	0,014563	0,005327	0,102593	0,004891	3,31E-05	-0,11286
3	1	0,447006	0,002848	0,039135	0,043784	0,039215	0,03964	0,003137	0,012743	-0,09752
3	1	0,547682	0,003065	0,226721	0,006134	0,000136	0,002994	0,012383	0,002605	-0,2289
3	1	0,380801	0,004401	0,097081	0,001951	0,006918	0,166495	0,010037	0,001405	-0,22627
3	1	0,50743	0,129036	0,057628	0,003359	0,023339	0,001274	0,004223	0,00396	0,070799
3	1	0,814371	0,054578	0,008735	0,003405	0,000728	0,000756	0,006264	0,001101	0,041522
3	1	0,528757	0,093671	0,028351	0,013124	0,038482	0,00621	0,004768	0,003913	0,05354
3	1	0,55621	0,004401	0,097081	0,001951	0,006918	0,166495	0,010037	0,001405	-0,22627
3	1	0,628941	0,001288	0,025695	0,112558	0,000394	0,001695	0,011694	0	-0,13273
3	1	0,732706	0,004188	0,02375	0,001243	0,011349	0,002607	0,062473	0,027588	-0,06168
3	1	0,8783	0,005483	0,013632	0	0,00428	0,003596	0,015698	0,003908	-0,0135
3	1	0,967397	0,00097	0,001366	0,00041	0,020974	0,008214	0,000159	0,002084	-0,00724
3	1	0,482817	0,136906	0,004549	0,053118	0,001329	0,007616	0,044009	0,004349	0,069101
3	1	0,6588	0,003792	0,006121	1,23E-07	8,8E-05	1E-09	0,034405	0,012215	-0,03061
3	1	0,674456	0,141723	0,001027	0,020358	0,005112	0,017924	0,002118	0,015016	0,109888
3	1	0,543691	0,002666	0	0,014995	0,007441	0,002364	0,010365	0,016328	-0,01736
3	2	0,997404	6,14E-07	0,001368	7E-09	1,53E-06	0	0,000235	0,000863	-0,00137
3	2	0,921392	0,000508	0,093962	0,003194	0,003347	0,000349	0,013027	0,003139	-0,10383
3	2	0,892905	6,03E-05	0,009397	0,021701	0,001188	0,003775	0,000594	0,00148	-0,02768
3	2	0,545894	0,041183	0,000746	0,005848	0,000115	1,05E-05	0,002357	0,000318	0,033078
3	2	0,930777	0,000915	0,001213	0,016399	0,0047	0,00813	0,003675	0,000731	-0,0212
3	2	0,365598	0,000353	0,395803	0,081962	0,00078	0,007871	0,016829	0,005208	-0,4573
3	2	0,650836	0,001416	0,178825	0,000199	0,000415	0,00442	0,000201	0,00199	-0,17741
3	2	0,490082	0,087773	0,020285	0,003667	0,000587	0,017312	0,009153	0,004068	0,048738
3	2	0,837341	0,020053	0,05137	0,000848	0,012895	0,011481	0,006946	0,001542	-0,03695
3	2	0,728269	0,002198	0,033659	0,000728	0,00346	0,032189	0,001132	0,033757	-0,05837
3	2	0,736184	0,000159	0,122461	0,00852	0,003181	0,009732	0,00064	0,003621	-0,13078
3	2	0,830325	0,000981	0,034139	0,008981	0,032625	0,015357	0,000557	0,000773	-0,0518

3	2	0,817842	0,000433	0,021852	0,049652	0,003475	0,002716	0,000225	0,004559	-0,06736
3	2	0,541728	0,008019	0,036694	0,130951	0,00601	0,022723	0,000773	0,022918	-0,15089
3	2	0,904288	0,001794	0,022441	0,017564	0,023693	0,004858	0,002082	0,000717	-0,03082
3	2	0,846468	0,001036	0,00538	0,039643	0,004144	0,003823	0,002369	0,000957	-0,04375
3	2	0,485046	0,112855	0,00823	0,017707	0,004221	0,000223	0,002013	0,009746	0,088981
3	2	0,609708	0,035124	0,029357	0,019407	0,015946	0,005034	0,001784	0,046358	-0,01517
3	2	0,627718	0,064945	0,041775	0,014158	0,000687	0,016366	0,014199	0,002422	-0,0005
3	2	0,639709	0,001608	0,108184	0,018166	0,013957	0,000476	0,010223	0,001621	-0,11501
3	2	0,557007	0,003363	0,07615	0,009695	0,006876	0,042366	0,013206	0,000559	-0,10334
3	2	0,943991	0,000946	0,001828	2,63E-07	0,001317	0,001125	4,79E-06	0,010623	-0,00172
3	2	0,937235	0,001581	0,002837	0,00192	0,007051	0,001723	0,000615	0,002092	-0,00327
3	2	0,490907	0,107389	0,001011	0,044594	0,008998	0,014855	0,003766	0,011437	0,059266
3	2	0,600894	0,018259	0,019993	0,019371	0,005118	0,008219	0,045324	0,002268	-0,05888
3	2	0,399466	0,001876	0,163378	0,044037	0,011901	0,047459	0,108275	0,007161	-0,22251
3	2	0,463062	0,08261	0,023029	0,003006	0,041244	0,023632	0,000647	0,002031	0,041423
3	2	0,713079	0	0,004485	0,096803	0,002787	0,001148	0,014038	0,000768	-0,10588
3	2	0,70679	0,003942	0,040339	0,006227	0,009325	0,015492	1,7E-08	0,006175	-0,04596
3	2	0,862812	1,92E-05	0,00801	0,145467	0,002862	0,000838	0,000153	0,002277	-0,14587
3	2	0,832147	0,000472	0,029625	0,029824	0,011017	0,007684	0,001463	0,003664	-0,04064
3	2	0,4374	0,001231	0,261115	0,002301	0,000216	0,002161	0,009457	0,002803	-0,26301
3	2	0,837594	0,000587	0,118193	0,001471	0,008353	0,002113	0,033429	0,000306	-0,11834
3	2	0,559884	2,71E-05	0,058064	0,011327	0,000177	0,064216	0,033049	4,11E-05	-0,12475
3	2	0,470382	0,005733	0,070404	0,000148	0,020558	7,17E-05	0,033255	0,004458	-0,07755
3	2	0,492022	0,011288	0,284464	0,013138	0,000231	0,002624	0,011877	0,003065	-0,28556
3	2	0,383217	0,013648	0,087555	0,00805	3E-06	0,15211	0,002988	0,007177	-0,21796
3	2	0,542186	0,004661	0,002113	5E-09	0,020784	0,00489	0,004725	0,001598	-0,00526
3	2	0,839551	0,041696	0,012764	0,00047	0,001596	9,85E-05	0,001151	0,002237	0,028668
3	2	0,55725	0,058385	0,103257	0,021831	0,042149	0,022117	0,003518	0,001665	-0,06223
3	2	0,9214	6,75E-05	0,060279	0,011038	0,001528	0	0,003007	0,000328	-0,07411
3	2	0,624059	0	0,050856	0,031518	0,012236	1,65E-06	0,005921	0,000207	-0,07408
3	2	0,736691	0,008246	0,010165	0,00091	0,004426	0,007227	0,048774	0,01586	-0,05089
3	2	0,740392	0,069928	0,006742	0	0,00851	0,007649	0,028696	0,011041	0,041232
3	2	0,896498	7E-05	1,12E-05	1,2E-06	0,034415	0,028579	1,48E-06	0,009176	-0,02851
3	2	0,57735	0,000331	0,01113	0,010038	4,98E-07	0,035967	0,001531	0,024844	-0,03564
3	2	0,731246	0,000103	0,001221	0,028646	0,098518	0,006432	0,000175	0,00946	-0,03056
3	2	0,675656	0,008341	0,007642	0,051857	7,2E-05	0,017157	0,016845	0,001453	-0,04352
3	2	0,582688	0,004393	0	0,009536	0,045943	0,0113	0,000527	0,001449	-0,01349
3	2	0,759422	9,11E-05	0,115075	0,001935	0,002255	0,004792	0,001454	0,001647	0,124636
3	3	0,933217	0	0,112366	1,73E-06	0	0	0	0,000198	-0,11237
3	3	0,976066	5,64E-06	0,026783	9,17E-05	0,000343	0,000523	0,001321	0,001308	-0,02797
3	3	0,83279	0,001381	0,030849	0,02395	0,003215	0,002883	0,002665	0,003657	-0,0381
3	3	0,505575	0,005103	0,059941	0,050313	0,00708	0	0,044193	9,23E-05	-0,10821
3	3	0,408365	1,41E-05	0,218363	0,185209	0,000404	0,000658	0,002422	0,001093	-0,37652
3	3	0,670286	0,000257	0,12992	0,000501	0,000894	0,018657	0,002989	0,000588	-0,13704
3	3	0,540705	0,019275	0,004255	0,001808	0,000293	0,036145	0,00036	0,029979	-0,02075
3	3	0,830057	0,011192	0,028401	0,015692	0,012988	0,009532	0,005265	0,004316	-0,03233
3	3	0,737823	0,001355	0,115075	0,002589	1,81E-05	0,042891	0,00476	0,01113	-0,12645
3	3	0,759422	9,11E-05	0,124146	0,001935	0,002255	0,004792	0,001454	0,001647	-0,12764

3	3	0,757251	0,000972	0,074012	0,010858	0,008448	0,017611	0,020757	0,002261	-0,11275
3	3	0,861994	0,000155	0,065079	0,004741	0,003456	0,006059	0,001132	0,004374	-0,07095
3	3	0,450472	0,004986	0,078417	0,336771	0,005651	0,00037	0,002581	0,001881	-0,40372
3	3	0,893284	0,005068	0,007335	0,031358	0,006606	0,006291	0,000599	0,000685	-0,03614
3	3	0,679119	0,001023	0,021176	0,129126	0,009506	0,007742	0,003196	0,002091	-0,14833
3	3	0,460669	0,13614	0,002378	0,04531	0,015651	0,000178	0,000773	0,036488	0,08934
3	3	0,589882	0,004747	0,025281	0,013085	0,005592	0,066523	0,001173	0,001404	-0,07978
3	3	0,669887	0,018963	0,108898	0,027601	0,007769	0,006957	0,012448	0,089611	-0,11577
3	3	0,485215	4,6E-06	0,224143	0,031723	0	0,018656	3,52E-05	0,032512	-0,23198
3	3	0,921658	2,92E-05	0,031114	0,001207	0,000232	0,001572	6,74E-06	0,011605	-0,03121
3	3	0,941617	6,63E-05	0,010355	0,001094	0,004983	0,000527	0,000715	0,00393	-0,01069
3	3	0,54965	0,012496	0,004703	0	0,035862	0,035072	0,005986	0,009275	-0,02647
3	3	0,386998	0,180911	0,085475	0,000138	0,058366	1E-08	0,172931	0	-0,0466
3	3	0,507087	0,007527	0,042631	0,027133	0,000444	0,040758	0,043554	0,003426	-0,09249
3	3	0,54606	0,001277	0,019855	9,2E-05	9,55E-06	0,032125	8,97E-05	0,001573	-0,04234
3	3	0,745564	0,003595	0,001014	0,02305	0,01769	0,004033	0,00609	0,000266	-0,02373
3	3	0,632895	0,020989	0,02707	0,007669	0,003251	0,010925	0,000653	0,01309	-0,01888
3	3	0,885632	7,23E-07	0,007027	0,084822	1,26E-05	0,000149	0,001138	0,000716	-0,08816
3	3	0,908204	0,000314	0,023576	0,019015	0,002644	0,006411	0,000517	0,003262	-0,03375
3	3	0,333623	0,001633	0,48124	0,000637	0,000344	0,001019	0,000787	0,000132	-0,47992
3	3	0,948848	2,53E-06	0,004052	0,008981	0,016692	0,002418	0,001387	0,001896	-0,01158
3	3	0,512301	5,77E-05	0,089107	0,008788	0,000148	0,086929	0,002379	0,000828	-0,15251
3	3	0,513326	0,027299	0,012636	0,00142	0,015284	0	0,000581	0,034183	0,013029
3	3	0,757489	0,003438	0,03928	0,077409	0,000652	0,050461	0,005273	0,001251	-0,12101
3	3	0,463172	0,024168	0,073554	5E-08	0,004682	0,016646	9,64E-07	0,004642	-0,06238
3	3	0,650777	0,052697	0,001905	4,38E-05	0,007426	0,005515	0,001544	0,002918	0,044547
3	3	0,630722	0,143011	0,042637	0,007071	0,002335	0,001546	0,017904	2,61E-05	0,08661
3	3	0,654196	0,003515	0,007557	0,002133	0,039211	0,009767	0,000413	0,000468	-0,01114
3	3	0,971966	8,89E-06	0	0,003825	0,004811	0	0,000523	0,001118	-0,00434
3	3	0,698724	0,002788	0,000184	0,004373	1,41E-05	0	0,141818	0,000108	-0,13998
3	3	0,850456	0,021421	0,015507	0,000561	0,006039	0,031305	0,006235	0,00212	-0,02492
3	3	0,883248	0,0145	0,000939	0	0,001223	0,00465	0,0041	0,0056	0,009392
3	3	0,774824	0,001657	1,43E-05	6E-09	0,019448	0,004882	7E-09	0,017705	-0,00322
3	3	0,590933	0,000583	0,026154	0,009032	8,78E-07	0,03404	0,002699	0,014701	-0,03969
3	3	0,808227	9,13E-06	1,32E-07	3,09E-05	0,074355	0,001803	7,8E-08	6,55E-06	-0,00179
3	3	0,675422	0,054077	0,001195	0,006877	0,011983	0,045061	0,004914	0,006667	0,009016
3	3	0,534607	0,064261	0	0,006516	0,022776	0,031461	5,33E-05	0,001583	0,028273
3	3	0,501149	0,114114	0,034442	0,003825	6,71E-05	0,026605	8,69E-05	0,006046	-0,05969
3	3	0,60922	0,057223	0,021463	0,001336	0,009393	0,019518	0,001236	0,00212	0,022884
3	3	0,654196	7,12E-06	0,007557	0,002133	0,039211	0,009767	0,000413	0,000468	0,011144
3	4	0,971184	0,004211	2,06E-05	8,66E-06	0,00174	0,000236	0,012854	7,82E-06	-0,00871
3	4	0,943556	0	0,094217	0	5,37E-07	0	9E-09	6,7E-08	-0,09422
3	4	0,95363	0	0,083466	4E-09	0,001243	4E-09	0	0,003586	-0,08347
3	4	0,946638	0,001844	7,31E-05	0,019606	0,000371	0,00116	0,005142	0,00424	-0,02136
3	4	0,617436	0	0,03071	0,0321	0,001802	0	0,054691	8,67E-05	-0,09412
3	4	0,876678	0,001889	0,060425	0,001876	0,008567	0,000236	0,001823	0,001457	-0,05863
3	4	0,590036	0,000109	0,188629	0,055875	0,000549	0,000494	0,006864	0,000562	-0,2374
3	4	0,797404	0,000214	0,040235	0,000326	2,22E-05	0,010912	7,72E-05	0,000535	-0,04978

3	4	0,537838	0,001477	0,013714	0,005345	0,001478	0,006373	0,000928	0,004153	-0,02191
3	4	0,935643	0,000192	0,023284	9,16E-05	5E-09	0,005801	0,000247	2,35E-05	-0,0249
3	4	0,771415	0,000905	0,062781	2,23E-05	0,000645	0,049739	0,000137	0,012266	-0,0791
3	4	0,760611	4,01E-07	0,127144	0,001876	0,001579	0,000939	0,000279	0,001746	-0,12851
3	4	0,84726	7,39E-07	0,007632	0,026149	0,00222	0,020916	4,23E-05	0,000176	-0,04081
3	4	0,816147	0,000175	0,045892	0,037471	0,001814	0,000765	0,001095	0,013271	-0,07459
3	4	0,660186	0,003098	0,034031	0,019496	0,00237	0,024288	0,002371	8,04E-05	-0,05551
3	4	0,819534	0,003923	3,84E-07	0,067374	0,044384	0,018428	3,55E-05	0,000359	-0,07496
3	4	0,756763	0,000534	0,01692	0,119582	0,002345	0,008533	0,00021	0,000788	-0,13287
3	4	0,830803	0	0,004314	0,036312	0,022283	0,00033	0,000317	0,005396	-0,03905
3	4	0,596461	0,002918	0,030866	0,009967	0,000195	0,041137	0,000616	0,007151	-0,06696
3	4	0,693552	0,000151	0,112917	0,002588	0,009036	0,002014	0,012615	0	-0,11455
3	4	0,517079	6,95E-05	0,232335	0,001652	0,013034	0,000145	0	0,000133	-0,23261
3	4	0,522474	0,009979	0,03122	0,007202	0,003018	0,14894	0,024089	0,003117	-0,16162
3	4	0,935055	0,005288	0,001465	0,000425	0,000743	0,000759	4,85E-06	0,002586	0,002829
3	4	0,913527	0,002703	0,00922	0,001363	0,004916	0,001067	0,000638	0,001451	-0,0073
3	4	0,640307	0	0,018299	0,030995	0,028242	0,055789	0,010162	0,005583	-0,07391
3	4	0,759908	0,002287	0,01928	0,001316	0,000655	0,015009	4,3E-08	0,002015	-0,02943
3	4	0,465555	0,015974	0,053422	0,062916	0,002849	0,025368	0,039046	0,000822	-0,13159
3	4	0,475168	0,048498	0,027397	0,016991	0,024218	0,006916	0,001055	0,003508	0,002699
3	4	0,675025	0,002274	0,005369	0,023176	0,019137	0,010479	0,007662	0,000846	-0,03122
3	4	0,600019	0,001252	0,016602	0,039134	0,00587	0,022943	0,032938	0,008673	-0,08913
3	4	0,930616	4,41E-05	0,00557	0,061221	0,00224	0,000667	0,001068	0,004649	-0,06237
3	4	0,894505	0,000442	0,017078	0,010334	2,38E-06	0,003174	0,001446	0,005659	-0,01664
3	4	0,32988	0,000168	0,465245	0,001653	0,000178	0,003169	0,00239	0,000231	-0,4675
3	4	0,898319	0,000229	0,066571	0,001489	0,002072	0,000361	0,01674	0,00021	-0,06789
3	4	0,397588	0,000208	0,00227	0,004568	0,000144	0,314418	0,004173	0,001126	-0,31923
3	4	0,539603	0,004135	0,000965	0,012745	0,012847	0,060374	1,17E-05	0,003221	-0,06028
3	4	0,611251	0,003552	0,074857	0,006084	0,000283	0,025114	0,004898	0,00403	-0,09458
3	4	0,511058	0,002548	0,053402	0	4,76E-07	0,019168	0	0,005917	-0,06801
3	4	0,539796	0,090304	0,005824	0,000218	0,047491	0,000458	0,007868	0,001745	0,078983
3	4	0,79312	0,014007	0,00349	0,010699	0,005341	7,01E-05	0,004772	0,000103	-0,00413
3	4	0,735751	0,006387	0,007701	3E-06	0,010655	0,024412	0,001134	0,005212	-0,02196
3	4	0,953774	2,3E-08	0	0,003635	0,000199	0	0,009911	0,001568	-0,01354
3	4	0,768186	0,007696	0,000438	0,009033	7,04E-05	0	0,030495	0,007215	-0,02637
3	4	0,823485	6,6E-05	0,016523	0,001096	0,003143	0,019803	0,028776	0,002537	-0,04488
3	4	0,924223	0,003395	0,00022	0	0,000286	0,006755	0,00096	0,005607	-0,00336
3	4	0,698249	0,017305	0,01527	0,000611	0,04033	0,047441	0,000238	0,021542	-0,03132
3	4	0,594214	0,016473	0,014535	0,057624	2,48E-05	0,016043	0,033002	0,023663	-0,05656
3	4	0,799533	0,002447	0,014673	0,000142	0,076433	0,008026	0,000439	0,002223	-0,02027
3	4	0,664304	0,080196	0,000132	0,003363	0,008948	0,067614	0,000487	0,005516	0,012582
3	4	0,528182	0,067031	0	0,071806	0,003433	0,010746	0,001323	0,000946	-0,00526
4	1	0,921307	0,035533	0,01968	0,000138	0,000967	0,001134	0,016182	0,000214	-0,00065
4	1	0,95331	0,000246	0,055025	0,000433	0,000185	6,9E-05	0,003872	0,002754	-0,05511
4	1	0,448086	0,207844	0,025295	0,005942	0,001696	0,005652	0,010392	0,005277	0,169843
4	1	0,635717	0,002923	0,035199	0,007098	0,003854	0,000359	0,066545	0,000297	-0,07019
4	1	0,852894	0,001734	0,035666	0,033982	0,002488	0,005351	0,00423	0,002494	-0,06637
4	1	0,598644	0,000632	0,120091	0,12101	0,000851	0,000201	0,016098	0,007267	-0,23046

4	1	0,901003	0,004031	0,024717	0,000666	0,000951	0,001975	0,000287	0,001868	-0,02145
4	1	0,512685	0,076027	0,016485	4,28E-05	0,017028	0,008627	0,003604	0,004019	0,051383
4	1	0,827941	0,06568	0,014772	0,005685	0,002709	0,008489	0,001669	0,001055	0,042068
4	1	0,60922	0,057223	0,021463	0,001336	0,009393	0,019518	0,001236	0,037152	0,022884
4	1	0,815496	5,78E-05	0,080099	0,007579	0,004279	0,002041	0,000284	0,002926	-0,08249
4	1	0,769232	0,001207	0,042981	0,018091	0,011289	0,007977	0,00388	0,00324	-0,05789
4	1	0,859233	0,000609	0,044248	0,01337	0,005648	0,00389	0,000579	0,002998	-0,05326
4	1	0,635669	0,00539	0,018621	0,105253	0,000292	0,008331	0,000246	0,001029	-0,10959
4	1	0,879016	0,015324	0,007536	0,013846	0,009573	0,005676	0,00169	0,000278	-0,00818
4	1	0,710798	0,00173	0,022604	0,03666	0,004847	0,011257	0,000688	0,000705	-0,04901
4	1	0,51932	0,197472	0,006763	0,008597	0,008297	0,000186	0,000278	0,002534	0,18401
4	1	0,616859	0,018204	0,014248	0,031747	0,005419	0,035627	0,00116	0,009725	-0,05328
4	1	0,64898	0,146909	0,018528	0,007758	0,0059	0,002761	0,021907	0,002091	0,110184
4	1	0,503707	0,000471	0,225034	0,01119	0,012991	9,36E-05	0,005232	0,00204	-0,22914
4	1	0,509783	0,10785	0,010926	0,026062	0,005341	0,003229	0,014523	0,004395	0,064038
4	1	0,759454	0,128684	0,01052	0,006236	0,001433	0,000914	0,00152	0,005469	0,112237
4	1	0,806231	0,059052	0,008643	0,00096	0,009406	0,004464	0,001086	0,007051	0,045898
4	1	0,440256	0,12001	0,001143	0,013541	0,002743	0,008754	0,038329	0,00237	0,074833
4	1	0,545295	0,087807	0,003788	0,042183	0,028191	0,015936	0,075319	0,004365	-0,02225
4	1	0,461713	0,027767	0,092724	0,049723	0,001029	1,36E-07	0,053752	0,020699	-0,11559
4	1	0,347492	0,308789	0,004477	0,005217	0,000915	0,000808	0,001837	0,002748	0,299366
4	1	0,486983	0	0,001328	0,29944	0,010627	0,003532	0,020367	0,000495	-0,30741
4	1	0,533987	0,067313	0,009756	0,018957	0,002939	0,037098	0,025118	0,01362	0,001818
4	1	0,790467	3,52E-05	0,004773	0,191614	1,47E-05	0,001875	0,000333	0,001283	-0,19378
4	1	0,57135	0,006888	0,133368	0,000171	0,001524	0,018893	0,003077	0,002036	-0,14549
4	1	0,897172	0,010614	0,053147	0,000706	0,006348	0,001126	0,012288	0,000357	-0,04383
4	1	0,580537	0,055333	0,026362	0,02076	0,003521	0,113607	0,00158	0,000928	-0,09085
4	1	0,401995	0,093802	0,031277	0,008367	0,054738	0,051964	0,017397	0,004452	0,005172
4	1	0,501149	0,114114	0,034442	0,00511	6,71E-05	0,026605	8,69E-05	0,006046	0,05969
4	1	0,461144	0,00155	0,110629	1,04E-05	2,86E-05	0,021719	0,012644	0,001995	-0,12174
4	1	0,501401	0,019765	0,003247	1,65E-06	0,002458	0,001475	0,000134	0,0044	0,015528
4	1	0,792432	0,047498	0,023401	0,007511	0,001587	0,002006	0,001072	0,001072	0,017017
4	1	0,427736	0,180215	0,020784	0,011747	0,063516	0,031041	0,016369	0,001516	0,123756
4	1	0,646212	0,031966	0,005819	0,024952	0,005242	0,015102	0,009827	0,001682	-0,01535
4	1	0,344181	0,378451	0,000536	0,006973	0,008975	0,004352	0,035228	0,000943	0,337838
4	1	0,646454	0,160224	0,01033	0,001586	0,008294	0,00549	0,018781	0,020475	0,12887
4	1	0,628659	0,004906	0,062074	0,123074	0,00681	0,0072	0,020098	0,017214	-0,13442
4	1	0,3849	0,230422	0,006703	0,00212	0,000715	0,00035	0,001954	0,003133	0,223638
4	1	0,818283	0,011084	0,011921	0,040881	0,029919	0,003694	0,034683	0,001686	-0,06539
4	1	0,573203	0,000823	0,019034	0,01911	0,004957	0,000561	0,003285	0,000962	-0,02919
4	1	0,8129	0,000463	0,047932	0,015875	0,005948	0,001572	0,002112	9,62E-05	-0,05544
4	1	0,718596	0,006345	0,061246	0,00046	0,000479	0,001345	0,043094	0,001478	-0,07947
4	1	0,588315	0,002987	0,111317	0,098517	0,000602	0,008149	0,012515	0,000352	-0,18018
4	1	0,799732	0,034333	0,005633	0,050119	0,002395	0,003039	0,020294	0,006991	-0,02084
4	2	0,975822	5,05E-06	0,024146	0,000151	0,004782	1,77E-07	0,001336	0,001309	-0,02539
4	2	0,959248	0,001019	0,059927	5,44E-05	0,000139	6,16E-06	0,000827	9,18E-05	-0,05949
4	2	0,516658	0,044055	0,066037	0,004752	0,026936	0,020383	0,003046	0,008126	-0,03033
4	2	0,576884	0,002186	0,045086	0,035619	0,001925	0,000318	0,033812	0,000997	-0,08598

4	2	0,850002	0,001615	0,052372	0,007573	0,022457	0,000776	0,003613	0,00453	-0,05846
4	2	0,319089	0,000118	0,155443	0,339428	0,000162	0,00531	0,006123	0,00325	-0,4825
4	2	0,727051	0,001512	0,235275	0,004058	0,000917	0,012942	0,008908	0,001401	-0,23541
4	2	0,374302	0,341921	6E-05	1,34E-05	0,004908	0,001375	3E-09	0,018642	0,340517
4	2	0,941822	0	0,001319	0,003823	0,00011	0,007498	0,00154	0,001101	-0,01027
4	2	0,773888	0,000353	0,050315	0,002498	0,002192	0,015548	7,78E-05	0,006018	-0,05737
4	2	0,744871	3,28E-05	0,148034	0,003585	0,000691	0,010738	0,00448	0,000138	-0,14945
4	2	0,736317	0,003603	0,051529	0,052423	0,020098	0,004531	0,000111	0,016368	-0,09736
4	2	0,818978	0,00024	0,080793	0,034105	0,005079	0,001797	0,000848	0,002405	-0,11003
4	2	0,538849	0	0,099914	0,117782	0,000235	0,011328	0,000703	0,005955	-0,21017
4	2	0,722118	0,011732	7,74E-06	0,008347	0,127091	0,006847	0,000103	0,000799	-0,00252
4	2	0,841588	9,22E-06	0,011993	0,043332	0,003574	0,011036	0,000165	0,001766	-0,05121
4	2	0,44886	0,23932	0,000408	0,012109	0,005405	0,000251	0,000246	0,011639	0,227017
4	2	0,631071	0,030801	0,002847	0,014354	7,71E-05	0,018807	0,000858	0,00878	0,002131
4	2	0,79435	0,000142	0,002216	0,029135	0,02964	0,003947	1,42E-06	0,001674	-0,03175
4	2	0,62425	0,000624	0,099606	0,01912	0,04108	3,2E-06	0,001049	0,00352	-0,11532
4	2	0,772476	0,00169	0,004538	0,054522	0,004355	0,00175	0,005808	0,007575	-0,06001
4	2	0,796307	0,115027	0,01177	0,000679	0,000111	0,000891	0,000369	0,004763	0,102735
4	2	0,93572	0	0,016898	0,003644	0,000774	0,000386	7,24E-06	0,001602	-0,01721
4	2	0,512683	0,060216	0,001127	0,002915	0,021416	0,005224	0,006652	0,01576	0,047635
4	2	0,458859	0,018008	0,018813	0,06492	0,000842	0,000755	0,127952	0,002713	-0,16889
4	2	0,384215	0,128766	0,003772	0,003114	0,10612	0,040696	0,000273	0,00446	0,082455
4	2	0,704462	0,005479	0	0,009645	0,008615	0,0005	0,001349	0,000696	-0,00559
4	2	0,618435	0,019872	0,003445	0,030291	0,010623	0,041811	0,004994	0,003875	-0,04033
4	2	0,93319	0	0,003967	0,031888	0,001478	0,001012	0,005648	0,006209	-0,03964
4	2	0,633761	9E-09	0,085283	0	0,000249	0,011878	0,000723	0	-0,09327
4	2	0,882894	0,000192	0,048633	6,71E-06	0,005776	0,001102	0,021248	6,14E-05	-0,06312
4	2	0,575706	6,97E-07	0,001765	0,002051	0,000181	0,180104	0,008013	0,00385	-0,19069
4	2	0,562954	0,005183	0,004491	0,005855	0,009847	0,000606	0,002474	0,005632	-0,00515
4	2	0,530356	0,029427	0,078853	0,022371	0,000393	0,000975	0,002599	0,00172	-0,05853
4	2	0,462201	0,021466	0,057901	0,000205	0,000169	0,042413	0,001832	0,008464	-0,06915
4	2	0,577036	0,003087	0,017609	0,000492	0,019338	0,003145	0,002964	0,003101	-0,01749
4	2	0,747421	0,049896	0,010415	0,000184	0,000193	0,001472	0,001045	0,000529	0,037315
4	2	0,451688	0,111272	0,033807	0,025344	0,064509	0,030765	0,019829	0,005932	0,037099
4	2	0,463919	0,161491	0,004124	0,034032	0,02584	0	0,005505	0,00589	0,126833
4	2	0,49491	0,258261	3,99E-05	0,014999	0,032934	0,002428	0,06441	0,002003	0,183545
4	2	0,675639	0,014749	0,023729	0,012817	0,043466	4,76E-05	0,056775	0,015092	-0,05438
4	2	0,281932	0,631661	4,47E-05	0,02751	0,008568	0,000449	1,48E-05	0,002422	0,604152
4	2	0,287936	0,427537	0,001652	0,00251	0,004497	1,61E-05	0,000721	0,015331	0,424885
4	2	0,88087	0,000853	0,05053	0,002853	0,122246	0	4,76E-05	0,001318	-0,04968
4	2	0,666143	0,043499	0,024823	0,02823	0,000134	0,000448	9,15E-05	0,003197	-0,00665
4	2	0,763647	0,001214	0,0369	0,031489	0,001359	0,000528	0,011186	0,000824	-0,06613
4	2	0,803563	0,000234	0,015667	0,002676	0,024703	0,018625	0,001484	0,005888	-0,02349
4	2	0,953643	0,00013	0,003493	0,009232	0,000263	0,000376	4,06E-05	1,68E-05	-0,01235
4	2	0,955032	0,01331	0,009525	0,001131	0,002108	6,05E-06	0,004313	0,00877	0,001483
4	2	0,586147	0,631661	0,036948	0,026296	0,00041	0,000241	0,059758	0,000416	0,082302
5	1	0,672802	0,006927	0,243502	0,0937	0,004377	0,00026	0,001859	0,001083	-0,25607
5	1	0,979808	3,49E-06	0,016269	1,12E-06	4,17E-05	0	0,000424	0,000332	-0,01641

5	1	0,98693	4,62E-06	0,00271	5,37E-05	0,00133	0,000821	0,001546	0,00225	-0,00452
5	1	0,849718	0,001769	0,041162	0,017938	0,001453	0,00231	0,001182	0,007972	-0,0526
5	1	0,586147	0,006771	0,036948	0,026296	0,00041	0,000241	0,059758	0,000416	-0,0823
5	1	0,897825	0,001264	0,027253	0,021285	0,015485	0,00531	0,001103	0,006306	-0,04319
5	1	0,677851	5,34E-05	0,160177	0,093999	0,000939	0,001319	0,004389	0,004138	-0,24215
5	1	0,52171	0,128129	0,027888	0	0,002296	0,014446	0,000911	0,005278	0,095821
5	1	0,547844	0,006732	0,009435	0,001147	0,018149	0,010775	0,008845	0,003241	-0,01632
5	1	0,895331	0,02114	0,018349	0,001407	0,007441	0,00144	0,002317	0,002126	0,00059
5	1	0,793217	0,004302	0,016602	0,000594	0,003192	0,023368	0,000173	0,040844	-0,03301
5	1	0,752408	0,000155	0,071318	0,007378	0,002174	0,005047	0,000382	0,002215	-0,0748
5	1	0,739664	0,005007	0,090097	0,01639	0,038029	0,012739	0,004582	0,000913	-0,10818
5	1	0,847705	0,000707	0,032622	0,036386	0,003036	0,000935	0,000526	0,005243	-0,06627
5	1	0,629387	5,41E-06	0,017836	0,177321	0,000161	0,012322	0,000548	0,000334	-0,1807
5	1	0,925452	0,000795	0,008825	0,015442	0,006085	0,002914	0,000576	0,000949	-0,02428
5	1	0,738842	0,000921	0,024562	0,101285	0,00289	0,011355	0,002367	0,001989	-0,11342
5	1	0,678848	0,001523	0,011919	0,001461	0,008062	5,09E-05	0,001046	0,013139	-0,01159
5	1	0,600484	0,020235	0,016929	0,066208	8,57E-06	0,034585	0,005379	0,000316	-0,06664
5	1	0,786643	0,002332	0,032748	0,003867	0,004206	0,003975	0,00492	0,004352	-0,03711
5	1	0,806051	3,52E-05	0,053291	0,02235	0,001044	0,000511	0,00098	0,00107	-0,06832
5	1	0,912202	0,003666	0,027961	2,7E-05	0,007177	0,001955	8E-05	0,005453	-0,02572
5	1	0,897306	0,000535	0,042739	0,004222	0,004854	0,000714	0,000492	0,000784	-0,04489
5	1	0,55801	0,124672	0,002495	0,057969	0,004802	0,005925	0,0091	0,064849	0,063421
5	1	0,626447	0,035035	0,043806	0,012421	0,005627	0,003694	0,016662	0,0135	-0,02289
5	1	0,466814	0,006831	0,06695	0,00732	0,002532	0,038206	0,090778	0,000249	-0,12302
5	1	0,535583	0,017219	0,040566	0,003302	0,018574	0,000765	0,005347	0,003395	-0,02402
5	1	0,576161	0,006673	0,000132	0,077726	0,002183	0,001308	0,032125	0,001001	-0,09417
5	1	0,622134	0,035186	0,005057	0,046599	0,012289	0,019869	0,02346	0,009367	-0,04976
5	1	0,897047	8,86E-05	0,002524	0,073244	0,002989	0,001264	1,99E-06	0,005817	-0,07409
5	1	0,395798	0,001106	0,469129	0,000688	8,5E-08	0,00184	0,006511	0,000214	-0,46835
5	1	0,78738	0,002589	0,086172	0,00726	0,033483	0,004245	0,010218	0,002185	-0,08825
5	1	0,525261	5E-09	0,095651	0,00586	3,48E-05	0,018089	0,006553	7,79E-05	-0,10941
5	1	0,67183	0,000302	0,004003	0,000515	0,005171	0,001099	0,001432	4,41E-06	-0,00489
5	1	0,565047	0,059725	0,049805	0,010882	0,000107	0,019108	6,15E-05	0,000672	0,002915
5	1	0,490526	0,020671	0,078355	0,001554	0,000483	0,049174	0,005379	0,004838	-0,09262
5	1	0,632335	0,020656	0,003948	0,001045	0,009478	0,004617	0,000916	0,00139	0,012625
5	1	0,834249	0,060635	0,008885	0,002304	0,011871	0,000343	0,007211	0,000425	0,046161
5	1	0,548501	0,073291	0,028946	0,010129	0,021513	0,038478	0,002399	0,004435	0,007286
5	1	0,792539	3,11E-07	0,029852	0,01453	0,001112	3,28E-05	0,03062	0,007957	-0,03453
5	1	0,733155	0,002593	0,005712	0,003817	3,17E-05	0,006602	0,03635	0,006868	-0,04002
5	1	0,832026	0,001771	0,061325	0,000603	0,023564	0,010683	0,016095	0,006629	-0,06297
5	1	0,858448	0,00162	0,007655	0,001345	0,017669	0,00847	0,005473	0,004968	-0,01358
5	1	0,836092	0	0,028324	0,01427	8,34E-06	1,58E-06	0,000808	5,72E-05	-0,03464
5	1	0,642384	0,131844	0	0,004606	0,004164	0,00052	0,00055	0,002373	0,127238
5	1	0,539648	0,004944	0,008663	0,060197	0,071142	0,005463	0,005181	0,016488	-0,05819
5	1	0,625105	0,086891	0,012635	0,006355	0,008108	0,002184	0,037724	0,000173	0,047905
5	1	0,649513	0,019514	0,009873	0,003503	6,93E-07	2,35E-05	0,00233	0,000483	0,008759
5	1	0,406149	0,181314	0,000525	0	1,83E-05	2,33E-06	0,022059	0,000818	0,159255
5	1	0,792539	0,068519	0,029852	0,01453	0,001112	3,28E-05	0,03062	0,007957	0,034533

5	2	0,845442	0,023158	6,33E-06	1,94E-06	0,021243	0,00032	0,007651	0,000291	0,015502
5	2	0,602798	0	0,194896	0	0,000394	0	0	0,000991	-0,1949
5	2	0,971544	1,84E-07	0,025843	0,000106	0,000141	7,58E-05	0,008165	0,007378	-0,03014
5	2	0,804837	0,000516	0,097828	0,016284	0,000822	0,001293	0,001446	0,009081	-0,10211
5	2	0,791024	0,068519	0,037178	0,001376	0,000965	0,000122	0,013978	0,00298	0,019201
5	2	0,972033	2,9E-05	0,00412	0,000864	0,011149	0	0,000407	0,00727	-0,00409
5	2	0,540043	5,63E-05	0,360977	0,038804	0,001073	0,005825	0,003615	0,001945	-0,38589
5	2	0,395175	0,235094	0,015708	0,011658	0,004834	0,018128	0,005531	0,003098	0,210516
5	2	0,638772	0	0,005912	0	0,001771	0,001065	0	0,003623	-0,00686
5	2	0,896368	0,005976	0,009685	0,012055	0,000555	0,004813	0,000488	0,001583	-0,01454
5	2	0,653657	0,026219	0,010406	6,3E-05	0,004981	0,023215	0,000208	0,042236	-0,00046
5	2	0,766138	8,49E-05	0,112756	0,001269	0,000444	0,004693	0,00098	0,000644	-0,11267
5	2	0,661965	0,00312	0,001607	0,056003	0,11023	0,019366	1,5E-08	0,002917	-0,06575
5	2	0,785175	9,27E-05	0,079607	0,037668	0,006295	0,01771	0,003553	0,007266	-0,10123
5	2	0,554968	0	0,075908	0,109864	0,013342	0,013299	0,000102	0,000894	-0,16479
5	2	0,887957	0,001262	0,001056	0,015507	0,001991	0,00684	0,001096	0,000885	-0,01908
5	2	0,836955	0,000671	0,008481	0,02698	0,005049	0,0229	0,000222	0,000515	-0,04201
5	2	0,754362	0,053937	0,000616	0,030194	0,004543	6,94E-05	1,73E-05	0,003477	0,023743
5	2	0,623282	0,046204	0,026305	0,010504	0,004938	0,044584	0,004146	0,025868	-0,01626
5	2	0,538992	0,015526	0,056066	0,002398	0,001444	0,002974	0,242591	0,000806	-0,24989
5	2	0,594484	0,000263	0,114399	0,007996	0,028713	0,001706	4,26E-05	0,002869	-0,11469
5	2	0,606007	0,001102	0,017041	0,05177	0,003671	0,018733	0,037785	0,005718	-0,06181
5	2	0,911693	0	0,027538	0,001298	0,00703	0,000651	0	0,004649	-0,02819
5	2	0,786682	0,068188	0,009063	0,000875	0,012013	0,007881	0,033687	0,005029	0,028725
5	2	0,593024	0,00697	0,007088	0,054902	0,016601	0,017906	0,021604	0,014904	-0,06473
5	2	0,44098	0,141007	0	0,088738	0,004165	0	0,035472	9E-09	0,052268
5	2	0,391465	0,013398	0,174448	0,061114	0,01618	0,011131	0,095884	0,003391	-0,22167
5	2	0,486335	0,085895	0,014235	0,017126	0,009415	0,010657	0,002043	0,000472	0,055946
5	2	0,630872	0,037079	0,000764	0,002401	0,013931	0,00356	0,017092	0,000472	0,01822
5	2	0,523099	0,133441	0,004503	0,007918	0,008301	0,057193	0,040626	0,003501	0,030556
5	2	0,900567	0,000253	0,003491	0,046121	0,003856	0,001028	0,001308	0,004755	-0,04685
5	2	0,279236	0,000281	0,573358	0,000982	0,004136	0,002878	0,003475	0,00011	-0,57582
5	2	0,891561	0,001304	0,05655	0,003356	0,000336	0,00048	0,012062	0,000844	-0,0587
5	2	0,409116	3,09E-05	0,017424	0,002459	0,000361	0,365384	0,000586	0,000174	-0,36714
5	2	0,650303	0,003131	0,006127	0,001257	0,007577	0,011061	0,002525	0,003679	-0,01524
5	2	0,525349	0,020345	0,072226	0,019591	0,000563	0,065974	0,019215	0,000747	-0,11681
5	2	0,224608	0,019178	0,012383	5,29E-05	0,000344	0,551117	0,001752	0,007599	-0,53795
5	2	0,653423	0,011222	0,091528	0,004468	0,003554	4,37E-05	0,009832	0,009972	-0,08038
5	2	0,849318	0,016746	0,01305	0,009463	0	0,000937	0,000696	0,000127	-0,00203
5	2	0,570668	0,093166	0,052537	0,002793	0,029984	0,129835	0,007111	0,012368	-0,08453
5	2	0,905484	0,000668	0,012446	0,014822	0,001857	0,000221	0,000179	0,000112	-0,0266
5	2	0,705355	0	0,008156	0,02521	8,64E-05	0	0,006022	0,001492	-0,02763
5	2	0,82781	0,022323	0,021975	0,006599	0,006344	0,006682	0,065046	0,011819	-0,05935
5	2	0,79958	0,001433	0,032657	0,003634	0,00132	0,006789	0,009744	0,00097	-0,03482
5	2	0,580954	0,001917	0,248683	0,031436	1,45E-07	0,003388	0,025569	0,000726	-0,25318
5	2	0,508804	0,283925	0,007676	0,000124	0,001279	3,17E-05	0,018636	0,002145	0,259057
5	2	0,46587	0,052126	0,016886	0,031438	0,058924	0,00088	0,00737	0,012305	0,00947
5	2	0,361625	0,235969	0,001959	0,012074	0,023756	0,004604	0,19522	0,001738	0,040749

5	2	0,541765	0,019432	0,021578	0,007252	0	1,48E-05	2E-05	0,000232	-0,0034
5	2	0,324836	0,399737	0,010224	0	0,011652	0,000147	0,003634	0,005051	0,388895
